# How is the “most bothersome symptom” construct measured across health conditions? A systematic review of patient-reported outcome measures

**DOI:** 10.1080/1179271X.2026.2732841

**Published:** 2026-09-20

**Authors:** Alice M. Mitchell, Vincent Singh Paramanandam, Dongzhe Lei, Theresa Donhauser, Niamh Waters, Cathy Hayles, Bethany Gower, Danushi Rajapakse, Eleanor Dickens, Ariane Suhood, K. Jane Chalmers, Steven J. Kamper, Claudia Cheng, Chen X. Chen, Richard B. Lipton, Martha Hickey, Marian Showell, Sarah Lensen

**Affiliations:** a School of Health Sciences, Western Sydney University, Penrith, Australia; b Department of Obstetrics, Gynaecology and Newborn Health, Royal Women’s Hospital, University of Melbourne, Melbourne, Australia; c Australian Lymphoedema Education, Research and Treatment (ALERT) Centre, Department of Health Sciences, Macquarie University, Sydney, Australia; d Department of Medical Education, Western Clinical School, University of Melbourne, Melbourne, Australia; e Institute for Social Medicine and Health Systems Research, Otto-von-Guericke-University Magdeburg, Magdeburg, Germany; f College of Medicine and Public Health, Flinders University, Adelaide, Australia; g Movement & Me Exercise Physiology, Leabrook, South Australia, Australia; h Alliance for Research in Exercise Nutrition and Activity (ARENA), Adelaide University, Adelaide, Australia; i Melbourne School of Population and Global Health, University of Melbourne, Melbourne, Australia; j Allied Health and Human Performance, Adelaide University, Adelaide, Australia; k IIMPACT in Health Research, Adelaide University, Adelaide, Australia; l School of Health Sciences, University of Sydney, Sydney, Australia; m Nepean Blue Mountains Local Health District, New South Wales, Australia; n University of Arizona College of Nursing, Tucson, Arizona, USA; o Department of Neurology, Albert Einstein College of Medicine, Bronx, New York, USA; p Department of Epidemiology and Population Health, Albert Einstein College of Medicine, Bronx, New York, USA; q Department of Obstetrics and Gynaecology, University of Auckland, Auckland, New Zealand

**Keywords:** COSMIN, patient-reported outcome, measurement properties, psychometrics, bother, symptoms

## Abstract

**Objective:**

Patient-reported outcome measures (PROMs) capture patients’ experiences and perspectives of symptoms. One approach involves measuring the “most bothersome symptom,” an outcome recommended in some regulatory guidance and core outcome sets. This systematic review aimed to identify and characterize PROMs to measure the “most bothersome symptom,” describe available measurement properties, and examine terminology used to describe “bother.”

**Methods:**

This review was registered in PROSPERO (CRD42022210384). A systematic search of EMBASE, MEDLINE, PsycINFO, and PROQOLID was conducted. Reviewers independently screened and extracted data on PROMs that directly measured the “most bothersome symptom” or indirectly assessed symptom bothersomeness. PROMs using related terminology (e.g. troublesome, distressing) were included. PROMs directly measuring the “most bothersome symptom” were evaluated using COSMIN and modified GRADE criteria.

**Results:**

In total, 270 articles describing 171 PROMs were included. Most PROMs (n = 135) indirectly captured bothersomeness through symptom scoring. Terminology inconsistencies were common; among 130 PROMs measuring “bother,” authors also used “distress” (10%), “severity” (8%), and “burden” (6%). Fourteen PROMs directly measured the “most bothersome symptom,” with only three demonstrating adequate content validity.

**Conclusions:**

Limited high-quality evidence supporting the measurement properties of PROMs designed to directly assess this construct, particularly content validity, restricts interpretability. More rigorous PROM development is required.

## PLAIN LANGUAGE SUMMARY

There is growing interest, both in research settings and clinical practice, in measuring the “most bothersome symptom.” Recently, two independent international consensus groups recommended measuring this outcome in all interventional clinical trials for endometriosis and adenomyosis. Additionally, the United States Food and Drug Administration has recommended measuring the “most bothersome symptom” as an outcome in drug trials for menopause and the acute treatment of migraine. Despite these recommendations, no guidelines exist on how to appropriately measure the “most bothersome symptom.” This review aimed to identify the different questionnaires, otherwise known as patient-reported outcome measures (PROMs), available for any health condition in which the “most bothersome symptom” is measured. The PROMs were characterized and the measurement properties of eligible PROMs were evaluated (i.e. validity, reliability, and responsiveness). The review found 171 PROMs that were capable of measuring the “most bothersome symptom.” However, only 14 directly assessed this outcome; that is, they included a specific question asking for the “most bothersome symptom” rather than deriving it from multiple symptom scores. These 14 PROMs were assessed for their measurement properties. Overall, the quality of evidence for their measurement properties was poor. Therefore, it is unknown whether the PROMs are suitable for measuring the “most bothersome symptom.” There is considerable variation in how researchers have enquired about and measured the “most bothersome symptom,” and the outcome has been assessed in a range of conditions. Further research is needed to establish the validity of many of these measures.

## 1. Introduction

There is growing recognition that healthcare interventions should target improvement in outcomes that are relevant and meaningful to patients (rather than clinicians or researchers), and that research trials should measure and prioritize these outcomes.^1^ This position acknowledges that patients are the experts of their own experiences of living with a condition and its associated symptoms.^2^^,^^3^ As such, patient-reported outcome measures (PROMs) have increasingly been used in clinical and research settings, with most taking the form of questionnaires or surveys. PROMs, which are self-reported measures by design, are now recognized as the gold standard approach for assessing symptoms.^4^ This is reflected internationally, with several major drug regulatory authorities endorsing the use of PROMs in clinical trials, including in the United States,^5^ European Union,^6^ and Australia.^7^

The “most bothersome symptom” (MBS) is an outcome that is measured in conditions with diverse symptoms.^8^ This outcome is valuable as it captures symptoms that matter most to an individual, enabling more person-centered research and care. Measuring the MBS outcome may be particularly useful in conditions where symptom presentation is heterogeneous and how bothersome a symptom is can vary between patients. In clinical trials, the MBS outcome can serve as a primary endpoint, reducing the need for multiple symptom-specific comparisons and improving statistical power when less common symptoms are being evaluated. Two international consensus groups have recently prioritized measuring the MBS as part of core outcome sets for endometriosis^9^ and adenomyosis^10^ clinical trials. Measuring the MBS has been endorsed by the United States Food and Drug Administration (FDA) in drug trials for treatments of vulvovaginal atrophy associated with menopause^11^ and acute migraine.^12^ However, beyond these conditions, it is unknown which other health areas measure this outcome and if PROMs to assess it have been developed rigorously and evaluated.

To date, no systematic review has comprehensively identified and evaluated PROMs to measure the MBS across health conditions, Consequently, it remains unclear how the MBS has been operationalized and whether available instruments demonstrate adequate measurement properties. Terminology used by PROM developers to describe the MBS and related constructs is also unclear. Inconsistencies have already been noted in the FDA guidance documents, where the MBS is assessed using a “severity” scale.^11^^,^^12^ Furthermore, PROMs may measure the MBS either directly by asking respondents to nominate their most bothersome symptom, or indirectly by inferring the MBS from ratings of multiple symptoms. The extent to which these approaches have been developed and psychometrically evaluated is currently unknown. The primary aim of this systematic review was to identify and describe PROMs measuring the MBS of any health condition, including the intended populations, recall periods, and item formats. The secondary aims were to evaluate all available measurement properties of these PROMs, and to identify definitions and synonyms used to describe the term “bother” within the included PROMs and articles. Through this review, we hoped to identify PROMs that are appropriate to measure this outcome, highlight limitations of current PROMs, and guide future studies to improve existing measurement instruments.

## 2. Methods

This systematic review was reported in accordance with the Preferred Reporting Items for Systematic Reviews and Meta-Analyses (PRISMA)-COSMIN guideline^13^ (Appendix 1) and conducted following the COSMIN guideline for systematic reviews.^14^ A review protocol was developed by a team with expertise in symptom research and systematic reviews. It was prospectively registered on Open Science Framework (osf.io/db3av) on 14 January 2022 and on PROSPERO (CRD42022210384) on 11 February 2022.

### 2.1. Eligibility criteria

Since both PROMs and articles were included in the review, separate eligibility criteria were applied. PROMs measuring the MBS, irrespective of the health condition, were included if they met all of the following criteria: (a) the question(s) included the term “bother” or a synonym (specifically: “trouble,” “worry,” “annoy,” “upset,” “burden,” “distress”); (b) it had an associated scale or rating component (e.g. a scale measuring the degree of symptom bother, frequency, severity, etc.); (c) it asked for the specific nomination of the MBS from either a pre-specified list or through free-text, or it inquired about at least two different symptoms (related to the same health condition) so that the MBS could be determined based on relative scoring; (d) the question(s) relating to the MBS must have been answered by the participant/patient or their proxy; and (e) the MBS questions may or may not have been part of a larger instrument that measured other outcomes. PROMs were excluded if they assessed the “most bothersome condition” instead of the MBS.

Studies describing the development of an eligible PROM or the evaluation of any of its measurement properties were included. If no development or validation studies existed for an eligible PROM, a single representative article that used and described the PROM in its methods was included. The representative article was selected if it was the first one identified during screening, as sorted by “relevance” on Covidence. This approach allowed us to identify and describe as many existing PROMs measuring the MBS as possible, even those which had not undergone robust development or validation. Non-English articles were excluded as language translation may have impacted the interpretability of the results.

For the purpose of organizing the results of this review, PROMs were categorized as follows: “Direct”—those with a single item specifically inquiring about the MBS (e.g. “Which of the following symptoms is your MBS?”); “Indirect”—PROMs that request individuals to rate the bother associated with multiple symptoms, such that the symptom with the highest score can be inferred as the MBS; “Assessed”—those that have been rigorously developed and/or evaluated for one or more psychometric property; and “Unassessed”—PROMs that have not undergone such evaluation. We considered the “Direct + Assessed” PROMs of greatest relevance to the review question.

### 2.2. Search strategy

A sensitive, systematic database search was undertaken in MEDLINE (1946–10 January 2022, Ovid platform), EMBASE (1980–10 January 2022, Ovid platform), PsycINFO (1806–10 January 2022, Ovid platform) and PROQOLID (searched to 10 January 2022) (Appendix 2). An updated search was conducted in MEDLINE, EMBASE, and PsycINFO in May 2026 and was updated to improve precision and focus on studies reporting the development, validation, or other measurement properties of PROMs measuring the MBS, which were the primary focus of this review. For all searches, we limited the searches to English language articles only. The search strategy was developed using both keywords and controlled vocabulary taken from the measurement instruments (i.e. survey, questionnaires, and scales), the outcomes of interest (i.e. bother, trouble, worry, annoy, upset, burden, and distress), in any population or health condition, and measurement properties from the COSMIN taxonomy. Additional searches were performed in Google Scholar (first 100 references), Google (first 150 hits), and reference lists of included articles. Conference abstracts were included; however, they were replaced by the corresponding full text if it was discovered.

A manual search for the original development article was undertaken, and where identified, it replaced the “representative” article identified in the initial search. A secondary search using the COSMIN filter^15^ was conducted for all eligible PROMs found in the initial search that were identified as being Direct PROMs, to identify development and/or validation studies. This search was run on MEDLINE, EMBASE, and PubMed. If validation studies were not identified in this secondary search, we contacted the authors of articles using each PROM for further information about any development or validation processes.

### 2.3. Selection of articles

Screening was performed by two independent reviewers using Covidence^16^ in two stages: titles and abstracts, and full text articles. AM screened all articles at both stages, while the remaining reviewers (DL, CH, BG, DR, ED, VP, and AS) conducted the parallel screening. Disagreements were resolved by discussion or an independent third reviewer (SL) when required. Reasons for changes to the study protocol regarding the selection of articles can be found in Appendix 3.

### 2.4. Extraction of PROM characteristics and study characteristics

Extraction of PROM characteristics was completed independently by two authors (AM and either DL, CH, BG, DR, or AS) using a blinded Microsoft Excel^17^ sheet. Data were extracted for all PROMs regardless of psychometric evaluation status, and included the intended population or health condition, construct, scale type and wording, recall period, open- or closed-ended item lists, number of symptoms measured, and method of completion. Where applicable, we also collected any synonyms for “bother” in the original development article or paper that described the instrument in the words of the original authors. Additional PROM-specific information was extracted for all Assessed PROMs (both Direct and Indirect), including the official name of the PROM, the PROM’s abbreviation, instructions or question wording, and average time to complete.

For all Direct + Assessed PROMs, additional study-level characteristics including the sample size (and proportion of females), age of participants, health conditions, country, language, and setting were extracted by AM and VP.

### 2.5. Methodological quality assessment of PROMs

The methodological quality of only the Direct + Assessed PROMs was evaluated in three stages. Indirect + Assessed PROMs were not evaluated for their methodological quality because their design does not explicitly capture the MBS as a distinct outcome but rather infers it from multiple symptom rankings. Although these PROMs could determine the MBS, this was not their intended purpose during development. Therefore, the review team decided to evaluate the measurement properties of only the Direct + Assessed PROMs, which were developed specifically to measure the MBS.

#### 2.5.1. Risk of bias assessment

The methodological quality was first evaluated using the COSMIN Risk of Bias checklist.^14^ Two reviewers independently completed the ratings (AM and either VP or TD or NW). Disagreements were resolved through discussion or with another member of the team (VP or TD) where required. The checklist assesses nine measurement properties which were rated as “very good,” “adequate,” “doubtful,” or “inadequate.” The “worst score counts” method was used, with the lowest rating for each standard of the measurement property forming the overall rating for each property.^14^ Those with low-quality ratings were considered to have the potential for a high risk of bias.

#### 2.5.2. Rating of measurement properties

The measurement properties of each study were evaluated using the criteria for good measurement properties.^14^ The measurement properties were assessed in duplicate, with AM completing all ratings and VP and TD completing the duplicate assessment between them. Disagreements were resolved through discussion or through a third reviewer (VP or TD). Measurement properties either: met criteria and were rated as “sufficient” (+), did not meet criteria and were rated as “insufficient” (-), or lacked information to determine sufficiency or insufficiency and were rated as “indeterminate” (?). Results from multiple studies reporting on the same measurement property (including development and content validity) for a PROM were qualitatively summarized.^14^ Whenever studies reporting the same measurement properties were rated differently (i.e. one with a “sufficient” rating and another with an “insufficient” rating), the reason for the inconsistency was explored. Where inconsistencies arose, we used ratings from the low-risk studies, ignoring those with high risk of bias. If there were inconsistencies between two or more studies with similar risk of bias, we considered possible explanations for this, such as differences in population or context, and rated them separately. If unresolved, the overall rating was deemed to be “inconsistent” (+/-).

#### 2.5.3. Grading the quality of evidence

Finally, the modified Grading of Recommendation, Assessment, Development, and Evaluation (GRADE) criteria were applied to evaluate the overall quality of the evidence, and were graded as “high,” “moderate,” “low,” or “very low.”^14^ Each measurement property was initially evaluated as “high” and was downgraded according to the following factors: (1) risk of bias due to methodological quality of the studies, (2) small sample size leading to imprecision, (3) indirectness of the result (i.e. evidence which has come from populations different from those for which the PROM was developed and where content validity had not been established). We did not grade the overall quality if the summarized measurement property was deemed to be “inconsistent” (+/-) or “indeterminate” (?).

## 3. Results

### 3.1. Search results

**Figure 1.**
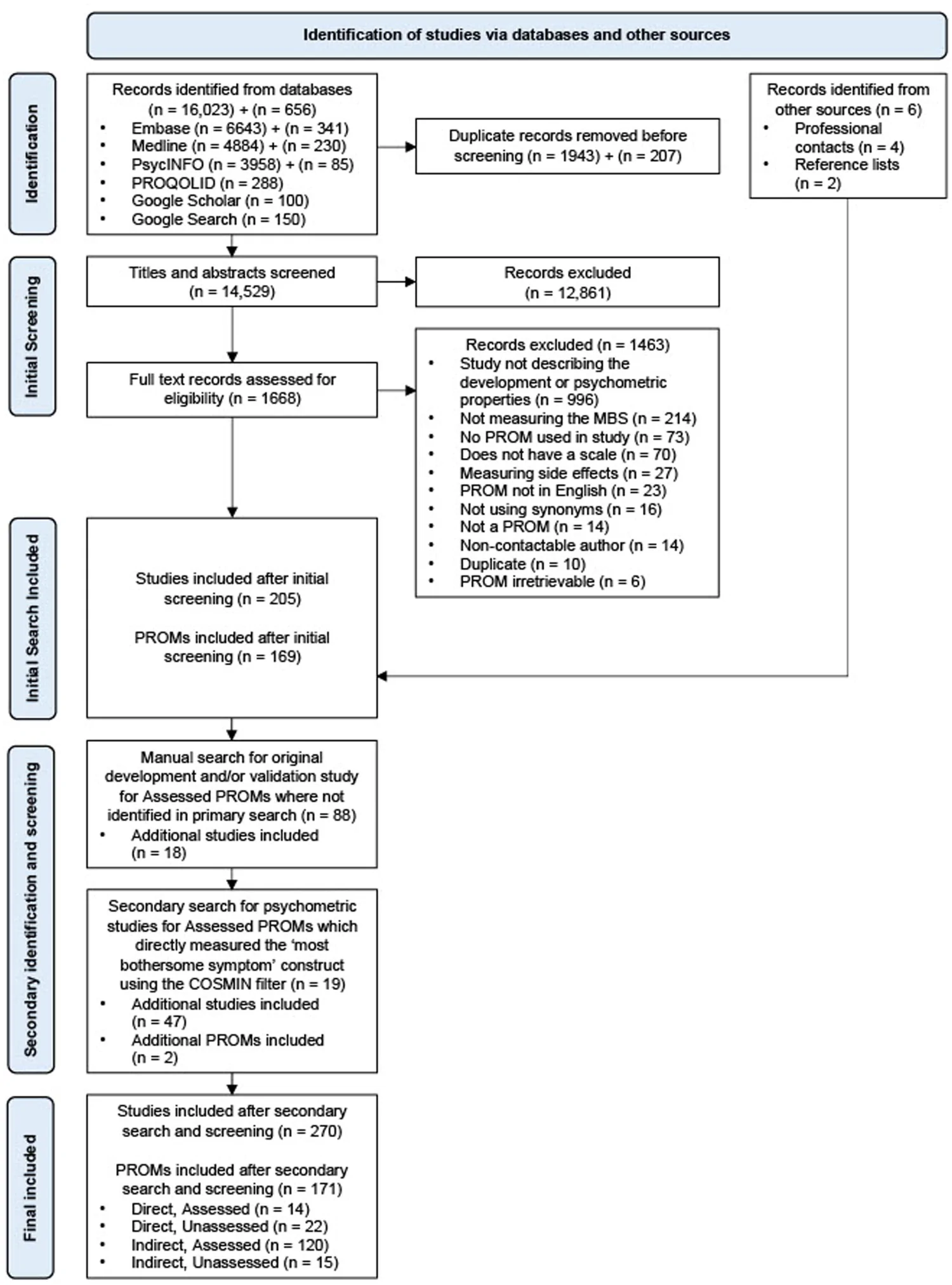
PRISMA flow chart of study selection.

After the removal of duplicates, 14,529 articles were screened. A total of 270 full texts relating to 171 PROMs were included (Figure 1). Six records were identified through professional contacts or reference lists, and 18 additional articles were included through a manual search for the original development and/or validation study where not identified in the primary search. The search using the COSMIN filter for the Direct + Assessed PROMs resulted in the inclusion of 48 additional articles and two PROMs.

### 3.2. Patient-reported outcome measures (PROMs)

A total of 171 PROMs were included in the review (Table 1). Most were Indirect (*n* = 135, 79%) and Assessed (*n* = 134, 78%). Of the Direct PROMs, only 14 (39%) had been rigorously developed and/or assessed for various measurement properties. The PROMs measured conditions of 17 distinct body systems or types of diseases (Table S1). The most common were related to conditions involving the urinary system (20%), reproductive system (14%), and cancer (11%). Most of the Direct + Assessed PROMs were developed for conditions of the urinary system (43%) and just over a fifth (21%) for generic use in any population or health condition.

**Table 1.** Overview of included PROMs.

		Assessed PROMs (*n* = 134)	Unassessed PROMs (*n* = 37)
Direct PROMs (*n* = 36)	Most bothersome symptom	10	18
	Most troublesome symptom	4	2
	Most annoying symptom	–	2
Indirect PROMs (*n* = 135)	Bother	87	15
	Trouble	13	–
	Distress	11	–
	Burden	1	–
	Bother and distress	7	–
	Bother and trouble	1	–

#### 3.2.1. Direct + assessed PROMs

Fourteen Direct + Assessed PROMs were identified. See Table 2 for a summary of their characteristics and Table S2 for the full data extracted. Ten PROMs asked about the most “bothersome” symptom and four asked about the most “troublesome” symptom. Five PROMs (MQOL,^18^ MYMOP1,^19^ MYMOP2,^20^ PSYCHLOPS,^21^ QUAL-E^22^) were developed for use in patients with more than one specific condition. The MYMOP1^19^ and MYMOP2^20^ were generic and could be used in any health condition, and the PSYCHLOPS^21^ was designed for any psychological condition. Recall periods ranged from the “Precise moment you fill in the questionnaire” to “3 months.” Over half (*n* = 8, 57%) contained open-ended fields for patients to self-nominate symptoms. Of those that used closed-ended item lists, two allowed respondents to optionally nominate an additional symptom through an open-ended field.

**Table 2.** Summary characteristics of direct + assessed PROMs (*n* = 14).

PROM	Construct measured	Population or health condition	Scale type, construct measured, and wording	Recall period	Open- or closed-ended items
Genitourinary Syndrome of Menopause Symptoms and Vaginal Treatments Acceptability Questionnaire (GSM-SVTAQ)^23^	Most bothersome symptom	Menopause	5-point Likert (bother): 1 Almost always or always, 2 Most times (more than half the time), 3 Sometimes (about half the time), 4 A few times (less than half the time), 5 Almost never or never	3 months	CE
McGill Quality of Life Questionnaire (MQOL)^18^	Top 3 most troublesome symptoms	Life-threatening illnesses	7-point Likert (problem): 1 no problem, 2, 3, 4, 5, 6, 7 tremendous problem	2 days	OE
Male Urogenital Distress Inventory (MUDI)^24^	Most bothersome symptom	Male urinary incontinence	4-point Likert Type (bother): Not at all, Slightly, Moderately, Greatly	4 weeks	CE + 1 optional OE
Measure Yourself Medical Outcome Profile (MYMOP1)^19^	Top 2 most bothersome symptoms	Any population or condition	7-point Likert (good/bad): 0 As good as it could be, 1, 2, 3, 4, 5, 6 As bad as it could be	1 week	OE
Measure Yourself Medical Outcome Profile – Revised version (MYMOP2)^20^	Top 2 most bothersome symptoms	Any population or condition	7-point Likert (good/bad): 0 As good as it could be, 1, 2, 3, 4, 5, 6 As bad as it could be	1 week	OE
Non-Motor Fluctuations Severity Scale (NMF2S)^25^	Most bothersome symptom	Parkinson’s disease	11-point NRS (intensity): 0, 1, 2, 3, 4, 5, 6, 7, 8, 9, 10	Precise moment you fill in the questionnaire	CE
Primary Overactive Bladder Symptom Questionnaire (POSQ)^26^	Most bothersome symptom	Overactive bladder	5-point Likert (bother): 1 Not at all, 2 A little, 3 Moderately, 4 A great deal, 5 A very great deal	2 weeks	CE
Psychological Outcome Profiles (PSYCHLOPS)^21^	Top 2 most troublesome symptoms	Any psychological condition	6-point Likert Type (affect): 0 Not at all affected, 1, 2, 3, 4, 5 Severely affected 5-point Likert (length of time concerned): Under one month, Between one and three months, Over three months but under one year, One to five years, Over five years	NR	OE
			6-point Likert Type (affect): 0 Not at all affected, 1, 2, 3, 4, 5 Severely affected	1 week	
			6-point Likert Type (affect): 0 Not at all affected, 1, 2, 3, 4, 5 Severely affected	1 week	
Questionnaire for female lower urinary tract symptoms and their impairment of quality of life^27^	Most troublesome symptom	Females with lower urinary tract dysfunction	4-point Likert Type (problem): No, Yes but only a small problem, Yes it is a problem, Yes it is a serious problem	1 month	CE
Quality of Life at the End of Life (QUAL-E)^22^	Most bothersome symptom	Cancer, congestive heart failure, COPD, end-stage renal disease	5-point Likert (frequency): 1 Rarely, 2 A few times, 3 Fairly often, 4 Very often, 5 Most of the time 5-point Likert (importance): 1 Not at all, 2 A little bit, 3 A moderate amount, 4 Quite a bit, 5 Completely 5-point Likert (interference): 1 Not at all, 2 A little bit, 3 A moderate amount, 4 Quite a bit, 5 Completely 5-point Likert (severity): 1 Very mild, 2 Mild, 3 Moderate, 4 Severe, 5 Very severe 5-point Likert (worry): 1 Not at all, 2 A little bit, 3 A moderate amount, 4 Quite a bit, 5 Completely	1 week	OE
Quality of Life at the End of Life – Cancer (QUAL-EC)^28^	Most bothersome symptom	Advanced cancer	5-point Likert (frequency): 1 Rarely, 2 A few times, 3 Fairly often, 4 Very often, 5 Most of the time 5-point Likert (interference): 1 Not at all, 2 A little bit, 3 A moderate Amount, 4 Quite a bit, 5 Completely 5-point Likert (severity): 1 Very mild, 2 Mild, 3 Moderate, 4 Severe, 5 Very severe	1 week	OE
Short-Form Leeds Dyspepsia Questionnaire (SF-LDQ)^29^	Most troublesome symptom	Dyspepsia	5-point Likert (frequency): Not at all, Less than once a month, Between once a month and once a week, Between once a week and once a day, Once a day or more 5-point Likert (interference): Not at all, Less than once a month, Between once a month and once a week, Between once a week and once a day, Once a day or more	2 months	CE
Urogenital Distress Inventory (UDI)^30^	Most bothersome symptom	Women with urinary incontinence	4-point Likert Type (bother): 0 Not at all, 1 Slightly, 2 Moderately, 3 Greatly	NR	CE + 1 optional OE
Urge Urinary Distress Inventory (U-UDI)^31^	Most bothersome symptom	Urge urinary incontinence or mixed urinary incontinence with a primary urge component	4-point Likert Type (bother): Not at all, Slightly, Moderately, Greatly	4 weeks	CE

Abbreviations. CE, closed-ended; COPD, chronic obstructive pulmonary disease; NR, not reported; NRS, numerical rating scale; OE, open-ended; SA, self-administered.

#### 3.2.2. Direct + unassessed PROMs

There were 22 Direct PROMs that had not undergone formal development or psychometric evaluation. These PROMs measured the most “bothersome” symptom (82%), the most “troublesome” symptom (9%), and the most “annoying” symptom (9%). The characteristics of these PROMs varied depending on their purpose and intended population (Table S3–Table S6). Most were questionnaires or phone surveys that were purposefully constructed for use in a single study. Two were identified as part of guidance documents published by the United States FDA for drug trials related to menopause^11^ and acute migraine.^12^ The majority did not report a recall period (77%) and most were self-administered (67%). One study involving patients with dementia receiving palliative care collected data through a proxy (5%). Closed-ended lists of symptoms (45%) were common, with only eight (26%) including free-text fields.

#### 3.2.3. Indirect PROMs

Most of the PROMs identified in this review could indirectly measure the MBS by rating multiple symptoms (*n* = 135), allowing for relative score comparison. Of these, 120 were formally developed (Table S7–Table S12) and 15 were not (Table S13). The Indirect PROMs mostly measured the degree of “bother” (76%), “trouble” (10%), and “distress” (8%) caused by symptoms (Table 1). Although characteristics were diverse (Table S14), the most common reported recall periods were “1 week” (18%), “4 weeks” (13%), and “7 days” (13%). All but one PROM used a closed-ended list of symptoms, with 19 (14%) allowing additional symptoms through optional free-text fields. Most were designed to be self-administered (97%).

#### 3.2.4. Bother and its synonyms

Of all included PROMs, 130 (76%) measured the degree of “bother” caused by symptoms (Table 1). PROMs measuring the amount of “trouble” (*n* = 19, 11%) and “distress” (*n* = 11, 6%) were the next most common. No PROMs measured “worry” or “upset.”

Although consistent language was used to describe the construct measured in most PROMs (i.e. a PROM that measured the most “bothersome” symptom consistently used the term “bother”), some variation was observed. Table S15 contains all the synonyms used in the PROMs themselves, as sorted by the construct measured. For example, among the PROMs that measured the construct of “bother,” 14 synonyms were used, with “distress” (*n* = 7) and “impact” (*n* = 2) being the most common. These synonyms were often found in the PROM’s instructions or the rating scales. Of the ten Direct + Assessed PROMs that measured the most “bothersome” symptom, five used rating scales with different terms. For example, the QUAL-E^22^ used various scales, one of which was a “frequency” scale ranging from “Not at all” to “Most of the time.”

Eight of the Indirect PROMs used interchangeable terminology for two constructs in either their questions or scale wording, with six asking about both “bother” and “distress” caused by symptoms. For example, the Memorial Symptom Assessment Scale (MSAS)^23^ and its adaptations ask individuals to consider “How much did (the symptom) distress or bother you?.” The titles of some PROMs also differed according to which construct was being measured. For instance, the Male Urogenital Distress Inventory (MUDI),^24^ despite being titled as a PROM that measures “distress,” asked about symptoms that were “bothersome.”

Within the articles themselves, authors were less likely to use consistent language when describing the construct measured by their PROMs (Table S16). For example, of the 130 PROMs that measured “bother,” only 111 (85%) described their PROMs using the same wording in the published articles. “Distress” (*n* = 13), “severity” (*n* = 11), and “burden” (*n* = 9) were the most common synonyms used. Among the seven PROMs that measured the degree of “bother and distress,” all seven associated articles used the term “distress,” but only one also used “bother” when describing the PROM.

### 3.3. Measurement properties

#### 3.3.1. Study characteristics

After completing the secondary search for relevant papers using the COSMIN filter, 60 studies were found that evaluated the measurement properties of the 14 Direct + Assessed PROMs (Table S17). Overall, 12,689 participants were included (range: *n* = 4–*n* = 2239) and most were female (62%). Studies were most frequently conducted in the United Kingdom (23%), United States of America (18%), and Canada (12%).

#### 3.3.2. Measurement properties

Eight measurement properties were assessed for the 14 PROMs. Table 3 provides a summary of the measurement properties, using a traffic light system to indicate the certainty of the results. Green represents sufficient measurement property ratings that are supported by moderate-to-high quality evidence, amber indicates uncertainty of results and the need for further evaluation, and red signifies insufficient measurement property ratings that are supported by moderate-to-high quality evidence. The most frequently evaluated properties were content validity (*n* = 13) and construct validity (*n* = 10). PROMs that had the greatest number of measurement properties evaluated were the MQOL and UDI (*n* = 7). Cross-cultural validity could not be rated due to a lack of measurement invariance assessment in the included studies,^14^ despite 25 reporting translation and validation of PROMs in languages other than English (Table S17). For complete risk of bias, content validity, and other measurement property assessments, please refer to Appendices 4–6, respectively.

**Table 3.** Summary of findings of the measurement properties of the included direct + assessed PROMs.

PROM	Content validity	Structural validity	Internal consistency*	Reliability	Measurement error	Criterion validity	Hypothesis testing (construct validity)	Responsiveness
GSM-SVTAQ	+(H)		?(NG)					
MQOL	+(M)^a^	-(VL)	?(NG)	+(L)	?(NG)		+(H)	+(H)
MUDI	+(L)	?(NG)	?(NG)				?(NG)	+(L)
MYMOP1	±(NG)						+(H)	+(H)
MYMOP2	±(NG)						+(H)	+(M)
NMF2S	+(VL)	+(H)						+(H)
POSQ				+(VL)				
PSYCHLOPS	+(VL)	+(H)	+(H)	+(L)			+(H)^c^	+(H)
Q Female LUTS	+(VL)			?(NG)				
QUAL-E	+(L)	?(NG)	?(NG)	±(L)			+(M)	
QUAL-EC	+(M)	-(H)	?(NG)				+(M)	
SF-LDQ	+(VL)		?(NG)	+(L)		+(H)	+(H)	+(M)
UDI	+(VL)^b^	?(NG)	?(NG)	-(M)		±(NG)	+(H)	+(H)
U-UDI	+(VL)	?(NG)	?(NG)	+(M)			+(H)	+(H)

+, sufficient rating; -, insufficient rating; ±, inconsistent rating;?, indeterminate rating; (), grading of overall quality of evidence based on modified GRADE approach. Green denotes measurement criteria that have sufficient rating and are of moderate-to-high quality; Amber denotes measurement properties that are limited by low or very low quality of evidence or are not graded; Red denotes measurement properties which are insufficient and are of a moderate-to-high quality; Empty cells denote measurement properties that were not assessed.

Abbreviations. H, high; A, adequate; M, moderate; L, low; VL, very low; NG, not graded; GSM-SVTAQ, Genitourinary Syndrome of Menopause (GSM) Symptoms and Vaginal Treatments Acceptability Questionnaire; MQOL, McGill Quality of Life Questionnaire; MUDI, Male Urogenital Distress Inventory; MYMOP 1, Measure Yourself Medical Outcome Profile; MYMOP2, Measure Yourself Medical Outcome Profile—Revised version; NMF2S, Non-Motor Fluctuations Severity Scale; POSQ, Primary OAB Symptom Questionnaire; PROM, patient-reported outcome measure; PSYCHLOPS, Psychological Outcome Profiles; Q Female LUTS, Questionnaire for female lower urinary tract symptoms and their impairment of quality of life; QUAL-E, Quality of Life at the End of Life; QUAL-EC, QUAL-E Cancer; SF-LDQ, Short Form Leeds Dyspepsia Questionnaire; UDI, Urogenital Distress Inventory; U-UDI, Urge Urinary Distress Inventory.

* Internal consistencies were rated as “indeterminate” in the absence of sufficient structural validity.

^a^ Content validity only scored from studies with English participants (source language) studies. MQOL was translated into Chinese, Dutch, Italian, Korean, and Taiwanese, and all scored VL.

^b^ Content validity rating comprised of English (USA) study and only comprehensibility for Italian (Italy) study.

^c^ Not all studies were of high quality, however 3 were very good and 2 were adequate.

Three PROMs were rated as having sufficient content validity and supported by moderate quality of evidence (GSM-SVTAQ,^25^ MQOL,^18^^,^^26–29^ QUAL-EC.^30^) Eight PROMs were rated as sufficient but were limited by low (MUDI^24^ and QUAL-E^22^^,^^31^) or very low (NMF2S,^32^ PSYCHLOPS,^21^^,^^33^ Questionnaire for female lower urinary tract symptoms and their impairment of quality of life,^34^ SF-LDQ,^35^ UDI,^36^^,^^37^ and U-UDI^38^) quality of evidence. Three PROMs had inconsistent or no evidence of content validity (MYMOP1,^19^^,^^20^^,^^39^^,^^40^ MYMOP2,^20^^,^^39^ and POSQ^41^).

Of the eight PROMs assessed for structural validity, only the NMF2S,^32^ and PSYCHLOPS^42^ had sufficient and high-level evidence. The majority of PROMs assessed for structural validity could not be graded due to an indeterminant rating (MUDI, QUAL-E, UDI, U-UDI). Despite overall high Cronbach’s alpha scores (≥0.70) for internal consistency among the PROMs, most were not graded due to lack of established structural validity.^43^ The PSYCHLOPS^42^^,^^44–50^ was the only PROM rated as sufficient and of high quality for internal consistency.

For reliability and criterion validity, the U-UDI and the SF-LDQ were the only properties rated as sufficient and of a moderate or high quality of evidence, respectively. Overall, the evidence for construct validity through hypothesis testing and responsiveness was sufficient and of moderate or high quality for all those assessed apart from the MUDI. Evidence for the MUDI was limited by the lack of evaluation of the individual subscales.

## 4. Discussion

This review identified and evaluated PROMs measuring the MBS, revealing substantial variation in their characteristics and measurement properties. A total of 171 PROMs were identified, highlighting widespread interest in assessing “bothersome” symptoms. However, quality varied, with only 14 directly measuring the MBS and having undergone psychometric assessment.

A key finding was the lack of strong evidence to support content validity among the 14 PROMs that directly measured the MBS and had undergone psychometric evaluation. Content validity, which is arguably the most crucial psychometric property,^51^^,^^52^ was sufficient and of moderate quality for only the GSM-SVTAQ,^25^ MQOL,^18^^,^^26–29^ and QUAL-EC.^30^ Therefore, these three PROMs show the most promise for measuring the MBS. The lack of strong evidence for content validity in the remaining PROMs is concerning given their widespread use in clinical practice and research trials. A PROM’s ability to accurately reflect symptom experience is essential, otherwise it may lead to the provision of misguided interventions. While content validity was sufficient for all but three of the 14 PROMs, the supporting evidence was of limited quality. This was often due to low numbers of studies assessing content validity and inadequate involvement of patients in determining the relevance, comprehensiveness, and comprehensibility of the PROMs. Future studies should aim to integrate robust methodologies, such as cognitive interviewing, and actively involve patients to refine and validate these instruments. This is particularly important considering “bother” was recently found to not be appropriate in an endometriosis population, as the term significantly downplays the symptom experience.^53^

The inconsistent use of “bother” was another notable finding. Among the 130 (76%) PROMs measuring “bothersome” symptoms, only 111 explicitly used this term in the associated publication. Within the PROMs themselves, seven were even found to measure both “bother” and “distress” within the same items. Inconsistent terminology suggests that PROM developers may view these constructs as interchangeable when describing symptom experiences. However, these terms may not be conceptually equivalent. Existing literature has conceptualized “bother” in various ways, including the extent of worry and burden caused by a condition,^54^ the degree of difficulty associated with a symptom,^55^ its perceived importance,^56^ and its impact on daily activities.^57^ In contrast, while “distress” also lacks a universally accepted definition,^58^ it is generally considered to reflect broader emotional or psychological responses to symptoms.^59^ This distinction may be particularly important in chronic conditions such as endometriosis, where symptoms can have substantial emotional and psychological consequences, and the term “bother” has been reported to minimize symptom experience.^53^ Furthermore, in an advanced cancer population, the “severity” and “bothersomeness” of symptoms were found not to correlate.^60^ Despite this, 10 studies in this review described PROMs measuring “bothersome” symptoms as measures of symptom “severity,” suggesting that the concepts of bother and severity are treated as interchangeable. Without a clear conceptual definition of “bother,” the clinical and research utility of many MBS assessments is undermined, making cross-study comparisons challenging. Establishing standardized terminology through stakeholder consensus, such as Delphi studies, is urgently needed to improve the validity and interpretability of MBS measurements.

Most of the PROMs directly measuring the MBS had not undergone formal psychometric evaluation, indicating a gap in rigorously developed tools with established measurement properties. While this is unsurprising given that many were created for single studies, some of these PROMs are widely used in clinical drug trials. For example, the FDA guidance documents for menopause^11^ and the acute treatment of migraine^12^ state that the MBS should be measured in all drug trials, but fail to provide a validated instrument. This is concerning considering the widespread reach of these recommendations. The absence of a standardized PROM can lead to inconsistent outcome assessment across trials, reducing data comparability. Without rigorous development and pilot-testing, PROMs developed specifically for regulatory submissions may fail to capture patient experiences accurately. Developing more robust instruments is essential to ensure meaningful assessments in clinical research. A stakeholder-led consensus process to define and operationalize the concept of “bother” would provide an important foundation for the development of standardized PROMs that align with regulatory recommendations and should be considered a research priority.

Structural validity was similarly poor among the assessed PROMs. Sample sizes were small, and studies were often limited to exploratory rather than confirmatory factor analysis. The lack of structural validity among most PROMs meant the internal consistency could not be graded according to the methodology developed by COSMIN. The PSYCHLOPS was the only PROM that could be recommended for structural validity and internal consistency (although it does have low quality evidence for content validity), underscoring the urgent need for improved psychometric evaluation of the remaining PROMs measuring this construct.

Cross-cultural validity was another critical gap among the 26 studies that used translated PROMs. Although many employed adequate translation methodologies, due to the lack of measurement invariance testing, the cross-cultural validity was not graded based on COSMIN recommendations.^14^ Without this, it is unclear whether the PROMs measure the constructs consistently across different languages. This issue is further complicated by the fact that “bother” lacks conceptual equivalence in other languages and its meaning can differ significantly across cultural contexts.^61^ Such variability challenges the global applicability of PROMs measuring the MBS and highlights the need for rigorous content validation and culturally relevant construct definitions. Future research should prioritize measurement invariance testing and incorporate cognitive interviewing with native speakers to ensure constructs are interpreted consistently across diverse populations.

The characteristics of the 171 PROMs varied considerably, likely reflecting the diverse populations and purposes they are designed for. A key difference was the recall periods, which ranged from “currently” to “one year.” Notably, among the 12 PROMs with recall periods of three months or longer, half (50%) were developed for gynecological-related conditions (Genitourinary Syndrome of Menopause (GSM) Symptoms and Vaginal Treatments Acceptability Questionnaire,^25^ EndoWheel,^62^ Uterine Fibroid Symptom and Health-Related Quality of Life Questionnaire^63^) or female pelvic floor dysfunction (Urogenital Distress Inventory-Short Form,^64^ Pelvic Floor Distress Inventory-46,^65^ Pelvic Floor Distress Inventory-Short Form 20^66^) While shorter recall periods are generally recommended to minimize recall bias,^67^ it is interesting that most with relatively longer recall periods are related to gynecological conditions, where symptoms can fluctuate due to the menstrual cycle, necessitating a broader timeframe. Recall period is an important consideration when assessing the MBS as it may influence which symptom is identified as most bothersome. Shorter recall periods may capture symptoms that are most salient at the time of assessment, whereas longer recall periods may capture symptoms that are intermittent or have a greater cumulative effect over time. Specifically for conditions characterized by symptom fluctuation, such as those influenced by the menstrual cycle, the symptom identified as the most bothersome may vary according to the timing of assessment, potentially affecting the consistency of measurement.

This review examined the measurement properties of PROMs that directly measured the MBS and had available psychometric evidence, which may limit the generalizability of the findings. The decision to focus on these PROMs was driven by the need to evaluate instruments specifically designed to measure the MBS. While the majority (79%) of the included PROMs were Indirect, these instruments may not have been developed for this purpose. It may be challenging to use these PROMs to measure the MBS if two or more symptoms are scored equally. Consequently, using these PROMs may result in a less tailored assessment, as patients may struggle to determine which symptom is the “most bothersome” if symptoms are scored the same. However, the psychometrically evaluated PROMs that inferred the MBS from symptom ratings still provide valuable insights, and future research should explore their potential utility in MBS measurement.

To our knowledge, this is the first review to systematically evaluate PROMs measuring the MBS. This study adhered to PRISMA-COSMIN guidelines, used a comprehensive search strategy, and employed robust methodology, including duplicate screening and data extraction. Eligible PROMs were assessed using the COSMIN criteria and a modified GRADE approach to appraise evidence quality.^14^ However, this review is not without limitations. We acknowledge that updated COSMIN guidelines have been published (August 2024) since the completion of this review.^43^ However, due to the substantial size of this review, it was not feasible to update the methods in light of the new guidelines. Additionally, we do not anticipate that the updated guidelines would have significantly altered the outcomes of this review. Non-English studies were excluded in view of the review team being English-speaking and lacking the resources to translate studies published in other languages. This omission of studies in other languages may have introduced language bias. Additionally, the list of included synonyms for “bother” was not exhaustive, somewhat limiting its scope. However, this was deliberate to ensure the terms were relevant to the construct and to maintain feasibility of the review. Although the included terms were derived from existing literature^61^ and expert input, future research may benefit from a more expansive linguistic analysis to explore whether other terms share conceptual overlap with “bother.”

## 5. Conclusion

Many PROMs measuring the MBS have been poorly developed and validated, particularly regarding content validity. The lack of established content validity in most of the PROMs in this review is a concerning finding. There is an urgent need for more robust development and establishment of measurement properties to ensure PROMs accurately capture the patient’s experience. Improving the quality of existing PROMs is crucial for clinical decision-making and research evaluation. Patient-centered outcomes are valuable in identifying patient-prioritized problems and enabling appropriate treatment. However, “bother” is an ambiguous term with no universally accepted definition. With the increasing popularity of measuring this construct with PROMs, future research must clearly define “bother” and ensure that it is both applicable and well-understood by users of these instruments.

## Supplementary Material

Appendices 1 2 3_Clean.docx

Appendix 4 ROB_Clean.docx

Supplementary Tables S1 though S17_Clean.docx

Appendix 5 Content Validity_Clean.docx

Appendix 6 Measurement Properties_Clean.docx

## Data Availability

The datasets used and analyzed during the current study are available from the corresponding author on reasonable request.
