# Supplementary material for: How is the “most bothersome symptom” construct measured across health conditions? A systematic review of patient-reported outcome measures
Source: Patient Relat Outcome Meas. 2026 Sep 20;17(1):107–22. doi: 10.1080/1179271X.2026.2732841 (PMC13596887; doi:10.1080/1179271X.2026.2732841)
Supplement: Supplementary file 1 [file DPRO_A_2732841_SM9470.docx]

**Appendix 6: Measurement property ratings**

| **GSM-SVTAQ** | | | | | | | | |
| --- | --- | --- | --- | --- | --- | --- | --- | --- |
| **Reference** | **Country (language)** | **Content validity** | | | | **Internal consistency** | | |
|  |  | **Rel** | **CHV** | **CHB** | **CV** | **N** | **MQ** | **R** |
| Hassanein et al., 2024 | United Arab Emirates (English and Arabic) | + | + | + | + | 72 | VG | ? |
| **Pooled summary result (overall rating)** | | **+** | **+** | **+** | **+** | **72** | **1VG** | **?** |
| **Grade** | |  |  |  | **High** |  |  | **NG** |

Rel: relevance; CHV: comprehensiveness, CHB: comprehensibility; CV: content validity; N: number (sample); MQ: methodological quality; R: rating; VG: very good; NG: not graded; +: sufficient; ?: indeterminant

| **MQOL** | | | | | | | | | | | | | | | | | | | | | | | |
| --- | --- | --- | --- | --- | --- | --- | --- | --- | --- | --- | --- | --- | --- | --- | --- | --- | --- | --- | --- | --- | --- | --- | --- |
| **Reference** | **Country (language)** | **Content validity** | | | | **Structural validity** | | | **Internal consistency** | | | **Reliability** | | | **Measurement error** | | | **Hypothesis testing  (A & B)** | | | **Responsiveness  (A – D)** | | |
|  |  | **Rel** | **CHV** | **CHB** | **CV** | **N** | **MQ** | **R** | **N** | **MQ** | **R** | **N** | **MQ** | **R** | **N** | **MQ** | **R** | **N** | **MQ** | **R** | **N** | **MQ** | **R** |
| Cohen et al., 1995 | Canada (English, French) | ? | ? | ? | ? | 40 | I | ? (EFA) | 40 | VG | ? |  |  |  |  |  |  | 40 | VG | + |  |  |  |
|  |  |  |  |  |  |  |  |  |  |  |  |  |  |  |  |  |  | 40 | VG | - |  |  |  |
| Cohen et al., 1996 | Canada (English, French) |  | + | + | + | 247 | A | ? (EFA) | 247 | VG | ? |  |  |  |  |  |  | 247 | VG | + |  |  |  |
|  |  |  |  |  |  |  |  |  |  |  |  |  |  |  |  |  |  | 247 | I | + |  |  |  |
|  |  |  |  |  |  |  |  |  |  |  |  |  |  |  |  |  |  | 247 | A | + |  |  |  |
| Cohen, Hassan et al., 1996 | Canada (English, French) |  | + | +/- | + | 109 | A | ? (EFA) | 109 | VG | ? |  |  |  |  |  |  | 109 | I | + |  |  |  |
|  |  |  |  |  |  |  |  |  |  |  |  |  |  |  |  |  |  | 109 | A | + |  |  |  |
| Cohen et al., 1997 | Canada (English, French) |  |  |  |  | 143 | A | ? (EFA) | 143 | VG | ? |  |  |  |  |  |  | 143 | VG | + |  |  |  |
|  |  |  |  |  |  |  |  |  |  |  |  |  |  |  |  |  |  | 143 | I | + |  |  |  |
| Cohen & Mount, 2000 | Canda (English, French) |  |  |  |  |  |  |  | 100 | VG | ? | 44-59 | A | +/- |  |  |  |  |  |  | 186-189 | I | + |
|  |  |  |  |  |  |  |  |  |  |  |  |  |  |  |  |  |  |  |  |  | 66 | VG | + |
| Tsujikawa et al., 2009 | Japan (Japanese) |  |  |  |  | 83 | A | ? (EFA) | 83 | VG | ? |  |  |  |  |  |  |  |  |  |  |  |  |
| Lo et al., 2001 | Hong Kong (Chinese) |  |  |  |  | 462 | A | ? (EFA) | 462 | VG | ? | 20 | D | + |  |  |  | 462 | I | + |  |  |  |
|  |  |  |  |  |  |  |  |  |  |  |  | 20 | D | + |  |  |  | 462 | VG | + |  |  |  |
| Gofton et al., 2015 | Canda (English) |  |  |  |  | 81 | D | ? (EFA) | 81 | VG | ? |  |  |  |  |  |  | 81 | D | + |  |  |  |
| Bentur & Reszinsky, 2005 | Canda (English) |  |  |  |  | 160 | A | ? (EFA) | 160 | VG | ? |  |  |  |  |  |  |  |  |  |  |  |  |
| Hu et al., 2003 | Taiwan (Chinese) | ? |  |  | ? | 64 | I | - | 64 | VG | ? |  |  |  |  |  |  | 64 | D | + |  |  |  |
| Hu et al., 2015 | China (Chinese) |  |  |  |  | 63 | I | ? (EFA) | 126 | VG | ? | 84 | D | ? |  |  |  |  |  |  |  |  |  |
| Sguazzin et al., 2010 | Italy (Italian) |  |  | ? | ? | 175 | A | ? (EFA) | 175 | VG | ? |  |  |  |  |  |  | 175 | D | + |  |  |  |
|  |  |  |  |  |  |  |  |  |  |  |  |  |  |  |  |  |  | 175 | I | + |  |  |  |
| Kim et al., 2007 | Korea (Korean) |  |  | + | + | 140 | A | ? (EFA) | 140 | VG | ? |  |  |  |  |  |  | 140 | D | + |  |  |  |
|  |  |  |  |  |  |  |  |  |  |  |  |  |  |  |  |  |  | 140 | VG | + |  |  |  |
| Shahidi et al., 2008 | Iran (Persian) |  |  |  |  |  |  |  | 61 | VG | ? | 61 | D | ? |  |  |  | 61 | VG | + |  |  |  |
|  |  |  |  |  |  |  |  |  |  |  |  |  |  |  |  |  |  | 61 | I | + |  |  |  |
| Paratheewapat et al., 2001 | United Kingdom (English) | ? |  | ? | ? |  |  |  | 36 | VG | ? |  |  |  |  |  |  | 36 | VG | + |  |  |  |
| De Vrieze et al., 2016 | Belgium (Dutch) | + |  | + | + |  |  |  | 26 | VG | ? | 26 | I | + | 26 | I | + | 26 | D | + |  |  |  |
| Zhang et al., 2003 | China (Chinese) | ? | ? | ? |  | 562 | A | ? (EFA) | 562 | VG | ? |  |  |  |  |  |  |  |  |  |  |  |  |
| **Pooled summary result (overall rating)** | | **+** | **+** | **+** | **+** | **2329** | **9A, 1D, 3I** | **-** | **2615** | **17D** | **?** | **90-105** | **1A, 3D, 1I** | **+** | **26** | **1I** | **?** | **1584** | **8VG, 2A, 5D, 6I** | **+** | **252-255** | **1VG, 1I** | **+** |
| **Grade** | |  |  |  | **Moderate** |  |  | **Very low** |  |  | **NG** |  |  | **Low** |  |  | **NG** |  |  | **High** |  |  | **High** |

Rel: relevance; CHV: comprehensiveness, CHB: comprehensibility; CV: content validity; N: number (sample); MQ: methodological quality; R: rating; EFA: exploratory factor analysis; VG: very good; A: adequate; D: doubtful; I: inadequate; NG: not graded; +: sufficient; -: insufficient; ?: indeterminant

| **MUDI** | | | | | | | | | | | | | | | | | |
| --- | --- | --- | --- | --- | --- | --- | --- | --- | --- | --- | --- | --- | --- | --- | --- | --- | --- |
| **Reference** | **Country (language)** | **Content validity** | | | | **Structural validity** | | | **Internal consistency** | | | **Hypothesis testing  (A & B)** | | | **Responsiveness  (A – D)** | | |
|  |  | **Rel** | **CHV** | **CHB** | **CV** | **N** | **MQ** | **R** | **N** | **MQ** | **R** | **N** | **MQ** | **R** | **N** | **MQ** | **R** |
| Robinson & Shea, 2002 | United States of America (English) | + | + | + | + | 139 | A | ? (EFA) | 139 | I | ? | 139 | D | ?^*^ |  |  |  |
| Lim et al., 2009 | Korea (Korean) |  |  |  |  | 143 | A | ? (EFA) | 143 | I | ? | 143 | D | ?^*^ |  |  |  |
| Robinson et al., 2007 | United States of America (English) |  |  |  |  |  |  |  | 100 | I | ? | 100 | D | ?^*^ | 100 | D | + |
| **Pooled summary result (overall rating)** | | **+** | **+** | **+** | **+** | **282** | **2A** | **?** | **382** | **3I** | **?** | **382** | **3D** | **?** | **100** | **1D** | **+** |
| **Grade** | |  |  |  | **Low** |  |  | **NG** |  |  | **NG** |  |  | **NG** |  |  | **Low** |

Rel: relevance; CHV: comprehensiveness, CHB: comprehensibility; CV: content validity; N: number (sample); MQ: methodological quality; R: rating; EFA: exploratory factor analysis; A: adequate; D: doubtful; I: inadequate; NG: not graded; +: sufficient; ; ?: indeterminant
^*^: ratings for hypothesis testing (construct validity) were downgraded as individual subscales were not evaluated

| **MYMOP1** | | | | | | | | | | | |
| --- | --- | --- | --- | --- | --- | --- | --- | --- | --- | --- | --- |
| **Reference** | **Country (language)** | **Content validity** | | | | **Hypothesis testing  (A & B)** | | | **Responsiveness  (A – D)** | | |
|  |  | **Rel** | **CHV** | **CHB** | **CV** | **N** | **MQ** | **R** | **N** | **MQ** | **R** |
| Rosenberg et al., 2002 | Denmark (Danish) |  |  |  |  |  |  |  |  |  |  |
| Chung et al., 2010 | China (Chinese) |  |  | + |  | 272 | VG | + | 272 | VG, I | +, + |
| Paterson et al., 2000 | United Kingdom (English) |  |  |  |  |  |  |  | 64-71 | D, A, D | ?, +, + |
| Peterson & Britten, 2000 | United Kingdom (English) | ? | - | ? | ? |  |  |  |  |  |  |
| Paterson, 1996 | United Kingdom (English) |  |  |  |  | 265 | VG | + | 265 | D, A, D | ?, +, + |
| Murphy, 2018 | United Kingdom (English) |  |  | + |  |  |  |  |  |  |  |
| **Pooled summary result (overall rating)** | | **+** | **-** | **+** | **+/-** | **537** | **2VG** | **+** | **601-608** | **1VG, 2A, 4D, 1I** | **+** |
|  | |  |  |  | **NG** |  |  | **High** |  |  | **High** |

Rel: relevance; CHV: comprehensiveness, CHB: comprehensibility; CV: content validity; N: number (sample); MQ: methodological quality; R: rating; VG: very good; A: adequate; D: doubtful; I: inadequate; NG: not graded; +: sufficient; -: insufficient; ?: indeterminant

| **MYMOP2** | | | | | | | | | | | |
| --- | --- | --- | --- | --- | --- | --- | --- | --- | --- | --- | --- |
| **Reference** | **Country (language)** | **Content validity** | | | | **Hypothesis testing  (A & B)** | | | **Responsiveness  (A – D)** | | |
|  |  | **Rel** | **CHV** | **CHB** | **CV** | **N** | **MQ** | **R** | **N** | **MQ** | **R** |
| Ersoy et al., 2019 | Turkish (Turkey) |  |  |  |  | 50 | A | + | 50 | D | + |
| Herman et al., 2014 | German (Germany) |  |  |  |  | 567 | VG | ? | 341 | A | + |
| Paterson, 2004 | United Kingdom (English) |  |  |  |  |  |  |  |  |  |  |
| **Pooled summary result (overall rating)** | |  |  |  | **+/-** | **617** | **1VG, 1A** | **+** | **391** | **1A, 1D** | **+** |
|  | |  |  |  | **NG** |  |  | **High** |  |  | **Moderate** |

Rel: relevance; CHV: comprehensiveness, CHB: comprehensibility; CV: content validity; N: number (sample); MQ: methodological quality; R: rating; VG: very good; A: adequate; D: doubtful; NG: not graded; +: sufficient; ?: indeterminant

| **NMF2S** | | | | | | | | | | | |
| --- | --- | --- | --- | --- | --- | --- | --- | --- | --- | --- | --- |
| **Reference** | **Country (language)** | **Content validity** | | | | **Structural validity** | | | **Responsiveness  (A – D)** | | |
|  |  | **Rel** | **CHV** | **CHB** | **CV** | **N** | **MQ** | **R** | **N** | **MQ** | **R** |
| Faggianelli et al., 2024 | France (French) | + | + | + | + | 310 | VG | + | 310 | VG | + |
| **Pooled summary result (overall rating)** | | **+** | **+** | **+** | **+** | **310** | **1VG** | **+** | **310** | **1VG** | **+** |
|  | |  |  |  | **Very low** |  |  | **High** |  |  | **High** |

Rel: relevance; CHV: comprehensiveness, CHB: comprehensibility; CV: content validity; N: number (sample); MQ: methodological quality; R: rating; VG: very good; +: sufficient

| **POSQ** | | | | |
| --- | --- | --- | --- | --- |
| **Reference** | **Country (language)** | **Reliability** | | |
|  |  | **N** | **MQ** | **R** |
| Matza et al., 2005 | United States of America (English) | 46 | A | + |
| **Pooled summary result (overall rating)** | | **46** | **1A** | **+** |
| **Grade** | |  |  | **Very low** |

N: number (sample); MQ: methodological quality; R: rating; A: adequate; +: sufficient

| **PSYCHLOPS** | | | | | | | | | | | | | | | | | | | | |
| --- | --- | --- | --- | --- | --- | --- | --- | --- | --- | --- | --- | --- | --- | --- | --- | --- | --- | --- | --- | --- |
| **Reference** | **Country (language)** | **Content validity** | | | | **Structural validity** | | | **Internal consistency** | | | **Reliability** | | | **Hypothesis testing  (A & B)** | | | **Responsiveness  (A – D)** | | |
|  |  | **Rel** | **CHV** | **CHB** | **CV** | **N** | **MQ** | **R** | **N** | **MQ** | **R** | **N** | **MQ** | **R** | **N** | **MQ** | **R** | **N** | **MQ** | **R** |
| Sales et al., 2021 | Portuguese (Portugal) |  |  |  |  | 939 | VG | + | 939 | VG | + |  |  |  | 939 | A | + | 939 | A, D | + |
| Ashworth, 2004 | United Kingdom (English) | + | ? | - | +/- |  |  |  |  |  |  |  |  |  |  |  |  |  |  |  |
| Evans et al., 2010 | United Kingdom (English) |  |  |  |  |  |  |  | 70 | VG | + | 56 | A | + |  |  |  |  |  |  |
| Austin et al., 2021 | United Kingdom (English) |  |  |  |  |  |  |  | 278 | VG | + |  |  |  |  |  |  | 278 | A, A | ? |
| Ashworth et al., 2009 | United Kingdom (English) |  |  |  |  |  |  |  | 114 | VG | + |  |  |  | 114 | VG | ? | 114 | VG, VG | ? |
| Ashworth, Robinson, Godfrey, Shepherd et al., 2005 | United Kingdom (English) |  |  |  |  |  |  |  | 110 | VG | + |  |  |  | 110 | VG | ? | 110 | VG, VG | + |
| Alves et al., 2020 | Portugal (Portuguese) |  |  |  |  |  |  |  | 58 | VG | +/- |  |  |  | 58 | A, D | ? |  |  |  |
| Heoinsson et al., 2013 | Iceland (Icelandic) |  |  |  |  |  |  |  | 100 | VG | + |  |  |  | 100 | VG | + | 100 | VG | + |
| Czachwoski et al., 2011 | Poland (Polish) |  |  |  |  |  |  |  | 135 or 238 | VG | + |  |  |  | 135 | I | ? | 135 | VG | + |
| Ashworth, Robinson, Godfrey, Parmentier et al., 2005 | United Kingdom (English) | + |  |  |  |  |  |  |  |  |  |  |  |  |  |  |  |  |  |  |
| **Pooled summary result (overall rating)** | | **+** | **+** | **+** | **+** | **939** | **1VG** | **+** | **1287*** | **8VG** | **+** | **56** | **1A** | **+** | **939** | **3VG, 2A, 1D, 1I** | **+** | **1217*** | **6VG, 3A, 1D** | **+** |
| **Grade** | |  |  |  | **Very low** |  |  | **High** |  |  | **High** |  |  | **Low** |  |  | **High** |  |  | **High** |

Rel: relevance; CHV: comprehensiveness, CHB: comprehensibility; CV: content validity; N: number (sample); MQ: methodological quality; R: rating; VG: very good; A: adequate; D: doubtful; I: inadequate; NG: not graded; +: sufficient; -: insufficient; ?: indeterminant
* Pooled sample size excludes individual sample sizes from Ashworth et al., 2009, Ashworth, Robinson, Godfrey, Shepherd et al., 2005, Alves et al., 2020, Heoinsson et al., 2013, Czachowski et al., 2011, and Ashworth, Robinson, Godfrey, Parmentier et al., 2005 due to being included in Sales et al., 2021.

| **Questionnaire Female LUTS** | | | | | | | | |
| --- | --- | --- | --- | --- | --- | --- | --- | --- |
| **Reference** | **Country (language)** | **Content validity** | | | | **Reliability** | | |
|  |  | **Rel** | **CHV** | **CHB** | **CV** | **N** | **MQ** | **R** |
| Bernstein et al., 1996 | Denmark (English) | + | + | + | + | 56 | I | ? |
| **Pooled summary result (overall rating)** | | **+** | **+** | **+** | **+** | **56** | **1I** | **?** |
| **Grade** | |  |  |  | **Very low** |  |  | **NG** |

Rel: relevance; CHV: comprehensiveness, CHB: comprehensibility; CV: content validity; N: number (sample); MQ: methodological quality; R: rating; I: inadequate; NG: not graded; +: sufficient; ?: indeterminant

| **QUAL-E** | | | | | | | | | | | | | | | | | |
| --- | --- | --- | --- | --- | --- | --- | --- | --- | --- | --- | --- | --- | --- | --- | --- | --- | --- |
| **Reference** | **Country (language)** | **Content validity** | | | | **Structural validity** | | | **Internal consistency** | | | **Reliability** | | | **Hypothesis testing  (A & B)** | | |
|  |  | **Rel** | **CHV** | **CHB** | **CV** | **N** | **MQ** | **R** | **N** | **MQ** | **R** | **N** | **MQ** | **R** | **N** | **MQ** | **R** |
| Steinhauser et al., 2002 | United States of America (English) | + | + | + | + | 200 | A | ? (EFA) | 200 | VG | ? |  |  |  |  |  |  |
| Steinhauser et al., 2004 | United States of America (English) |  |  |  |  | 248 | VG | ? (EFA) | 248 | VG | ? | 135 – 170 | D | no changes group: completion 0.73 (+), healthcare 0.55 (-), symptoms 0.18 (-), preparation 0.72 (+) | 248 | D | completion (+), healthcare (+), symptoms (+), preparation (+) |
|  |  |  |  |  |  |  |  |  |  |  |  |  |  |  | 248 | A | completion (+), healthcare (+), symptoms (+), preparation (+) |
| Wilkinson et al., 2014 | Australia (English) |  | ? |  |  |  |  |  |  |  |  |  |  |  |  |  |  |
| **Pooled summary result (overall rating)** | | **+** | **+** | **+** | **+** | **448** | **1VG, 1A** | **?** | **448** | **2VG** | **?** | **135 – 170** | **1D** | **+/-** | **248** | **1A, 1D** | **+** |
| **Grade** | |  |  |  | **Low** |  |  | **NG** |  |  | **NG** |  |  | **Low** |  |  | **Moderate** |

Rel: relevance; CHV: comprehensiveness, CHB: comprehensibility; CV: content validity; N: number (sample); MQ: methodological quality; R: rating; EFA: exploratory factor analysis; VG: very good; A: adequate; D: doubtful; NG: not graded; +: sufficient; -: insufficient; ?: indeterminant

| **QUAL-EC** | | | | | | | | | | | | | | |
| --- | --- | --- | --- | --- | --- | --- | --- | --- | --- | --- | --- | --- | --- | --- |
| **Reference** | **Country (language)** | **Content validity** | | | | **Structural validity** | | | **Internal consistency** | | | **Hypothesis testing  (A & B)** | | |
|  |  | **Rel** | **CHV** | **CHB** | **CV** | **N** | **MQ** | **R** | **N** | **MQ** | **R** | **N** | **MQ** | **R** |
| Lo et al., 2011 | Canada (English) | + | - | + | +/- | 468 | VG | - | 468 | VG | ? | 468 | A | + |
| **Pooled summary result (overall rating)** | | **+** | **+** | **+** | **+** | **468** | **1VG** | **-** | **468** | **1VG** | **?** | **468** | **1A** | **+** |
| **Grade** | |  |  |  | **Moderate** |  |  | **High** |  |  | **NG *** |  |  | **Moderate** |

* Structural validity insufficient therefore internal consistency unable to be graded

Rel: relevance; CHV: comprehensiveness, CHB: comprehensibility; CV: content validity; N: number (sample); MQ: methodological quality; R: rating; VG: very good; A: adequate; NG: not graded; +: sufficient; -: insufficient; ?: indeterminant

| **SF-LDQ** | | | | | | | | | | | | | | | | | | | | |
| --- | --- | --- | --- | --- | --- | --- | --- | --- | --- | --- | --- | --- | --- | --- | --- | --- | --- | --- | --- | --- |
| **Reference** | **Country (language)** | **Content validity** | | | | **Internal consistency** | | | **Reliability** | | | **Criterion validity** | | | **Hypothesis testing  (A & B)** | | | **Responsiveness  (A – D)** | | |
|  |  | **Rel** | **CHV** | **CHB** | **CV** | **N** | **MQ** | **R** | **N** | **MQ** | **R** | **N** | **MQ** | **R** | **N** | **M** | **R** | **N** | **MQ** | **R** |
| Fraser et al., 2007 | United Kingdom (English) | + | + | + | + | 592 | D | ? | 151 | D | ? | 375 | VG | + | 375 | VG | + | 37 | D | + |
| Allabdulkareem et al., 2021 | Saudi Arabia (Arabic) |  |  |  |  |  |  |  |  |  |  | 245 | VG | + | 244 | VG | + |  |  |  |
| Gatta et al., 2010 | Italy (Italian) |  |  |  |  | 469 | D | ? | 162-182 | D | + | 469 | VG | + | 365 | VG | + | 35 | VG | + |
| Nkurunziza et al., 2016 | Rwanda (Kinyarwanda) |  |  |  |  | 200 | D | ? | 200 | I | ? | 200 | VG | ? |  |  |  | 20 | VG | + |
| **Pooled summary result (overall rating)** | | **+** | **+** | **+** | **+** | **1261** | **3D** | **?** | **513-533** | **2D, 1I** | **+** | **1289** | **4VG** | **+** | **1201** | **3VG** | **+** | **92** | **2VG, 1D** | **+** |
| **Grade** | |  |  |  | **Very low** |  |  | **NG** |  |  | **Low** |  |  | **High** |  |  | **High** |  |  | **Moderate** |

Rel: relevance; CHV: comprehensiveness, CHB: comprehensibility; CV: content validity; N: number (sample); MQ: methodological quality; R: rating; VG: very good; D: doubtful; I: inadequate; NG: not graded; +: sufficient; ?: indeterminant

| **UDI** | | | | | | | | | | | | | | | | | | | | | | | |
| --- | --- | --- | --- | --- | --- | --- | --- | --- | --- | --- | --- | --- | --- | --- | --- | --- | --- | --- | --- | --- | --- | --- | --- |
| **Reference** | **Country (language)** | **Content validity** | | | | **Structural validity** | | | **Internal consistency** | | | **Measurement invariance** | | | **Criterion validity** | | | **Hypothesis testing  (A & B)** | | | **Responsiveness  (A – D)** | | |
|  |  | **Rel** | **CHV** | **CHB** | **CV** | **N** | **MQ** | **R** | **N** | **MQ** | **R** | **N** | **MQ** | **R** | **N** | **MQ** | **R** | **N** | **MQ** | **R** | **N** | **MQ** | **R** |
| Shumaker et al., 1994 | United States of America (English) | + | + | + | + | 162 | A | ? | 162 | VG | ? |  |  |  | 162 | VG | + |  |  |  | 131 | VG | + |
| Harvey et al., 2001 | United States of America (English) |  |  |  |  |  |  |  | 150 | I | ? |  |  |  | 150 | VG | - |  |  |  | 150 | I | + |
|  |  |  |  |  |  |  |  |  |  |  |  |  |  |  |  |  |  |  |  |  | 150 | VG | + |
| Hagen et al., 2002 | United Kingdom (English) |  |  |  |  |  |  |  |  |  |  |  |  |  |  |  |  |  |  |  | 237 | A | + |
|  |  |  |  |  |  |  |  |  |  |  |  |  |  |  |  |  |  |  |  |  | 237 | A | + |
| Van der Vaart, 2003 | Netherlands (Dutch) |  |  |  |  | 2043 | A | ? | 2043 | VG | ? |  |  |  |  |  |  | 960 | A | +/- |  |  |  |
|  |  |  |  |  |  |  |  |  |  |  |  |  |  |  |  |  |  | 196 | A | + |  |  |  |
| Artibani, 2006 | Italy (Italian) |  |  | + |  | 106 | A | ? | 106 | VG | ? |  |  |  |  |  |  | 52 | I | ? | 41 | D | + |
|  |  |  |  |  |  |  |  |  |  |  |  |  |  |  |  |  |  | 52 | VG | + |  |  |  |
| **Pooled summary result (overall rating)** | | **+** | **+** | **+** | **+** | **2311** | **3A** | **?** | **2461** | **3VG, 1I** | **?** |  |  |  | **312** | **2VG** | **+/-** | **1012*** | **1VG, 2A, 1I** | **+** | **559*** | **2VG, 2A, 1D, 1I** | **+** |
| **Grade** | |  |  |  | **Very low** |  |  | **NG** |  |  | **NG** |  |  |  |  |  | **Moderate** |  |  | **High** |  |  | **High** |

Rel: relevance; CHV: comprehensiveness, CHB: comprehensibility; CV: content validity; N: number (sample); MQ: methodological quality; R: rating; VG: very good; A: adequate; D: doubtful; I: inadequate; NG: not graded; +: sufficient; -: insufficient; ?: indeterminant
* Subsamples or duplicates of total study samples were not included in pooled total number of participants for hypothesis testing and responsiveness

| **U-UDI** | | | | | | | | | | | | | | | | | | | | |
| --- | --- | --- | --- | --- | --- | --- | --- | --- | --- | --- | --- | --- | --- | --- | --- | --- | --- | --- | --- | --- |
| **Reference** | **Country (language)** | **Content validity** | | | | **Structural validity** | | | **Internal consistency** | | | **Reliability** | | | **Hypothesis testing  (A & B)** | | | **Responsiveness  (A – D)** | | |
|  |  | **Rel** | **CHV** | **CHB** | **CV** | **N** | **MQ** | **R** | **N** | **MQ** | **R** | **N** | **MQ** | **R** | **N** | **MQ** | **R** | **N** | **MQ** | **R** |
| Brown, 1999 | United States of America (English) | + | + | + | + | 82 | A | ? | 82 | VG | ? | 40 | A | - | 83 | A | + |  |  |  |
| Borello France et al., 2008 | United States of America (English) |  |  |  |  |  |  |  |  |  |  | 49 | A | +/- |  |  |  |  |  |  |
| Lubeck et al., 1999 | United States of America (English) |  |  |  |  |  |  |  | 218 | VG | ? | 218 | A | + | 129 | A | + | 206 | VG | + |
| **Pooled summary result (overall rating)** | | **+** | **+** | **+** | **+** | **82** | **1A** | **?** | **300** | **2VG** | **?** | **307** | **3A** | **+** | **212** | **2A** | **+** | **206** | **1VG** | **+** |
| **Grade** | |  |  |  | **Very low** |  |  | **NG** |  |  | **NG** |  |  | **Moderate** |  |  | **High** |  |  | **High** |

Rel: relevance; CHV: comprehensiveness, CHB: comprehensibility; CV: content validity; N: number (sample); MQ: methodological quality; R: rating; VG: very good; A: adequate; NG: not graded; +: sufficient; ?: indeterminant
