# Supplementary material for: How is the “most bothersome symptom” construct measured across health conditions? A systematic review of patient-reported outcome measures
Source: Patient Relat Outcome Meas. 2026 Sep 20;17(1):107–22. doi: 10.1080/1179271X.2026.2732841 (PMC13596887; doi:10.1080/1179271X.2026.2732841)
Supplement: Supplementary file 2 [file DPRO_A_2732841_SM9471.docx]

**Appendix 5: Content validity ratings**

| **GSM-SVTAQ (English and Arabic)** | | **PROM development study**  **(Hassanein et al., 2024)** | **Content validity study 1**  **(Hassanein et al., 2024)** | **Rating of reviewers** | **OVERALL RATINGS PER PROM^3^** | **QUALITY OF EVIDENCE** |
| --- | --- | --- | --- | --- | --- | --- |
|  | | **+ / - / ?** | **+ / - / ?** | **+ / - / ?** | **+ / - / ± / ?** | **High, moderate, low, very low** |
| **Relevance** | |  |  |  |  |  |
| 1 | Are the included items relevant for the construct of interest?^1^ | + | + | + |  |  |
| 2 | Are the included items relevant for the target population of interest?^1^ | + | + | + |  |  |
| 3 | Are the included items relevant for the context of use of interest?^1^ | + | + | + |  |  |
| 4 | Are the response options appropriate? | ? | + | + |  |  |
| 5 | Is the recall period appropriate? | + | + | + |  |  |
|  | **RELEVANCE RATING (+ / - / ± / ?)** | **+** | **+** | **+** | **+** | **High** |
|  |  |  |  |  |  |  |
| **Comprehensiveness** | |  |  |  |  |  |
| 6 | Are all key concepts included? | + | ? | + |  |  |
|  | **COMPREHENSIVENESS RATING (+ / - / ± / ?)** | **+** | **?** | **+** | **+** | **Moderate** |
|  |  |  |  |  |  |  |
| **Comprehensibility** | |  |  |  |  |  |
| 7 | Are the PROM instructions understood by the population of interest as intended? | + | + |  |  |  |
| 8 | Are the PROM items and response options understood by the population of interest as intended? | + | + |  |  |  |
| 9 | Are the PROM items appropriately worded? |  |  | + |  |  |
| 10 | Do the response options match the question? |  |  | + |  |  |
|  | **COMPREHENSIBILITY RATING (+ / - / ± / ?)** | **+** | **+** | **+** | **+** | **High** |
|  |  |  |  |  |  |  |
|  | **CONTENT VALIDITY RATING (+ / - / ± / ?)** | **+** | **+** | **+** | **+** | **High** |

+: sufficient rating; -: insufficient rating; ±: inconsistent rating; ?: indeterminate rating

| **MQOL (English and English/French)** | | **PROM development study**  **(Cohen et al., 1995)** | **Content validity study 1**  **(Cohen et al., 1996)** | **Content validity study 2**  **(Cohen, Hassan et al.,1996)** | **Content validity study 3 (Pratheepawanit et al., 1999)** | **Content validity study 4**  **(Gofton et al., 2015)** | **Rating of reviewers** | **OVERALL RATINGS PER PROM^3^** | **QUALITY OF EVIDENCE** |
| --- | --- | --- | --- | --- | --- | --- | --- | --- | --- |
|  | | **+ / - / ?** | **+ / - / ?** | **+ / - / ?** | **+ / - / ?** | **+ / - / ?** | **+ / - / ?** | **+ / - / ± / ?** | **High, moderate, low, very low** |
| **Relevance** | |  |  |  |  |  |  |  |  |
| 1 | Are the included items relevant for the construct of interest?^1^ | + |  | ? |  |  | + |  |  |
| 2 | Are the included items relevant for the target population of interest?^1^ | + |  | ? |  |  | + |  |  |
| 3 | Are the included items relevant for the context of use of interest?^1^ | + |  | ? |  |  | + |  |  |
| 4 | Are the response options appropriate? | ? |  | ? |  |  | + |  |  |
| 5 | Is the recall period appropriate? | ? |  | ? |  |  | + |  |  |
|  | **RELEVANCE RATING (+ / - / ± / ?)** | **?** |  | **?** |  |  | **+** | **+** | **Very low** |
|  |  |  |  |  |  |  |  |  |  |
| **Comprehensiveness** | |  |  |  |  |  |  |  |  |
| 6 | Are all key concepts included? | ? | + |  | + | - | + |  |  |
|  | **COMPREHENSIVENESS RATING (+ / - / ± / ?)** | **?** | **+** |  | **+** | **-** | **+** | **+** | **Moderate** |
|  |  |  |  |  |  |  |  |  |  |
| **Comprehensibility** | |  |  |  |  |  |  |  |  |
| 7 | Are the PROM instructions understood by the population of interest as intended? | ? | + | ? | - | ? |  |  |  |
| 8 | Are the PROM items and response options understood by the population of interest as intended? | ? | + | ? | + | + |  |  |  |
| 9 | Are the PROM items appropriately worded? |  |  |  |  |  | + |  |  |
| 10 | Do the response options match the question? |  |  |  |  |  | + |  |  |
|  | **COMPREHENSIBILITY RATING (+ / - / ± / ?)** | **?** | **+** | **?** | **+/-** | **+** | **+** | **+** | **Moderate** |
|  |  |  |  |  |  |  |  |  |  |
|  | **CONTENT VALIDITY RATING (+ / - / ± / ?)** | **?** | **+** | **?** | **+** | **+/-** | **+** | **+** | **Moderate** |

+: sufficient rating; -: insufficient rating; ±: inconsistent rating; ?: indeterminate rating

| **MQOL (Italian)** | | **PROM development study**  **(Cohen et al., 1995)** | **Content validity study 1**  **(Sguazzin et al., 2010)** | **Rating of reviewers** | **OVERALL RATINGS PER PROM^3^** | **QUALITY OF EVIDENCE** |
| --- | --- | --- | --- | --- | --- | --- |
|  | | **+ / - / ?** | **+ / - / ?** | **+ / - / ?** | **+ / - / ± / ?** | **High, moderate, low, very low** |
| **Relevance** | |  |  |  |  |  |
| 1 | Are the included items relevant for the construct of interest?^1^ | + |  | + |  |  |
| 2 | Are the included items relevant for the target population of interest?^1^ | + |  | + |  |  |
| 3 | Are the included items relevant for the context of use of interest?^1^ | + |  | + |  |  |
| 4 | Are the response options appropriate? | ? |  | + |  |  |
| 5 | Is the recall period appropriate? | ? |  | + |  |  |
|  | **RELEVANCE RATING (+ / - / ± / ?)** | **?** |  | **+** | **+** | **Very low** |
|  |  |  |  |  |  |  |
| **Comprehensiveness** | |  |  |  |  |  |
| 6 | Are all key concepts included? | ? |  | + |  |  |
|  | **COMPREHENSIVENESS RATING (+ / - / ± / ?)** | **?** |  | **+** | **+** | **Very low** |
|  |  |  |  |  |  |  |
| **Comprehensibility** | |  |  |  |  |  |
| 7 | Are the PROM instructions understood by the population of interest as intended? | ? | ? |  |  |  |
| 8 | Are the PROM items and response options understood by the population of interest as intended? | ? | ? |  |  |  |
| 9 | Are the PROM items appropriately worded? |  |  | + |  |  |
| 10 | Do the response options match the question? |  |  | + |  |  |
|  | **COMPREHENSIBILITY RATING (+ / - / ± / ?)** | **?** | **?** | **+** | **+** | **Very low** |
|  |  |  |  |  |  |  |
|  | **CONTENT VALIDITY RATING (+ / - / ± / ?)** | **?** | **?** | **+** | **+** | **Very low** |

+: sufficient rating; -: insufficient rating; ±: inconsistent rating; ?: indeterminate rating

| **MQOL (Korean)** | | **PROM development study**  **(Cohen et al., 1995)** | **Content validity study**  **(Kim et al., 2007)** | **Rating of reviewers** | **OVERALL RATINGS PER PROM^3^** | **QUALITY OF EVIDENCE** |
| --- | --- | --- | --- | --- | --- | --- |
|  | | **+ / - / ?** | **+ / - / ?** | **+ / - / ?** | **+ / - / ± / ?** | **High, moderate, low, very low** |
| **Relevance** | |  |  |  |  |  |
| 1 | Are the included items relevant for the construct of interest?^1^ | + |  | + |  |  |
| 2 | Are the included items relevant for the target population of interest?^1^ | + |  | + |  |  |
| 3 | Are the included items relevant for the context of use of interest?^1^ | + |  | + |  |  |
| 4 | Are the response options appropriate? | ? |  | + |  |  |
| 5 | Is the recall period appropriate? | ? |  | + |  |  |
|  | **RELEVANCE RATING (+ / - / ± / ?)** | **?** |  | **+** | **+** | **Very low** |
|  |  |  |  |  |  |  |
| **Comprehensiveness** | |  |  |  |  |  |
| 6 | Are all key concepts included? | ? |  | + |  |  |
|  | **COMPREHENSIVENESS RATING (+ / - / ± / ?)** | **?** |  | **+** | **+** | **Very low** |
|  |  |  |  |  |  |  |
| **Comprehensibility** | |  |  |  |  |  |
| 7 | Are the PROM instructions understood by the population of interest as intended? | ? | ? |  |  |  |
| 8 | Are the PROM items and response options understood by the population of interest as intended? | ? | + |  |  |  |
| 9 | Are the PROM items appropriately worded? |  |  | + |  |  |
| 10 | Do the response options match the question? |  |  | + |  |  |
|  | **COMPREHENSIBILITY RATING (+ / - / ± / ?)** | **?** | **+** | **+** | **+** | **Moderate** |
|  |  |  |  |  |  |  |
|  | **CONTENT VALIDITY RATING (+ / - / ± / ?)** | **?** | **+** | **+** | **+** | **Very low** |

+: sufficient rating; -: insufficient rating; ±: inconsistent rating; ?: indeterminate rating

| **MQOL (Dutch)** | | **PROM development study**  **(Cohen et al., 1995)** | **Content validity study 1**  **(DeVrieze et al., 2016)** | **Rating of reviewers** | **OVERALL RATINGS PER PROM^3^** | **QUALITY OF EVIDENCE** |
| --- | --- | --- | --- | --- | --- | --- |
|  | | **+ / - / ?** | **+ / - / ?** | **+ / - / ?** | **+ / - / ± / ?** | **High, moderate, low, very low** |
| **Relevance** | |  |  |  |  |  |
| 1 | Are the included items relevant for the construct of interest?^1^ | + | ? | + |  |  |
| 2 | Are the included items relevant for the target population of interest?^1^ | + | + | + |  |  |
| 3 | Are the included items relevant for the context of use of interest?^1^ | + | ? | + |  |  |
| 4 | Are the response options appropriate? | ? | ? | + |  |  |
| 5 | Is the recall period appropriate? | ? | ? | + |  |  |
|  | **RELEVANCE RATING (+ / - / ± / ?)** | **?** | **?** | **+** | **+** | **Very low** |
|  |  |  |  |  |  |  |
| **Comprehensiveness** | |  |  |  |  |  |
| 6 | Are all key concepts included? | ? |  | + |  |  |
|  | **COMPREHENSIVENESS RATING (+ / - / ± / ?)** | **?** |  | **+** | **+** | **Very low** |
|  |  |  |  |  |  |  |
| **Comprehensibility** | |  |  |  |  |  |
| 7 | Are the PROM instructions understood by the population of interest as intended? | ? | ? |  |  |  |
| 8 | Are the PROM items and response options understood by the population of interest as intended? | ? | + |  |  |  |
| 9 | Are the PROM items appropriately worded? |  |  | + |  |  |
| 10 | Do the response options match the question? |  |  | + |  |  |
|  | **COMPREHENSIBILITY RATING (+ / - / ± / ?)** | **?** | **+** | **+** | **+** | **Moderate** |
|  |  |  |  |  |  |  |
|  | **CONTENT VALIDITY RATING (+ / - / ± / ?)** | **?** | **+** | **+** | **+** | **Very low** |

+: sufficient rating; -: insufficient rating; ±: inconsistent rating; ?: indeterminate rating

| **MQOL (Chinese)** | | **PROM development study (Cohen et al., 1995)** | **Content validity study 1 (Zhang et al., 2003)** | **Content validity study 2**  **(Hu et al., 2003)** | **Rating of reviewers** | **OVERALL RATINGS PER PROM^3^** | **QUALITY OF EVIDENCE** |
| --- | --- | --- | --- | --- | --- | --- | --- |
|  | | **+ / - / ?** | **+ / - / ?** | **+ / - / ?** | **+ / - / ?** | **+ / - / ± / ?** | **High, moderate, low, very low** |
| **Relevance** | |  |  |  |  |  |  |
| 1 | Are the included items relevant for the construct of interest?^1^ | + | ? | ? | + |  |  |
| 2 | Are the included items relevant for the target population of interest?^1^ | + | ? | + | + |  |  |
| 3 | Are the included items relevant for the context of use of interest?^1^ | + | ? | ? | + |  |  |
| 4 | Are the response options appropriate? | ? | ? | ? | + |  |  |
| 5 | Is the recall period appropriate? | ? | ? | ? | + |  |  |
|  | **RELEVANCE RATING (+ / - / ± / ?)** | **?** | ? | **?** | **+** | **+** | **Very low** |
|  |  |  |  |  |  |  |  |
| **Comprehensiveness** | |  |  |  |  |  |  |
| 6 | Are all key concepts included? | ? |  |  | + |  |  |
|  | **COMPREHENSIVENESS RATING (+ / - / ± / ?)** | **?** |  |  | **+** | **+** | **Very low** |
|  |  |  |  |  |  |  |  |
| **Comprehensibility** | |  |  |  |  |  |  |
| 7 | Are the PROM instructions understood by the population of interest as intended? | ? | ? |  |  |  |  |
| 8 | Are the PROM items and response options understood by the population of interest as intended? | ? | ? |  |  |  |  |
| 9 | Are the PROM items appropriately worded? |  |  |  | + |  |  |
| 10 | Do the response options match the question? |  |  |  | + |  |  |
|  | **COMPREHENSIBILITY RATING (+ / - / ± / ?)** | **?** | ? |  | **+** | **+** | **Very low** |
|  |  |  |  |  |  |  |  |
|  | **CONTENT VALIDITY RATING (+ / - / ± / ?)** | **?** | ? | **?** | **+** | **+** | **Very low** |

+: sufficient rating; -: insufficient rating; ±: inconsistent rating; ?: indeterminate rating

| **MUDI** | | **PROM development study**  **(Robinson et al., 2002)** | **Rating of reviewers** | **OVERALL RATINGS PER PROM^3^** | **QUALITY OF EVIDENCE** |
| --- | --- | --- | --- | --- | --- |
|  | | **+ / - / ?** | **+ / - / ?** | **+ / - / ± / ?** | **High, moderate, low, very low** |
| **Relevance** | |  |  |  |  |
| 1 | Are the included items relevant for the construct of interest?^1^ | + | + |  |  |
| 2 | Are the included items relevant for the target population of interest?^1^ | + | + |  |  |
| 3 | Are the included items relevant for the context of use of interest?^1^ | + | + |  |  |
| 4 | Are the response options appropriate? | ? | + |  |  |
| 5 | Is the recall period appropriate? | ? | + |  |  |
|  | **RELEVANCE RATING (+ / - / ± / ?)** | **?** | + | **+** | **Low** |
|  |  |  |  |  |  |
| **Comprehensiveness** | |  |  |  |  |
| 6 | Are all key concepts included? | - | + |  |  |
|  | **COMPREHENSIVENESS RATING (+ / - / ± / ?)** | **-** | **+** | **+** | **Low** |
|  |  |  |  |  |  |
| **Comprehensibility** | |  |  |  |  |
| 7 | Are the PROM instructions understood by the population of interest as intended? | ? |  |  |  |
| 8 | Are the PROM items and response options understood by the population of interest as intended? | + |  |  |  |
| 9 | Are the PROM items appropriately worded? |  | + |  |  |
| 10 | Do the response options match the question? |  | + |  |  |
|  | **COMPREHENSIBILITY RATING (+ / - / ± / ?)** | **_+** | **+** | **+** | **Low** |
|  |  |  |  |  |  |
|  | **CONTENT VALIDITY RATING (+ / - / ± / ?)** | **+/-** | **+** | **+** | **Low** |

+: sufficient rating; -: insufficient rating; ±: inconsistent rating; ?: indeterminate rating

| **MYMOP1** | | **PROM development study**  **(Patterson et al., 2000)** | **Content validity study 1**  **(Murphy et al., 2018)** | **Content validity study 2 (Paterson & Britton, 2000)** | **Content validity study 3 (Chung et al., 2010)** | **Rating of reviewers** | **OVERALL RATINGS PER PROM^3^** | **QUALITY OF EVIDENCE** |
| --- | --- | --- | --- | --- | --- | --- | --- | --- |
|  | | **+ / - / ?** | **+ / - / ?** | **+ / - / ?** | **+ / - / ?** | **+ / - / ?** | **+ / - / ± / ?** | **High, moderate, low, very low** |
| **Relevance** | |  |  |  |  |  |  |  |
| 1 | Are the included items relevant for the construct of interest?^1^ |  |  | ? |  | + |  |  |
| 2 | Are the included items relevant for the target population of interest?^1^ |  |  | + |  | + |  |  |
| 3 | Are the included items relevant for the context of use of interest?^1^ |  |  | ? |  | + |  |  |
| 4 | Are the response options appropriate? |  |  | ? |  | + |  |  |
| 5 | Is the recall period appropriate? |  |  | ? |  | + |  |  |
|  | **RELEVANCE RATING (+ / - / ± / ?)** |  |  | **?** |  | + | **+** | **Very Low** |
|  |  |  |  |  |  |  |  |  |
| **Comprehensiveness** | |  |  |  |  |  |  |  |
| 6 | Are all key concepts included? |  |  | - |  | - |  |  |
|  | **COMPREHENSIVENESS RATING (+ / - / ± / ?)** |  |  | **-** |  | **-** | **-** | **High** |
|  |  |  |  |  |  |  |  |  |
| **Comprehensibility** | |  |  |  |  |  |  |  |
| 7 | Are the PROM instructions understood by the population of interest as intended? |  | + | ? | + |  |  |  |
| 8 | Are the PROM items and response options understood by the population of interest as intended? |  | + | ? | + |  |  |  |
| 9 | Are the PROM items appropriately worded? |  |  |  |  | + |  |  |
| 10 | Do the response options match the question? |  |  |  |  | + |  |  |
|  | **COMPREHENSIBILITY RATING (+ / - / ± / ?)** |  | **+** | **?** | + | **+** | **+** | **High** |
|  |  |  |  |  |  |  |  |  |
|  | **CONTENT VALIDITY RATING (+ / - / ± / ?)** |  |  | **?** |  | **+/-** | **+/-** | **Not graded** |

+: sufficient rating; -: insufficient rating; ±: inconsistent rating; ?: indeterminate rating

Development study unable to be rated due to inadequate information provided (Paterson et al., 2000)

| **MYMOP2** | | **PROM development study**  **(Patterson et al., 2000)** | **Content validity study 1**  **(Paterson et al. 2004)** | **Rating of reviewers** | **OVERALL RATINGS PER PROM^3^** | **QUALITY OF EVIDENCE** |
| --- | --- | --- | --- | --- | --- | --- |
|  | | **+ / - / ?** | **+ / - / ?** | **+ / - / ?** | **+ / - / ± / ?** | **High, moderate, low, very low** |
| **Relevance** | |  |  |  |  |  |
| 1 | Are the included items relevant for the construct of interest?^1^ |  |  | + |  |  |
| 2 | Are the included items relevant for the target population of interest?^1^ |  |  | + |  |  |
| 3 | Are the included items relevant for the context of use of interest?^1^ |  |  | + |  |  |
| 4 | Are the response options appropriate? |  |  | + |  |  |
| 5 | Is the recall period appropriate? |  |  | + |  |  |
|  | **RELEVANCE RATING (+ / - / ± / ?)** |  |  | + | **+** | **Very low** |
|  |  |  |  |  |  |  |
| **Comprehensiveness** | |  |  |  |  |  |
| 6 | Are all key concepts included? |  | - | - |  |  |
|  | **COMPREHENSIVENESS RATING (+ / - / ± / ?)** |  | **-** | **-** | **-** | **Very low** |
|  |  |  |  |  |  |  |
| **Comprehensibility** | |  |  |  |  |  |
| 7 | Are the PROM instructions understood by the population of interest as intended? |  |  |  |  |  |
| 8 | Are the PROM items and response options understood by the population of interest as intended? |  |  |  |  |  |
| 9 | Are the PROM items appropriately worded? |  |  | + |  |  |
| 10 | Do the response options match the question? |  |  | + |  |  |
|  | **COMPREHENSIBILITY RATING (+ / - / ± / ?)** |  |  | **+** | **+** | **Very low** |
|  |  |  |  |  |  |  |
|  | **CONTENT VALIDITY RATING (+ / - / ± / ?)** |  |  | **+/-** | **+/-** | **Not graded** |

+: sufficient rating; -: insufficient rating; ±: inconsistent rating; ?: indeterminate rating

Development study unable to be rated due to inadequate information provided (Paterson et al., 2000)

| **NMF2S (French)** | | **Rating of reviewers*** | **OVERALL RATINGS PER PROM^3^** | **QUALITY OF EVIDENCE** |
| --- | --- | --- | --- | --- |
|  | | **+ / - / ?** | **+ / - / ± / ?** | **High, moderate, low, very low** |
| **Relevance** | |  |  |  |
| 1 | Are the included items relevant for the construct of interest?^1^ | + |  |  |
| 2 | Are the included items relevant for the target population of interest?^1^ | + |  |  |
| 3 | Are the included items relevant for the context of use of interest?^1^ | + |  |  |
| 4 | Are the response options appropriate? | + |  |  |
| 5 | Is the recall period appropriate? | + |  |  |
|  | **RELEVANCE RATING (+ / - / ± / ?)** | **+** | **+** | **Very low** |
|  |  |  |  |  |
| **Comprehensiveness** | |  |  |  |
| 6 | Are all key concepts included? | + |  |  |
|  | **COMPREHENSIVENESS RATING (+ / - / ± / ?)** | **+** | **+** | **+** |
|  |  |  |  |  |
| **Comprehensibility** | |  |  |  |
| 7 | Are the PROM instructions understood by the population of interest as intended? |  |  |  |
| 8 | Are the PROM items and response options understood by the population of interest as intended? |  |  |  |
| 9 | Are the PROM items appropriately worded? | + |  |  |
| 10 | Do the response options match the question? | + |  |  |
|  | **COMPREHENSIBILITY RATING (+ / - / ± / ?)** | **+** | **+** | **+** |
|  |  |  |  |  |
|  | **CONTENT VALIDITY RATING (+ / - / ± / ?)** | **+** | **+** | **+** |

+: sufficient rating; -: insufficient rating; ±: inconsistent rating; ?: indeterminate rating

*The NMF2S was derived from the previously developed NMF-Park questionnaire; however, no formal content validity evaluation of the modified version was reported. As the instrument differs from the original questionnaire (including the removal and modification of symptom items and changes to instructions), the relevance, comprehensiveness, and comprehensibility of the MFS-2 for the target population cannot be assumed. Consistent with COSMIN guidance, modifications to an existing PROM require re-evaluation of content validity. Consequently, risk of bias and content validity ratings were based on reviewer judgement rather than empirical evidence reported in the development study.

| **PSYCHLOPS** | | **PROM development study (Ashworth 2004)** | **Content validity study 1**  **(Ashworth, Robinson, Godfrey, Parmentier et al., 2005)** | **Rating of reviewers** | **OVERALL RATINGS PER PROM^3^** | **QUALITY OF EVIDENCE** |
| --- | --- | --- | --- | --- | --- | --- |
|  | | **+ / - / ?** | **+ / - / ?** | **+ / - / ?** | **+ / - / ± / ?** | **High, moderate, low, very low** |
| **Relevance** | |  |  |  |  |  |
| 1 | Are the included items relevant for the construct of interest?^1^ | + | + | + |  |  |
| 2 | Are the included items relevant for the target population of interest?^1^ | + | + | + |  |  |
| 3 | Are the included items relevant for the context of use of interest?^1^ | + | + | + |  |  |
| 4 | Are the response options appropriate? | + | + | + |  |  |
| 5 | Is the recall period appropriate? | + | + | + |  |  |
|  | **RELEVANCE RATING (+ / - / ± / ?)** | **+** | **+** | + | **+** | **Low** |
|  |  |  |  |  |  |  |
| **Comprehensiveness** | |  |  |  |  |  |
| 6 | Are all key concepts included? | ? |  | + |  |  |
|  | **COMPREHENSIVENESS RATING (+ / - / ± / ?)** | **?** |  | + | **+** | **Very low** |
|  |  |  |  |  |  |  |
| **Comprehensibility** | |  |  |  |  |  |
| 7 | Are the PROM instructions understood by the population of interest as intended? | - |  |  |  |  |
| 8 | Are the PROM items and response options understood by the population of interest as intended? | - |  |  |  |  |
| 9 | Are the PROM items appropriately worded? |  |  | + |  |  |
| 10 | Do the response options match the question? |  |  | + |  |  |
|  | **COMPREHENSIBILITY RATING (+ / - / ± / ?)** | **-** |  | + | **+** | **Very low** |
|  |  |  |  |  |  |  |
|  | **CONTENT VALIDITY RATING (+ / - / ± / ?)** | **+/-** |  | **+** | **+** | **Very low** |

+: sufficient rating; -: insufficient rating; ±: inconsistent rating; ?: indeterminate rating

| **Q Female LUTS** | | **PROM development study**  **(Bernstein et al., 1996)** | **Rating of reviewers** | **OVERALL RATINGS PER PROM^3^** | **QUALITY OF EVIDENCE** |
| --- | --- | --- | --- | --- | --- |
|  | | **+ / - / ?** | **+ / - / ?** | **+ / - / ± / ?** | **High, moderate, low, very low** |
| **Relevance** | |  |  |  |  |
| 1 | Are the included items relevant for the construct of interest?^1^ | ? | + |  |  |
| 2 | Are the included items relevant for the target population of interest?^1^ | ? | + |  |  |
| 3 | Are the included items relevant for the context of use of interest?^1^ | ? | + |  |  |
| 4 | Are the response options appropriate? | ? | + |  |  |
| 5 | Is the recall period appropriate? | ? | + |  |  |
|  | **RELEVANCE RATING (+ / - / ± / ?)** | ? | + | **+** | **Very low** |
|  |  |  |  |  |  |
| **Comprehensiveness** | |  |  |  |  |
| 6 | Are all key concepts included? | ? | + |  |  |
|  | **COMPREHENSIVENESS RATING (+ / - / ± / ?)** | ? | **+** | **+** | **Very low** |
|  |  |  |  |  |  |
| **Comprehensibility** | |  |  |  |  |
| 7 | Are the PROM instructions understood by the population of interest as intended? | ? |  |  |  |
| 8 | Are the PROM items and response options understood by the population of interest as intended? | ? |  |  |  |
| 9 | Are the PROM items appropriately worded? |  | + |  |  |
| 10 | Do the response options match the question? |  | + |  |  |
|  | **COMPREHENSIBILITY RATING (+ / - / ± / ?)** | **?** | **+** | **+** | **Very low** |
|  |  |  |  |  |  |
|  | **CONTENT VALIDITY RATING (+ / - / ± / ?)** | **?** | **+** | **+** | **Very low** |

+: sufficient rating; -: insufficient rating; ±: inconsistent rating; ?: indeterminate rating

| **QUAL-E** | | **PROM development study (Steinhauser et al., 2002)** | **Content validity study 1 (Wilkinson et al., 2014)** | **Rating of reviewers** | **OVERALL RATINGS PER PROM** | **QUALITY OF EVIDENCE** |
| --- | --- | --- | --- | --- | --- | --- |
|  | | **+ / - / ?** | **+ / - / ?** | **+ / - / ?** | **+ / - / ± / ?** | **High, moderate, low, very low** |
| **Relevance** | |  |  |  |  |  |
| 1 | Are the included items relevant for the construct of interest?^1^ | + |  | + |  |  |
| 2 | Are the included items relevant for the target population of interest?^1^ | + |  | + |  |  |
| 3 | Are the included items relevant for the context of use of interest?^1^ | + |  | + |  |  |
| 4 | Are the response options appropriate? | + |  | + |  |  |
| 5 | Is the recall period appropriate? | ? |  | + |  |  |
|  | **RELEVANCE RATING (+ / - / ± / ?)** | **+** |  | **+** | **+** | **Low** |
|  |  |  |  |  |  |  |
| **Comprehensiveness** | |  |  |  |  |  |
| 6 | Are all key concepts included? | + |  | + |  |  |
|  | **COMPREHENSIVENESS RATING (+ / - / ± / ?)** | **+** |  | **+** | **+** | **Low** |
|  |  |  |  |  |  |  |
| **Comprehensibility** | |  |  |  |  |  |
| 7 | Are the PROM instructions understood by the population of interest as intended? | + | ? |  |  |  |
| 8 | Are the PROM items and response options understood by the population of interest as intended? | + | ? |  |  |  |
| 9 | Are the PROM items appropriately worded? |  |  | + |  |  |
| 10 | Do the response options match the question? |  |  | + |  |  |
|  | **COMPREHENSIBILITY RATING (+ / - / ± / ?)** | **+** | **?** | **+** | **+** | **Low** |
|  |  |  |  |  |  |  |
|  | **CONTENT VALIDITY RATING (+ / - / ± / ?)** | **+** |  | **+** | **+** | **Low** |

+: sufficient rating; -: insufficient rating; ±: inconsistent rating; ?: indeterminate rating

| **QUAL-EC** | | **PROM development study**  **(Lo et al., 2011)** | **Rating of reviewers** | **OVERALL RATINGS PER PROM^3^** | **QUALITY OF EVIDENCE** |
| --- | --- | --- | --- | --- | --- |
|  | | **+ / - / ?** | **+ / - / ?** | **+ / - / ± / ?** | **High, moderate, low, very low** |
| **Relevance** | |  |  |  |  |
| 1 | Are the included items relevant for the construct of interest?^1^ | + | + |  |  |
| 2 | Are the included items relevant for the target population of interest?^1^ | + | + |  |  |
| 3 | Are the included items relevant for the context of use of interest?^1^ | + | + |  |  |
| 4 | Are the response options appropriate? | + | + |  |  |
| 5 | Is the recall period appropriate? | + | + |  |  |
|  | **RELEVANCE RATING (+ / - / ± / ?)** | + | **+** | **+** | **Moderate** |
|  |  |  |  |  |  |
| **Comprehensiveness** | |  |  |  |  |
| 6 | Are all key concepts included? | - | + |  |  |
|  | **COMPREHENSIVENESS RATING (+ / - / ± / ?)** | **-** | **+** | **+** | **Very low** |
|  |  |  |  |  |  |
| **Comprehensibility** | |  |  |  |  |
| 7 | Are the PROM instructions understood by the population of interest as intended? | + |  |  |  |
| 8 | Are the PROM items and response options understood by the population of interest as intended? | + |  |  |  |
| 9 | Are the PROM items appropriately worded? |  | + |  |  |
| 10 | Do the response options match the question? |  | + |  |  |
|  | **COMPREHENSIBILITY RATING (+ / - / ± / ?)** | **+** | **+** | **+** | **Moderate** |
|  |  |  |  |  |  |
|  | **CONTENT VALIDITY RATING (+ / - / ± / ?)** | **+/-** | **+** | **+** | **Moderate** |

+: sufficient rating; -: insufficient rating; ±: inconsistent rating; ?: indeterminate rating

| **SF-LDQ** | | **PROM development study**  **(Fraser et al., 2007)** | **Content validity study 1**  **(Fraser et al., 2007)** | **Rating of reviewers** | **OVERALL RATINGS PER PROM^3^** | **QUALITY OF EVIDENCE** |
| --- | --- | --- | --- | --- | --- | --- |
|  | | **+ / - / ?** | **+ / - / ?** | **+ / - / ?** | **+ / - / ± / ?** | **High, moderate, low, very low** |
| **Relevance** | |  |  |  |  |  |
| 1 | Are the included items relevant for the construct of interest?^1^ | ? |  | + |  |  |
| 2 | Are the included items relevant for the target population of interest?^1^ | ? |  | + |  |  |
| 3 | Are the included items relevant for the context of use of interest?^1^ | ? |  | + |  |  |
| 4 | Are the response options appropriate? | ? |  | + |  |  |
| 5 | Is the recall period appropriate? | ? |  | + |  |  |
|  | **RELEVANCE RATING (+ / - / ± / ?)** | ? |  | **+** | **+** | **Very low** |
|  |  |  |  |  |  |  |
| **Comprehensiveness** | |  |  |  |  |  |
| 6 | Are all key concepts included? | ? |  | + |  |  |
|  | **COMPREHENSIVENESS RATING (+ / - / ± / ?)** | ? |  | **+** | **+** | **Very low** |
|  |  |  |  |  |  |  |
| **Comprehensibility** | |  |  |  |  |  |
| 7 | Are the PROM instructions understood by the population of interest as intended? | ? | + |  |  |  |
| 8 | Are the PROM items and response options understood by the population of interest as intended? | ? | + |  |  |  |
| 9 | Are the PROM items appropriately worded? |  |  | + |  |  |
| 10 | Do the response options match the question? |  |  | + |  |  |
|  | **COMPREHENSIBILITY RATING (+ / - / ± / ?)** | **?** | **+** | **+** | **+** | **Moderate** |
|  |  |  |  |  |  |  |
|  | **CONTENT VALIDITY RATING (+ / - / ± / ?)** | **?** |  | **+** | **+** | **Very low** |

+: sufficient rating; -: insufficient rating; ±: inconsistent rating; ?: indeterminate rating

| **UDI (English) (Content validity study: Italian)** | | **PROM development study (Shumaker et al., 1994)** | **Content validity study 1   (Artibani et al., 2006)** | **Rating of reviewers** | **OVERALL RATINGS PER PROM^3^** | **QUALITY OF EVIDENCE** |
| --- | --- | --- | --- | --- | --- | --- |
|  | | **+ / - / ?** | **+ / - / ?** | **+ / - / ?** | **+ / - / ± / ?** | **High, moderate, low, very low** |
| **Relevance** | |  |  |  |  |  |
| 1 | Are the included items relevant for the construct of interest?^1^ | + |  | + |  |  |
| 2 | Are the included items relevant for the target population of interest?^1^ | + |  | + |  |  |
| 3 | Are the included items relevant for the context of use of interest?^1^ | + |  | + |  |  |
| 4 | Are the response options appropriate? | + |  | + |  |  |
| 5 | Is the recall period appropriate? | ? |  | ? |  |  |
|  | **RELEVANCE RATING (+ / - / ± / ?)** | **+** |  | **+** | **+** | **Very low** |
|  |  |  |  |  |  |  |
| **Comprehensiveness** | |  |  |  |  |  |
| 6 | Are all key concepts included? | ? |  | + |  |  |
|  | **COMPREHENSIVENESS RATING (+ / - / ± / ?)** | **?** |  | **+** | **+** | **Very low** |
|  |  |  |  |  |  |  |
| **Comprehensibility** | |  |  |  |  |  |
| 7 | Are the PROM instructions understood by the population of interest as intended? | ? | + |  |  |  |
| 8 | Are the PROM items and response options understood by the population of interest as intended? | ? | + |  |  |  |
| 9 | Are the PROM items appropriately worded? |  |  | + |  |  |
| 10 | Do the response options match the question? |  |  | + |  |  |
|  | **COMPREHENSIBILITY RATING (+ / - / ± / ?)** | **?** | **+** | **+** | **+** | **Very low** |
|  |  |  |  |  |  |  |
|  | **CONTENT VALIDITY RATING (+ / - / ± / ?)** | **?** |  | **+** | **+** | **Very low** |

+: sufficient rating; -: insufficient rating; ±: inconsistent rating; ?: indeterminate rating

| **U-UDI** | | **PROM development study**  **(Brown et al., 1999)** | **Rating of reviewers** | **OVERALL RATINGS PER PROM^3^** | **QUALITY OF EVIDENCE** |
| --- | --- | --- | --- | --- | --- |
|  | | **+ / - / ?** | **+ / - / ?** | **+ / - / ± / ?** | **High, moderate, low, very low** |
| **Relevance** | |  |  |  |  |
| 1 | Are the included items relevant for the construct of interest?^1^ | + | + |  |  |
| 2 | Are the included items relevant for the target population of interest?^1^ | + | + |  |  |
| 3 | Are the included items relevant for the context of use of interest?^1^ | + | + |  |  |
| 4 | Are the response options appropriate? | + | + |  |  |
| 5 | Is the recall period appropriate? | - | + |  |  |
|  | **RELEVANCE RATING (+ / - / ± / ?)** | **+** | **+** | **+** | **Very low** |
|  |  |  |  |  |  |
| **Comprehensiveness** | |  |  |  |  |
| 6 | Are all key concepts included? | ? | + |  |  |
|  | **COMPREHENSIVENESS RATING (+ / - / ± / ?)** | **?** | **+** | **+** | **Very low** |
|  |  |  |  |  |  |
| **Comprehensibility** | |  |  |  |  |
| 7 | Are the PROM instructions understood by the population of interest as intended? | - |  |  |  |
| 8 | Are the PROM items and response options understood by the population of interest as intended? | - |  |  |  |
| 9 | Are the PROM items appropriately worded? |  | + |  |  |
| 10 | Do the response options match the question? |  | + |  |  |
|  | **COMPREHENSIBILITY RATING (+ / - / ± / ?)** | **-** | **+** | **+** | **Very low** |
|  |  |  |  |  |  |
|  | **CONTENT VALIDITY RATING (+ / - / ± / ?)** | **+/-** | **+** | **+** | **Very low** |

+: sufficient rating; -: insufficient rating; ±: inconsistent rating; ?: indeterminate rating
