# Supplementary material for: How is the “most bothersome symptom” construct measured across health conditions? A systematic review of patient-reported outcome measures
Source: Patient Relat Outcome Meas. 2026 Sep 20;17(1):107–22. doi: 10.1080/1179271X.2026.2732841 (PMC13596887; doi:10.1080/1179271X.2026.2732841)
Supplement: Supplementary file 3 [file DPRO_A_2732841_SM9472.docx]

**Table S1 Conditions relating to body systems or types of diseases measured by included PROMs (n = 171)**

| Body System / Disease | Total, *n (%)* | Direct PROMs (n=36) | | Indirect PROMs (n=135) | |
| --- | --- | --- | --- | --- | --- |
|  |  | Assessed (n=14) | Unassessed (n=22) | Assessed (n=120) | Unassessed (n=15) |
| Urinary | 34 (20%) | 6 | 1 | 27 | 1 |
| Reproductive | 24 (14%) | 1 | 2 | 19 | 2 |
| Cancer | 19 (11%) | 2 | 2 | 13 | 2 |
| Allergy / Immune | 17 (10%) |  | 3 | 11 | 3 |
| Psychological | 17 (10%) | 1 |  | 16 |  |
| Digestive | 15 (9%) | 1 | 5 | 6 | 3 |
| Respiratory | 14 (8%) | 1 | 1 | 7 | 5 |
| Nervous | 13 (8%) | 1 | 8 | 3 | 1 |
| Sensory | 11 (7%) |  |  | 11 |  |
| Integumentary | 7 (4%) |  | 1 | 5 | 1 |
| Endocrine | 6 (4%) |  |  | 6 |  |
| Cardiovascular | 5 (3%) | 1 |  | 4 |  |
| Genetic | 4 (2%) |  | 2 | 1 | 1 |
| Lymphatic | 4 (2%) |  |  | 4 |  |
| Infection | 4 (2%) |  |  | 4 |  |
| Circulatory | 2 (1%) |  |  |  | 2 |
| Musculoskeletal | 2 (1%) |  | 1 | 1 |  |
| Any condition or population | 6 (3%) | 3 | 1 | 2 |  |

Direct + Assessed PROMs n=17 due to 1 PROM belonging to 3 categories; Direct + Unassessed PROMS n = 27 due to 5 PROMs belonging to 2 categories; Indirect + Assessed PROMs n = 140 due to 4 PROMs not being able to be categorised, 14 PROMs belonging to 2 categories, and 5 PROMs belonging to 3 categories; Indirect + Unassessed PROMs n = 21 due to 6 PROMs belonging to 2 categories.

**Table S2 Complete characteristics of Direct + Assessed PROMs (n = 14)**

| **PROM** | **Construct measured** | **Population or health condition** | **Instructions and/or question wording** | **‘MBS’ items / total items (%)** | **Scale type and wording** | **Recall period** | **Average time to complete (minutes)** | **Open- or closed-ended items** | **Method of administration** | **‘Bother’ synonyms in manuscript** | **‘Bother’ synonyms in PROM** |
| --- | --- | --- | --- | --- | --- | --- | --- | --- | --- | --- | --- |
| GSM-SVTAQ | Most bothersome symptom | Menopause | During the past three months, which of the following vaginal symptoms has been the single most bothersome symptom when you have it?  During the past three months, which of the following sexual symptoms has been the single most bothersome symptom when you have it?  During the past three months, which of the following urinary symptoms has been the single most bothersome symptom when you have it? | 3/42 (7%) | 5-point Likert (bother): 1 Almost always or always, 2 Most times (more than half the time), 3 Sometimes (about half the time), 4 A few times (less than half the time), 5 Almost never or never | 3 months | NR | CE | SA | Bother, disturbance, importance | Bother |
| MQOL | Top 3 most troublesome symptoms | Life threatening illnesses | THIS QUESTIONNAIRE HAS 17 QUESTIONS, WHICH WILL HELP US KNOW HOW YOU ARE FEELING. PLEASE DO NOT LEAVE OUT ANY QUESTIONS, IT IS IMPORTANT THAT THEY ALL BE ANSWERED.   - For the next three questions, please list the physical symptoms that are the biggest problem to you. - Choose the number that best shows how much of a problem each one was for you IN THE PAST TWO DAYS. - Some examples are: pain, tiredness, weakness, nausea, constipation, diarrhoea, trouble sleeping, shortness of breath, lack of appetite. Feel free to use others if necessary. - If you have less than three physical problems, write ’none’ for that question.   My troublesome symptom 1 is:  My troublesome symptom 2 is:  My troublesome symptom 3 is: | 3/17 (18%) | 7-point Likert (problem): 1 no problem, 2, 3, 4, 5, 6, 7 tremendous problem | 2 days | Completed on own: 15-20  Supervised verbally: 15-35 | OE | SA | Problem, trouble | Problem, trouble |
| MUDI | Most bothersome symptom | Male urinary incontinence | Older men report various urinary symptoms. Each question below asks about a different symptom. Thinking back over your symptoms during the past 4 weeks, please answer yes or no to each question. Be sure to answer all questions completely. | 1/55 (2%) + 1 optional OE | 4-piont Likert Type (bother): Not at all, Slightly, Moderately, Greatly | 4 weeks | 20 | CE + 1 optional OE | SA | Bother | Bother, distress |
| MYMOP1 | Top 2 most bothersome symptoms | Any population or condition | Choose one or two symptoms which bother you the most. Write them on the dotted lines. Now consider how bad each symptom is, over the last week, and score it by circling your chosen number. | 3/6 (50%) | 7-point Likert (good/bad): 0 As good as it could be, 1, 2, 3, 4, 5, 6 As bad as it could be | 1 week | NR | OE | SA | Affect, importance | Bother |
| MYMOP2 | Top 2 most bothersome symptoms | Any population or condition | Choose one or two symptoms (physical or mental) which bother you the most. Write them on the lines. Now consider how bad each symptom is, over the last week, and score it by circling your chosen number. | 2/6 (33%) | 7-point Likert (good/bad): 0 As good as it could be, 1, 2, 3, 4, 5, 6 As bad as it could be | 1 week | NR | OE | SA | Affect, importance | Bother |
| NMF2S | Most bothersome symptom | Parkinson’s disease | We suggest you use a numerical scale to measure the intensity of non-motor fluctuations in Parkinson's disease, both in the "OFF" and "ON" phases. Circle the number from 0 to 10 that best corresponds, in your opinion, to the importance of non-motor fluctuations. A score of 0 corresponds to "no sign of fluctuation", while a score of 10 corresponds to the maximum intensity of the sign. Base your assessment on the signs you are experiencing at the precise moment you fill in the questionnaire.  In this list, in the present moment, circle the sign that is most bothersome for you. | 1/13 (8%) | 11-point NRS (intensity): 0, 1, 2, 3, 4, 5, 6, 7, 8, 9, 10  *Different anchoring labels for 0 and 10 were provided for each of the symptom items* | Precise moment you fill in the questionnaire | NR | CE | SA | Bother, severity, disabling | Bother, importance, intensity |
| POSQ | Most bothersome symptom | Overactive bladder | Instructions: Please read each of the following questions carefully. Please answer the questions to the best of your ability and do not skip questions. There are no right or wrong answers.  We are interested in learning about how bothered you are by your symptoms. Please answer every question by marking a (tick) or a (cross) in the box that best represents how you feel. If you did not experience a symptom, please mark “Not at all”.  Please mark (x) *the one bladder symptom that bothers you the most.* | 1/5 (20%) | 5-point Likert (bother): 1 Not at all, 2 A little, 3 Moderately, 4 A great deal, 5 A very great deal | 2 weeks | NR | CE | SA | Bother | Bother |
| PSYCHLOPS | Top 2 most troublesome symptoms | Any psychological condition | Pre therapy: A questionnaire about you and how you are feeling – now that you are starting therapy.  Choose the problem that troubles you most. *(Please write it in the box below.)* | 2/9 (22%) | 6-point Likert Type (affect): 0 Not at all affected, 1, 2, 3, 4, 5 Severely affected  5-point Likert (length of time concerned): Under one month, Between one and three months, Over three months but under one year, One to five years, Over five years | Not reported | 5 | OE | SA | Importance, trouble | Trouble |
|  |  |  |  |  |  |  |  |  |  |  |  |
|  |  |  | During therapy: A questionnaire about you and how you are feeling – now that you are having therapy.  The is the problem you said troubled you the most when we first asked. *(Therapist – please write it in the box below.)* How much has it affected you over the last week? *(Please tick one box below.)* | 2/5 (40%) | 6-point Likert Type (affect): 0 Not at all affected, 1, 2, 3, 4, 5 Severely affected | 1 week |  |  |  |  |  |
|  |  |  | Post therapy: A questionnaire about you and how you are feeling – now that you are finishing therapy.  This is the problem you said troubled you the most when we first asked. *(Therapist – please write it in the box below.)* How much has it affected you over the last week? *(Please tick one box below.)* | 2/6 (30%) | 6-point Likert Type (affect): 0 Not at all affected, 1, 2, 3, 4, 5 Severely affected | 1 week |  |  |  |  |  |
| Questionnaire for female lower urinary tract symptoms and their impairment of quality of life | Most troublesome symptom | Females with lower urinary tract dysfunction | This questionnaire is designed to find out what sorts of symptoms are experienced by women with urinary trouble and how much of a problem they are. We kindly ask you to answer the following questions based on what has troubled you most within the last month. Please put a tick in one box for each question. If you have difficulty in answering some of the questions, please ask the nurse or the doctor in the outpatient clinic.  Which of your urinary symptoms trouble you most at the moment? Please describe the problems in your own words or write the numbers of those of the aforementioned questions that best describe your trouble: | 1/66 (27%) | 4-point Likert Type (problem): No, Yes but only a small problem, Yes it is a problem, Yes it is a serious problem | 1 month | NR | CE | SA | Bother, trouble | Problem, trouble |
| QUAL-E | Most bothersome symptom | Cancer, congestive heart failure, COPD, end-stage renal disease | I’d like you to think back over the last month. Please tell me the three physical symptoms or problems that have bothered you the most during that time. Some examples are pain, nausea, lack of energy, confusion, depression, anxiety, and shortness of breath.  Which of these symptoms or problems has bothered you the most this past week? | 4/28 (14%) | 5-point Likert (frequency): 1 Rarely, 2 A few times, 3 Fairly often, 4 Very often, 5 Most of the time  5-point Likert (importance): 1 Not at all, 2 A little bit, 3 A moderate amount, 4 Quite a bit, 5 Completely  5-point Likert (interference): 1 Not at all, 2 A little bit, 3 A moderate amount, 4 Quite a bit, 5 Completely  5-point Likert (severity): 1 Very mild, 2 Mild, 3 Moderate, 4 Severe, 5 Very severe  5-point Likert (worry): 1 Not at all, 2 A little bit, 3 A moderate amount, 4 Quite a bit, 5 Completely | 1 week | 15 | OE | SA | Distress | Bother |
| QUAL-EC | Most bothersome symptom | Advanced cancer | I’d like you to think back over the last month. Please tell me the three physical or emotional symptoms that have bothered you the most during that time. Some examples are pain, nausea, lack of energy, confusion, depression, anxiety, and shortness of breath.  Of the symptoms listed above, which one symptom has bothered you the most this past week? | 4/22 (9%) | 5-point Likert (frequency): 1 Rarely, 2 A few times, 3 Fairly often, 4 Very often, 5 Most of the time  5-point Likert (interference): 1 Not at all, 2 A little bit, 3 A moderate Amount, 4 Quite a bit, 5 Completely  5-point Likert (severity): 1 Very mild, 2 Mild, 3 Moderate, 4 Severe, 5 Very severe | 1 week | NR | OE | SA | Bother | Bother |
| SF-LDQ | Most troublesome symptom | Dyspepsia | Which, if any, of these symptoms has been the most troublesome to you in the last 2 months? Please tick one box only | 1/9 (11%) | 5-point Likert (frequency): Not at all, Less than once a month, Between once a month and once a week, Between once a week and once a day, Once a day or more  5-point Likert (interference): Not at all, Less than once a month, Between once a month and once a week, Between once a week and once a day, Once a day or more | 2 months | NR | CE | SA | Trouble | Trouble |
| UDI | Most bothersome symptom | Women with urinary incontinence | The next set of questions deals specifically with your accidental urine loss and/or prolapse.  The following symptoms have been described by women who experience accidental urine loss and/or prolapse. Please indicate which symptoms you are now experiencing, and how bothersome they are for you. Be sure to answer all items.  Please go back and review all the symptoms listed for question 1 (a-t). Write the letter of the symptom which has bothered you the most. (Please write in only one letter) | 1/39 + 1 optional OE | 4-point Likert Type (bother): 0 Not at all, 1 Slightly, 2 Moderately, 3 Greatly | NR | NR | CE + 1 optional OE | SA | Bother | Bother, distress |
| U-UDI | Most bothersome symptom | Urge urinary incontinence or mixed urinary incontinence with a primary urge component | The next set of questions deals specifically with your urine leakage and/or bladder problems. The following symptoms have been described by people who have bladder problems and/or experience urine leakage. Please indicate which symptoms you have experienced *in the past four weeks*, and how bothersome they were for you. Be sure to answer all items.  *In the past four weeks did you experience the following problems (yes/no), and if yes, how much did it bother you (Not at all, Slightly, Moderately, Greatly)*  Please go back and review all the symptoms. Write the question number of the symptom which has bothered you the most ___ (Please write in only one number.) | 1/19 (5%) | 4-point Likert Type (bother): Not at all, Slightly, Moderately, Greatly | 4 weeks | NR | CE | SA | Bother | Bother, distress |

CE: closed-ended; GSM-SVTAQ: Genitourinary Syndrome of Menopause (GSM) Symptoms and Vaginal Treatments Acceptability Questionnaire; MQOL: McGill Quality of Life Questionnaire; MUDI: Male Urogenital Distress Inventory; MYMOP 1: Measure Yourself Medical Outcome Profile; MYMOP2: Measure Yourself Medical Outcome Profile – Revised version; NMF2S: Non-Motor Fluctuations Severity Scale; NR: not reported; OE: open-ended; POSQ: Primary OAB Symptom Questionnaire; PROM: patient-reported outcome measure; PSYCHLOPS: Psychological Outcome Profiles; Q Female LUTS: Questionnaire for female lower urinary tract symptoms and their impairment of quality of life; QUAL-E: Quality of Life at the End of Life; QUAL-EC: QUAL-E Cancer; SA: self-administered; SF-LDQ; Short Form Leeds Dyspepsia Questionnaire; UDI: Urogenital Distress Inventory; U-UDI: Urge Urinary Distress Inventory

**Table S3 Summary of characteristics of Direct PROMs (n = 36)**

| Characteristics | | Assessed PROMs (n=14) | Unassessed PROMs (n=22) |
| --- | --- | --- | --- |
| Recall period | Precise moment you fill in the questionnaire | 1 |  |
|  | 2 days | 1 |  |
|  | 1 week | 6 | 1 |
|  | 2 weeks | 1 | 1 |
|  | Last 2 weeks of life |  | 1 |
|  | 4 weeks | 2 | 1 |
|  | 1 month | 1 |  |
|  | 2 months | 1 |  |
|  | 3 months | 1 |  |
|  | 6 months |  | 1 |
|  | Not reported | 2 | 17 |
| Open or closed-item lists | Open-ended | 6 | 8 |
|  | Closed-ended | 6 | 9 |
|  | Closed-ended + 1 optional open-ended | 2 | 1 |
|  | Not reported |  | 4 |
| Scale type | 4-point Likert Type | 4 | 7 |
|  | 5-point Likert | 13 | 1 |
|  | 6-point Likert Type | 3 | 1 |
|  | 6-point Likert Type (Pictorial) |  | 1 |
|  | 7-point Likert | 3 | 1 |
|  | 3-point NRS |  | 1 |
|  | 4-point NRS |  | 5 |
|  | 5-point NRS |  | 2 |
|  | 10-point NRS |  | 1 |
|  | 11-point NRS | 1 | 7 |
|  | 10cm VAS |  | 2 |
|  | 10-point VAS |  | 1 |
|  | 5-point scale (unspecified) |  | 1 |
|  | Binary |  | 2 |
| Method of completion | Self-administered | 14 | 14 * |
|  | Interviewer-administered |  | 6† |
|  | Not reported |  | 2 |

* N=1 PROM was proxy-administered; † N = 1 PROM was completed via a focus-group interview and N = 1 PROM was completed via a face-to-face semi-structured concept elicitation interview; NRS: numerical rating scale; VAS: visual analogue scale. N=16 for Direct Assessed PROMs recall period due to one PROM having 3 different recall periods depending on time of administration; N = 24 for Direct Assessed PROMs scale type due to four PROMs having more than 1 rating scale; N = 33 for Direct Unassessed PROMs scale type due to eight PROMs having more than 1 rating scale.

**Table S4 Direct + Unassessed PROMs measuring the construct of the ‘most bothersome symptom’ (n = 18)**

| **Paper reference**  **(PROM name if applicable)** | **Population or health condition** | **Measuring what?** | **Scale type and wording** | **Recall period** | **Open- or closed-ended item list** | **Number of ‘bother’ items (symptoms)** | **Method of completion** | **‘Bother’ synonyms used in manuscript** | **‘Bother’ synonyms used in PROM / description of PROM** |
| --- | --- | --- | --- | --- | --- | --- | --- | --- | --- |
| Allergies in America, 2006 | Allergic rhinitis | MBS | 4-point Likert Type (bother): extremely bothersome, moderately bothersome, slightly bothersome, not bothersome | NR | CE | 10 | IA telephone survey | Bother | Bother |
| Day, 2004 (MYMOP-P) | Any population or condition | Top 2 MBS | 6-point Likert Type (good/bad): 0 As GOOD as it could be, 1, 2, 3, 4, 5 As BAD as it could be  6-point Likert Type Pictorial: Faces ranging from 0 Happy to 5 Extreme crying | 1 week | OE | 2 | SA | Distress, severity | Bother |
| Javidnia et al., 2021 (PD-PROP) | Parkinson’s Disease | Up to top 5 MBS | 4-point Likert Type (severity): 0 Not at all, 1 Mildly (minimally or rarely), 2 Moderately (more often than not), 3 Severely (plenty or all of the time) | NR | OE | 5 | SA | Bother | Bother |
| Kang et al., 2011 | Men with lower urinary tract symptoms or benign prostatic hyperplasia being treated by tamsulosin | MBS | 5-point scale – unspecified type (bother): NR | NR | NR | NR | NR | Bother | Bother |
| Karamanolis et al., 2006 † | Functional dyspepsia | MBS | 4-point NRS (intensity): 0 absent, 1 mid, 2 moderate, 3 severe / interfering with daily activities | NR | CE | 8 | SA questionnaire | Bother | Bother |
| Lembo et al., 1999 (BSQ) ‡ | Irritable bowel syndrome | MBS | 10cm VAS (intensity): anchors ranging from No sensation – Extremely intense  10cm VAS (unpleasantness): anchors ranging from Neutral – Very intolerable | 2 weeks | CE | 1 | SA | Bother, disturb | Bother |
| Lipton et al., 2021 | Chronic migraine | MBS | 7-point Likert (severity): Very much improved, much improved, minimally improve, no change, minimally worse, much worse, very much worse | NR  Measured at baseline, weeks 4 8 12 16 20 24 32 | OE | NR | IA | Bother | Bother |
| Nelson & Esty, 2010 | Traumatic brain injury | MBS | 11-point NRS (bother): NR, mentions has ‘appropriate anchors’ | NR | NR | NR | NR | Bother | Bother |
| Nicholson et al., 2018 | Amyotrophic lateral sclerosis | MBS | 4-point Likert Type (severity): None, Mild, Moderate, Severe | 4-weeks | CE+ 1 optional OE | 1 + 6 additional questions if ‘other’ symptom is identified in open-ended field | SA | Bother, severity | Bother |
| Park et al., 2010 | Eosinophilic esophagitis | MBS | 4-point NRS (frequency): 0, 1, 2, 3  4-point NRS (severity): 0, 1, 2, 3 | NR | CE | 7 | SA questionnaire | Bother | Bother |
| Shega et al., 2008 | Palliating patients with dementia | MBS | 4-point NRS (bother / distress): 1 a little bit, 2 somewhat, 3 quite a bit, 4 very much  4-point NRS (frequency): 1 occasional, 2 half of the time, 3 most of the time, 4 all of the time  3-point NRS (severity): 1 not at all, 2 moderately severe, 3 extremely severe | Last 2 weeks of life | OE | N/A | Proxy-completed telephone interview | Bother, distress | Bother |
|  |  |  |  |  |  |  |  |  |  |
|  |  |  |  |  |  |  |  |  |  |
| Shoulson, 2013 (HD-PROP) ‡ | Huntington’s Disease | Up to top 8 MBS | 4-point Likert Type (severity): 1 Not at all, 2 Mildly (minimally or rarely), 3 Moderately (more often than not), 4 Severely (plenty or all the time) | NR | OE | 8 | IA | Bother | Bother |
| Stokes et al., 2017‡ | Barth syndrome | MBS | 11-point NRS (bother): 0 no bother, 10 most bothersome  11-point NRS (impact): 0 no impact, 10 most impactful  11-point NRS (severity): 0 no severity, 10 most severe  11-point NRS (worry): 0 no worry, 10 most worrisome | NR | OE | N/A | Face to face semi-structured concept elicitation interviews | Bother | / |
|  |  |  |  |  |  |  |  |  |  |
|  |  |  |  |  |  |  |  |  |  |
|  |  |  |  |  |  |  |  |  |  |
| Tack et al., 2014 | Upper abdominal symptoms of functional dyspepsia | MBS | 5-point NRS (frequency): 1 less than once per week, 2 once per week, 3 two to three times per week, 4 four to five times per week, 5 daily  5-point NRS (severity): 1 very mild, 2 mild - present but not bothersome, 3 moderate - bothersome, 4 severe, interfering with daily activities, 5 severe, rendering daily activities impossible | NR | CE | 9 | SA questionnaire | Bother | Bother |
|  |  |  |  |  |  |  |  |  |  |
| Tornatta et al., 2009 | Cervical cancer survivors | Top 3 MBS | 11-point NRS (severity): 0 do not have the symptom, 10 as severe as I can imagine | NR | CE | 17 | SA survey | Bother | Bother |
| Vakil et al., 2015 | Overlapping functional disorders including constipation-predominant irritable bowel syndrome, functional dyspepsia, chronic idiopathic constipation, and gastroesophageal reflux disease | Top 3 MBS | 5-point Likert (bother): 5 Extremely Bothersome, 4 Very Bothersome, 3 Somewhat Bothersome, 2 A Little Bothersome, 1 Not at all Bothersome | NR | CE | 16 | SA web-based survey | Bother, burden | Bother |
| FDA, 2018 ‡ | Acute treatment of migraine (drug trials) | MBS | 4-point Likert Type (severity: pain): 0 None, 1 Mild, 2 Moderate, 3 Severe  Binary (presence: nausea, photophobia, phonophobia, presence): present, absent | NR  Identification can take place before the attack is treated (e.g., at baseline visit) or at the time of the attack but before administration of the study drug  Is measured a co-primary endpoint where the effect on the most bothersome migraine associated symptom 2 hours after dose | CE | 4 | SA | Bother, importance | / |
| FDA, 2003 ‡ | Menopause associated vulvar and vaginal atrophy and vasomotor symptoms (estrogen and estrogen/progestin drug products) | MBS | 4-point Likert Type (severity: vaginal dryness; vaginal and/or vulvar irritation/itching; dysuria; vaginal pain associated with sexual activity): None, mild, moderate, severe  Binary (presence: vaginal bleeding associated with sexual activity): Present, absent | NR  Recommends 12 weeks mean change during trial as co-primary endpoint | CE | 5 | SA | Bother | / |

† Study used an established PROM (ref Tack 1998) which measure the ‘intensity’ of symptoms as part of MBS assessment which was unique to this study. ‡ Partial or incomplete PROM only able to be retrieved, therefore some missing data. CE: closed-ended; HD-PROP Huntington’s Disease Patient Reports of Problems; IA: interviewer-administered; MBS: most bothersome symptom; MYMOP-P: MYMOP-Pictorial; NR: not reported; NRS: numerical rating scale; OE: open-ended; PD-PROP: Parkinson’s Disease Patient Reports of Problems; SA: self-administered; VAS: visual analogue scale.

**Table S5 Direct + Unassessed PROMs measuring the construct of the ‘most troublesome symptom’ (n = 2)**

| **Paper reference** | **Population or health condition** | **Measuring what?** | **Scale type and wording** | **Recall period** | **Open- or closed-ended item list** | **Number of ‘bother’ items (symptoms)** | **Method of completion** | **‘Bother’ synonyms used in manuscript** | **‘Bother’ synonyms used in PROM / description of PROM** |
| --- | --- | --- | --- | --- | --- | --- | --- | --- | --- |
| Globe et al., 2009 | Psoriasis | Most troublesome symptom | 10-point VAS (trouble): 1 least troublesome, 10 most troublesome | NR | Open-ended component with additional probing for common symptoms | NR | Focus group interview | Trouble | Trouble |
| Vuletic, 2020 | Generalised dystonia | Most troublesome symptom | 10-point NRS (intensity): 1-10 | 6 months | NR | NR | SA survey | Trouble, hardest | Trouble, intensity |

VAS: visual analogue scale; NR: not reported; NRS: numerical rating scale; SA: self-administered

**Table S6 Direct + Unassessed PROMs measuring the construct of the ‘most annoying symptom’ (n = 2)**

| **Paper reference** | **Population or health condition** | **Measuring what?** | **Scale type and wording** | **Recall period** | **Open- or closed-ended item list** | **Number of ‘bother’ items (symptoms)** | **Method of completion** | **‘Bother’ synonyms used in manuscript** | **‘Bother’ synonyms used in PROM / description of PROM** |
| --- | --- | --- | --- | --- | --- | --- | --- | --- | --- |
| Alsirafy et al., 2016 | Newly diagnosed incurable cancer patients | Most annoying symptom | 4-point Likert Type (severity): None, mild, moderate, severe | NR | Open-ended to identify ‘most annoying symptom’. Closed-ended to rate the severity of the 57 symptoms. | 57 | IA survey | Burden | Annoy |
| Boukhettala & Bourgeon, 2012 | Dust mite allergy | Most annoying symptom | 11-point NRS (discomfort): 0 no discomfort, 10 severe discomfort | NR | NR | NR | SA survey | Annoy, discomfort | Discomfort |

IA: interviewer-administered; NR: not reported; NRS: numerical rating scale; SA: self-administered

**Table S7 Indirect + Assessed PROMs measuring the construct of ‘bother’ (n = 87)**

| **PROM (abbreviation)** | **Population or health condition** | **Instructions and/or question wording** | **‘Bother’ items / total items (%)** | **Scale type and wording** | **Recall period** | **Average time to complete (minutes)** | **Open- or closed-ended** | **Method of completion** | **‘Bother’ synonyms used in manuscript** | **‘Bother’ synonyms used in PROM** |
| --- | --- | --- | --- | --- | --- | --- | --- | --- | --- | --- |
| Overactive Bladder Questionnaire (Short Form) (OAB-q SF) | Continent and incontinent patients with overactive bladder | This questionnaire asks about how much you have been bothered by selected bladder symptoms during the past 4 weeks. Please place a (tick) or (cross) in the box that best describes the extent to which you were bothered by each symptom during the past 4 weeks. There are no right or wrong answers. Please be sure to answer every question.  During the past 4 weeks, how bothered were you by… | 6/19 (32%) | 6-point Likert Type (bother): 1 Not at all, 2 A little bit, 3 Somewhat, 4 Quite a bit, 5 A great deal, 6 A very great deal | 4 weeks | NR | CE | SA | Bother | Bother |
| Overactive Bladder Questionnaire (OAB-q) | Adult overactive bladder | This questionnaire asks about how much you have been bothered by selected bladder symptoms during the past 4 weeks. Please place a (tick) or (cross) in the box that best describes the extent to which you were bothered by each symptom during the past 4 weeks. There are no right or wrong answers. Please be sure to answer every question.  During the past 4 weeks, how bothered were you by… | 8/33 (24%) | 6-point Likert Type (bother): 1 Not at all, 2 A little bit, 3 Somewhat, 4 Quite a bit, 5 A great deal, 6 A very great deal | 4 weeks | 5-10 | CE | SA | Bother, distress | Bother |
| Pelvic Floor Bother Questionnaire (PFBQ) | Women with pelvic floor disorders | The following is a questionnaire about your pelvic health. All information is strictly confidential. Please mark (X) in the box that best describes your symptoms in the last month.  If yes, how much does it bother you? | 9/18 (50%) | 5-point Likert (bother): Not at all, Only a little bit, Somewhat, A moderate amount, A lot | 1 month | 4.6±1.1 | CE | SA | Bother | Bother |
| Australian Pelvic Floor Questionnaire (APFQ) | Female urogenital diseases | Please circle your most applicable answer. Consider your experience during the last month.  How much does your ___ bother you? | 4/42 (10%) + 3 optional open-ended | 4-point Likert Type (bother): 0 Not at all 1 Slightly 2 Moderately 3 Greatly | 1 month | NR | CE + 3 optional OE | IA | Bother | Bother |
| Symptom Screening in Pediatrics Tool (SSPedi) | Paediatric cancer 8-18 years | Please tell us how much each of these things bothered you yesterday or today by ticking the circle that best describes the amount it bothered you: | 15/15 (100%) + 1 optional open-ended | 5-point Likert (bother): Not at all bothered, A little, Medium, A lot, Extremely bothered | Yesterday or today | 1.8 (range 0.5 - 10.1) | CE + 1 optional OE | SA | Bother | Bother |
| Mini Symptom Screening in Pediatrics Tool (mini-SSPedi) | Paediatric cancer 4-7 years | Please tell us how much each of these things bothered you today by ticking the circle that best describes the amount it bothered you: | 15/15 (100%) + 1 optional open-ended | 3-point Likert (pictorial) (bother): Not bothered et all, Medium, Extremely Bothered | Today | NR | CE + 1 optional OE | SA, IA | Bother, distress | Bother |
| Peyronie's Disease Questionnaire (PDQ) | Peyronie's Disease | Men with Peyronie’s Disease (Penile Curvature) may have problems during vaginal intercourse. The questions below ask about the severity of any problems that you may be having during vaginal intercourse. Completing this questionnaire will help us best evaluate your condition and offer the best and most effective treatment of your Peyronie’s Disease (Penile Curvature).  1) Thinking about the LAST TIME you had an erection, how bothered were you by any pain or discomfort did you may have felt in your erect penis? Please answer for the LAST TIME YOU HAD AN ERECTION. E.g. 2) Thinking about the LAST TIME you looked at your erect penis, how bothered were you by the way your penis looked? Please answer for the LAST TIME YOU HAD AN ERECTION. | 2/15 (13.3%) | 5-point Likert (bother): Not at all bothered, A little bit bothered, Moderately bothered, Very bothered, Extremely bothered | NR (‘last erection’) | NR | CE | SA | Bother | Bother |
| Dry Eye related Quality of life Score (DEQS) | Dry eye disease | This questionnaire asks about how much you experience various eye symptoms, and also what kind of problems you experience in your daily life. Your answers will be used to inform future medical care. Please do not think too hard about the questions; just answer based on what you feel. For each question below, circle one response from 0-4 in Column A. If your answer is 0 ("Never") in Column A ➜ Move onto the next question. If your answer is 1-4 in Column A ➜ Also circle one from 1-4 in Column B. Please answer all questions without missing any.  During the past 7 days, did you experience the following symptoms? | 6/31 (19%) | 4-point Likert Type (bother): 1 Hardly bothered me, 2 Bothered me a little, 3 Bothered me, 4 Bothered me very much | 7 days | NR | CE | SA | Bother, burden | Bother |
| Menopause-Specific Quality-of-Life Questionnaire (MENQOL) | Menopause | For each of the following items, indicate whether you have experienced the problem in the past month. If you have, rate how much you have been bothered by the problem. | 29/58 (50%) | 7-point Likert (bother): 0 Not at all bothered, 1, 2, 3, 4, 5, 6 Extremely bothered | 1 month | 7 (range 5 - 15) | CE | SA | Bother | Bother |
| Pelvic Pain and Urgency/Frequency Patient Symptom Scale (PUF) | Interstitial cystitis, gynaecologic patients with pelvic pain | Please circle the answer that best describes how you feel for each question.  If you ___, does it bother you? Does ___ bother you? | 4/12 (33%) | 4-point Likert Type (bother):  2b: 0 Never bothers, 1 Occasionally, 2 Usually 3 Always 3b, 5b, 7b: 0 Never, 1 Occasionally, 2 Usually, 3 Always | NR | 5 | CE | SA | Trouble | Avoid, bother |
| Underactive Thyroid Symptom Rating Questionnaire (THYSRQ) | Hypothyroidism | This questionnaire asks you about symptoms that can be associated with underactive thyroid. You might have experienced some of these symptoms in recent weeks. Each question has two parts: for part (a) put an “X” in the box to indicate if you have had the symptom in recent weeks, regardless of the cause; for part (b) put an “X” in the box to indicate how much the symptom has bothered you. ONLY answer part (b) if you answered “yes” to part (a).  If yes, how much has this bothered you? | 15/30 (50%) | 4-point Likert Type (bother): Not at all, A little, Moderately, A lot | Recent weeks | NR | CE | SA | Bother, severity, trouble | Bother |
| Generalized Anxiety Disorder scale-7 (GAD-7) | Generalised anxiety disorders | Over the last 2 weeks, how often have you been bothered by the following problems | 7/7 (100%) | 4-point Likert Type (bother): 0 Not at all, 1 Several days, 2 More than half the days, 3 Nearly every day | 2 weeks | NR | CE | SA, IA | Bother, severity | Bother |
| Generalized Anxiety Disorder scale-2 (GAD-2) | Generalised anxiety disorders | Over the last 2 weeks, how often have you been bothered by the following problems | 2/2 (100%) | 4-point Likert Type (bother): 0 Not at all, 1 Several days, 2 More than half the days, 3 Nearly every day | 2 weeks | NR | CE | SA | Severity | Bother |
| Skindex-16 | Skin diseases | During the past week, how often have you been bothered by: | 4/16 (25%) | 7-point numerical scale (bother): 0 Never bothered, 1, 2, 3, 4, 5, 6 Always Bothered | 1 week | NR | CE | SA | Bother | Bother |
| Atrial Fibrillation Effect on QualiTy-of-life Questionnaire (AFEQT) | Atrial fibrillation | The following questions refer to how atrial fibrillation affects your quality of life. On a scale of 1 to 7, over the past 4 weeks, as a result of your atrial fibrillation, how much were you bothered by:  Please circle one number which best describes your situation | 4/21 (19%) | 7-point Likert (bother): 1 Not at all bothered Or I did not have this symptom, 2 Hardly bothered, 3 A little bothered, 4 Moderately bothered, 5 Quite a bit bothered, 6 Very bothered, 7 Extremely bothered | 4 weeks | 5 | CE | SA | Burden | Bother |
| Patient Health Questionnaire 15 (PHQ-15) | Somatoform disorders | During the past 4 weeks, how much have you been bothered by any of the following problems? | 15/15 (100%) | 3-point Likert (bother): 0 not bothered at all, 1 bothered a little, 2 bothered a lot | 4 weeks | NR | CE | SA | Severity | Bother |
| Urogenital Distress Inventory – Short Form (UDI-6 or UDI-SF) | Women with urinary incontinence | Please answer each question by checking the best response. While answering these questions, please consider your symptoms over the last 3 months. We realize that you may not be having problems in some of these areas, but please fill out this form as completely as possible.  During the past 4 weeks, how bothered were you by… | 6/6 (100%) | 4-point Likert Type (bother): 0 Not at all, 1 A little bit, 2 Moderately, 3 Greatly | 3 months | NR | CE | SA | Bother, distress | Bother, distress |
| HIV Symptom Index (HIV-SI or Symptom Distress Module (SDM) or ADSM) | Human immunodeficiency virus | Please answer the following questions by placing a (tick) in the appropriate box. The following questions ask about symptoms you might have had during the past four weeks. Please check the box that describes how much you have been bothered by each symptom.  I have this symptom and… | 20/20 (100%) | 5-point Likert (bother): 0 I do not have this symptom, 1 I have this symptom and it doesn't bother me, 2 I have this symptom and it bothers me a little, 3 I have this symptom and it bothers me, 4 I have this symptom and it bothers me a lot | 4 weeks | 5 | CE | SA | Bother | Bother, distress |
| Beck Anxiety Inventory (BAI) | Anxiety | Below is a list of common symptoms of anxiety. Please carefully read each item in the list. Indicate how much you have been bothered by that symptom during the past month, including today, by circling the number in the corresponding space in the column next to each symptom. | 21/21 (100%) | 4-point Likert Type (bother): 0) Not at all, 1) Mildly, but it didn't bother me much, 2) Moderately - it wasn't pleasant at times, 3) Severely - it bothered me a lot | 1 month | 5-10 | CE | SA | / | Bother |
| Gastrointestinal Symptom Rating Scale – IBS (GSRS-IBS) | Irritable Bowel Syndrome | Have you been bothered by (SYMPTOM) during the past week? | 13/13 (100%) | 7-point Likert (bother): 1 No discomfort at all, 2 Minor discomfort, 3 Mild discomfort, 4 Moderate discomfort, 5 Moderately severe discomfort, 6 Severe discomfort, 7 Very severe discomfort | 1 week | 3-5 | CE | SA | Bother | Bother |
| International Consultation on Incontinence Questionnaire Female Lower Urinary Tract Symptoms (ICIQ-FLUTS) | Women with lower urinary tract symptoms including urinary incontinence | Many people experience urinary symptoms some of the time. We are trying to find out how many people experience urinary symptoms, and how much they bother them. We would be grateful if you could answer the following questions, thinking about how you have been, on average, over the PAST FOUR WEEKS.  How much does this bother you? Please ring a number between 0 (not at all) and 10 (a great deal) | 12/24 (50%) | 11-point numerical rating scale (bother): 0 not at all 1 2 3 4 5 6 7 8 9 10 a great deal | 4 weeks | 4-5 | CE | SA | Bother | Bother |
| International Consultation on Incontinence Questionnaire Overactive Bladder (ICIQ-OAB) | Overactive bladder | Many people experience urinary symptoms some of the time. We are trying to find out how many people experience urinary symptoms, and how much they bother them. We would be grateful if you could answer the following questions, thinking about how you have been, on average, over the PAST FOUR WEEKS.  How much does this bother you? Please ring a number between 0 (not at all) and 10 (a great deal) | 4/10 (10%) | 11-point numerical rating scale (bother): 0 not at all 1 2 3 4 5 6 7 8 9 10 a great deal | 4 weeks | 4-5 | CE | SA | Bother | Bother |
| International Consultation on Incontinence Questionnaire Vaginal Symptoms (ICIQ-VS) | Vaginal symptoms | Many people experience vaginal symptoms some of the time. We are trying to find out how many people experience vaginal symptoms, and how much they bother them. We would be grateful if you could answer the following questions, thinking about how you have been, on average, over the PAST FOUR WEEKS.  How much does this bother you? Please ring a number between 0 (not at all) and 10 (a great deal) | 9/25 (36%) | 11-point numerical rating scale (bother): 0 not at all 1 2 3 4 5 6 7 8 9 10 a great deal | 4 weeks | 4-5 | CE | SA | Bother, problem | Bother |
| International Consultation on Incontinence Questionnaire Male Lower Urinary Tract Symptoms (ICIQ-MLUTS) | Males with symptoms suggestive of benign prostatic hyperplasia | Many people experience urinary symptoms some of the time. We are trying to find out how many people experience urinary symptoms, and how much they bother them. We would be grateful if you could answer the following questions, thinking about how you have been, on average, over the PAST FOUR WEEKS.  How much does this bother you? Please ring a number between 0 (not at all) and 10 (a great deal) | 13/26 (50%) | 11-point numerical rating scale (bother): 0 not at all 1 2 3 4 5 6 7 8 9 10 a great deal | 4 weeks | 4-5 | CE | SA | Bother, problem | Bother |
| Patient Health Questionnaire-12 Somatic Symptom scale (PHQ-12 SS) | Irritable bowel syndrome and symptomatic diverticular disease | During the past 4 weeks, how much have you been bothered by any of the following problems? | 12/12 (100%) | 3-point Likert (bother): 0 Not bothered at all, 1 Bothered a little, 2 Bothered a lot | 4 weeks | NR | CE | SA | Bother, trouble | Bother |
| Epidemiology of Prolapse and Incontinence Questionnaire (EPIQ) | Pelvic floor disorders | 1) These next questions are about problems some women have with their bowel and bladder control and female organs. Please answer these questions about your CURRENTPROBLEMS, not those that you may have had in the past that resolved through treatment or on their own. If you do not know the exact answer to any of these questions, please estimate the answer. We understand that your habits change with activity, location (work vs. home), how much you drink, medications, etc., so please base your answers on an average day. Some of the questions below use a scale to estimate how much the problems bother you. Here is an example of how to answer this type of question. (example shown) Remember: Answer these questions based on your CURRENT SYMPTOMS. 2) Some women will experience loss of support that can cause heaviness, pressure or a bulge around their vagina. This is called ‘‘pelvic prolapse’ ’You may have heard it called ‘‘cystocele’’, ‘‘rectocele’’ or ‘‘dropped bladder’’. Please answer each of the following questions about pelvic prolapse to the best of your ability. We realize that you may have good days and bad days. Please answer these questions based on a typical day. 3) Some women will experience problems with their bowels including difficulty passing stools, or involuntary loss of solid stool, liquid stool, or gas from the rectum. Please answer each of the following questions about these problems to the best of your ability.  How much are you bothered by (SYMPTOM). Please place an "I" on the line | 15/130 (12%) | 10cm VAS (bother): 0 Not at all, 10 Greatly | Current, typical day | 20+/- 6 | CE | SA | Bother | Bother |
| Overactive Bladder-V8 (OAB-V8) | Overactive bladder | The questions below ask about how bothered you may be by some bladder symptoms. Some people are bothered by bladder symptoms and may not realize that there are treatments available for their symptoms. Please circle the number that best describes how much you have been bothered by each symptom. Add the numbers together for a total score and record the score in the box provided at the bottom.  How bothered have you been by… | 8/8 (100%) | 6-point Likert Type (bother): 0 Not at all, 1 A little bit, 2 Somewhat, 3 Quite a bit, 4 A great deal, 5 A very great deal | NR | NR | CE | SA | Bother | Bother |
| Overactive Bladder - Bladder Assessment Tool (OAB-BAT) | Overactive bladder | Patient instructions: Questions 1 – 12 asks about your daily experience with bladder problems over the past 7 days. Please check ONE box for each question. There are no right or wrong answers. (For Questions 13-15) Patient Instructions: This questionnaire asks about your satisfaction with your bladder treatment over the past 7 days. Please check ONE box for each question. There are no right or wrong answers.  How bothered were you by ___? | 4/15 (27%) | 5-point verbal descriptor scale (bother):  Q5: 0 Not at all bothered, 1 A little bothered, 2 Moderately bothered, 3 Very bothered, 3 Extremely bothered  6-point verbal descriptor scale (bother): Q6: 0 I did not get up from sleeping to urinate, 0 Not at all bothered, 1 A little bothered, 2 Moderately bothered, 3 Very bothered, 3 Extremely bothered  Q7: 0 I did not have an urgent need to urinate, 0 Not at all bothered, 1 A little bothered, 2 Moderately bothered, 3 Very bothered, 3 Extremely bothered  Q8: I did not leak urine, 0 Not at all bothered, 1 A little bothered, 2 Moderately bothered, 3 Very bothered, 3 Extremely bothered | 7 days | NR | CE | SA | Bother | Bother |
| Rotterdam Symptom Check List (RSCL) | Cancer patients | In this questionnaire you will be asked about your symptoms. Would you please, for all symptoms mentioned, indicate to what extent you have been bothered by it, by circling the answer most applicable to you. The questions are related to the past week.  Have you, during the past week, been bothered by: | 30/39 (77%) | 4-point Likert Type (bother): 1 not at all, 2 a little, 3 quite a bit, 4 very much | 1 week | 8 | CE | SA, IA, PA | Bother, distress | Bother |
| Benign Prostatic Hyperplasia Health-related Quality of Life Survey (BPHQoL) | Benign prostatic hyperplasia | Below is a list of symptoms associated with urination you may or may not have experienced:  On a 0-to-6-point scale, please rate how bothersome the following symptoms have been during the PAST MONTH: (Please mark an "X" in the box). During the PAST MONTH, have you been bothered by nervousness or your "nerves"? (Please mark an "X" in the box). | 13/49 (27%) | 6-point Likert Type (bother): Q33: 1 Extremely so - to the point where I could not work or take care of things; 2 Very much so; 3 Quite a bit; 4 Some - enough to bother me; 5 A little; 6 Not at all  Q33: 1 Extremely so - to the point where I could not work or take care of things, 2 Very much so, 3 Quite a bit, 4 Some - enough to bother me, 5 A little, 6 Not at all  7-point Likert (bother): Q13-24: 0 I do not have the symptoms; 1 & 2 Not Bothersome; 3 & 4 Somewhat bothersome; 5 & 6 Extremely bothersome | 1 month | NR | CE | SA | Bother | Bother |
| Seizure Severity Questionnaire (SSQ) | Seizure | Please describe the MOST COMMON TYPE OF SEIZURE that you have had in the past 4 weeks. Consider only this type of seizure when answering questions. When answering the following questions, please mark or circle the number that best describes your feelings about each topic. For example, "Somewhat helpful", "Same", "Moderate"  How BOTHERSOME (interfere with your life) were the (SYMPTOM)? | 5/23 (21%) | 7-point numerical scale (bother):  1 no bother at all, 2, 3, 4 moderate, 5, 6, 7 very bothersome | 4 weeks | NR | CE | IA | Bother | Bother, interfere |
| Hypoglycaemia Symptom Rating Questionnaire (HypoSRQ) | Hypoglycaemia | This questionnaire is for people who are having treatment for diabetes. It asks about symptoms that can be associated with hypoglycaemia (low blood sugar levels) often known as hypos. You might have experienced some of these symptoms in recent weeks (i.e. about 4 weeks). Each question has two parts: for part (a) put an “X” in the box to indicate if you have had the symptom in recent weeks, regardless of the cause; for part (b) put an “X” in the box to indicate how much the symptom has bothered you. ONLY answer part (b) if you answered “yes” to part (a).  If yes, how much has this bothered you? | 18/36 (50%) | 4-point Likert Type (bother): Not at all, a little, moderately, a lot | Recent weeks (about 4 weeks) | 5 | CE | SA | Bother | Bother |
| Heart Failure Somatic Perception Scale (HFSPS) | Heart failure | Please read each statement. Check the number that closely measures how you felt in the past week. Please respond to each statement. | 18/18 (100%) | 6-point Likert Type (bother): 0 I did not have this symptom, 1 Not at all bothersome, 2 Slightly bothersome, 3 Moderately bothersome, 4 Quite a bit bothersome, 5 Extremely bothersome | 1 week | NR | CE | SA | Burden | Bother |
| Pelvic Floor Distress Inventory-46 (PFDI-46) | Female pelvic floor disorders | Please answer all of the questions in the following survey. The questions will ask you if you have certain bowel, bladder or pelvic symptoms and if you do how much they bother you. Answer these questions by putting an X in the appropriate box or boxes. If you are unsure about how to answer a question, give the best answer you can. While answering these questions please consider your symptoms of the last 3 months.  If yes, how much does this bother you? | 46/46 (100%) | 4-point Likert Type (bother): 1 Not at all, 2 Somewhat, 3 Moderately, 4 Quite a bit | 3 months | NR | CE | SA | Bother, distress | Bother, distress |
| Pelvic Floor Distress Inventory - Short Form 20 (PFDI-20) | Women with disorders of the pelvic floor including urinary incontinence, pelvic organ prolapse, and faecal incontinence | Please answer all of the questions in the following survey. These questions will ask you if you have certain bowel, bladder or pelvic symptoms and if you do how much they bother you. Answer each question by putting an X in the appropriate box or boxes. If you are unsure about how to answer, please give the best answer you can. While answering these questions, please consider your symptoms over the last 3 months.  If yes, how much does it bother you? | 20/40 (50%) | 4-point Likert Type (bother): Not at all, Somewhat, Moderately, Quite a bit | 3 months | NR | CE | SA | Bother, distress | Bother, distress |
| BDS Checklist | Bodily distress syndrome (BDS) | During the last 4 weeks, have you been bothered by… | 25/25 (100%) | 5-point Likert (bother): Not at all, A bit, Somewhat, Quite a bit, A lot | 4 weeks | NR | CE | SA | Bother | Bother |
| PRO-Kid | Paediatric chronic kidney disease | Please tell us how often you had each of these feelings and how much they bothered you in the past week by clicking the bubble that best describes the amount. Please rate the following items for frequency and impact:  IMPACT: In the past week, how much did this symptom bother you? | 14/28 (50%) | 5-point Likert (bother): Not at all, A little, Medium, A lot, Extremely bothered | 1 week | 5 | CE | SA | Bother, impact | Bother, impact |
| New Mexico Refugee Symptom Checklist-121 (NMRSCL-121) | Refugees | Using the scale beside each symptom, please indicate the degree to which the symptom has been bothersome to you over the past year. If the symptom has not been bothersome to you during the past year, circle "NOT AT ALL.” | 121/121 (100%) | 5-point Likert (bother): 0 Not at all, 1 A little bit, 2 Moderately, 3 Quite a bit, 4 Extremely | 1 year | NR | CE | SA | Bother, distress | Bother |
| Bladder Control Self-Assessment Questionnaire (B-SAQ) | Lower urinary tract symptoms | Please put the NUMBER that applies to you in the boxes shown by the arrows based on the following: Not at all = 0, A little = 1, Moderately = 3, A great deal = 3  How much does it bother you? | 4/8 (50%) | 4-point Likert Type (bother): 0 Not at all, 1 A little, 2 Moderately, 3 A great deal | NR | Less than 5 | CE | SA | Bother | Bother |
| Children's Somatic Symptoms Inventory (formally Children's Somatization Inventory) (CSSI or CSI) | Somatic symptoms in those aged 8 - 18 years | Below is a list of symptoms that children and teens sometimes have. Please circle a number telling how much you were bothered by each symptom during the past two weeks.  In the past 2 weeks, how much were you bothered by each symptom? | 24/24 (100%) | 5-point Likert (bother): 0 Not at all, 1 A little, 2 Some, 3 A lot, 4 A whole lot | 2 weeks | NR | CE | SA  In cases of younger age and low reading ability the PROM can be IA | Severity | Bother |
| Somatic Symptom Scale-8 (SSS-8) | Somatic symptoms in mental disorders | During the past 7 days, how much have you been bothered by any of the following problems? | 8/8 (100%) | 5-point Likert (bother): 0 Not at all, 1 A little bit, 2 Somewhat, 3 Quite a bit, 4 Very much | 7 days | 1 | CE | SA | Burden | Bother |
| Greene Climacteric Scale | Menopause | Please indicate the extent to which you are bothered at the moment by any of these symptoms by placing a tick in the appropriate box: | 21/21 (100%) | 4-point Likert Type (bother): 0 Not at all, 1 A little, 2 Quite a bit, 3 Extremely | At the moment | NR | CE | SA | Bother | Bother |
| Psoriasis Symptom Diary (PSD) † | Chronic plaque psoriasis | Overall how bothered were you by ___ over the past 24 h? | 6/20 (30%) | 11-point NRS with labelled anchors (bother): 0 Not bothered at all, 10 Bothered as bad as you can imagine | 24 hours | NR | CE | SA | Bother | Bother |
| PTSD Checklist - Civilian Version (PCL-C) | Post-traumatic stress disorder | Below is a list of problems and complaints that veterans sometimes have in response to stressful life experiences. Please read each one carefully, put an “X” in the box to indicate how much you have been bothered by that problem in the last month. | 17/17 (100%) | 5-point Likert (bother): 1 Not at all, 2 A little bit, 3 Moderately, 4 Quite a bit, 5 Extremely | 1 month | 5-10 | CE | SA | Bother, severity | Bother |
| Kidney Disease and Quality of Life Short Form (KDQOL-SF) | Kidney disease and undergoing dialysis | This survey includes a wide variety of questions about your health and your life. We are interested in how you feel about each of these issues.  During the past 4 weeks, to what extent were you bothered by each of the following? | 13/82 (16%) | 5-point Likert (bother): 1 Not at all bothered, 2 Somewhat bothered, 3 Moderately bothered, 4 Very much bothered, 5 Extremely bothered | 4 weeks | 16 | CE | SA  Can be IA if needed | Bother | Bother |
| Danish Prostatic Symptom Score (DAN-PSS-1) ‡ | Benign prostatic hyperplasia | The questionnaire is made up of two kinds of questions: question A asks about the intensity of a symptom and question B asks about how much you are bothered by the symptom. For each of the 15 questions, please indicate how intense and how bothersome the symptom is by ticking the box which best describes your situation. If you tick "0" for question A, please do not answer question B. When completing the questionnaire, please refer to the last 2 weeks.  … how bothersome is this for you? | Everyone: 12/24 (50%)  Those who are sexually active: 15/30 (50%) | 4-point Likert type (bother): 0 Not at all, 1 A little bit, 2 Moderately, 3 Very much | 2 weeks – Part A  1 month – Sexually active items | NR | CE | SA | Bother, trouble | Bother |
| Kidney Disease and Quality of Life Instrument - 36 items (KDQOL-36) | Kidney disease | This survey includes a wide variety of questions about your health and your life. We are interested in how you feel about each of these issues.  During the past 4 weeks, to what extent were you bothered by each of the following? | 13/36 (36%) | 5-point Likert (bother): 1 Not at all bothered, 2 Somewhat bothered, 3 Moderately bothered, 4 Very much bothered, 5 Extremely bothered | 4 weeks | NR | CE | SA | Bother | Bother |
| Cervical Dystonia Impact Profile (CDIP-58) | Cervical dystonia (spasmodic torticollis) | We are interested in finding out how spasmodic torticollis (also known as cervical dystonia) impacts on your day to day life. We would be grateful if you could help us by filling out this questionnaire. All of the information you provide is COMPLETELY CONFIDENTIAL.PLEASE NOTE: All questions are about the impact of spasmodic torticollis. People like you who have spasmodic torticollis are bothered by different problems. The following questions ask about problems you may have been bothered by during the past 2 weeks.  During the past 2 weeks, how much were you bothered by each of the following problems? (Please circle the number in the box that best describes your situation). | 11/58 (19%) | 5-point Likert (bother): 1 Not at all, 2 A little, 3 Moderately, 4 Quite a bit, 5 Extremely | 2 weeks | NR | CE | SA | Impact | Bother, impact |
| Sciatica Bothersomeness Index (SBI) | Sciatica | / | 4/4 (100%) | 7-point Likert (bother): 0 Not Bothersome, 1, 2, 3 Somewhat Bothersome, 4, 5, 6 Extremely Bothersome | NR | NR | CE | SA | Bother | Bother |
| OAB-q (1-week-recall) | Adult overactive bladder | This questionnaire asks about how much you have been bothered by selected bladder symptoms during the past 4 weeks. Please place a (tick) or (cross) in the box that best describes the extent to which you were bothered by each symptom during the past 4 weeks. There are no right or wrong answers. Please be sure to answer every question.  During the past 4 weeks, how bothered were you by… | 8/33 (24%) | 6-point Likert Type (bother): 1 Not at all, 2 A little bit, 3 Some-what, 4 Quite a bit, 5 A great deal, 6 A very great deal | 1 week | 5-10 | CE | SA | Bother, distress | Bother |
| Urinary Tract Infection Symptom Assessment (UTISA) | Uncomplication urinary tract infection (uUTI) | Baseline: If you have experienced these symptoms/problems in the past 24 hours, please indicate how bothersome they were? (Please circle one number for each symptom). Follow up: Since you last completed this questionnaire, if you have experienced these symptoms/problems, please indicate how bothersome they were? (Please circle one number for each symptom) | Baseline: 7/15 (47%)  Follow up: 7/17 (41%) | 4-point Likert Type (bother): 0 Not at all, 1 A little, 2 Moderately, 3 A lot | 24 hours – Baseline  ‘Since last visit’ – Follow up | NR | CE | SA | Bother, trouble | Bother |
| OverActive Bladder Awareness Tool - 3-item (OAB-V3) | Overactive bladder | The questions below ask about how bothered you may be by some bladder symptoms. Some people are bothered by bladder symptoms and may not realise that there are treatments available for their symptoms. Please circle that number that best describes how much you have been bothered by each symptom. Add the numbers together for a total score and record the score in the box provided at the bottom.  How bothered have you been by… | 3/3 (100%) | 6-point Likert Type (bother): 0 Not at all, 1 A little bit, 2 Somewhat, 3 Quite a bit, 4 A great deal, 5 A very great deal | NR | NR | CE | SA | Bother | Bother |
| Weight-Related Symptom Measure (WRSM) | Weight related symptoms in those who are overweight or obese | For each of the following questions, read the list of symptoms below, and mark an ý in the one box that best describes your answer.  If Yes, how much did these symptoms bother you? | 20/40 (50%) | 7-point Likert (bother): 0 Not at all, 1 Hardly, 2 Somewhat, 3 Moderately, 4 A good deal, 5 A great deal, 6 A very great deal | 4 weeks | 2 | CE | SA | Bother, impact | Bother |
| Patient Perspective of Arrhythmia Questionnaire (PPAQ) | Supraventricular tachycardia (SVT) | We are conducting a research study trying to learn more about symptoms and quality of life in patients like you who have an irregular heart rhythm. Quality of life is a term which is used to describe one’s daily life and overall health, including physical emotional and social activities. We are also interested in how these activities are affected by having an irregular heart rhythm or by the treatment for the irregular heart rhythm. These questions will ask you information about yourself and your health, as well as which symptoms you may have experienced and how bothersome you found these symptoms to be. All answers will be confidential and may benefit future patients, so please try to answer honestly and completely. Please answer the question by thinking back over the last 4 weeks. You may fill out the questionnaire with either a pen or a pencil. Answer the questions by filling in the circle like this or by making an X or √ in the circle. If you make a mistake, please erase your answer completely or place a box around the right answer like this. Please complete the questionnaire to the best of your ability without help from others. Some questions may look alike, but each one is different. There are NO right or wrong answers. If you are unsure about how to answer a question, please give the best answer you can and make a comment at the end of the questionnaire. Please return the questionnaire booklet back to us within 2 weeks. A self-addressed envelope has been enclosed for your convenience. Thank you for taking part in this study!  (a) Have you had any of these symptoms with your fast heart rhythm in the last 4 weeks? (Check “yes” or “no” on each line) (b) If present, how much did it bother you? (Check appropriate box on each line) | 18/50 (36%) + 1 optional open-ended | 5-point Likert (bother): Not at all, A little bit, Moderately bothersome, Quite a bit, Extremely bothersome | 4 weeks | 20 (15-30) | CE + 1 optional OE | SA | Bother, distress | Bother |
| Diabetes Quality of Life Clinical Trial Questionnaire (DQLCTQ) | Diabetes (type 1 and 2) | Below is a list of symptoms commonly associated with diabetes. Please indicate how often you have experienced each symptom in the past week AND how bothered you have been by each symptom.  How bothered have you been by these problems in the past week? (Circle one number) | 7/144 (5%) | 5-point Likert (bother): 1 VERY BOTHERED, 2, 3, 4, 5 NOT AT ALL BOTHERED | 1 week | 10 | CE | SA | / | Bother |
| Paediatric Rhinoconjunctivitis Quality of Life Questionnaire (PRQLQ) | Children 6-12 years with allergic rhinoconjunctivitis | I want you to tell me how much you have been bothered by your nose and eye allergies during the past week. I will tell you which card to use. Pick the number that best describes how much you were bothered by your allergies during the past week.  How much were you bothered by … during the past week? | 11/23 (48%) | 7-point Likert (bother): 0 Not bothered, 1 Hardly bothered at all, 2 Bothered a lot, 3 Somewhat bothered, 4 Quite bothered, 5 Very bothered, 6 Extremely bothered | 1 week | NR | CE | IA (parents not to be present) | Bother, trouble | Bother |
| Paediatric Asthma Quality of Life Questionnaire (PAQLQ) | 7–17-year-olds with asthma | How much did … bother you during the past week? | 5/60 (12%) | 7-point Likert (bother): 1 Extremely bothered, 2 Very bothered, 3 Quite bothered, 4 Somewhat bothered, 5 Bothered a bit, 5 Hardly bothered at all, 7 Not bothered | 1 week | Initial consult: 10-15  Follow-up occasions: 5-10 | CE | IA | Bother, trouble | Bother |
| Quality of Life Questionnaire Onychomycosis (Nail fungal condition) (OnyCOE-t) | Toenail onychomycosis | Instructions: The purpose of this questionnaire is to find out about your health in general and about how your onychomycosis (nail fungal condition) affects your quality of life. Please read each question carefully before answering. If you are unsure about how to answer a question, please give the best answer you can. Remember, there are no right or wrong answers. The information you give will be combined with the responses of other patients completing this questionnaire, and you will not be identifiable as an individual in research projects. 1) Please answer the following questions regarding problems you may have had in the PAST 4 WEEKS with your nail fungal condition. Answer each question completely by indicating BOTH: I - HOW MUCH OF THE TIME you experienced the problem, and then II - HOW BOTHERED you were by the problem. (If you are unsure about how to answer a question, give the best answer you can.)  In the past four weeks, when you had this problem, how bothered were you? (Circle one number on each line) | 6/38 16%) + 2 optional open-ended | 5-point Likert (bother): 1 Not at all bothered, 2 Slightly bothered, 3 Moderately bothered, 4 Very bothered, 5 Extremely bothered | 4 weeks | NR | CE + 1 optional OE | SA | Bother | Bother |
| Lee Chronic Graft-versus-Host Disease Symptom Scale (cGVHD Symptom Scale) | Chronic graft-versus-host disease (cGVHD) | By circling one (1) number per line, please indicate how much you have been bothered by the following problems in the past month: | 30/30 (100%) | 5-point Likert (bother): 0 Not at all, 1 Slightly, 2 Moderately, 3 Quite a bit, 4 Extremely | 1 month | 5 | CE | SA | Bother, burden | Bother |
| The Modified 7-Day Lee Chronic Graft-versus-Host Disease Symptom Scale (Modified cGVHD Symptom Scale) | Chronic graft-versus-host disease (cGVHD) | By circling one (1) number per line, please indicate how much you have been bothered by the following problems in the past 7 days: | 28/28 (100%) | 5-point Likert (bother): 0 Not at all, 1 Slightly, 2 Moderately, 3 Quite a bit, 4 Extremely | 7 days | NR | CE | SA | Bother | Bother |
| Chronic Otitis Media Questionnaire 12 (COMQ -12) | Active chronic otitis media | These questions are to find out how badly your ear problems affect you. No machine can do this: only you can tell us. We expect the results from this questionnaire to help us understand which of your ear symptoms is the most important to you. Knowing this will help us improve the ways patients with ear problems are looked after. Please answer the questions below by considering carefully each question asked and then ringing the appropriate number; the numbers each refer to a particular description. There are no right or wrong answers, but please try to think carefully about each question before ringing the appropriate number. Please consider each problem as it has been over the past 6 months.  For the following questions, please indicate how severe the various elements described affect you, using the scale below and by ringing the appropriate number: 0 Doesn’t bother me at all 1 A minor inconvenience 2 A moderate inconvenience 3 A major inconvenience but I can cope 4 A major inconvenience and I am finding it hard to cope 5 The worst thing that has ever affected my life | 7/12 (58%) | 6-point NRS (bother): 0 Doesn’t bother me at all 1 A minor inconvenience 2 A moderate inconvenience 3 A major inconvenience but I can cope 4 A major inconvenience and I am finding it hard to cope 5 The worst thing that has ever affected my life | 6 months | NR | CE | SA | / | Bother, severity, inconvenience, affect |
| Gastrointestinal Symptom Rating Scale - self-administered version (GSRS-self) | Upper gastrointestinal diseases | Please read this first: This survey contains questions about how you have been feeling and what it has been like DURING THE PAST WEEK. Mark the choice that best applies to you and your situation with an “X” in the box.  Have you been bothered by (SYMPTOM) during the past week? | 15/15 (100%) | 7-point Likert (bother): No discomfort at all, minor discomfort, mild discomfort, moderate discomfort, moderately severe discomfort, severe discomfort, very severe discomfort | 1 week | 3-5 | CE | SA | / | Bother, discomfort |
| Postpartum Symptom Inventory (PSI) | Postpartum somatic symptoms | In the last 7 days, how often have you been bothered by the following: (Please mark one answer for each symptom): | 20/20 (100%) | 5-point Likert (bother): Never, Rarely, Sometimes, Often, Always | 7 days | NR | CE | SA | Bother | Bother |
| General Symptom Questionnaire-30 (GSQ-30) | Acute and post-treatment Lyme disease syndrome | Rate "bother" for the past 2 weeks.  During the past 2 weeks, how much have you been bothered by any of the following? | 30/32 (94%) | 5-point Likert (bother): 0 Not at all, 1 A little bit, 2 Somewhat, 3 Quite a bit, 4 Very much | 2 weeks | NR | CE | SA | Bother, burden, impact | Bother, impairment |
| Male Sexual Health Questionnaire (MSHQ) | Aging men with urogenital health concerns | INTRODUCTION: The following questions concern various aspects of your ability to have sex. In answering these questions, please think about all aspects of the sexual activity you have had with your main partner, with other partners, or masturbating. By sexual activity, we mean any type of sex you may have had, including intercourse, oral sex or other sexual activities that could lead to ejaculation. Some of these questions might be difficult to answer. Please answer as many as possible and be as honest as you can when answering them. Please remember that all of your answers are confidential.    4) In the last month, if you have had difficulty getting hard or staying hard without using drugs like Viagra, have you been bothered by this problem?... (Check only one) 12) In the last month, if you have had any ejaculation difficulties or have been unable to ejaculate, have you been bothered by this? 24) in the last month, have you been bothered by your level of sexual desire? | 3/27 (11%) + 2 optional items if applicable | 5-point Likert (bother): Q4: 5 Not at all bothered/Did not have a problem with erection, 4 A little bit bothered, 3 Moderately bothered, 2 Very bothered, 1 Extremely bothered  Q 12 and 24: 5 Not at all bothered, 4 A little bit bothered, 3 Moderately bothered, 2 Very bothered, 1 Extremely bothered | 1 month | 10-15 | CE | SA | Bother | Bother |
| Quality of Vision Questionnaire (QOV) | Patients with and without refractive correction in the form of spectacles, contact lenses, laser refractive surgery, and intraocular refractive surgery with various types of intraocular lenses (IOLs) and patients who have eye diseases that cause QoV problems, such as cataract. | Instructions: Look at the pictures on the accompanying page and familiarise yourself with the meaning of each symptom. Now answer the questions below about your eyesight in your everyday life. If you have had surgery, please respond based on how you are now, not prior to surgery. Please respond based on how you have felt in the past week. Please circle your answer  How bothersome is …? | 10/30 (33%) | 4-point Likert Type (bother): Not at all, A little, Quite, Very | 1 week  Current (now) – For those who have had surgery within the past week | NR | CE | SA | Bother | Bother |
| Symptom checklist (SCL) | Chronic liver disease | This questionnaire captures symptoms that can occur in persons with liver disease. For each of these you are asked whether you have the symptom and how much you are bothered by it. For each symptom, mark one box, depending on whether you are not bothered by it at all (“none at all”) or either “a little bit”, “moderately”, “quite a bit”, or “extremely” bothered by it. If you do not have the symptom, you should mark “none at all”.  During the last month, how much have you been bothered by the following: | 10/10 (100%) | 5-point Likert scale (bother): None at all, A little bit, Moderately, Quite a bit, Extremely | 1 month | NR | CE | SA | / | Bother |
| Cutaneous neurofibromas Skindex (cNF-Skindex) | Cutaneous neurofibromas | During the past week, how often have you been bothered by: | 6/18 (33%) | 7-point Likert (bother): 0 Never bothered, 1, 2, 3, 4, 5, 6 Always bothered | 1 week | NR | CE | SA | Bother | Bother |
| MASK Allergy app (MASK-air) | Allergic rhinitis (hay fever) | How much are your ___ symptoms bothering you? | 4/10 (40%) | VAS with anchors (bother): Not at all bothersome --> Extremely bothersome | Today | NR | CE | SA | Bother | Bother |
| Psoriasis Skin Appearance Bothersomeness measure (PSAB) † | Plaque psoriasis | / | 3/3 (100%) | 11-point NRS (bother): 0 Not at all bothered, 1, 2, 3, 4, 5, 6, 7, 8, 9, 10 Extremely bothered | NR | NR | CE | SA | Bother | Bother |
| Cataract Symptom Score (CSS) † | Cataracts | Are you bothered by… (SYMPTOM)? | 5/5 (100%) | 4-point Likert Type (bother): 0 Do not have symptom or not bothered by it, 1 A little bothered, 2 Somewhat bothered, 3 Very bothered | Q1-4: NR  Q5: last month | NR | CE | SA | Bother | Bother |
| EndoWheel | Endometriosis | For each statement below, indicate your level of agreement by circling the score directly on the coloured EndoWheel on the following page.  *Statement wording is unique for each symptom* | 5/12 (42%) | 11-point NRS with anchors (bother): 0 (I do not agree at all), 1 2 3 4 5 6 7 8 9, 10 (I completely agree) | 3 months | 5-6 | CE | SA | Bother, burden | Bother |
| Symptom Checklist 90 – Revised (SCL-90-R) | Psychological problems and symptoms of psychopathology in people aged over 13 years | Below is a list of problems and complaints that people sometimes have. Please read each one carefully and enter the number that best describes how much you were bothered by that problem during the past week. Please enter only ONE.  FOR THE PAST WEEK, HOW MUCH WERE YOU BOTHERED BY: | 90/90 (100%) | 5-point Likert scale (bother): 0 Not at all, 1 A little bit, 2 Moderately, 3 Quite a bit, 4 Extremely | 1 week | 12-15 | CE | SA | Distress | Bother |
| Visual Analog Scale for QOL Questionnaire for Bother about a Symptom (OABSS-VAS) | Lower urinary tract symptoms | “How would you rate your bother? Please check (x) on the linear scale above in accordance with your satisfaction with each urinary symptom.” | 7/8 (88%) | 10cm VAS with 7 written descriptors and 5-face points (bother): Delighted, Pleased, Mostly satisfied, Mixed about equally satisfied and dissatisfied, Mostly dissatisfied, Unhappy, Terrible  Face scale: 1) laughing face to represent delight or pleased above left end of VAS, 2 ) smiling face to represent mostly satisfied above left side to centre of VAS, 3) face with neutral expression to represent neither satisfied and dissatisfied above centre of VAS, 4) face in trouble to present mostly dissatisfied above right side to centre of VAS, 5) crying face to represent unhappy or terrible above right end of VAS" | NR | NR | CE | SA | Bother | Bother, satisfaction |
| Simplified Personal Questionnaire (PQ) | Psychological difficulties | Please complete before each session. Rate each of the following problems according to how much it has bothered you during the past seven days, including today. | (100%). Number of items varies. Aim for between 1-10, space to list up to 12. | 7-point Likert scale (bother): 1 Not at all, 2 Verry little, 3 Little, 4 Moderately, 5 Considerably, 6 Very considerable, 7 Maximum possible | 7 days | Initial: 30 to generate items  Follow up: less than 1 | OE | IA | Bother | Bother |
| Impact of Dry Eye on Everyday Life (IDEEL) | Drye eye (non-Sjögren’s kerato conjunctivitis sicca (non-SSKCS) and Sjögren’s Syndrome (SS)) | The following questions ask about how bothersome dry eye symptoms were to you OVER THE LAST TWO WEEKS. If you had the symptom, please choose how much the symptom bothered you (not at all, slightly, moderately, or very much). If you did not have the symptom over the last two weeks, choose the “I did not have this symptom / Not applicable” box. Please choose only one box per question.  OVER THE LAST TWO WEEKS, how much did each of the following symptoms bother you? | 19/20 (95%) | 5-point Likert (bother): I did not have this symptom / Not applicable; OVER THE LAST TWO WEEKS, I had this symptom and it bothered me: Not at all, Slight, Moderately, Very much | 2 weeks | 30 | CE | SA | Bother | Bother |
| Menoscale | Menopause | To what extent have you been bothered by these as a menopause symptom in the past week? | 20/25 (80%) | 4-point NRS (bother): Not at all, A little, Quite a bit, Extremely | 1 week | NR | CE | SA | Bother, impact | Bother |
| Skindex-8AA | Alopecia areata | During the past 7 days, how often have you been bothered by: | 7/8 (88%) | 7-point Likert (bother): 0 (never bothered), 6 (always bothered) | 7 days | NR | CE | SA | Bother, impact | Bother |
| NIH PROMIS GI Symptoms Scales | Gastrointestinal symptoms | In the past 7 days, how much did ___ bother you? | 9/62 (15%) | 5-point Likert (bother): 1 Not at all, 2 A little bit, 3 Somewhat, 4 Quite a bit, 5 Very much | 7 days | NR | CE | SA | Bother, impact | Bother |
| Brisk walking- and running-induced lower Urinary tract symptoms questionnaire (BRUTS-Q) | Lower urinary tract symptoms | How bothersome do you find ___ while running/jogging or brisk walking? (Circle) | 3/122 (2%) | 11-point NRS (bother): 0 Not bothersome, 10 Very bothersome | NR | NR | CE | SA | Bother | Bother |
| International Consultation on Incontinence Questionnaire-Underactive Bladder (ICIQ-UAB) | Underactive bladder | How much did this bother you? Please circle a number between 0 (not at all) and 10 (a great deal) | 15/52 (29%) | 11-point NRS (bother): 0 Not at all, 10 A great deal | 24 hours | NR | CE | SA | Bother | Bother |
| International Trauma Questionnaire (ITQ) | Post-traumatic stress disorder (PTSD) and chronic post-traumatic stress disorder (CPTSD) in adults | Below are a number of problems that people sometimes report in response to traumatic or stressful life events. Please read each item carefully, then circle one of the numbers to the right to indicate how much you have been bothered by that problem in the past month. | 6/20 (30%) | 5-point Likert (bother): 0 Not at all, 1 A little bit, 2 Moderately, 3 Quite a bit, 4 Extremely | 1 month | N4 | CE | SA | NR | Bother |
| International Trauma Questionnaire – Child and Adolescent Version (ITQ-CA) | Post-traumatic stress disorder (PTSD) and chronic post-traumatic stress disorder (CPTSD) in children and adolescents | Below are problems people can have after an upsetting or a stressful event. Thinking about that event, circle 0, 1, 2, 3 or 4 for how much the following things have bothered you in the past month.  Below are problems people report after traumatic or stressful events. They are about how you feel, what you believe about yourselves and others. Circle 0, 1, 2, 3 or 4 for how much the following things have bothered you in the past month. | 12/22 (55%) | 5-point Likert (bother): 0 Never, 1 A little bit, 2 Sometimes, 3 A lot, 4 Almost always | 1 month | NR | CE | SA | Bother | Bother |
| International Consultation on Incontinence Questionnaire - Bowels (ICIQ-B) | Faecal incontinence | Many people experience bowel accidents or bowel leakages. We are trying to find out how many people experience these symptoms and how much this bothers them. We would be grateful if you could answer the following questions, thinking about how you have been over the PAST THREE MONTHS.  How much does this bother you? Please ring a number between 0 (not at all) and 10 (a great deal) | 13/45 (29%) | 11-point NRS (bother): 0 Not at all, 1, 2, 3, 4, 5, 6, 7, 8, 9, 10 A great deal | 3 months | NR | CE | SA | NR | Bother |
| PTSD Checklist-Military version (PCL-M) | Post-traumatic stress disorder (PTSD) | Below is a list of problems and complaints that veterans sometimes have in response to stressful military experiences. Please read each one carefully, then circle one of the numbers to the right to indicate how much you have been bothered by that problem in the past month. | 17/17 (100%) | 5-point Likert (bother): 1 Not at all, 2 A little bit, 3 Moderately, 4 Quite a bit, 5 Extremely | 1 month | NR | CE | SA | Bother | Bother |
| Posttraumatic Stress Disorder Checklist for DSM-5 (PCL-S) | Post-traumatic stress disorder (PTSD) | Below is a list of problems that people sometimes have in response to a very stressful experience. Keeping your worst event in mind, please read each problem carefully and then select one of the numbers to the right to indicate how much you have been bothered by that problem in the past month.  In the past month, how much were you bothered by: | 20/20 (100%) | 5-point Likert (bother): 0 Not at all, 1 A little bit, 2 Moderately, 3 Quite a bit, 4 Extremely | 1 month | NR | CE | SA | Bother, severity | Bother |
| Physical Symptoms Questionnaire (PSQ-51) | Generic (any known medical conditions, somatic symptom disorders, general population) | The following 51 physical symptoms occur frequently. Please indicate for each of them whether you have been bothered by any of them during the last 7 days.  You can choose one of the following answers:  0 = I was not bothered by this  1 = I was sometimes bothered by this  2 = I was regularly bothered by this  3 = I was often bothered by this  For each of the symptoms, indicate which answer applies to you. | 51/51 (100%) | 4-point Likert-Type (bother): 0 I was not bothered by this, 1 I was sometimes bothered by this, 2 I was regularly bothered by this, 3 I was often bothered by this | 7 days | NR | CE | SA | Bother, burden | Bother |

† Partial or incomplete PROM retrieved only able to be retrieved, therefore some missing data; ‡ DAN-PSSsex not extracted as makes up last three items in DAN-PSS-1 PROM; CE: closed-ended; IA: interviewer-administered; NR: not reported; NRS: Numerical rating scale; OE: open-ended; PA: proxy-administered; SA: self-administered; VAS: Visual analogue scale

**Table S8 Indirect + Assessed PROMs measuring the construct of ‘trouble’ (n = 13)**

| **PROM (abbreviation)** | **Population or health condition** | **Instructions and/or question wording** | **Number of ‘bother’ items / total items (%)** | **Scale type and wording** | **Recall period** | **Average time to complete (minutes)** | **Open- or closed-ended item list** | **Method of completion** | **‘Bother’ synonyms used in manuscript** | **‘Bother’ synonyms used in PROM** |
| --- | --- | --- | --- | --- | --- | --- | --- | --- | --- | --- |
| Nocturnal Rhinoconjunctivitis Quality of Life Questionnaire (NRQLQ) | Adults with nocturnal allergic rhinoconjunctivitis | Please complete all questions by circling the number that best describes how troubled you have been during the last week as a result of your nose/eye symptoms? | 16/16 (100%) | 7-point Likert (trouble): 0 Not troubled, 1 Hardly troubled at all, 2 Somewhat troubled, 3 Moderately troubled, 4 Quite a bit troubled, 5 Very troubled, 6 Extremely troubled | 1 week | 5 | CE | SA | Bother, impact, trouble | Trouble |
| Eye Allergy Patient Impact Questionnaire (EAPIQ) | Ocular allergy | The following questions refer to Seasonal Eye Allergy Symptoms you may have or have had. The time period in each question may be different, so please read each question carefully. Please answer each question by writing in a number or marking/circling the number which best describes your situation. Your answers, combined with answers from other allergy sufferers, will help us determine the impact of eye allergy symptoms. All answers will be kept confidential. Please answer each question to the best of your ability. Answers should come from you alone, not family, friends, or office personnel. Thank you for your time and participation.  On a scale of 1 to 6, in the past week, how much were you TROUBLED by the following eye allergy symptoms: (1 = not troubled at all, 6 = extremely troubled) | 5/35 (14%) | 6-point Likert Type (trouble): 1 Not troubled at all, 2, 3, 4, 5, 6 Extremely troubled | 1 week | NR | CE | SA | Bother, problem, trouble | Trouble |
| Diabetes Symptom Checklist (DSC) | Type 2 diabetes | People with diabetes can experience various discomforting physical and mental symptoms related to their disease. In order to know how much you are troubled by particular symptoms, we would like you to fill in this questionnaire. Please circle whether you have experienced the symptom or not in the past month, today included. If you circle “yes” then indicate to what extent the symptom listed has caused you discomfort by circling the number that most closely reflects your experience. If a symptom did NOT occur, please circle “No” in the column “DID SYMPTOM OCCUR”  How much trouble have these symptoms given you over the last month? | 34/68 (50%) + 3 optional OE | 5-point Likert (trouble): 1 not at all, 2 a little, 3 moderately, 4 very, 5 extremely | 1 month | NR | CE + 3 optional OE | SA | Bother, distress, trouble | Trouble |
| Measure of Ovarian cancer Symptoms and Treatment concerns Version 1 (MOST V1) | Ovarian cancer | Please circle one number for each line to best show how much that aspect troubled you on average during the last 3 to 4 weeks. | 15/35 (43%) | 11-point NRS (trouble): 0 Not trouble at all, 1, 2 Mild, 3, 4, 5 Moderate, 6, 7, 8 Severe, 9, 10 Worst I can image | 3-4 weeks | NR | CE | SA | Burden, trouble | Trouble |
| Measure of Ovarian cancer Symptoms and Treatment concerns Version 2 (MOST V2) | Ovarian cancer | Please circle one number for each line to best show how much that aspect troubled you on average during the last 3 to 4 weeks. | 13/24 (54%) | 11-point NRS (trouble): 0 Not trouble at all, 1, 2 Mild, e, 4, 5 Moderate, 6, 7, 8 Severe, 9, 10 Worst I can image | 3-4 weeks | NR | CE | SA | Burden, trouble | Trouble |
| Elderly Diabetes Burden Scale (EDBS) | Diabetes in the elderly | The following questions are concerned with your symptoms. If you have the symptoms, please tell us how much you have trouble with them these a few months.  How much trouble do you have with ___? | 4/27 (15%) | 4-point Likert Type (trouble): 1 none, 2 not much, 3 a little, 4 a lot | Few months | 5-10 | CE | SA | Burden | Trouble |
| McMaster University Head and Neck Radiotherapy Questionnaire (HNRQ) | Head and neck cancer treated with radiotherapy | (Insert the number of weeks since previous HNRQ administration) __ weeks ago, you answered a questionnaire designed for people who have received radiation treatment for head and neck cancer. Please repeat the questionnaire today to find out how the treatments have been affecting how you have been feeling during the past week. Please think about how the treatments have been affecting you.  How TROUBLESOME was this for you? | 13/45 (29%) | 6-point Likert Type (trouble): 1 A great deal, 2 A lot, 3 A fair bit, 4 Somewhat, 5 A little, 6 Hardly any | 1 week | 10 | CE | IA | Distress | Trouble |
| Mini Rhinoconjunctivitis Quality of Life Questionnaire (MiniRQLQ) | Adults with rhinoconjunctivitis | Please complete all questions by circling the number that best describes how troubled you have been during the last week as a result of your nose/eye symptoms. | 14/14 (100%) | 7-point Likert (trouble): 0 Not troubled, 1 Hardly troubled at all, 2 Somewhat troubled, 3 Moderately troubled, 4 Quite a bit troubled, 5 Very troubled, 6 Extremely troubled | 1 week | Initial: 7  Follow-up: 2-3 | CE | SA | Trouble | Trouble |
| Rhinoconjunctivitis Quality of Life Questionnaire (RQLQ) | Adults with rhinoconjunctivitis | Please indicate how much you have been troubled by each item, during the last week, as a result of your nose/eye symptoms. Please use the following scale: (6) Extremely troubled (5) Very troubled (4) Quite a bit troubled (3) Moderately troubled (2) Somewhat troubled (1) Hardly troubled at all (0) Not troubled | 15/28 (54%) + 3 optional OE for Visit 1 | 7-point Likert (trouble): 0 Not troubled, 1 Hardly troubled at all, 2 Somewhat troubled, 3 Moderately troubled, 4 Quite a bit troubled, 5 Very troubled, 6 Extremely troubled | 1 week | Initial: 5-10, maximum 15  Follow up: <8, maximum 10 | CE + 3 optional OE | SA, IA | Bother, trouble | Trouble |
| Adolescent Rhinoconjunctivitis Quality of Life Questionnaire (AdolRQLQ) | 12–17-year-olds with allergic rhinoconjunctivitis | Please indicate how much you have been troubled by each item, during the last week, as a result of your nose/eye symptoms. Please use the following scale: 1. Not troubled 2. Hardly troubled at all 3. Somewhat troubled 4. Moderately troubled 5. Quite a bit troubled 6. Very troubled 7. Extremely troubled GREEN = Please indicate how often during the last week you have been troubled by each of these items as a result of your nose/eye symptoms. Please use the following scale: 1. None of the time 2. Hardly any time at all 3. A small part of the time 4. Some of the time 5. A large part of the time 6. Most of the time 7. All of the time | Baseline: 17/28 (60.7%) | 7-point Likert (trouble): 1. Not troubled, 2 Hardly troubled at all, 3 Somewhat troubled, 4 Moderately troubled, 5 Quite a bit troubled, 6 Very troubled, 7 Extremely troubled | 1 week | Not reported | CE + 3 OE | SA, IA | Bother, impact, impair | Trouble |
| Patient-Rated Troubling Symptoms Scale for Anxiety (PaRTS-A) † | Generalised anxiety disorder (GAD) | Welcome to the Telephone Assessment Call. During this study, you will be asked to rate your symptoms, how those symptoms affect your work and social life, and any change you may have felt. For today’s call, it may be helpful to review your 0 to 10 visual scale. First, I’d like for you to record how your anxiety is affecting your life. Please think about your anxiety’s effects on your work and other activities, your family, your health, your personality, and any other aspects of your life that are important to you. Your recording will be replayed for you later in the study to help you remember how you are feeling now, at the beginning of the study.  Fine, let’s begin. Please remember to rate each symptom based on how troublesome it has been during the past week. We’ll start with the first symptom. Please rate on a scale from 0 to 10. 0 means excessive anxiety or worry is not at all troubling for you and 10 means you are extremely troubled by excessive anxiety or worry. Enter a number from 0 to 10 now | 7/9 (78%) | 11-point NRS (trouble) but presented in 2 ways:  Script: 0 not at all troubling; 1 to 3 mildly troubling; 4 to 6 anxiety that is moderately troubling; 7 to 9 markedly troubling, 10 extremely troubled.  Patients also provided a VAS for reference: 0 (none), 1 2 3 (mild), 4 5 6 (moderately), 7 8 9 (very), 10 (most) | 1 week | NR | CE | SA (via interactive touch-tone telephone-based voice response system (IVRS)) | Bother, severity, trouble | Trouble |
| Patient-Rated Troubling Symptoms Scale for Depression (PaRTS-D) † | Depression | / | 9/9 (100%) | 11-point NRS (via an interactive voice response system): 0 absent, 1-3 mild, 4-6 moderate, 7-9 marked, 10 extreme | NR | NR | CE | SA (via interactive touch-tone telephone-based voice response system (IVRS)) | Severity, trouble | Severity, trouble |
| Bolognese Questionnaire † | Benign prostatic hyperplasia | / | 11/23 (48%) | 3-point Likert scale (trouble): 0 Not troublesome, 1 Somewhat troublesome, 2 Very troublesome | NR | NR | CE | SA | Trouble | Trouble |

† Partial or incomplete PROM retrieved only able to be retrieved, therefore some missing data; CE: closed-ended; IA: interviewer-administered; NRS: Numerical rating scale; OE: open-ended; SA: self-administered; VAS: Visual analogue scale

**Table S9 Indirect + Assessed PROMs measuring the construct of ‘distress’ (n = 11)**

| **PROM (abbreviation)** | **Population or health condition** | **Instructions and/or question wording** | **Number of ‘bother’ items / total items (%)** | **Scale type and wording** | **Recall period** | **Average time to complete (minutes)** | **Open- or closed-ended item list** | **Method of completion** | **‘Bother’ synonyms used in manuscript** | **‘Bother’ synonyms used in PROM** |
| --- | --- | --- | --- | --- | --- | --- | --- | --- | --- | --- |
| Symptom Distress Scale (SDS) | Cancer | Below are 5 different numbered statements. Think about what each statement says, then place a circle around the one statement that most closely indicates how you have been feeling lately. The statements are ranked from 1 to 5, where number one indicates no problems and number five indicates the maximum amount of problems. Numbers two through four indicate you feel somewhere in between these two extremes. Please circle one number on each card. | 13/13 (100%) | 5-point Likert (distress): *Different labels were provided for each of the symptom items* | Lately | 5 | CE | SA | Discomfort, distress | Distress |
| Uterine Fibroid Symptom and Health-Related Quality of Life Questionnaire (UFS-QOL) | Uterine fibroids | Listed below are symptoms experienced by women who have uterine fibroids. Please consider each symptom as it relates to your uterine fibroids or menstrual cycle. Each question asks how much distress you have experienced from each symptom during the previous three months. There are no right or wrong answers. Please be sure to answer every question by checking the most appropriate box. If a question does not apply to you, please mark “not at all” as a response.  During the previous three months, how distressed were you by: | 8/37 (22%) | 5-point Likert (distress): 1) None of the time, 2) A little of the time, 3) Some of the time, 4) Most of the time, 5) All of the time | 3 months | 10-15 | CE | SA | Severity | Distress |
| Menopausal Symptoms Scale (MSS) | Menopausal breast cancer patients | DIRECTIONS: During and following treatment for breast cancer, women experience a variety of symptoms. We have listed 46 symptoms below that women may experience. Red each one carefully. If you DID NOT HAVE the symptom in the past week, make an "X" in the tan column marked "DID NOT HAVE". If you had the symptom in the past week, then rate the SEVERITY of the symptom in the yellow column using the 0 to 10 SYMPTOM SEVERITY SCALE below. In addition, rate how much the symptom DISTRESSED OR BOTHERED you in the green column using the green 0 to 10 SYMPTOM DISTRESS SCALE below. | 46/138 (33%) | 11-point NRS (distress): 0 not at all distressing, 1, 2 a little bit distressing, 3, 4, 5, somewhat distressing, 6, 7, 8 quite a bit distressing, 9, 10 very distressing | 1 week | NR | CE | SA | Distress | Bother, distress |
| Lymphedema Symptom Intensity and Distress Survey-Arm (LSIDS-A) | Lymphoedema in the upper limb associated with breast cancer | For each symptom below circle Yes or No to indicate whether you have had this symptom DURING THE PAST WEEK. If you circle Yes, please rate how intense this symptom was using the 1 to 5 point scale. Also rate how distressed you were by this symptom using the 1 to 5 point scale. | 30/90 (33%) | 5-point Likert with descriptive anchors (distress): 1 Slight, 2, 3, 4, 5 Severe | 1 week | <10 | CE | SA | Burden, distress | Distress |
| Lymphedema Symptom Intensity and Distress Survey-Leg (LSIDS-L) | Lymphoedema in the lower limb | For each symptom below circle Yes or No to indicate whether you have had this symptom DURING THE PAST WEEK. If you circle Yes, please rate how intense this symptom was using the 1 to 5 point scale. Also rate how distressed you were by this symptom using the 1 to 5 point scale. | 31/93 (33%) | 5-point Likert with descriptive anchors (distress): 1 Slight, 2, 3, 4, 5 Severe | 1 week | NR | CE | SA | Burden, distress | Distress |
| Lymphedema Symptom Intensity and Distress Survey-H&N V2 (LSIDS-H&N V2) | Lymphoedema in the head and neck | For each symptom below circle Yes or No to indicate whether you have had this symptom DURING THE PAST WEEK. If you circle Yes, please rate how intense this symptom was using the 1 to 5 point scale. Also rate how distressed you were by this symptom using the 1 to 5 point scale. | 31/93 (33%) | 5-point Likert with descriptive anchors (distress): 1 Slight, 2, 3, 4, 5 Severe | 1 week | 5-7 | CE | SA | Burden, distress | Distress |
| Lymphedema Symptom Intensity and Distress Survey-Truncal (LSIDS-T) | Lymphoedema in the trunk | For each symptom below circle Yes or No to indicate whether you have had this symptom DURING THE PAST WEEK. If you circle Yes, please rate how intense this symptom was using the 1 to 5 point scale. Also rate how distressed you were by this symptom using the 1 to 5 point scale. | 24/72 (33%) | 5-point Likert with descriptive anchors (distress): 1 Slight, 2, 3, 4, 5 Severe | 1 week | 5 | CE | SA | Burden, distress | Distress |
| Menstrual Distress Questionnaire (MEDI-Q) † | Menstruation | \ | 75/100 (75%) | 4-point Likert Type (distress): 0 no distress, 1 slight distress, 2 moderate distress, 3 severe distress | 12 months | NR | CE | SA | Distress | Distress |
| General Symptom Distress Scale – Revised (GSIDS-Revised) ‡ | Chronically ill populations | Examiner: *OK, now I am going to read a list of several symptoms. I want you to tell me which symptoms you have had in the past 7 days.* Examiner will read the list in threes. Then, say *if you have the symptom, how distressing it is to you. On a scale of 0 to 10, where 0 = “I do not have this symptom,” 1 = “mildly distressing,” 5= “moderately distressing” and 10 = “extremely distressing,” how distressing is each symptom?*  Ask about other possible symptoms after going through the list from the handbook. Final is other to write in. | 18/18 (100%) + 2 optional open-ended | 11-point NRS (distress): 0 Do not have this symptom, 1 Mildly distressing, 2, 3, 4, 5, Moderately distressing, 6, 7, 8, 9, 10 Extremely distressing | 7 days | NR | CE + 2 optional OE | IA | Distress | Distress |
| IgG4-Related Disease Symptom Distress Index OR The Symptom Severity Index (SSI) | IgG4-related disease (IgG4-RD) | 1. This should reflect any symptoms you may have experienced over the last 30 days. For each symptom, choose how often, if at all, you experience the symptom. 2. If present, choose how much the symptom causes you distress. 3. Indicate each of your answers with an X.  How much distress does the symptom cause you? (Check one) | 27/54 (50%) | 5-point Likert (distress): None, A little bit, Somewhat, Quite a bit, Very much | 30 days | NR | CE + 3 optional OE | SA | Bother, distress, important | Distress, bother, important |
| Patient Symptom Survey (PSS) | Patients in the intensive care unit (ICU) | NR – patients were asked whether symptom was present, and if present, its intensity and distress | 10/30 (33%) | 3-point Likert (distress): 1 Not very distressing, 2 Moderately distressing, 3 Very distressing | NR | NR | CE | IA | Distress | Distress |

† Partial or incomplete PROM retrieved only able to be retrieved, therefore some missing data; ‡ Data extracted from revised version of the GSDS as per PROM provided by author. NRS: Numerical rating scale

**Table S10 Indirect + Assessed PROMs measuring the mixed construct of ‘bother and distress’ (n = 7)**

| **PROM (abbreviation)** | **Population or health condition** | **Instructions and/or question wording** | **Number of ‘bother’ items / total items (%)** | **Scale type and wording** | **Recall period** | **Average time to complete (minutes)** | **Open- or closed-ended item list** | **Method of completion** | **‘Bother’ synonyms used in manuscript** | **‘Bother’ synonyms used in PROM** |
| --- | --- | --- | --- | --- | --- | --- | --- | --- | --- | --- |
| CKD Symptom Burden Index (CKD-SBI) | Chronic kidney disease | Below is a list of physical and emotional symptoms that people with chronic kidney disease may have. Read each one carefully. If you have had the symptom during the past four weeks, let us know how OFTEN you had it, how SEVERE it was usually and how much it DISTRESSED or BOTHERED you by circling the appropriate number.  How much did it distress or bother you on a scale of 0-10? 0 = none 10= very much | 32/128 (15%) + 3 optional open-ended | 11-point NRS (bother and distress): 0 none, 1, 2, 3, 4, 5, 6, 7, 8, 9, 10 very much | 4 weeks | 15-30 | CE + 3 optional OE | SA | Distress | Bother, distress |
| Memorial Symptom Assessment Scale - Heart Failure (MSAS-HF) | Heart failure | Section 1: We have listed 26 symptoms below. Read each one carefully. If you have had the symptom during this past WEEK, let us know how OFTEN you had it, how SEVERE it was usually and how much it DISTRESSED OR BOTHERED you by circling the appropriate number. If you DID NOT HAVE the symptom, make an “X” in the box marked “DID NOT HAVE.” Section 2: We have listed 6 symptoms below.  Read each one carefully.  If you have had the symptom during the past week, let us know how SEVERE it was usually and how much it DISTRESSED OR BOTHERED you by circling the appropriate number.  If you DID NOT HAVE the symptom, make an “X” in the box marked “DID NOT HAVE.”  How much did it DISTRESS or BOTHER you? | 32/90 (36%) + 3 optional open-ended | 5-point Likert (bother and distress): 0 Not at all, 1 A little bit, 2 Somewhat, 3 Quite a bit, 4 Very much | 1 week | NR | CE + 3 optional OE | SA | Distress | Bother, distress |
| Memorial Symptom Assessment Scale - Paediatric 10-18 (MSAS (10-18)) | Children with cancer aged 10-18 years | We have listed 22 symptoms below. Read each one carefully. If you have had the symptom during this past week, circle YES. If YES, let us know how OFTEN you had it, how SEVERE it was usually and how much it BOTHERED OR DISTRESSED by circling the appropriate answer. If you DID NOT HAVE the symptom circle NO.  How much did it distress or bother you? | 31 /116 (27%) | 5-point Likert (bother and distress): 0 Not at all, 1 A little bit, 2 Somewhat, 3 Quite a bit, 4 Very Much | 1 week | NR | Original: CE  Revised: CE + 1 optional OE | SA | Distress | Bother, distress |
| Memorial Symptom Assessment Scale (MSAS) | Cancer | Instructions: We have listed 24 symptoms below. Read each one carefully. If you have had the symptom during this past week, let us know how OFTEN you had it, how SEVERE it was usually and how much it DISTRESSED or BOTHERED you by circling the appropriate number. If you DID NOT HAVE the symptom, make an "X" in the box marked "DID NOT HAVE."  If yes, how much did it DISTRESS or BOTHER you? | 32/120 (27%) + 3 optional open-ended | 5-point Likert (bother and distress): 0 Not at all, 1 A little bit, 2 Somewhat, 3 Quite a bit, 4 Very Much | 1 week | <15 | CE + 3 optional OE | SA | Distress | Bother, distress |
| Memorial Symptom Assessment Sale Short Form (MSAS-SF) | Cancer | Below is a list of symptoms. If you had the symptom DURING THE PAST WEEK, please check Yes. If you did have the symptom, please check the box that tells us how much the symptom DISTRESSED or BOTHERED you.  How much did it DISTRESS or BOTHER you? | 27/32 (84%) + 2 optional open-ended | 5-point Likert (bother and distress): 0 Not at all, 1 A little bit, 2 Somewhat, 3 Quite a bit, 4 Very much | 1 week | <5 | CE + 2 optional OE | SA | Bother, distress | Bother, distress |
| Condensed Memorial Symptom Assessment Scale (Condensed MSAS) | Adult cancer patients | How much did this symptom bother or distress you in the past 7 days | 11/ 14 (79%) | 5-point Likert (bother and distress): 0 Not at all, 1 A little bit, 2 Somewhat, 3 Quite a bit, 4 Very much | 1 week | 2-4 | CE | SA | Distress | Bother, distress |
| Memorial Symptom Assessment Scale-Long COVID (MSAS-LC) | Post-Acute COVID-19 Syndrome (Long COVID) | We have listed symptoms of Long COVID below. Read each one carefully. If you have had the symptom during the past 3 MONTHS, let us know how OFTEN you had it, how SEVERE it usually was, and how much it  DISTRESSED or BOTHERED you, by circling the appropriate number. If you DID NOT HAVE the symptom, make an "X" in the box marked "DID NOT HAVE."  How much did it DISTRESS or BOTHER you? | 45/135 (33%) | 5-point Likert (distress or bother): 0 Not at all, 1 A little bit, 2 Somewhat, 3 Quite a bit, 4 Very much | 3 months | NR | CE + 7 optional OE | SA | Distress | Bother, distress |

CE: closed-ended; NR: not reported; NRS: numerical rating scale; OE: open-ended; SA: self-administered

**Table S11 Indirect + Assessed PROMs measuring the mixed construct of ‘bother and trouble’ (n = 1)**

| **PROM (abbreviation)** | **Population or health condition** | **Instructions and/or question wording** | **Number of ‘bother’ items / total items (%)** | **Scale type and wording** | **Recall period** | **Average time to complete (minutes)** | **Open- or closed-ended item list** | **Method of completion** | **‘Bother’ synonyms used in manuscript** | **‘Bother’ synonyms used in PROM** |
| --- | --- | --- | --- | --- | --- | --- | --- | --- | --- | --- |
| Memorial Symptom Assessment Scale - Paediatric 7-12 (MSAS (7-12)) | Children with cancer aged 7-12 years | We want to find out how you have been feeling the last 2 days Use a pencil or crayon to circle your answers.  How much did ___ bother you or trouble you? | 8/28 (29%) + 2 optional OE | 4-point Likert-Type (bother and trouble): 0 Not at all, 1 A little, 2 A medium amount, 3 Very much | 2 days | 5.8 | CE + 2 optional OE | SA | Distress | Bother, trouble |

CE: closed-ended; SA: self-administered; OE: open-ended

**Table S12 Indirect + Assessed PROMs measuring the construct of ‘burden’ (n = 1)**

| **PROM (abbreviation)** | **Population or health condition** | **Instructions and/or question wording** | **Number of ‘bother’ items / total items (%)** | **Scale type and wording** | **Recall period** | **Average time to complete (minutes)** | **Open- or closed-ended item list** | **Method of completion** | **‘Bother’ synonyms used in manuscript** | **‘Bother’ synonyms used in PROM** |
| --- | --- | --- | --- | --- | --- | --- | --- | --- | --- | --- |
| Leiden Bother and Needs Questionnaire for patients with Pituitary disease (LBNQ-Pituitary) | Pituitary disease | This survey investigates the disease impact and potential healthcare and coaching of patients treated for pituitary gland conditions. Using the following questionnaire, we wish to map condition-related experiences of patients with a pituitary disease. We also wish to use this questionnaire to identify patients’ need for both healthcare and coaching in how to deal with specific symptoms and consequences. We would like to ask you to consider a number of statements about the consequences you (may) experience in your daily life as a result of your pituitary condition. We would like to ask you to circle/tick the answer that most closely represents your opinion. There are no right or wrong answers. If you are unsure about a given answer, try to select the answer that is most applicable. On the next page, you can see an example. EXAMPLE Every question consists of three elements. Part A consists of a statement regarding a difficult problem. You will be asked to indicate whether this statement applies to you: You will then be asked to indicate to what extent you experience this problem. You can do so by checking the box that most closely matches your opinion: Finally you will be asked to indicate how important you find it that the care you receive addresses this specific problem. You can do so by checking the box that most closely matches your opinion:  This problem and its consequences are a burden to me. | 15/99 (15%) + 1 optional OE | 5-point Likert (burden): Not at all, Slightly, Moderately, Considerably, Extremely | NR | NR | CE +1 optional OE | SA | Bother | Burden |

CE: closed-ended NR: not reported; OE: open-ended; SA: self-administered

**Table S13 Indirect + Unassessed PROMs measuring the construct of ‘bother’ (n = 15)**

| **Paper reference** | **Population or health condition** | **Scale type and wording** | **Recall period** | **Open- or closed-ended item list** | **Number of ‘bother’ items (symptoms)** | **Method of completion** | **‘Bother’ synonyms used in manuscript** | **‘Bother’ synonyms used in PROM / description of PROM** |
| --- | --- | --- | --- | --- | --- | --- | --- | --- |
| Bethoux & Marrie, 2016 | Multiple sclerosis with spasticity | 4-point Likert Type (bother): 0 not at all, 1 slightly bothered, 2 moderately bothered, 3 greatly bothered | NR | CE | 4 | SA survey | Bother | Bother |
| Bielory et al., 2014 | Ocular and/or nasal allergies | 4-point Likert Type (bother): Extremely bothersome, bothersome, slightly bothersome, not bothersome | NR | CE | 9 | Telephone interview | Bother | Bother |
| Blais et al., 2015 | Acute viral upper respiratory tract infection (i.e. the common cold) | 4-point Likert Type (bother): Does not bother me, Bothers me a little bit, Bothers me a fair amount, Bothers me very much | NR | CE | 16 | SA survey | Bother | Bother |
| Cella et al., 2021 | Patients with acute myeloid leukemia in the posttransplant period | 11-point NRS (bother): 0 -10 (most bothersome) | NR | OE | N/A | One-on-one patient interviews conducted via telephone | Bother | Bother |
| Danial et al., 2015 | Epidermolysis bullosa | 5-point Likert (bother): 1 not bothersome, 2 a little bothersome, 3 somewhat bothersome, 4 very bothersome, 5 most bothersome | NR | CE | 13 | Telephone survey | Bother | Bother |
| Demoly et al., 2016 | House dust mite respiratory allergy | Post inclusion questionnaire: 4-point Likert Type (bother): Post inclusion questionnaire: Not bothered at all, slightly bothered, very bothered, extremely bothered  Final questionnaire: 4-point Likert Type (bother): Final questionnaire: Not bothersome, Slightly bothersome, Very bothersome, Extremely bothersome | NR | Post-inclusion questionnaire: CE + optional OE  Final questionnaire: CE | Post-inclusion questionnaire: 12 + 2 optional OE  Final questionnaire: 12 | Internet- and telephone-based questionnaire | Trouble, bother | Bother |
| Faubion et al., 2012 | Menopause | 4-point Likert Type (bother): Not at all, A little bit, Quite a bit, Extremely | Few weeks | CE | 33 | SA questionnaire | Bother | Bother |
| Fromer et al., 2013 | Allergic rhinitis | 11-point scale (bother): Not all descriptors reported – Extremely bothersome, very bothersome, minimally bothersome, not at all bothersome | NR | NR | NR | Telephone interview | Bother | Bother |
| Johanson & Kralstein, 2006 | Chronic constipation | 5-point Likert (bother): Not at all, not very, somewhat, very, extremely bothersome | NR | CE. Unclear if OE but participants reported 'other' symptoms in results. | NR | SA online survey | Bother | Bother |
| Kiuchi et al., 2020 | Post micturition dribble | 4-point Likert Type (bother): None, mild, moderate, severe | NR | CE | 3 | SA questionnaire | Bother | Bother |
| Lee et al., 2001 | Stem cell transplantation for hematologic diseases | 5-point Likert (bother): I do not have this symptom, not bothered at all, bothered a little, bothered a lot, extremely bothered | NR | CE | 12 | SA survey | Bother | Bother |
| Liu et al., 2002 | Post laparoscopic anti-reflux surgery for gastroesophageal reflux disease | Scale type not reported (bother): Full rating scale not reported. Some options reported include "a lot" or "terribly", but not clear | 2 weeks | CE | 6 | SA mailed survey; Telephone survey | Bother | Bother |
| McCarrier et al., 2015 | Advanced non-small cell lung carcinoma | 11-point NRS (bother): 0-11 | 7 days | NR | NR | Semi-structured interviews | Bother, importance | Bother |
| Rosemurgy et al., 2012 | Achalasia patients who have undergone laparoscopic heller myotomy with anterior fundoplication | 10-point Likert Type (bother): 1 least bothersome / never, 10 most bothersome / always | NR | NR | NR | SA questionnaire | Bother, frequency, severity | Bother, frequency |
| Soliman et al., 2017 | Endometriosis | 3-point Likert (bother): Not at all bothersome, Somewhat bothersome, Extremely bothersome | 1 month | CE | NR | SA online survey | Bother | Bother |

CE: closed-ended; NR: not reported; NRS: numerical rating scale; N/A: not applicable; SA: self-administered

**Table S14 Summary of characteristics of Indirect PROMs (n = 135)**

| **Characteristics** | | **Assessed PROMs (n=120)** | **Unassessed PROMs (n=15)** |
| --- | --- | --- | --- |
| **Recall period** | At the moment / currently | 3 |  |
|  | 24 hours | 3 |  |
|  | Today | 2 |  |
|  | Yesterday or today | 1 |  |
|  | 2 days | 1 |  |
|  | 7 days | 17 | 1 |
|  | 1 week | 24 |  |
|  | 2 weeks | 7 | 1 |
|  | Lately | 1 |  |
|  | Since last visit | 1 |  |
|  | Few weeks / recent weeks | 2 | 1 |
|  | 3-4 weeks | 2 |  |
|  | 4 weeks | 18 |  |
|  | 30 days | 1 |  |
|  | 1 month | 16 | 1 |
|  | 3 months | 7 |  |
|  | Few months | 1 |  |
|  | 6 months | 1 |  |
|  | 12 months | 1 |  |
|  | 1 year | 1 |  |
|  | Not reported | 14 | 11 |
| **Open or closed-item list** | Open-ended |  | 1 |
|  | Closed-ended | 103 | 11 |
|  | Closed-ended + 1 optional open-ended | 5 | 1 |
|  | Closed-ended + 2 optional open-ended | 3 |  |
|  | Closed-ended + 3 optional open-ended | 9 |  |
|  | Closed-ended + 7 optional open-ended | 1 |  |
|  | Not reported |  | 3 |
| **Scale type =122** | 3-point Likert | 4 | 1 |
|  | 3-point Likert (pictorial) | 1 |  |
|  | 4-point Likert Type | 22 | 6 |
|  | 5-point Likert | 44 | 3 |
|  | 6-point Likert Type | 9 |  |
|  | 7-point Likert | 16 |  |
|  | 10-point Likert Type |  | 1 |
|  | 4-point NRS | 1 |  |
|  | 6-point NRS | 1 |  |
|  | 11-point NRS | 17 | 2 |
|  | 5-point verbal descriptor scale | 1 |  |
|  | 6-point verbal descriptor scale | 1 |  |
|  | 10cm VAS | 1 |  |
|  | 10cm VAS with 7 written descriptors and 5 faces | 1 |  |
|  | VAS with anchors | 1 |  |
|  | 7-point numerical scale | 2 |  |
|  | 11-point scale (unspecified) |  | 1 |
|  | Not reported |  | 1 |
| **Method of completion** | Self-administered | 113 * | 13 † |
|  | Interviewer-administered | 15 | 4 ‡ |

*** N = 1 PROM was proxy-administered; † N= 3 PROMs were self-completed using a telephone; ‡ N = 3 PROMs were interviewer administered over the telephone; NRS: numerical rating scale; VAS: visual analogue scale; N = 122 Indirect Assessed scale type due to two PROMs having two different rating scales; N = 124 Indirect Assessed recall period due to four PROMs having two recall periods; N = 121 Indirect Assessed open- or closed-ended item list due to one PROM having both types; N = 128 Indirect Assessed method of completion due to six PROMs having two modes and one PROM having three modes; N = 16 Indirect Unassessed open- or closed-ended item lists due to one PROM having two versions depending on time of administration; N = 17 Indirect Unassessed method of completion due to two PROMs having two methods of completion.

**Table S15 Synonyms used within the PROMs, as sorted by construct measured by the PROMs (n = 171)**

|  | Bother | Trouble | Distress | Annoy | Burden | Problem | Intensity | Discomfort | Frequency | Severity | Avoid | Interfere | Impact | Inconvenience | Affect | Satisfaction | Impairment | Importance | Disabling |
| --- | --- | --- | --- | --- | --- | --- | --- | --- | --- | --- | --- | --- | --- | --- | --- | --- | --- | --- | --- |
| Bother, n = 130 | 127 |  | 7 |  |  |  | 1 | 1 | 1 | 1 | 1 | 1 | 2 | 1 | 1 | 1 | 1 | 1 | 1 |
| Trouble, n = 19 |  | 19 |  |  |  | 2 | 1 |  |  | 1 |  |  |  |  |  |  |  |  |  |
| Distress, n = 11 | 2 |  | 11 |  |  |  |  |  |  |  |  |  |  |  |  |  |  | 1 |  |
| Annoy, n = 2 |  |  |  | 1 |  |  |  | 1 |  |  |  |  |  |  |  |  |  |  |  |
| Burden, n =1 |  |  |  |  | 1 |  |  |  |  |  |  |  |  |  |  |  |  |  |  |
| Bother and distress, n = 7 | 7 |  | 7 |  |  |  |  |  |  |  |  |  |  |  |  |  |  |  |  |
| Bother and trouble, n = 1 | 1 | 1 |  |  |  |  |  |  |  |  |  |  |  |  |  |  |  |  |  |

3 PROMs were unable to be extracted due to incomplete data from one PROM and two not formally been developed due to existing from FDA guidance documents

4

**Table S16 Synonyms used within the studies, as sorted by the construct measured by the PROMs (n=171)**

|  | Bother | Trouble | Distress | Annoy | Burden | Problem | Discomfort | Frequency | Severity | Impair | Impact | Importance | Disturb | Hardest | Affect |
| --- | --- | --- | --- | --- | --- | --- | --- | --- | --- | --- | --- | --- | --- | --- | --- |
| Bother, n = 130 | 111 | 7 | 13 |  | 9 | 2 |  | 1 | 11 |  | 7 | 4 | 2 |  | 1 |
| Trouble, n = 19 | 7 | 16 | 2 |  | 3 | 2 |  |  | 2 | 1 | 2 | 1 |  | 1 |  |
| Distress, n = 11 | 1 |  | 9 |  | 4 |  | 1 |  | 1 |  |  | 1 |  |  |  |
| Annoy, n = 2 |  |  |  | 1 | 1 |  | 1 |  |  |  |  |  |  |  |  |
| Burden, n =1 |  |  |  |  | 1 |  |  |  |  |  |  |  |  |  |  |
| Bother and distress, n = 7 | 1 |  | 7 |  |  |  |  |  |  |  |  |  |  |  |  |
| Bother and trouble, n = 1 |  |  | 1 |  |  |  |  |  |  |  |  |  |  |  |  |

**Table S17 Characteristics of studies included in COSMIN psychometric analysis**

| **PROM** | **Reference** | **Sample size  (% female)** | **Age – Mean (SD)** | **Health condition(s)** | **Country (language)** | **Setting** | **Response rate** |
| --- | --- | --- | --- | --- | --- | --- | --- |
| **GSM-SVTAQ** | Hassanein et al., 2024 | PROM development: 50 (100%)  Pilot testing: 12 (100%)  Psychometric testing: 72 (100%) | Study 3:  45-59: n=47  60-75: n=25 | Menopause | United Arab Emirates (English and French, % not reported) | Not specified | 72/72 (100%) |
| **MQOL** | Cohen et al., 1995 | Translation: 3 (NR)  40 (70%) | 66 (38-88) | Advanced cancer and receiving palliative care at home or in the inpatient unit | Canada (English – 62.5%; French – 37.5%) | Palliative care service – home care and inpatient unit | 40/43 (93%) |
|  | Cohen et al., 1996 | 247 (57.5%) | 51 (20-88) | Any stage of cancer | Canada (English – 66.4%; French – 33.6%) | Oncology day centre | 247/254 (97%) |
|  | Cohen, Hassan et al., 1996 | 109 (6%) | NR | HIV | Canada (English, French (% NR)) | Two outpatient clinics in Montreal | NR |
|  | Cohen et al., 1997 | 143 (61%) | 65 (12) | Palliative care | Canada (English – 60%; French – 40%) | Eight palliative care services in four cities; all dedicated palliative care inpatient units or a palliative home care service | 143/150 (95%) |
|  | Cohen & Mount, 2000 | 100 (59%) | 59 (24-87) | Cancer | Canada (English 69%; French 31%) | Patients attending an oncology day centre and receiving chemotherapy, or being treated by a palliative home care service | 99/100 (99%) |
|  | Tsujikawa et al., 2009 | 83 (45.8%) | 68.9 (10.1) | Terminal cancer receiving palliative care | Japan (Japanese) | Inpatient hospice or palliative care wards | NR |
|  | Lo et al., 2001 | 462 (47%) | 61.5 (1.5) | Advanced incurable cancer patients receiving palliative care | Hong Kong (Chinese) | Three palliative care providers in Hong Kong | NR |
|  | Gofton et al., 2015 | 81 (37%) | 65.2 (8.4) | Parkinson’s Disease | Canada (English) | Tertiary care neurology outpatient clinic | NR |
|  | Bentur & Resnizky, 2005 | Translation: 2 (NR)  160 (56%) | 66.8 (NR) | Advanced cancer | Israel (Hebrew) | Seven home hospice units (palliative care) | NR |
|  | Hu et al., 2003 | Translation: 9 (NR)  64 (37.5%) | 47.77 (16.23) | Terminal advanced cancer | Taiwan (Chinese) | Palliative care unit and oncology wards of National Taiwan University Hospital | 64/74 (86%) |
|  | Hu et al., 2015 | Translation: 6 (NR)  126 (44.4%) | 48.9 (15.8) | Cancer (all stages) | China (Chinese) | Admitted patients from two hospitals | 126/138 (91%)  84/138 test-retest reliability (61%) |
|  | Sguazzin et al., 2010 | Translation: 18 (NR)  175 (38.3%) | Mean 64.9 (12.9) | Cancer, CHF, ALS | Italy (Italian) | Palliative care unit inpatients | NR |
|  | Kim et al., 2007 | Translation: 20 (NR)  140 (47.9%) | 56.7 (12.5) | Terminal cancer (progressive and no longer treatable) | Korea (Korean) | Outpatient or inpatients from six conventional care facilities | 140/144 (97%) |
|  | Shahidi et al., 2008 | Translation: NR    61 (47.5%) | 51 (15.3) | Any type of incurable cancer with estimated life expectancy of less than 12 months | Iran (Persian) | Radiation oncology outpatient clinics in 2 hospitals | 61/62 (98%) |
|  | Tolentino & Sulmasy, 2002^†^ | Translation: 8 (NR) | Not relevant | Not relevant | United States of America (Spanish) | Not relevant | Not relevant |
|  | Pratheepawanit et al., 1999 | 36 (53%) | 64 (10.81) | Advanced cancer receiving palliative care | United Kingdom (Wales) (English) | Outpatient palliative care clinic | 36/38 (95%) |
|  | De Vrieze et al., 2016 | 26 (100%) | 63 (11) | Breast cancer patients who had finished their initial treatment (except hormone or targeted therapy) | Belgium (Dutch) | Antwerp University Hospital Interdisciplinary outpatient Breast Clinic | 26/30 (87%) |
|  | Zhang et al., 2003 | Doctors 156 (50.64%)  Nurses 286 (87.76%)  Families 120 (56.67%) | N = 562  <29 y = 48%^f^  30-39 y = 37%^f^  40-49 y = 9%^f^  >50 y = 6%^f^ | Not relevant | China (Chinese) | Inpatient ICU | Not relevant |
| **MUDI** | Robinson & Shea, 2002 | 139 (0%) | 65 (30-90)^a^ | Men with concerns about urine leakage | United States of America (English) | Urology outpatient clinic at large urban Veterans Affairs Medical Centre | 139/139 (100%) |
|  | Lim et al., 2009 | Translation, back-translation: 2 (NR)  Content equivalence: 1 (100%)  Semantic equivalence: NR (NR)  Field testing: 5 (NR)  Psychometric testing: 143 (0%) | 66.9 (8.5) | Lower urinary tract symptoms, with episodes of urine leakage | Korea (Korean) | Urology outpatient clinic at a suburban general hospital in Korea | 143/143 (100%) |
|  | Robinson et al., 2007 | 100 (0%) | 59.6 (6.2) | Prostate cancer who elected radical prostatectomy as primary treatment | United States of America (English) | Urology outpatient practices of two academic medical centres | 100/114 (88%) |
| **MYMOP1** | Rosenberg et al., 2022 | Translation: 4 (NR)  Pilot testing: NR | NR | Pilot testing: patients with late complications after treatment for colorectal and anal cancer | Denmark (Danish) | NR | NR |
|  | Chung et al., 2010 | Study 1 Translation process: 8 (NR)  Study 2 Pilot testing: 15 professionals (NR) and 28 patients (NR)  Study 3 Psychometrics: 539 recruited, 272 completed all follow ups and only reported on (83.8%) | Study 1: Not relevant  Study 2: NR  Study 3:  <20: 4.4%^f^  20-29: 13.6%^f^  30-39: 22.1%^f^  40-49: 22.4%^f^  50-59: 21.3%^f^  60-69: 9.6%^f^  70-79: 5.9%^f^  >79: 0.7%^f^ | Self-reporting any specific symptoms in past 14 days | China (Chinese) | Chinese medicine outpatient clinic | 2/52 follow up: 343/539 (64%)  4/52 follow up:  272/539 (50%) |
|  | Paterson et al., 2000 | 81 (53%) | 61 (32.4 - 82.8)^a^ | Acute exacerbation of type-1 chronic bronchitis | United Kingdom (English) | General private practice setting | Missing data for Symptom 1 77/81 (95%) and Symptom 2 75/81 (93%) |
|  | Peterson & Britten, 2000 | 20 (NR) | 16 – 86^d^ | New patients seeking treatment from a complementary practitioner | United Kingdom (English) | Outpatient treatment of complementary treatment | 20/26 |
|  | Paterson, 1996 | 265 (69%): 218 from general practice, 47 from complementary practitioners | 47 (17.6) | Experiencing symptoms >7 days | United Kingdom (English) | Outpatient clinic (medical and complementary practitioners) | NR |
|  | Murphy, 2018 | 20 (65%) | 18-34 y = 25%^f^  35-54 y = 25%^f^  55-64 y = 15%^f^  65-74 y = 20%^f^  75+ y = 15%^f^ | Recent symptoms | United Kingdom (English) | Outpatient clinics in UK | 17/20 completed the MYMOP |
| **MYMOP2** | Ersoy et al., 2019 | Study 1 Translation and back translation: 5 (NR)  Study 2 Pilot patient testing: 5 (NR)  Psychometrics:  50 (66%) | Study 1: Not relevant  Study 2: NR  Study 3 (n=50):  ≤ 25 y:32%^f^  25-40 y: 36%^f^  ≥ 40 y: 32%^f^ | Benign anorectal problems (chronic constipation, anal incontinence, chronic pelvic pain) | Turkey (Turkish) | Inpatients admitted to the center for Hemorrhoids and Anorectal Diseases at Fulya Acıbadem Hospital to receive biofeedback treatment | 50/57 (88%) |
|  | Herman et al., 2014 | Study 1 (translation and back translation): 4 (NR)  Study 2 (pilot study with patients): 16 (NR)  Study 3: Psychometrics  567 (64.9%);  476 from general practice  91 from CAM practitioners | Study 1: Not relevant  Study 2: Not reported  Study 3 (n = 567)  50.1 (17.9) | Newly developed symptoms | Germany (German) | Primary care | Follow up questionnaire: 341/567 (60.1%) |
|  | Paterson, 2004 | 23 (78%) | 26-83^d^ | Primary care acupuncture patients | United Kingdom (English) | Outpatient acupuncture clinics | 64 interviews conducted (N = 18 were interviewed three times; N = 5 were interviewed two times) |
| **NMF2S** | Faggianelli et al., 2024 | Psychometric study: 310 (101, 35.5%) | 59.6 (7.7) | Parkinson’s disease | France (French) | 17 centres in France, presurgical for deep brain stimulation of subthalamic nuclei | 310/310 (100%) |
| **POSQ** | Matza et al., 2005 | 47 (74.5%) | 66 (NR) | Clinically diagnosed OAB, including presence of urinary urgency with or without urinary incontinence | United States of America (English) | Five urology outpatient clinics | 46/47 (98%) |
| **PSYCHLOPS** | Ashworth, 2004 | Adapting PROM: Number not reported  Patient piloting: 30 (NR) | NR | Patients receiving therapy in primary care | United Kingdom (English) | Outpatient therapy | NR |
|  | Sales et al., 2021^‡^ | 825 (67.9%) | 39.9 (12.8) | Sample 1: Mental health service  Sample 2 – Sample 6* | Sample 1: Portugal (Portuguese)  Sample 2 – Sample 6* | Sampe 1: Outpatient mental health services  Sample 2 – Sample 6* | 825/939 (88%) |
|  | Austin et al., 2021 | 278 (93%) | 20.19 (2.39) | Eating disorders | United Kingdom (English) | Outpatient early intervention for eating disorders - First Episode Rapid Early Intervention for Eating Disorders (FREED) | 3/12: N=216 (78%)  6/12: N=182 (65%)  12/12: N=175 (63%) |
|  | Ashworth et al., 2009 | 114 (56%) | 39 (17-76)^a^ | Psychological therapy | United Kingdom (English) | Clinical psychology services in primary care | 114/336 (33.9%) |
|  | Ashworth, Robinson, Godfrey, Shepherd et al., 2005 | 110 (71%) | 32 (15-64)^a^ | Psychological therapy | United Kingdom (English) | Clinical psychology in primary care setting | 110/235 (47%) |
|  | Ashworth, Robinson, Godfrey, Parmentier et al., 2005^†^ | 4 (50%) | Not relevant | Not relevant | United Kingdom (English) | Primary care psychology services | 4/4 (100%) |
|  | Evans et al., 2010 | 73 (74%) | NR | NR | United Kingdom (English) | Students in three higher education institutions | Retest data: 56/73 (77%) |
|  | Alves et al., 2020 | 93 (41%) | 43 (11) | Substance misuse | Portugal (Portuguese) | Four services for drug and alcohol substance misuse | 93/94 (99%) |
|  | Heoinsson et al., 2013 | Translation: NR    Psychometrics: 100 (75%) | 37.5 (12.0) | Low self-esteem, mood or anxiety disorders | Iceland (Icelandic) | Cognitive behaviour group therapy at a hospital in Iceland | 100/215 analysed (46%) |
|  | Czachowski et al., 2011 | 238 (78.1%) | 41.5 (18-64)^a^ | Psychosomatic symptoms, anxiety, depression | Poland (Polish) | GP clinics | 238/243 (98%) |
| **Questionnaire for female lower urinary tract symptoms and their impairment of quality of life** | Bernstein et al., 1996 | Questionnaire design panel: NR  Preliminary pilot study: 56 (100%) | 54.5 (18-79)^c^ | LUTS | Denmark (English) | Community participants referred from six outpatient department clinics | 56/56 (100%) |
| **QUAL-E** | Steinhauser et al., 2002 | 200 (26.5%) | 62 (34-84)^a^ | At least one of four life-threatening conditions: Stage IV cancer (64%), CHF (19.5%), ESRD (10%), COPD (6.5%) | United States of America (English) | Oncology, heart failure, pulmonary, and dialysis outpatient clinics at Durham Veterans Affairs and Duke Medical Centres | 200/234 (85%) |
|  | Steinhauser et al., 2004 | 248 (41%) | 64 (22-88)^a^ | Stage IV cancer, CHF with ejection fraction ≤ 20%, COPD with FEV ≤ 1.01, dialysis dependent ESRD | United States of America (English) | Oncology, heart failure, pulmonary, and dialysis outpatient clinics at Durham Veterans Affairs and Duke Medical Centres | 248/325 (76%) |
|  | Wilkinson et al., 2014 | 52 (56%) | 67.35 (NR) | Patients receiving palliative care. Primary diagnosis of cancer (86%) or non-cancer diagnoses (cardiovascular disease, liver failure, ESRD, other non-malignancies) | Australia (English) | Acute care inpatients in public hospitals in Perth, Western Australia | 52/52 (100%) |
| **QUAL-EC** | Lo et al., 2021 | 468 (55%) | 61 (12) | Patients with advanced cancer enrolled in an ongoing cluster randomised controlled trial of early palliative care intervention vs routine oncology care for metastatic gastrointestinal, genitourinary, breast, lung, or gynaecological cancer | Canada (English) | 24 outpatient oncology clinics at Princess Margaret Hospital, Toronto, Canda | 464/468 (99%) |
| **SF-LDQ** | Fraser et al., 2007 | 592 (61%)  388 primary care patients  204 secondary care patients | Primary care: 18-65^d^  Secondary care: 18-81^d^ | Dyspepsia | United Kingdom (English) | Primary care patients: two inner city general practices in Brimingham  Secondary care patients: attending for endoscopy at the Leeds General Infirmary | 537/592 (91%) |
|  | Alabulkareem et al., 2021 | Translation and back-translation: 4 (NR)  245 (61.1%) | 52.2 (13.2) | Type II diabetes | Saudi Arabia (Arabic) | Community patients from Diabetes Clinic at King Abdullah Bin AbdulAziz University Hospital | 244/245 (99.6%) |
|  | Gatta et al., 2010 | Translation: 4 (NR)  469 (60.1%) | Primary care: 50 (35-61)^e^  Secondary care: 49 (39-62)^e^ | Dyspepsia | Italy (Italian) | Primary care: patients presenting to three city general practitioners  Secondary care patients: outpatients attending for upper endoscopy | 469/490 (96%) |
|  | Nkurunziza et al., 2016 | Translation: 10 (NR)  Pilot test: 10 (70%)  200 (62%) | 41 (NR) | Dyspepsia | Rwanda (Kinyarwanda) | Outpatients from a referral hospital in Rwanda or those awaiting EGD at same location | 200/200 (100%) |
| **UDI** | Shumaker et al.,1994 | 162 (100%) | 61.3 (±10.2) | At least one episode of UI per week fulfilling urodynamic criteria of genuine stress incontinence and/or detrusor instability | United States of America (English) | Two medical schools in Southeast United States | 162/162 (100%) |
|  | Harvey et al., 2001 | 150 (100%) | 55 (11.8) | UI | United States of America (English) | Prospective before/after clinical trial with participants recruited via referral from an academic urogynaecology practice or from the community | 98/155 (97%) |
|  | Hagen et al., 2002 | 237 (100%)  Community: 79  Clinic: 75  Surgery: 83 | Community: 76 (12)  Clinic: 50 (14)  Surgery: 50 (12) | UI | United Kingdom (Scotland) (English) | Community dwelling people, outpatient continence clinic, surgical waiting list | 210/237 (89%) |
|  | van der Vaart et al., 2003 | Translation process: NR  2239 (100%)  Community: 2043  Clinic: 196 | Community: 46.5 (0.3)^b^  Clinic: 54.7 (0.8)^b^ | Community: urogenital and defecation symptoms  Outpatient clinic: Urogenital symptoms | Netherlands (Dutch) | Community  Gynaecology outpatient clinic of the University Medical Center of Utrecht, The Netherlands | NR |
|  | Artibani et al., 2006 | Translation: 3 translators and the research team (NR)  Qualitative pilot testing: 9 (100%)  Psychometrics evaluation: 106 (100%) | NR | LUTS | Italy (Italian) | Two health centres in Italy | NR |
| **U-UDI** | Brown, 1999 | 83 (100%) | 63.8 (11.6) | Urge or mixed incontinence with a primary urge component | United States of America (English) | Community sample recruited via newspaper advertisements, flyers in seniors’ centres, and urology and gynaecology clinics at the University of California, San Francisco | 82/83 (99%) |
|  | Lubek et al., 1999 | 257 (91.4%) | 59.6 (13.9) | Non-neurogenic urge incontinence, neurogenic urge incontinence, or mixed incontinence with a clinically significant urge component | United States of America (English) | Multicentre, open-label, nonrandomised, single treatment study with participants recruited from the offices of 16 urologists | 256/257 (99.6%) |
|  | Borello France et al., 2008 | 57 (100%) | 48.6 (±8.3 years) | Multiple sclerosis with bladder dysfunction | United States of America (English) | Community dwelling women attending routine clinic visits at the University of Pittsburgh Medical Center’s outpatient Urology Centre, local multiple sclerosis support groups, and educational health-related programs | 57/65 (88%) returned both U-UDI questionnaires  49/65 (75%) included in test-retest analyses |

ALS: amyotrophic lateral sclerosis; CHF: congestive heart failure; COPD: chronic obstructive pulmonary disease; EGD: esophagogastroduodenoscopy; ESRD: end-stage renal disease; FEV: forced expiratory volume; GP: general practitioner; GSM-SVTAQ: Genitourinary Syndrome of Menopause (GSM) Symptoms and Vaginal Treatments Acceptability Questionnaire; HIV: human immunodeficiency virus; MQOL: McGill Quality of life Questionnaire; MUDI: Male Urinary Distress Inventory; MYMOP: Measure Yourself Medical Outcome Profile; NMF2S: Non-Motor Fluctuations Severity Scale; NR: not reported; OAB: overactive bladder; POSQ: Primary OAB Symptom Questionnaire; PSYCHLOPS: Psychological Outcome Profiles; QUAL-E: Quality-of-Life at the End of Life; QUAL-EC: Quality-of-Life at the End of Life-Cancer; SF-LDQ: Short-Form Leeds Dyspepsia Questionnaire; UDI: Urogenital Distress Inventory – long form; UI: urinary incontinence; U-UDI: Urge Urinary Distress Inventory; y: years; ^a^ mean and range; ^b^ mean and standard error; ^c^ median and range; ^d^ range; ^e^ median and interquartile range; ^f^ percentage of sample; † Study did not involve participants from target population; ‡ Study includes pooled data from five previously reported studies (Alves, 2016; Héðinsson et al., 2012; Czachowski et al., 2011; Ashworth, Robinson, Godfrey, Shepherd, et al., 2005; Ashworth et al., 2009); * See studies for individual information
