# Supplementary material for: How is the “most bothersome symptom” construct measured across health conditions? A systematic review of patient-reported outcome measures
Source: Patient Relat Outcome Meas. 2026 Sep 20;17(1):107–22. doi: 10.1080/1179271X.2026.2732841 (PMC13596887; doi:10.1080/1179271X.2026.2732841)
Supplement: Supplementary file 4 [file DPRO_A_2732841_SM9473.docx]

**Appendix 4: Risk of bias ratings**

| GSM-SVTAQ | | | | | |
| --- | --- | --- | --- | --- | --- |
| COSMIN Risk of Bias Checklist | | | Hassanein et al., 2024 | | |
|  | Item | Requirements | R1 | R2 | **C** |
| Box 1. PROM Development | | | | | |
| 1a. PROM design | | | | | |
|  | 1 | Is a clear description provided of the construct to be measured? | VG | VG | **VG** |
|  | 2 | Is the origin of the construct clear: was a theory, conceptual framework or disease model used or clear rationale provided to define the construct to be measured? | VG | VG | **VG** |
|  | 3 | Is a clear description provided of the target population for which the PROM was developed? | VG | VG | **VG** |
|  | 4 | Is a clear description provided of the context of use | VG | VG | **VG** |
|  | 5 | Was the PROM development study performed in a sample representing the target population for which the PROM was developed? | VG | VG | **VG** |
|  | 6 | Was an appropriate qualitative data collection method used to identify relevant items for a new PROM? | VG | VG | **VG** |
|  | 7 | Were skilled group moderators/interviewers used? | VG | VG | **VG** |
|  | 8 | Were the group meetings or interviews based on an appropriate topic or interview guide? | A | A | **A** |
|  | 9 | Were the group meetings or interviews recorded and transcribed verbatim? | A | A | **A** |
|  | 10 | Was an appropriate approach used to analyse the data? | VG | VG | **VG** |
|  | 11 | Was at least part of the data coded independently? | VG | VG | **VG** |
|  | 12 | Was data collection continued until saturation was reached? | A | A | **A** |
|  | 13 | For quantitative studies (surveys): was the sample size appropriate? | N/A | N/A | **N/A** |
| 1b. Cognitive interview study or pilot test | | | | | |
|  | 14 | Was a cognitive interview study or other pilot test conducted? | VG | VG | **VG** |
| General design requirements | 15 | Was the cognitive interview study or other pilot test performed in a sample representing the target population? | VG | VG | **VG** |
| Comprehensibility | 16 | Were patients asked about the comprehensibility of the PROM? | VG | VG | **VG** |
|  | 17 | Were all items tested in their final form? | A | A | **A** |
|  | 18 | Was an appropriate qualitative method used to assess the comprehensibility of the PROM instructions, items, response options, and recall period? | VG | VG | **VG** |
|  | 19 | Was each item tested in an appropriate number of patients? | VG | VG | **VG** |
|  |  | For qualitative studies | VG | VG | **VG** |
|  |  | For quantitative (survey) studies | VG | VG | **VG** |
|  | 20 | Were skilled interviewers used? | VG | VG | **VG** |
|  | 21 | Were the interviews based on an appropriate interview guide? | A | A | **A** |
|  | 22 | Were the interviews recorded and transcribed verbatim? | A | A | **A** |
|  | 23 | Was an appropriate approach used to analyse the data? | VG | VG | **VG** |
|  | 24 | Were at least two researchers involved in the analysis? | VG | VG | **VG** |
|  | 25 | Were problems regarding the comprehensibility of the PROM instructions, items, response options, and recall period appropriately addressed by adapting the PROM? | A | A | **A** |
| Comprehensiveness | 26 | Were patients asked about the comprehensiveness of the PROM? | VG | VG | **VG** |
|  | 27 | Was the final set of items tested? | A | A | **A** |
|  | 28 | Was an appropriate method used for assessing the comprehensiveness of the PROM? | VG | VG | **VG** |
|  | 29 | Was each item tested in an appropriate number of patients? | VG | VG | **VG** |
|  |  | For qualitative studies | VG | VG | **VG** |
|  |  | For quantitative (survey) studies | VG | VG | **VG** |
|  | 30 | Were skilled interviewers used? | VG | VG | **VG** |
|  | 31 | Were the interviews based on an appropriate interview guide? | A | A | **A** |
|  | 32 | Were the interviews recorded and transcribed verbatim? | A | A | **A** |
|  | 33 | Was an appropriate approach used to analyse the data? | VG | VG | **VG** |
|  | 34 | Were at least two researchers involved in the analysis? | A | A | **A** |
|  | 35 | Were problems regarding the comprehensiveness of the PROM appropriately addressed by adapting the PROM? | A | A | **A** |
| CONSENSUS | | |  |  | **A** |
| Box 2. Content validity | | | | | |
| 2a. Asking patients about relevance | | | | | |
| Design requirements | 1 | Was an appropriate method used to ask patients whether each item is relevant for their experience with the condition? | A | A | **A** |
|  | 2 | Was each item tested in an appropriate number of patients? |  |  |  |
|  |  | For qualitative studies |  |  |  |
|  |  | For quantitative (survey) studies | D | D | **D** |
|  | 3 | Were skilled group moderators/interviewers used? | N/A | N/A | **N/A** |
|  | 4 | Were the group meetings or interviews based on an appropriate topic or interview guide? | N/A | N/A | **N/A** |
|  | 5 | Were the group meetings or interviews recorded and transcribed verbatim? | N/A | N/A | **N/A** |
| Analyses | 6 | Was an appropriate approach used to analyse the data? | D | D | **D** |
|  | 7 | Were at least two researchers involved in the analysis? | D | D | **D** |
| 2b. Asking patients about comprehensiveness | | | | | |
| Design requirements | 8 | Was an appropriate method used for assessing the comprehensiveness of the PROM? | A | A | **A** |
|  | 9 | Was each item tested in an appropriate number of patients? |  |  |  |
|  |  | For qualitative studies |  |  |  |
|  |  | For quantitative (survey) studies | D | D | **D** |
|  | 10 | Were skilled group moderators/interviewers used? | N/A | N/A | **N/A** |
|  | 11 | Were the group meetings or interviews based on an appropriate topic or interview guide? | N/A | N/A | **N/A** |
|  | 12 | Were the group meetings or interviews recorded and transcribed verbatim? | N/A | N/A | **N/A** |
| Analyses | 13 | Was an appropriate approach used to analyse the data? | D | D | **D** |
|  | 14 | Were at least two researchers involved in the analysis? | D | D | **D** |
| 2c. Asking patients about comprehensibility | | | | | |
| Design requirements | 15 | Was an appropriate qualitative method used for assessing the comprehensibility of the PROM instructions, items, response options, and recall period? | D | D | **D** |
|  | 16 | Was each item tested in an appropriate number of patients? |  |  |  |
|  |  | For qualitative studies |  |  |  |
|  |  | For quantitative (survey) studies | D | D | **D** |
|  | 17 | Were skilled group moderators/interviewers used? |  |  |  |
|  | 18 | Were the group meetings or interviews based on an appropriate topic or interview guide? | N/A | N/A | **N/A** |
|  | 19 | Were the group meetings or interviews recorded and transcribed verbatim? | N/A | N/A | **N/A** |
| Analyses | 20 | Was an appropriate approach used to analyse the data? | D | D | **D** |
|  | 21 | Were at least two researchers involved in the analysis? | D | D | **D** |
| 2d. Asking professionals about relevance | | | | | |
| Design requirements | 22 | Was an appropriate method used to ask professionals whether each item is relevant for the construct of interest? | VG | VG | **VG** |
|  | 23 | Were professionals from all relevant disciplines included? | A | A | **A** |
|  | 24 | Was each item tested in an appropriate number of professionals? |  |  |  |
|  |  | For qualitative studies |  |  |  |
|  |  | For quantitative (survey) studies | D | D | **D** |
| Analyses | 25 | Was an appropriate approach used to analyse the data? | VG | VG | **VG** |
|  | 26 | Were at least two researchers involved in the analysis? | A | A | **A** |
| 2e. Asking professionals about comprehensiveness | | | | | |
| Design requirements | 27 | Was an appropriate method used for assessing the comprehensiveness of the PROM? | VG | VG | **VG** |
|  | 28 | Were professionals from all relevant disciplines included? | A | A | **A** |
|  | 29 | Was each item tested in an appropriate number of professionals? |  |  |  |
|  |  | For qualitative studies |  |  |  |
|  |  | For quantitative (survey) studies | D | D | **D** |
| Analyses | 30 | Was an appropriate approach used to analyse the data? | VG | VG | **VG** |
|  | 31 | Were at least two researchers involved in the analysis? | A | A | **A** |
| CONSENSUS | | |  |  | **D** |
| Box 4. Internal consistency | | | | | |
|  |  | Does the scale consist of effect indicators, i.e. is it based on a reflective model? | Y | Y | **Y** |
| Design requirements | 1 | Was an internal consistency statistic calculated for each unidimensional scale or subscale separately? | VG | VG | **VG** |
| Statistical methods | 2 | For continuous scores: Was Cronbach’s alpha or omega calculated? | VG | VG | **VG** |
|  | 3 | For dichotomous scores: Was Cronbach’s alpha or KR-20 calculated? | N/A | N/A | **N/A** |
|  | 4 | For IRT-based scores: Was standard error of the theta (SE (θ)) or reliability coefficient of estimated latent trait value (index of (subject or item) separation) calculated? | N/A | N/A | **N/A** |
| Other | 5 | Were there any other important flaws in the design or statistical methods of the study? | VG | VG | **VG** |
| CONSENSUS | | |  |  | **VG** |
| % | | | 100% | | |

A: adequate; C: consensus; D: doubtful; Genitourinary Syndrome of Menopause Symptoms and Vaginal Treatments Acceptability Questionnaire (GSM-SVTAQ); N/A: not applicable; R1: Reviewer 1; R2: Reviewer 2; VG: very good; Y: yes

| MQOL – PART 1 | | | | | | | | | | | | | | | | | | | | | | | | | | | | | | | | |
| --- | --- | --- | --- | --- | --- | --- | --- | --- | --- | --- | --- | --- | --- | --- | --- | --- | --- | --- | --- | --- | --- | --- | --- | --- | --- | --- | --- | --- | --- | --- | --- | --- |
| COSMIN Risk of Bias Checklist | | | Cohen et al., 1995 | | | Cohen, Mount, Tomas & Mount, 1996 | | | Cohen, Hassen, Lapointe & Mount, 1996 | | | Cohen et al., 1997 | | | Cohen & Mount, 2000 | | | Lo et al., 2001 | | | Paratheepawanit et al., 2001 | | | Tolentino & Sulmasy, 2002 | | | Zhang et al., 2023 | | | Hu et al., 2003 | | |
|  | Item | Requirement | R1 | R2 | **C** | R1 | R2 | **C** | R1 | R2 | **C** | R1 | R2 | **C** | R1 | R2 | **C** | R1 | R2 | **C** | R1 | R2 | **C** | R1 | R2 | **C** | R1 | R2 | **C** | R1 | R2 | **C** |
| Box 1. PROM Development | | | | | | | | | | | | | | | | | | | | | | | | | | | | | | | | |
| 1a. PROM design | | | | | | | | | | | | | | | | | | | | | | | | | | | | | | | | |
|  | 1 | Is a clear description provided of the construct to be measured? | VG | VG | **VG** |  |  |  |  |  |  |  |  |  |  |  |  |  |  |  |  |  |  |  |  |  |  |  |  |  |  |  |
|  | 2 | Is the origin of the construct clear: was a theory, conceptual framework or disease model used or clear rationale provided to define the construct to be measured? | VG | VG | **VG** |  |  |  |  |  |  |  |  |  |  |  |  |  |  |  |  |  |  |  |  |  |  |  |  |  |  |  |
|  | 3 | Is a clear description provided of the target population for which the PROM was developed? | VG | VG | **VG** |  |  |  |  |  |  |  |  |  |  |  |  |  |  |  |  |  |  |  |  |  |  |  |  |  |  |  |
|  | 4 | Is a clear description provided of the context of use | VG | VG | **VG** |  |  |  |  |  |  |  |  |  |  |  |  |  |  |  |  |  |  |  |  |  |  |  |  |  |  |  |
|  | 5 | Was the PROM development study performed in a sample representing the target population for which the PROM was developed? | A | A | **A** |  |  |  |  |  |  |  |  |  |  |  |  |  |  |  |  |  |  |  |  |  |  |  |  |  |  |  |
|  | 6 | Was an appropriate qualitative data collection method used to identify relevant items for a new PROM? | D | D | **D** |  |  |  |  |  |  |  |  |  |  |  |  |  |  |  |  |  |  |  |  |  |  |  |  |  |  |  |
|  | 7 | Were skilled group moderators/interviewers used? | N/A | N/A | **N/A** |  |  |  |  |  |  |  |  |  |  |  |  |  |  |  |  |  |  |  |  |  |  |  |  |  |  |  |
|  | 8 | Were the group meetings or interviews based on an appropriate topic or interview guide? | N/A | N/A | **N/A** |  |  |  |  |  |  |  |  |  |  |  |  |  |  |  |  |  |  |  |  |  |  |  |  |  |  |  |
|  | 9 | Were the group meetings or interviews recorded and transcribed verbatim? | N/A | N/A | **N/A** |  |  |  |  |  |  |  |  |  |  |  |  |  |  |  |  |  |  |  |  |  |  |  |  |  |  |  |
|  | 10 | Was an appropriate approach used to analyse the data? | D | D | **D** |  |  |  |  |  |  |  |  |  |  |  |  |  |  |  |  |  |  |  |  |  |  |  |  |  |  |  |
|  | 11 | Was at least part of the data coded independently? | N/A | N/A | **N/A** |  |  |  |  |  |  |  |  |  |  |  |  |  |  |  |  |  |  |  |  |  |  |  |  |  |  |  |
|  | 12 | Was data collection continued until saturation was reached? | N/A | N/A | **N/A** |  |  |  |  |  |  |  |  |  |  |  |  |  |  |  |  |  |  |  |  |  |  |  |  |  |  |  |
|  | 13 | For quantitative studies (surveys): was the sample size appropriate? | A | A | **A** |  |  |  |  |  |  |  |  |  |  |  |  |  |  |  |  |  |  |  |  |  |  |  |  |  |  |  |
| 1b. Cognitive interview study or pilot test | | | | | | | | | | | | | | | | | | | | | | | | | | | | | | | | |
|  | 14 | Was a cognitive interview study or other pilot test conducted? | I | I | **I** |  |  |  |  |  |  |  |  |  |  |  |  |  |  |  |  |  |  |  |  |  |  |  |  |  |  |  |
| General design requirements | 15 | Was the cognitive interview study or other pilot test performed in a sample representing the target population? |  |  |  |  |  |  |  |  |  |  |  |  |  |  |  |  |  |  |  |  |  |  |  |  |  |  |  |  |  |  |
| Comprehensibility | 16 | Were patients asked about the comprehensibility of the PROM? |  |  |  |  |  |  |  |  |  |  |  |  |  |  |  |  |  |  |  |  |  |  |  |  |  |  |  |  |  |  |
|  | 17 | Were all items tested in their final form? |  |  |  |  |  |  |  |  |  |  |  |  |  |  |  |  |  |  |  |  |  |  |  |  |  |  |  |  |  |  |
|  | 18 | Was an appropriate qualitative method used to assess the comprehensibility of the PROM instructions, items, response options, and recall period? |  |  |  |  |  |  |  |  |  |  |  |  |  |  |  |  |  |  |  |  |  |  |  |  |  |  |  |  |  |  |
|  | 19 | Was each item tested in an appropriate number of patients? |  |  |  |  |  |  |  |  |  |  |  |  |  |  |  |  |  |  |  |  |  |  |  |  |  |  |  |  |  |  |
|  |  | For qualitative studies |  |  |  |  |  |  |  |  |  |  |  |  |  |  |  |  |  |  |  |  |  |  |  |  |  |  |  |  |  |  |
|  |  | For quantitative (survey) studies |  |  |  |  |  |  |  |  |  |  |  |  |  |  |  |  |  |  |  |  |  |  |  |  |  |  |  |  |  |  |
|  | 20 | Were skilled interviewers used? |  |  |  |  |  |  |  |  |  |  |  |  |  |  |  |  |  |  |  |  |  |  |  |  |  |  |  |  |  |  |
|  | 21 | Were the interviews based on an appropriate interview guide? |  |  |  |  |  |  |  |  |  |  |  |  |  |  |  |  |  |  |  |  |  |  |  |  |  |  |  |  |  |  |
|  | 22 | Were the interviews recorded and transcribed verbatim? |  |  |  |  |  |  |  |  |  |  |  |  |  |  |  |  |  |  |  |  |  |  |  |  |  |  |  |  |  |  |
|  | 23 | Was an appropriate approach used to analyse the data? |  |  |  |  |  |  |  |  |  |  |  |  |  |  |  |  |  |  |  |  |  |  |  |  |  |  |  |  |  |  |
|  | 24 | Were at least two researchers involved in the analysis? |  |  |  |  |  |  |  |  |  |  |  |  |  |  |  |  |  |  |  |  |  |  |  |  |  |  |  |  |  |  |
|  | 25 | Were problems regarding the comprehensibility of the PROM instructions, items, response options, and recall period appropriately addressed by adapting the PROM? |  |  |  |  |  |  |  |  |  |  |  |  |  |  |  |  |  |  |  |  |  |  |  |  |  |  |  |  |  |  |
| Comprehensiveness | 26 | Were patients asked about the comprehensiveness of the PROM? |  |  |  |  |  |  |  |  |  |  |  |  |  |  |  |  |  |  |  |  |  |  |  |  |  |  |  |  |  |  |
|  | 27 | Was the final set of items tested? |  |  |  |  |  |  |  |  |  |  |  |  |  |  |  |  |  |  |  |  |  |  |  |  |  |  |  |  |  |  |
|  | 28 | Was an appropriate method used for assessing the comprehensiveness of the PROM? |  |  |  |  |  |  |  |  |  |  |  |  |  |  |  |  |  |  |  |  |  |  |  |  |  |  |  |  |  |  |
|  | 29 | Was each item tested in an appropriate number of patients? |  |  |  |  |  |  |  |  |  |  |  |  |  |  |  |  |  |  |  |  |  |  |  |  |  |  |  |  |  |  |
|  |  | For qualitative studies |  |  |  |  |  |  |  |  |  |  |  |  |  |  |  |  |  |  |  |  |  |  |  |  |  |  |  |  |  |  |
|  |  | For quantitative (survey) studies |  |  |  |  |  |  |  |  |  |  |  |  |  |  |  |  |  |  |  |  |  |  |  |  |  |  |  |  |  |  |
|  | 30 | Were skilled interviewers used? |  |  |  |  |  |  |  |  |  |  |  |  |  |  |  |  |  |  |  |  |  |  |  |  |  |  |  |  |  |  |
|  | 31 | Were the interviews based on an appropriate interview guide? |  |  |  |  |  |  |  |  |  |  |  |  |  |  |  |  |  |  |  |  |  |  |  |  |  |  |  |  |  |  |
|  | 32 | Were the interviews recorded and transcribed verbatim? |  |  |  |  |  |  |  |  |  |  |  |  |  |  |  |  |  |  |  |  |  |  |  |  |  |  |  |  |  |  |
|  | 33 | Was an appropriate approach used to analyse the data? |  |  |  |  |  |  |  |  |  |  |  |  |  |  |  |  |  |  |  |  |  |  |  |  |  |  |  |  |  |  |
|  | 34 | Were at least two researchers involved in the analysis? |  |  |  |  |  |  |  |  |  |  |  |  |  |  |  |  |  |  |  |  |  |  |  |  |  |  |  |  |  |  |
|  | 35 | Were problems regarding the comprehensiveness of the PROM appropriately addressed by adapting the PROM? |  |  |  |  |  |  |  |  |  |  |  |  |  |  |  |  |  |  |  |  |  |  |  |  |  |  |  |  |  |  |
| CONSENSUS |  |  |  |  | **I** |  |  |  |  |  |  |  |  |  |  |  |  |  |  |  |  |  |  |  |  |  |  |  |  |  |  |  |
| Box 2. Content validity | | | | | | | | | | | | | | | | | | | | | | | | | | | | | | | | |
| 2a. Asking patients about relevance | | | | | | | | | | | | | | | | | | | | | | | | | | | | | | | | |
| Design requirements | 1 | Was an appropriate method used to ask patients whether each item is relevant for their experience with the condition? |  |  |  |  |  |  |  |  |  |  |  |  |  |  |  |  |  |  | VG | VG | **VG** |  |  |  |  |  |  | A |  | **A** |
|  | 2 | Was each item tested in an appropriate number of patients? |  |  |  |  |  |  |  |  |  |  |  |  |  |  |  |  |  |  |  |  |  |  |  |  |  |  |  |  |  |  |
|  |  | For qualitative studies |  |  |  |  |  |  |  |  |  |  |  |  |  |  |  |  |  |  | VG | VG | **VG** |  |  |  |  |  |  |  |  |  |
|  |  | For quantitative (survey) studies |  |  |  |  |  |  |  |  |  |  |  |  |  |  |  |  |  |  |  |  |  |  |  |  |  |  |  | VG | VG | **VG** |
|  | 3 | Were skilled group moderators/interviewers used? |  |  |  |  |  |  |  |  |  |  |  |  |  |  |  |  |  |  | A | A | **A** |  |  |  |  |  |  | N/A | N/A | **N/A** |
|  | 4 | Were the group meetings or interviews based on an appropriate topic or interview guide? |  |  |  |  |  |  |  |  |  |  |  |  |  |  |  |  |  |  | D | D | **D** |  |  |  |  |  |  | N/A | N/A | **N/A** |
|  | 5 | Were the group meetings or interviews recorded and transcribed verbatim? |  |  |  |  |  |  |  |  |  |  |  |  |  |  |  |  |  |  | D | D | **D** |  |  |  |  |  |  | N/A | N/A | **N/A** |
| Analyses | 6 | Was an appropriate approach used to analyse the data? |  |  |  |  |  |  |  |  |  |  |  |  |  |  |  |  |  |  | D | D | **D** |  |  |  |  |  |  | VG | VG | **VG** |
|  | 7 | Were at least two researchers involved in the analysis? |  |  |  |  |  |  |  |  |  |  |  |  |  |  |  |  |  |  | D | A | **D** |  |  |  |  |  |  | D | D | **D** |
| 2b. Asking patients about comprehensiveness | | | | | | | | | | | | | | | | | | | | | | | | | | | | | | | | |
| Design requirements | 8 | Was an appropriate method used for assessing the comprehensiveness of the PROM? |  |  |  | A | A | **A** | A | A | **A** |  |  |  |  |  |  |  |  |  |  |  |  |  |  |  |  |  |  |  |  |  |
|  | 9 | Was each item tested in an appropriate number of patients? |  |  |  |  |  |  |  |  |  |  |  |  |  |  |  |  |  |  |  |  |  |  |  |  |  |  |  |  |  |  |
|  |  | For qualitative studies |  |  |  |  |  |  |  |  |  |  |  |  |  |  |  |  |  |  |  |  |  |  |  |  |  |  |  |  |  |  |
|  |  | For quantitative (survey) studies |  |  |  | VG | VG | **VG** | VG | VG | **VG** |  |  |  |  |  |  |  |  |  |  |  |  |  |  |  |  |  |  |  |  |  |
|  | 10 | Were skilled group moderators/interviewers used? |  |  |  | N/A | N/A | **N/A** | N/A | N/A | **N/A** |  |  |  |  |  |  |  |  |  |  |  |  |  |  |  |  |  |  |  |  |  |
|  | 11 | Were the group meetings or interviews based on an appropriate topic or interview guide? |  |  |  | N/A | N/A | **N/A** | N/A | N/A | **N/A** |  |  |  |  |  |  |  |  |  |  |  |  |  |  |  |  |  |  |  |  |  |
|  | 12 | Were the group meetings or interviews recorded and transcribed verbatim? |  |  |  | D | N/A | **N/A** | N/A | N/A | **N/A** |  |  |  |  |  |  |  |  |  |  |  |  |  |  |  |  |  |  |  |  |  |
| Analyses | 13 | Was an appropriate approach used to analyse the data? |  |  |  | D | D | **D** | D | D | **D** |  |  |  |  |  |  |  |  |  |  |  |  |  |  |  |  |  |  |  |  |  |
|  | 14 | Were at least two researchers involved in the analysis? |  |  |  | D | D | **D** | D | D | **D** |  |  |  |  |  |  |  |  |  |  |  |  |  |  |  |  |  |  |  |  |  |
| 2c. Asking patients about comprehensibility | | | | | | | | | | | | | | | | | | | | | | | | | | | | | | | | |
| Design requirements | 15 | Was an appropriate qualitative method used for assessing the comprehensibility of the PROM instructions, items, response options, and recall period? |  |  |  | D | D | **D** | D | D | **D** |  |  |  |  |  |  |  |  |  | VG | VG | **VG** |  |  |  | VG |  | **A** |  |  |  |
|  | 16 | Was each item tested in an appropriate number of patients? |  |  |  |  |  |  |  |  |  |  |  |  |  |  |  |  |  |  |  |  |  |  |  |  |  |  |  |  |  |  |
|  |  | For qualitative studies |  |  |  |  |  |  |  |  |  |  |  |  |  |  |  |  |  |  | VG | VG | **VG** |  |  |  | VG | VG | **VG** |  |  |  |
|  |  | For quantitative (survey) studies |  |  |  | VG | VG | **VG** | VG | VG | **VG** |  |  |  |  |  |  |  |  |  |  |  |  |  |  |  |  |  |  |  |  |  |
|  | 17 | Were skilled group moderators/interviewers used? |  |  |  | D |  | **N/A** | D | D | **D** |  |  |  |  |  |  |  |  |  | A | A | **A** |  |  |  | D | D | **D** |  |  |  |
|  | 18 | Were the group meetings or interviews based on an appropriate topic or interview guide? |  |  |  | N/A | N/A | **N/A** | N/A | N/A | **N/A** |  |  |  |  |  |  |  |  |  | D | D | **D** |  |  |  | D | D | **D** |  |  |  |
|  | 19 | Were the group meetings or interviews recorded and transcribed verbatim? |  |  |  | D | N/A | **N/A** | N/A | N/A | **N/A** |  |  |  |  |  |  |  |  |  | D | D | **D** |  |  |  | D | D | **D** |  |  |  |
| Analyses | 20 | Was an appropriate approach used to analyse the data? |  |  |  | D | D | **D** | D | D | **D** |  |  |  |  |  |  |  |  |  | D | D | **D** |  |  |  | D | D | **D** |  |  |  |
|  | 21 | Were at least two researchers involved in the analysis? |  |  |  | D | D | **D** | D | D | **D** |  |  |  |  |  |  |  |  |  | D | D | **D** |  |  |  | D | D | **D** |  |  |  |
| 2d. Asking professionals about relevance | | | | | | | | | | | | | | | | | | | | | | | | | | | | | | | | |
| Design requirements | 22 | Was an appropriate method used to ask professionals whether each item is relevant for the construct of interest? |  |  |  |  |  |  |  |  |  |  |  |  |  |  |  |  |  |  |  |  |  |  | D |  | VG | A | **A** |  |  |  |
|  | 23 | Were professionals from all relevant disciplines included? |  |  |  |  |  |  |  |  |  |  |  |  |  |  |  |  |  |  |  |  |  |  | D |  | VG | VG | **VG** |  |  |  |
|  | 24 | Was each item tested in an appropriate number of professionals? |  |  |  |  |  |  |  |  |  |  |  |  |  |  |  |  |  |  |  |  |  |  |  |  |  |  |  |  |  |  |
|  |  | For qualitative studies |  |  |  |  |  |  |  |  |  |  |  |  |  |  |  |  |  |  |  |  |  |  | A |  | VG | VG | **VG** |  |  |  |
|  |  | For quantitative (survey) studies |  |  |  |  |  |  |  |  |  |  |  |  |  |  |  |  |  |  |  |  |  |  |  |  |  |  |  |  |  |  |
| Analyses | 25 | Was an appropriate approach used to analyse the data? |  |  |  |  |  |  |  |  |  |  |  |  |  |  |  |  |  |  |  |  |  |  | D |  | VG | VG | **VG** |  |  |  |
|  | 26 | Were at least two researchers involved in the analysis? |  |  |  |  |  |  |  |  |  |  |  |  |  |  |  |  |  |  |  |  |  |  | D |  | VG | VG | **VG** |  |  |  |
| 2e. Asking professionals about comprehensiveness | | | | | | | | | | | | | | | | | | | | | | | | | | | | | | | | |
| Design requirements | 27 | Was an appropriate method used for assessing the comprehensiveness of the PROM? |  |  |  |  |  |  |  |  |  |  |  |  |  |  |  |  |  |  |  |  |  |  |  |  |  |  |  |  |  |  |
|  | 28 | Were professionals from all relevant disciplines included? |  |  |  |  |  |  |  |  |  |  |  |  |  |  |  |  |  |  |  |  |  |  |  |  |  |  |  |  |  |  |
|  | 29 | Was each item tested in an appropriate number of professionals? |  |  |  |  |  |  |  |  |  |  |  |  |  |  |  |  |  |  |  |  |  |  |  |  |  |  |  |  |  |  |
|  |  | For qualitative studies |  |  |  |  |  |  |  |  |  |  |  |  |  |  |  |  |  |  |  |  |  |  |  |  |  |  |  |  |  |  |
|  |  | For quantitative (survey) studies |  |  |  |  |  |  |  |  |  |  |  |  |  |  |  |  |  |  |  |  |  |  |  |  |  |  |  |  |  |  |
| Analyses | 30 | Was an appropriate approach used to analyse the data? |  |  |  |  |  |  |  |  |  |  |  |  |  |  |  |  |  |  |  |  |  |  |  |  |  |  |  |  |  |  |
|  | 31 | Were at least two researchers involved in the analysis? |  |  |  |  |  |  |  |  |  |  |  |  |  |  |  |  |  |  |  |  |  |  |  |  |  |  |  |  |  |  |
| CONSENSUSUS | | |  |  |  |  |  | **D** |  |  | **D** |  |  |  |  |  |  |  |  |  |  |  | **D** |  |  |  |  |  | **D** |  |  | **D** |
| Box 3. Structural validity | | | | | | | | | | | | | | | | | | | | | | | | | | | | | | | | |
|  |  | Does the scale consist of effect indicators, i.e. is it based on a reflective model? | Y | Y | **Y** | Y | Y | **Y** | Y | Y | **Y** | Y | Y | **Y** |  |  |  | Y | Y | **Y** |  |  |  |  |  |  | Y | Y | **Y** | Y | Y | **Y** |
|  |  | Does the study concern unidimensionality or structural validity? | SV | SV | **SV** | SV | SV | **SV** | SV | SV | **SV** | SV | SV | **SV** |  |  |  | SV | SV | **SV** |  |  |  |  |  |  | SV | SV | **SV** | SV | SV | **SV** |
| Statistical methods | 1 | For CTT: Was exploratory or confirmatory factor analysis performed? | A | VG | **A** | A | A | **A** | A | A | **A** | A | A | **A** |  |  |  | A | A | **A** |  |  |  |  |  |  | A | A | **A** | A | A | **A** |
|  | 2 | For IRT/Rasch: does the chosen model fit to the research question? | N/A |  | **N/A** | N/A | I | **N/A** | N/A | N/A | **N/A** | N/A | N/A | **N/A** |  |  |  | N/A | N/A | **N/A** |  |  |  |  |  |  | N/A | N/A | **N/A** | N/A | N/A | **N/A** |
|  | 3 | Was the sample size included in the analysis A? |  |  |  |  |  |  |  |  |  |  |  |  |  |  |  |  |  |  |  |  |  |  |  |  |  |  |  |  |  |  |
|  |  | For FA | I | I | **I** | VG | VG | **VG** | A | A | **A** | A | VG | **VG** |  |  |  | VG | VG | **VG** |  |  |  |  |  |  | VG | VG | **VG** | I | I | **I** |
|  |  | For Rasch/1PL models |  |  |  |  |  |  |  |  |  |  |  |  |  |  |  |  |  |  |  |  |  |  |  |  |  |  |  |  |  |  |
|  |  | For 2PL parametric IRT models OR Mokken scale analysis |  |  |  |  |  |  |  |  |  |  |  |  |  |  |  |  |  |  |  |  |  |  |  |  |  |  |  |  |  |  |
| Other | 4 | Were there any other important flaws in the design or statistical methods of the study? | VG | VG | **VG** | VG | VG | **VG** | VG | VG | **VG** | VG | VG | **VG** |  |  |  | VG | VG | **VG** |  |  |  |  |  |  | VG | VG | **VG** | VG | VG | **VG** |
| CONSENSUS |  |  |  |  | **I** |  |  | **A** |  |  | **A** |  |  | **A** |  |  |  |  |  | **A** |  |  |  |  |  |  |  |  | **A** |  |  | **I** |
| Box 4. Internal consistency | | | | | | | | | | | | | | | | | | | | | | | | | | | | | | | | |
|  |  | Does the scale consist of effect indicators, i.e. is it based on a reflective model? | Y | Y | **Y** | Y | Y | **Y** | Y | Y | **Y** | Y | N | **Y** | Y | Y | **Y** | Y | Y | **Y** | Y | Y | **Y** |  |  |  | Y | N | **Y** | Y | Y | **Y** |
| Design requirements | 1 | Was an internal consistency statistic calculated for each unidimensional scale or subscale separately? | VG | VG | **VG** | VG | VG | **VG** | VG | VG | **VG** | VG | VG | **VG** | VG | VG | **VG** | VG | VG | **VG** | VG | VG | **VG** |  |  |  | VG | VG | **VG** | VG | VG | **VG** |
| Statistical methods | 2 | For continuous scores: Was Cronbach’s alpha or omega calculated? | VG | VG | **VG** | VG | VG | **VG** | VG | VG | **VG** | VG | VG | **VG** | VG | VG | **VG** | VG | VG | **VG** | VG | VG | **VG** |  |  |  | VG | VG | **VG** | VG | VG | **VG** |
|  | 3 | For dichotomous scores: Was Cronbach’s alpha or KR-20 calculated? | N/A | N/A | **N/A** | N/A | N/A | **N/A** | N/A | N/A | **N/A** | N/A | N/A | **N/A** | N/A | N/A | **N/A** | N/A | N/A | **N/A** | N/A | N/A | **N/A** |  |  |  | N/A | N/A | **N/A** | N/A | N/A | **N/A** |
|  | 4 | For IRT-based scores: Was standard error of the theta (SE (θ)) or reliability coefficient of estimated latent trait value (index of (subject or item) separation) calculated? | N/A | N/A | **N/A** | N/A | N/A | **N/A** | I | N/A | **N/A** | N/A | N/A | **N/A** | N/A | N/A | **N/A** | N/A | N/A | **N/A** | N/A | N/A | **N/A** |  |  |  | N/A | N/A | **N/A** | N/A | N/A | **N/A** |
| Other | 5 | Were there any other important flaws in the design or statistical methods of the study? | VG | VG | **VG** | VG | VG | **VG** | VG | VG | **VG** | VG | VG | **VG** | VG | VG | **VG** | VG | VG | **VG** | VG | VG | **VG** |  |  |  | VG | VG | **VG** | VG | VG | **VG** |
| CONSENSUS | | |  |  | **VG** |  |  | **VG** |  |  | **VG** |  |  | **VG** |  |  | **VG** |  |  | **VG** |  |  | **VG** |  |  |  |  |  | **VG** |  |  | **VG** |
| Box 5. Cross-cultural validity/Measurement invariance | | | | | | | | | | | | | | | | | | | | | | | | | | | | | | | | |
| Design requirements | 1 | Were the samples similar for relevant characteristics except for the group variable? |  |  |  | D |  |  |  |  |  | D |  |  | D |  |  |  |  |  |  |  |  |  |  |  |  |  |  |  |  |  |
| Statistical methods | 2 | Was an appropriate approach used to analyse the data? |  |  |  | VG |  |  |  |  |  | A |  |  | A |  |  |  |  |  |  |  |  |  |  |  |  |  |  |  |  |  |
|  | 3 | Was the sample size included in the analysis A? |  |  |  |  |  |  |  |  |  |  |  |  |  |  |  |  |  |  |  |  |  |  |  |  |  |  |  |  |  |  |
|  |  | For Regression analyses or IRT/Rasch based analyses |  |  |  | I |  |  |  |  |  | I |  |  | I |  |  |  |  |  |  |  |  |  |  |  |  |  |  |  |  |  |
|  |  | For MGCFA* |  |  |  |  |  |  |  |  |  |  |  |  |  |  |  |  |  |  |  |  |  |  |  |  |  |  |  |  |  |  |
| Other | 4 | Were there any other important flaws in the design or statistical methods of the study? |  |  |  | VG |  |  |  |  |  | VG |  |  | VG |  |  |  |  |  |  |  |  |  |  |  |  |  |  |  |  |  |
| CONSENSUS |  |  |  |  |  |  |  |  |  |  |  |  |  |  |  |  |  |  |  |  |  |  |  |  |  |  |  |  |  |  |  |  |
| Box 6. Reliability | | | | | | | | | | | | | | | | | | | | | | | | | | | | | | | | |
| Design requirements | 1 | Were patients stable in the interim period on the construct to be measured? |  |  |  |  |  |  |  |  |  |  |  |  | VG | VG | **VG** | D | D | **D** |  |  |  |  |  |  |  |  |  |  |  |  |
|  | 2 | Was the time interval appropriate? |  |  |  |  |  |  |  |  |  |  |  |  | D | VG | **A** | D | D | **D** |  |  |  |  |  |  |  |  |  |  |  |  |
|  | 3 | Were the test conditions similar for the measurements? e.g. type of administration, environment, instructions |  |  |  |  |  |  |  |  |  |  |  |  | VG | VG | **VG** | VG | A | **A** |  |  |  |  |  |  |  |  |  |  |  |  |
| Statistical methods | 4 | For continuous scores: Was an intraclass correlation coefficient (ICC) calculated? |  |  |  |  |  |  |  |  |  |  |  |  | A | A | **A** | A | A | **A** |  |  |  |  |  |  |  |  |  |  |  |  |
|  | 5 | For dichotomous/nominal/ordinal scores: Was kappa calculated? |  |  |  |  |  |  |  |  |  |  |  |  | N/A | N/A | **N/A** | N/A | N/A | **N/A** |  |  |  |  |  |  |  |  |  |  |  |  |
|  | 6 | For ordinal scores: Was a weighted kappa calculated? |  |  |  |  |  |  |  |  |  |  |  |  | N/A | N/A | **N/A** | N/A | D | **N/A** |  |  |  |  |  |  |  |  |  |  |  |  |
|  | 7 | For ordinal scores: Was the weighting scheme described? e.g. linear, quadratic |  |  |  |  |  |  |  |  |  |  |  |  | N/A | N/A | **N/A** | N/A | A | **N/A** |  |  |  |  |  |  |  |  |  |  |  |  |
| Other | 8 | Were there any other important flaws in the design or statistical methods of the study? |  |  |  |  |  |  |  |  |  |  |  |  | VG | VG | **VG** | VG | I | **VG** |  |  |  |  |  |  |  |  |  |  |  |  |
| CONSENSUS |  |  |  |  |  |  |  |  |  |  |  |  |  |  |  |  | **A** |  |  | **D** |  |  |  |  |  |  |  |  |  |  |  |  |
| Box 7. Measurement error | | | | | | | | | | | | | | | | | | | | | | | | | | | | | | | | |
| Design requirements | 1 | Were patients stable in the interim period on the construct to be measured? |  |  |  |  |  |  |  |  |  |  |  |  |  |  |  |  |  |  |  |  |  |  |  |  |  |  |  |  |  |  |
|  | 2 | Was the time interval appropriate? |  |  |  |  |  |  |  |  |  |  |  |  |  |  |  |  |  |  |  |  |  |  |  |  |  |  |  |  |  |  |
|  | 3 | Were the test conditions similar for the measurements? e.g. type of administration, environment, instructions |  |  |  |  |  |  |  |  |  |  |  |  |  |  |  |  |  |  |  |  |  |  |  |  |  |  |  |  |  |  |
| Statistical methods | 4 | For continuous scores: Was the Standard Error of Measurement (SEM), Smallest Detectable Change (SDC) or Limits of Agreement (LoA) calculated? |  |  |  |  |  |  |  |  |  |  |  |  |  |  |  |  |  |  |  |  |  |  |  |  |  |  |  |  |  |  |
|  | 5 | For dichotomous/nominal/ordinal scores: Was the percentage (positive and negative) agreement calculated? |  |  |  |  |  |  |  |  |  |  |  |  |  |  |  |  |  |  |  |  |  |  |  |  |  |  |  |  |  |  |
| Other | 6 | Were there any other important flaws in the design or statistical methods of the study? |  |  |  |  |  |  |  |  |  |  |  |  |  |  |  |  |  |  |  |  |  |  |  |  |  |  |  |  |  |  |
| CONSENSUS |  |  |  |  |  |  |  |  |  |  |  |  |  |  |  |  |  |  |  |  |  |  |  |  |  |  |  |  |  |  |  |  |
| Box 8. Criterion validity | | | | | | | | | | | | | | | | | | | | | | | | | | | | | | | | |
| Statistical methods | 1 | For continuous scores: Were correlations, or the area under the receiver operating curve calculated? |  |  |  |  |  |  |  |  |  |  |  |  |  |  |  |  |  |  |  |  |  |  |  |  |  |  |  |  |  |  |
|  | 2 | For dichotomous scores: Were sensitivity and specificity determined? |  |  |  |  |  |  |  |  |  |  |  |  |  |  |  |  |  |  |  |  |  |  |  |  |  |  |  |  |  |  |
| Other | 3 | Were there any other important flaws in the design or statistical methods of the study? |  |  |  |  |  |  |  |  |  |  |  |  |  |  |  |  |  |  |  |  |  |  |  |  |  |  |  |  |  |  |
| CONSENS |  |  |  |  |  |  |  |  |  |  |  |  |  |  |  |  |  |  |  |  |  |  |  |  |  |  |  |  |  |  |  |  |
| Box 9. Hypotheses testing for construct validity | | | | | | | | | | | | | | | | | | | | | | | | | | | | | | | | |
| 9a. Comparison with other outcome measurement instruments (convergent validity) | | | | | | | | | | | | | | | | | | | | | | | | | | | | | | | | |
| Design requirements | 1 | Is it clear what the comparator instrument(s) measure(s)? | VG | VG | **VG** | VG | VG | **VG** | VG | VG | **VG** | VG | VG | **VG** |  |  |  | VG | VG | **VG** | VG | VG | **VG** |  |  |  |  |  |  | VG | I | **VG** |
|  | 2 | Were the measurement properties of the comparator instrument(s) sufficient? | VG | VG | **VG** | VG | VG | **VG** | VG | VG | **VG** | VG | I | **VG** |  |  |  | A | VG | **VG** | VG | VG | **VG** |  |  |  |  |  |  | VG | VG | **VG** |
| Statistical methods | 3 | Was the statistical method appropriate for the hypotheses to be tested? | VG | VG | **VG** | VG | VG | **VG** | I | VG | **I** | VG | VG | **VG** |  |  |  | VG | VG | **VG** | VG | VG | **VG** |  |  |  |  |  |  | I | I | **I** |
| Other | 4 | Were there any other important flaws in the design or statistical methods of the study? | VG | VG | **VG** | VG | VG | **VG** | VG | VG | **VG** | VG | VG | **VG** |  |  |  | VG | VG | **VG** | VG | VG | **VG** |  |  |  |  |  |  | VG | VG | **VG** |
| CONSENSUS |  |  |  |  | **VG** |  |  | **VG** |  |  | **I** |  |  | **VG** |  |  |  |  |  | **VG** |  |  | **VG** |  |  |  |  |  |  |  |  | **I** |
| 9b. Comparison between subgroups (discriminative or known-groups validity) | | | | | | | | | | | | | | | | | | | | | | | | | | | | | | | | |
| Design requirements | 5 | Was an A description provided of important characteristics of the subgroups? |  |  |  | D | A | **A** | D | VG | **VG** |  |  |  |  |  |  |  |  |  | VG | D | **VG** |  |  |  |  |  |  |  |  |  |
| Statistical methods | 6 | Was the statistical method appropriate for the hypotheses to be tested? |  |  |  | VG | VG | **VG** | A | VG | **A** |  |  |  |  |  |  |  |  |  | VG | VG | **VG** |  |  |  |  |  |  |  |  |  |
| Other | 7 | Were there any other important flaws in the design or statistical methods of the study? |  |  |  | VG | VG | **VG** | VG | VG | **VG** |  |  |  |  |  |  |  |  |  | VG | VG | **VG** |  |  |  |  |  |  |  |  |  |
| CONSENSUS | | |  |  |  |  |  | **A** |  |  | **A** |  |  |  |  |  |  |  |  |  |  |  | **VG** |  |  |  |  |  |  |  |  |  |
| Box 10. Responsiveness | | | | | | | | | | | | | | | | | | | | | | | | | | | | | | | | |
| 10a. Criterion approach (i.e. comparison to a gold standard) | | | | | | | | | | | | | | | | | | | | | | | | | | | | | | | | |
| Statistical methods | 1 | For continuous scores: Were correlations between change scores, or the area under the Receiver Operator Curve (ROC) curve calculated? |  |  |  |  |  |  |  |  |  |  |  |  |  |  |  |  |  |  |  |  |  |  |  |  |  |  |  |  |  |  |
|  | 2 | For dichotomous scales: Were sensitivity and specificity (changed versus not changed) determined? |  |  |  |  |  |  |  |  |  |  |  |  |  |  |  |  |  |  |  |  |  |  |  |  |  |  |  |  |  |  |
| Other | 3 | Were there any other important flaws in the design or statistical methods of the study? |  |  |  |  |  |  |  |  |  |  |  |  |  |  |  |  |  |  |  |  |  |  |  |  |  |  |  |  |  |  |
| CONSENSUS | | |  |  |  |  |  |  |  |  |  |  |  |  |  |  |  |  |  |  |  |  |  |  |  |  |  |  |  |  |  |  |
| 10b. Construct approach (i.e. hypotheses testing; comparison with other outcome measurement instruments) | | | | | | | | | | | | | | | | | | | | | | | | | | | | | | | | |
| Design requirements | 4 | Is it clear what the comparator instrument(s) measure(s)? |  |  |  |  |  |  |  |  |  |  |  |  | VG |  | **VG** |  |  |  |  |  |  |  |  |  |  |  |  |  |  |  |
|  | 5 | Were the measurement properties of the comparator instrument(s) sufficient? |  |  |  |  |  |  |  |  |  |  |  |  | I |  | **I** |  |  |  |  |  |  |  |  |  |  |  |  |  |  |  |
| Statistical methods | 6 | Was the statistical method appropriate for the hypotheses to be tested? |  |  |  |  |  |  |  |  |  |  |  |  | I |  | **I** |  |  |  |  |  |  |  |  |  |  |  |  |  |  |  |
| Other | 7 | Were there any other important flaws in the design or statistical methods of the study? |  |  |  |  |  |  |  |  |  |  |  |  | VG |  | **VG** |  |  |  |  |  |  |  |  |  |  |  |  |  |  |  |
| CONSENSUS | | |  |  |  |  |  |  |  |  |  |  |  |  |  |  | **I** |  |  |  |  |  |  |  |  |  |  |  |  |  |  |  |
| 10c. Construct approach (i.e. hypotheses testing: comparison between subgroups) | | | | | | | | | | | | | | | | | | | | | | | | | | | | | | | | |
| Design requirements | 8 | Was an A description provided of important characteristics of the subgroups? |  |  |  |  |  |  |  |  |  |  |  |  |  |  |  |  |  |  |  |  |  |  |  |  |  |  |  |  |  |  |
| Statistical methods | 9 | Was the statistical method appropriate for the hypotheses to be tested? |  |  |  |  |  |  |  |  |  |  |  |  |  |  |  |  |  |  |  |  |  |  |  |  |  |  |  |  |  |  |
| Other | 10 | Were there any other important flaws in the design or statistical methods of the study? |  |  |  |  |  |  |  |  |  |  |  |  |  |  |  |  |  |  |  |  |  |  |  |  |  |  |  |  |  |  |
| CONSENSUS | | |  |  |  |  |  |  |  |  |  |  |  |  |  |  |  |  |  |  |  |  |  |  |  |  |  |  |  |  |  |  |
| 10d. Construct approach (i.e. hypotheses testing: before and after intervention) | | | | | | | | | | | | | | | | | | | | | | | | | | | | | | | | |
| Design requirements | 11 | Was an A description provided of the intervention given? |  |  |  |  |  |  |  |  |  |  |  |  | VG | VG | **VG** |  |  |  |  |  |  |  |  |  |  |  |  |  |  |  |
| Statistical methods | 12 | Was the statistical method appropriate for the hypotheses to be tested? |  |  |  |  |  |  |  |  |  |  |  |  | VG | VG | **VG** |  |  |  |  |  |  |  |  |  |  |  |  |  |  |  |
| Other | 13 | Were there any other important flaws in the design or statistical methods of the study? |  |  |  |  |  |  |  |  |  |  |  |  | VG | VG | **VG** |  |  |  |  |  |  |  |  |  |  |  |  |  |  |  |
| CONSENSUS | | |  |  |  |  |  |  |  |  |  |  |  |  |  |  | **VG** |  |  |  |  |  |  |  |  |  |  |  |  |  |  |  |
| % | | | **93.33%** | | | **71.05%** | | | **87.88%** | | | **65.00%** | | | **64.00%** | | | **79.17%** | | | **92.59%** | | | **0.00%** | | | **87.50%** | | | **91.30%** | | |

A: adequate; C: consensus; D: doubtful; I: inadequate; N: no; N/A: not applicable; R1: Reviewer 1; R2: Reviewer 2; SV: structural validity; VG: very good; Y: yes

| MQOL – PART 2 | | | | | | | | | | | | | | | | | | | | | | | | | | |
| --- | --- | --- | --- | --- | --- | --- | --- | --- | --- | --- | --- | --- | --- | --- | --- | --- | --- | --- | --- | --- | --- | --- | --- | --- | --- | --- |
| COSMIN Risk of Bias Checklist | | | Bentur & Resnizky, 2005 | | | Kim et al., 2007 | | | Shahdi, et al., 2006 | | | Tsujilkawa et al., 2009 | | | Sguazzin et al., 2010 | | | Gofton et al., 2015 | | | Hu et al., 2015 | | | De Vrieze et al., 2016 | | |
|  | Item | Requirements | R1 | R2 | **C** | R1 | R2 | **C** | R1 | R2 | **C** | R1 | R2 | **C** | R1 | R2 | **C** | R1 | R2 | **C** | R1 | R2 | **C** | R1 | R2 | **C** |
| Box 1. PROM Development | | | | | | | | | | | | | | | | | | | | | | | | | | |
| 1a. PROM design | | | | | | | | | | | | | | | | | | | | | | | | | | |
|  | 1 | Is a clear description provided of the construct to be measured? |  |  |  |  |  |  |  |  |  |  |  |  |  |  |  |  |  |  |  |  |  |  |  |  |
|  | 2 | Is the origin of the construct clear: was a theory, conceptual framework or disease model used or clear rationale provided to define the construct to be measured? |  |  |  |  |  |  |  |  |  |  |  |  |  |  |  |  |  |  |  |  |  |  |  |  |
|  | 3 | Is a clear description provided of the target population for which the PROM was developed? |  |  |  |  |  |  |  |  |  |  |  |  |  |  |  |  |  |  |  |  |  |  |  |  |
|  | 4 | Is a clear description provided of the context of use |  |  |  |  |  |  |  |  |  |  |  |  |  |  |  |  |  |  |  |  |  |  |  |  |
|  | 5 | Was the PROM development study performed in a sample representing the target population for which the PROM was developed? |  |  |  |  |  |  |  |  |  |  |  |  |  |  |  |  |  |  |  |  |  |  |  |  |
|  | 6 | Was an appropriate qualitative data collection method used to identify relevant items for a new PROM? |  |  |  |  |  |  |  |  |  |  |  |  |  |  |  |  |  |  |  |  |  |  |  |  |
|  | 7 | Were skilled group moderators/interviewers used? |  |  |  |  |  |  |  |  |  |  |  |  |  |  |  |  |  |  |  |  |  |  |  |  |
|  | 8 | Were the group meetings or interviews based on an appropriate topic or interview guide? |  |  |  |  |  |  |  |  |  |  |  |  |  |  |  |  |  |  |  |  |  |  |  |  |
|  | 9 | Were the group meetings or interviews recorded and transcribed verbatim? |  |  |  |  |  |  |  |  |  |  |  |  |  |  |  |  |  |  |  |  |  |  |  |  |
|  | 10 | Was an appropriate approach used to analyse the data? |  |  |  |  |  |  |  |  |  |  |  |  |  |  |  |  |  |  |  |  |  |  |  |  |
|  | 11 | Was at least part of the data coded independently? |  |  |  |  |  |  |  |  |  |  |  |  |  |  |  |  |  |  |  |  |  |  |  |  |
|  | 12 | Was data collection continued until saturation was reached? |  |  |  |  |  |  |  |  |  |  |  |  |  |  |  |  |  |  |  |  |  |  |  |  |
|  | 13 | For quantitative studies (surveys): was the sample size appropriate? |  |  |  |  |  |  |  |  |  |  |  |  |  |  |  |  |  |  |  |  |  |  |  |  |
| 1b. Cognitive interview study or pilot test | | | | | | | | | | | | | | | | | | | | | | | | | | |
|  | 14 | Was a cognitive interview study or other pilot test conducted? |  |  |  |  |  |  |  |  |  |  |  |  |  |  |  |  |  |  |  |  |  |  |  |  |
| General design requirements | 15 | Was the cognitive interview study or other pilot test performed in a sample representing the target population? |  |  |  |  |  |  |  |  |  |  |  |  |  |  |  |  |  |  |  |  |  |  |  |  |
| Comprehensibility | 16 | Were patients asked about the comprehensibility of the PROM? |  |  |  |  |  |  |  |  |  |  |  |  |  |  |  |  |  |  |  |  |  |  |  |  |
|  | 17 | Were all items tested in their fIl form? |  |  |  |  |  |  |  |  |  |  |  |  |  |  |  |  |  |  |  |  |  |  |  |  |
|  | 18 | Was an appropriate qualitative method used to assess the comprehensibility of the PROM instructions, items, response options, and recall period? |  |  |  |  |  |  |  |  |  |  |  |  |  |  |  |  |  |  |  |  |  |  |  |  |
|  | 19 | Was each item tested in an appropriate number of patients? |  |  |  |  |  |  |  |  |  |  |  |  |  |  |  |  |  |  |  |  |  |  |  |  |
|  |  | For qualitative studies |  |  |  |  |  |  |  |  |  |  |  |  |  |  |  |  |  |  |  |  |  |  |  |  |
|  |  | For quantitative (survey) studies |  |  |  |  |  |  |  |  |  |  |  |  |  |  |  |  |  |  |  |  |  |  |  |  |
|  | 20 | Were skilled interviewers used? |  |  |  |  |  |  |  |  |  |  |  |  |  |  |  |  |  |  |  |  |  |  |  |  |
|  | 21 | Were the interviews based on an appropriate interview guide? |  |  |  |  |  |  |  |  |  |  |  |  |  |  |  |  |  |  |  |  |  |  |  |  |
|  | 22 | Were the interviews recorded and transcribed verbatim? |  |  |  |  |  |  |  |  |  |  |  |  |  |  |  |  |  |  |  |  |  |  |  |  |
|  | 23 | Was an appropriate approach used to analyse the data? |  |  |  |  |  |  |  |  |  |  |  |  |  |  |  |  |  |  |  |  |  |  |  |  |
|  | 24 | Were at least two researchers involved in the analysis? |  |  |  |  |  |  |  |  |  |  |  |  |  |  |  |  |  |  |  |  |  |  |  |  |
|  | 25 | Were problems regarding the comprehensibility of the PROM instructions, items, response options, and recall period appropriately addressed by adapting the PROM? |  |  |  |  |  |  |  |  |  |  |  |  |  |  |  |  |  |  |  |  |  |  |  |  |
| Comprehensiveness | 26 | Were patients asked about the comprehensiveness of the PROM? |  |  |  |  |  |  |  |  |  |  |  |  |  |  |  |  |  |  |  |  |  |  |  |  |
|  | 27 | Was the final set of items tested? |  |  |  |  |  |  |  |  |  |  |  |  |  |  |  |  |  |  |  |  |  |  |  |  |
|  | 28 | Was an appropriate method used for assessing the comprehensiveness of the PROM? |  |  |  |  |  |  |  |  |  |  |  |  |  |  |  |  |  |  |  |  |  |  |  |  |
|  | 29 | Was each item tested in an appropriate number of patients? |  |  |  |  |  |  |  |  |  |  |  |  |  |  |  |  |  |  |  |  |  |  |  |  |
|  |  | For qualitative studies |  |  |  |  |  |  |  |  |  |  |  |  |  |  |  |  |  |  |  |  |  |  |  |  |
|  |  | For quantitative (survey) studies |  |  |  |  |  |  |  |  |  |  |  |  |  |  |  |  |  |  |  |  |  |  |  |  |
|  | 30 | Were skilled interviewers used? |  |  |  |  |  |  |  |  |  |  |  |  |  |  |  |  |  |  |  |  |  |  |  |  |
|  | 31 | Were the interviews based on an appropriate interview guide? |  |  |  |  |  |  |  |  |  |  |  |  |  |  |  |  |  |  |  |  |  |  |  |  |
|  | 32 | Were the interviews recorded and transcribed verbatim? |  |  |  |  |  |  |  |  |  |  |  |  |  |  |  |  |  |  |  |  |  |  |  |  |
|  | 33 | Was an appropriate approach used to analyse the data? |  |  |  |  |  |  |  |  |  |  |  |  |  |  |  |  |  |  |  |  |  |  |  |  |
|  | 34 | Were at least two researchers involved in the analysis? |  |  |  |  |  |  |  |  |  |  |  |  |  |  |  |  |  |  |  |  |  |  |  |  |
|  | 35 | Were problems regarding the comprehensiveness of the PROM appropriately addressed by adapting the PROM? |  |  |  |  |  |  |  |  |  |  |  |  |  |  |  |  |  |  |  |  |  |  |  |  |
| CONSENSUS | | |  |  |  |  |  |  |  |  |  |  |  |  |  |  |  |  |  |  |  |  |  |  |  |  |
| Box 2. Content validity | | | | | | | | | | | | | | | | | | | | | | | | | | |
| 2a. Asking patients about relevance | | | | | | | | | | | | | | | | | | | | | | | | | | |
| Design requirements | 1 | Was an appropriate method used to ask patients whether each item is relevant for their experience with the condition? |  |  |  |  |  |  |  |  |  |  |  |  |  |  |  |  |  |  |  |  |  | A | VG | **A** |
|  | 2 | Was each item tested in an appropriate number of patients? |  |  |  |  |  |  |  |  |  |  |  |  |  |  |  |  |  |  |  |  |  |  |  |  |
|  |  | For qualitative studies |  |  |  |  |  |  |  |  |  |  |  |  |  |  |  |  |  |  |  |  |  | VG | VG |  |
|  |  | For quantitative (survey) studies |  |  |  |  |  |  |  |  |  |  |  |  |  |  |  |  |  |  |  |  |  |  |  | **A** |
|  | 3 | Were skilled group moderators/interviewers used? |  |  |  |  |  |  |  |  |  |  |  |  |  |  |  |  |  |  |  |  |  | N/A | D | **N/A** |
|  | 4 | Were the group meetings or interviews based on an appropriate topic or interview guide? |  |  |  |  |  |  |  |  |  |  |  |  |  |  |  |  |  |  |  |  |  | N/A | N/A | **N/A** |
|  | 5 | Were the group meetings or interviews recorded and transcribed verbatim? |  |  |  |  |  |  |  |  |  |  |  |  |  |  |  |  |  |  |  |  |  | N/A | N/A | **N/A** |
| Analyses | 6 | Was an appropriate approach used to analyse the data? |  |  |  |  |  |  |  |  |  |  |  |  |  |  |  |  |  |  |  |  |  | D | D | **D** |
|  | 7 | Were at least two researchers involved in the analysis? |  |  |  |  |  |  |  |  |  |  |  |  |  |  |  |  |  |  |  |  |  | D | D | **D** |
| 2b. Asking patients about comprehensiveness | | | | | | | | | | | | | | | | | | | | | | | | | | |
| Design requirements | 8 | Was an appropriate method used for assessing the comprehensiveness of the PROM? |  |  |  |  |  |  |  |  |  |  |  |  |  |  |  | A | A | **A** |  |  |  |  | VG |  |
|  | 9 | Was each item tested in an appropriate number of patients? |  |  |  |  |  |  |  |  |  |  |  |  |  |  |  |  |  |  |  |  |  |  |  |  |
|  |  | For qualitative studies |  |  |  |  |  |  |  |  |  |  |  |  |  |  |  |  |  |  |  |  |  |  |  |  |
|  |  | For quantitative (survey) studies |  |  |  |  |  |  |  |  |  |  |  |  |  |  |  | VG | VG | **VG** |  |  |  |  | A |  |
|  | 10 | Were skilled group moderators/interviewers used? |  |  |  |  |  |  |  |  |  |  |  |  |  |  |  | N/A | N/A | **N/A** |  |  |  |  | N/A |  |
|  | 11 | Were the group meetings or interviews based on an appropriate topic or interview guide? |  |  |  |  |  |  |  |  |  |  |  |  |  |  |  | N/A | N/A | **N/A** |  |  |  |  | N/A |  |
|  | 12 | Were the group meetings or interviews recorded and transcribed verbatim? |  |  |  |  |  |  |  |  |  |  |  |  |  |  |  | N/A | N/A | **N/A** |  |  |  |  | N/A |  |
| Analyses | 13 | Was an appropriate approach used to analyse the data? |  |  |  |  |  |  |  |  |  |  |  |  |  |  |  | D | D | **D** |  |  |  |  | D |  |
|  | 14 | Were at least two researchers involved in the analysis? |  |  |  |  |  |  |  |  |  |  |  |  |  |  |  | D | D | **D** |  |  |  |  | D |  |
| 2c. Asking patients about comprehensibility | | | | | | | | | | | | | | | | | | | | | | | | | | |
| Design requirements | 15 | Was an appropriate qualitative method used for assessing the comprehensibility of the PROM instructions, items, response options, and recall period? |  |  |  | VG | A | **VG** |  |  |  |  |  |  | VG |  | **VG** | D | D | **D** |  |  |  |  |  | **A** |
|  | 16 | Was each item tested in an appropriate number of patients? |  |  |  |  |  |  |  |  |  |  |  |  |  |  |  |  |  |  |  |  |  |  |  |  |
|  |  | For qualitative studies |  |  |  | VG | VG | **VG** |  |  |  |  |  |  | VG | VG | **VG** |  |  |  |  |  |  |  |  |  |
|  |  | For quantitative (survey) studies |  |  |  |  |  |  |  |  |  |  |  |  |  |  |  | VG | VG | **VG** |  |  |  |  |  | **A** |
|  | 17 | Were skilled group moderators/interviewers used? |  |  |  | D | D | **D** |  |  |  |  |  |  | VG | D | **D** | D | N/A | **N/A** |  |  |  |  |  | **N/A** |
|  | 18 | Were the group meetings or interviews based on an appropriate topic or interview guide? |  |  |  | D | D | **D** |  |  |  |  |  |  | D | D | **D** | N/A | N/A | **N/A** |  |  |  |  |  | **N/A** |
|  | 19 | Were the group meetings or interviews recorded and transcribed verbatim? |  |  |  | D | I | **D** |  |  |  |  |  |  | D | D | **D** | N/A | N/A | **N/A** |  |  |  |  |  | **N/A** |
| Analyses | 20 | Was an appropriate approach used to analyse the data? |  |  |  | D | D | **D** |  |  |  |  |  |  | D | D | **D** | D | D | **D** |  |  |  |  |  | **D** |
|  | 21 | Were at least two researchers involved in the analysis? |  |  |  | D | A | **D** |  |  |  |  |  |  | D | D | **D** | D | D | **D** |  |  |  |  |  | **D** |
| 2d. Asking professionals about relevance | | | | | | | | | | | | | | | | | | | | | | | | | | |
| Design requirements | 22 | Was an appropriate method used to ask professionals whether each item is relevant for the construct of interest? |  |  |  |  |  |  |  |  |  |  |  |  |  |  |  |  |  |  |  |  |  |  |  |  |
|  | 23 | Were professionals from all relevant disciplines included? |  |  |  |  |  |  |  |  |  |  |  |  |  |  |  |  |  |  |  |  |  |  |  |  |
|  | 24 | Was each item tested in an appropriate number of professionals? |  |  |  |  |  |  |  |  |  |  |  |  |  |  |  |  |  |  |  |  |  |  |  |  |
|  |  | For qualitative studies |  |  |  |  |  |  |  |  |  |  |  |  |  |  |  |  |  |  |  |  |  |  |  |  |
|  |  | For quantitative (survey) studies |  |  |  |  |  |  |  |  |  |  |  |  |  |  |  |  |  |  |  |  |  |  |  |  |
| Analyses | 25 | Was an appropriate approach used to analyse the data? |  |  |  |  |  |  |  |  |  |  |  |  |  |  |  |  |  |  |  |  |  |  |  |  |
|  | 26 | Were at least two researchers involved in the analysis? |  |  |  |  |  |  |  |  |  |  |  |  |  |  |  |  |  |  |  |  |  |  |  |  |
| 2e. Asking professionals about comprehensiveness | | | | | | | | | | | | | | | | | | | | | | | | | | |
| Design requirements | 27 | Was an appropriate method used for assessing the comprehensiveness of the PROM? |  |  |  |  |  |  |  |  |  |  |  |  |  |  |  |  |  |  |  |  |  |  |  |  |
|  | 28 | Were professionals from all relevant disciplines included? |  |  |  |  |  |  |  |  |  |  |  |  |  |  |  |  |  |  |  |  |  |  |  |  |
|  | 29 | Was each item tested in an appropriate number of professionals? |  |  |  |  |  |  |  |  |  |  |  |  |  |  |  |  |  |  |  |  |  |  |  |  |
|  |  | For qualitative studies |  |  |  |  |  |  |  |  |  |  |  |  |  |  |  |  |  |  |  |  |  |  |  |  |
|  |  | For quantitative (survey) studies |  |  |  |  |  |  |  |  |  |  |  |  |  |  |  |  |  |  |  |  |  |  |  |  |
| Analyses | 30 | Was an appropriate approach used to analyse the data? |  |  |  |  |  |  |  |  |  |  |  |  |  |  |  |  |  |  |  |  |  |  |  |  |
|  | 31 | Were at least two researchers involved in the analysis? |  |  |  |  |  |  |  |  |  |  |  |  |  |  |  |  |  |  |  |  |  |  |  |  |
| CONSENSUS | | |  |  |  |  |  | D |  |  |  |  |  |  |  |  | D |  |  | D |  |  |  |  |  | D |
| Box 3. Structural validity | | | | | | | | | | | | | | | | | | | | | | | | | | |
|  |  | Does the scale consist of effect indicators, i.e. is it based on a reflective model? | Y | Y | Y |  | Y | **Y** |  |  |  | Y | Y | **Y** | Y | Y | Y | Y | Y | **Y** | Y | Y | **Y** |  |  |  |
|  |  | Does the study concern unidimensionality or structural validity? | SV | SV | **SV** |  | SV | **SV** |  |  |  | SV | SV | **SV** | SV | SV | **SV** | SV | SV | **SV** | SV | SV | **SV** |  |  |  |
| Statistical methods | 1 | For CTT: Was exploratory or confirmatory factor analysis performed? | A | A | **A** |  | A | **A** |  |  |  | A | N/A | **A** | A | A | **A** | A | A | **A** | VG | A | **VG** |  |  |  |
|  | 2 | For IRT/Rasch: does the chosen model fit to the research question? | N/A | N/A | **N/A** |  | VG | **N/A** |  |  |  | N/A | N/A | **N/A** | N/A | N/A | **N/A** | N/A | N/A | **N/A** | N/A | N/A | **N/A** |  |  |  |
|  | 3 | Was the sample size included in the analysis A? |  |  |  |  |  |  |  |  |  |  |  |  |  |  |  |  |  |  |  |  |  |  |  |  |
|  |  | For FA | VG | VG | **VG** |  | VG | **VG** |  |  |  | D | D | **D** | VG | VG | **VG** | D | D | **D** | I | I | **I** |  |  |  |
|  |  | For Rasch/1PL models |  |  |  |  |  |  |  |  |  |  |  |  |  |  |  |  |  |  |  |  |  |  |  |  |
|  |  | For 2PL parametric IRT models OR Mokken scale analysis |  |  |  |  |  |  |  |  |  |  |  |  |  |  |  |  |  |  |  |  |  |  |  |  |
| Other | 4 | Were there any other important flaws in the design or statistical methods of the study? | VG | VG | **VG** |  | VG | **VG** |  |  |  | VG | VG | **VG** | VG | VG | **VG** | VG | VG | **VG** | VG | VG | **VG** |  |  |  |
| CONSENSUS | | |  |  | **A** |  |  | **A** |  |  |  |  |  | **D** |  |  | **A** |  |  | **D** |  |  | **I** |  |  |  |
| Box 4. Internal consistency | | | | | | | | | | | | | | | | | | | | | | | | | | |
|  |  | Does the scale consist of effect indicators, i.e. is it based on a reflective model? | Y | Y | **Y** | Y | Y | **Y** | Y | Y | **Y** | Y | Y | **Y** | Y | Y | **Y** | Y | Y | **Y** | Y | Y | **Y** | Y | Y | **Y** |
| Design requirements | 1 | Was an internal consistency statistic calculated for each unidimensional scale or subscale separately? | VG | VG | **VG** | VG | VG | **VG** | VG | VG | **VG** | VG | VG | **VG** | VG | VG | **VG** | VG | VG | **VG** | VG | VG | **VG** | VG | VG | **VG** |
| Statistical methods | 2 | For continuous scores: Was Cronbach’s alpha or omega calculated? | VG | VG | **VG** | VG | VG | **VG** | VG | VG | **VG** | VG | VG | **VG** | VG | VG | **VG** | VG | VG | **VG** | VG | VG | **VG** | VG | VG | **VG** |
|  | 3 | For dichotomous scores: Was Cronbach’s alpha or KR-20 calculated? | N/A | N/A | **N/A** | N/A | I | **N/A** | N/A | N/A | **N/A** | N/A | N/A | **N/A** | N/A | N/A | **N/A** | N/A | N/A | **N/A** | N/A | N/A | **N/A** | N/A | N/A | **N/A** |
|  | 4 | For IRT-based scores: Was standard error of the theta (SE (θ)) or reliability coefficient of estimated latent trait value (index of (subject or item) separation) calculated? | N/A | N/A | **N/A** | N/A | N/A | **N/A** | N/A | N/A | **N/A** | N/A | N/A | **N/A** | N/A | N/A | **N/A** | N/A | N/A | **N/A** | N/A | N/A | **N/A** | N/A | N/A | **N/A** |
| Other | 5 | Were there any other important flaws in the design or statistical methods of the study? | VG | VG | **VG** | VG | VG | **VG** | VG | VG | **VG** | VG | VG | **VG** | VG | VG | **VG** | VG | VG | **VG** | VG | VG | **VG** | VG | VG | **VG** |
| CONSENSUS | | |  |  | **VG** |  |  | **VG** |  |  | **VG** |  |  | **VG** |  |  | **VG** |  |  | **VG** |  |  | **VG** |  |  | **VG** |
| Box 5. Cross-cultural validity/Measurement invariance | | | | | | | | | | | | | | | | | | | | | | | | | | |
| Design requirements | 1 | Were the samples similar for relevant characteristics except for the group variable? |  |  |  |  |  |  |  |  |  |  |  |  |  |  |  |  |  |  |  |  |  |  |  |  |
| Statistical methods | 2 | Was an appropriate approach used to analyse the data? |  |  |  |  |  |  |  |  |  |  |  |  |  |  |  |  |  |  |  |  |  |  |  |  |
|  | 3 | Was the sample size included in the analysis A? |  |  |  |  |  |  |  |  |  |  |  |  |  |  |  |  |  |  |  |  |  |  |  |  |
|  |  | For Regression analyses or IRT/Rasch based analyses |  |  |  |  |  |  |  |  |  |  |  |  |  |  |  |  |  |  |  |  |  |  |  |  |
|  |  | For MGCFA* |  |  |  |  |  |  |  |  |  |  |  |  |  |  |  |  |  |  |  |  |  |  |  |  |
| Other | 4 | Were there any other important flaws in the design or statistical methods of the study? |  |  |  |  |  |  |  |  |  |  |  |  |  |  |  |  |  |  |  |  |  |  |  |  |
| CONSENSUS | | |  |  |  |  |  |  |  |  |  |  |  |  |  |  |  |  |  |  |  |  |  |  |  |  |
| Box 6. Reliability | | | | | | | | | | | | | | | | | | | | | | | | | | |
| Design requirements | 1 | Were patients stable in the interim period on the construct to be measured? |  |  |  |  |  |  | D | D | **D** |  |  |  |  |  |  |  |  |  | A | A | **A** | A | A | **A** |
|  | 2 | Was the time interval appropriate? |  |  |  |  |  |  | D | VG | **VG** |  |  |  |  |  |  |  |  |  | VG | VG | **VG** | VG | D | **D** |
|  | 3 | Were the test conditions similar for the measurements? e.g. type of administration, environment, instructions |  |  |  |  |  |  | A | A | **A** |  |  |  |  |  |  |  |  |  | VG | A | **A** | I | A | **I** |
| Statistical methods | 4 | For continuous scores: Was an intraclass correlation coefficient (ICC) calculated? |  |  |  |  |  |  | D | D | **D** |  |  |  |  |  |  |  |  |  | D | D | **D** | A | VG | **A** |
|  | 5 | For dichotomous/nominal/ordinal scores: Was kappa calculated? |  |  |  |  |  |  | N/A | N/A | **N/A** |  |  |  |  |  |  |  |  |  | N/A | N/A | **N/A** | N/A | N/A | **N/A** |
|  | 6 | For ordinal scores: Was a weighted kappa calculated? |  |  |  |  |  |  | N/A | N/A | **N/A** |  |  |  |  |  |  |  |  |  | N/A | N/A | **N/A** | N/A | N/A | **N/A** |
|  | 7 | For ordinal scores: Was the weighting scheme described? e.g. linear, quadratic |  |  |  |  |  |  | N/A | N/A | **N/A** |  |  |  |  |  |  |  |  |  | N/A | A | **N/A** | N/A | N/A | **N/A** |
| Other | 8 | Were there any other important flaws in the design or statistical methods of the study? |  |  |  |  |  |  | VG | VG | **VG** |  |  |  |  |  |  |  |  |  | VG | VG | **VG** | VG | VG | **VG** |
| CONSENSUS | | |  |  |  |  |  |  |  |  | **D** |  |  |  |  |  |  |  |  |  |  |  | **D** |  |  | **I** |
| Box 7. Measurement error | | | | | | | | | | | | | | | | | | | | | | | | | | |
| Design requirements | 1 | Were patients stable in the interim period on the construct to be measured? |  |  |  |  |  |  |  |  |  |  |  |  |  |  |  |  |  |  |  |  |  | A | A | **A** |
|  | 2 | Was the time interval appropriate? |  |  |  |  |  |  |  |  |  |  |  |  |  |  |  |  |  |  |  |  |  | VG | D | **D** |
|  | 3 | Were the test conditions similar for the measurements? e.g. type of administration, environment, instructions |  |  |  |  |  |  |  |  |  |  |  |  |  |  |  |  |  |  |  |  |  | I | A | **I** |
| Statistical methods | 4 | For continuous scores: Was the Standard Error of Measurement (SEM), Smallest Detectable Change (SDC) or Limits of Agreement (LoA) calculated? |  |  |  |  |  |  |  |  |  |  |  |  |  |  |  |  |  |  |  |  |  | VG | I | **VG** |
|  | 5 | For dichotomous/nominal/ordinal scores: Was the percentage (positive and negative) agreement calculated? |  |  |  |  |  |  |  |  |  |  |  |  |  |  |  |  |  |  |  |  |  | N/A | N/A | **N/A** |
| Other | 6 | Were there any other important flaws in the design or statistical methods of the study? |  |  |  |  |  |  |  |  |  |  |  |  |  |  |  |  |  |  |  |  |  | VG | VG | **VG** |
| CONSENSUS | | |  |  |  |  |  |  |  |  |  |  |  |  |  |  |  |  |  |  |  |  |  |  |  | **I** |
| Box 8. Criterion validity | | | | | | | | | | | | | | | | | | | | | | | | | | |
| Statistical methods | 1 | For continuous scores: Were correlations, or the area under the receiver operating curve calculated? |  |  |  |  |  |  |  |  |  |  |  |  |  |  |  |  |  |  |  |  |  |  |  |  |
|  | 2 | For dichotomous scores: Were sensitivity and specificity determined? |  |  |  |  |  |  |  |  |  |  |  |  |  |  |  |  |  |  |  |  |  |  |  |  |
| Other | 3 | Were there any other important flaws in the design or statistical methods of the study? |  |  |  |  |  |  |  |  |  |  |  |  |  |  |  |  |  |  |  |  |  |  |  |  |
| CONSENSUS | | |  |  |  |  |  |  |  |  |  |  |  |  |  |  |  |  |  |  |  |  |  |  |  |  |
| Box 9. Hypotheses testing for construct validity | | | | | | | | | | | | | | | | | | | | | | | | | | |
| 9a. Comparison with other outcome measurement instruments (convergent validity) | | | | | | | | | | | | | | | | | | | | | | | | | | |
| Design requirements | 1 | Is it clear what the comparator instrument(s) measure(s)? |  |  |  | VG | VG | **VG** | VG | VG | **VG** |  |  |  | VG | VG | **VG** | VG | VG | **VG** |  |  |  | VG | VG | **VG** |
|  | 2 | Were the measurement properties of the comparator instrument(s) sufficient? |  |  |  | VG | VG | **VG** | VG | VG | **VG** |  |  |  | VG | VG | **VG** | VG | VG | **VG** |  |  |  | VG | VG | **VG** |
| Statistical methods | 3 | Was the statistical method appropriate for the hypotheses to be tested? |  |  |  | I | D | **D** | VG | D | **VG** |  |  |  | VG | VG | **VG** | I | D | **I** |  |  |  | I | D | **D** |
| Other | 4 | Were there any other important flaws in the design or statistical methods of the study? |  |  |  | VG | VG | **VG** | VG | VG | **VG** |  |  |  | VG | VG | **VG** | VG | VG | **VG** |  |  |  | VG | VG | **VG** |
| CONSENSUS | | |  |  |  |  |  | **D** |  |  | **VG** |  |  |  |  |  | **VG** |  |  | **I** |  |  |  |  |  | **D** |
| 9b. Comparison between subgroups (discriminative or known-groups validity) | | | | | | | | | | | | | | | | | | | | | | | | | | |
| Design requirements | 5 | Was an A description provided of important characteristics of the subgroups? |  |  |  | VG | VG | **VG** | VG |  |  |  |  |  |  |  |  |  |  |  |  |  |  |  |  |  |
| Statistical methods | 6 | Was the statistical method appropriate for the hypotheses to be tested? |  |  |  | VG | VG | **VG** | VG |  |  |  |  |  |  |  |  |  |  |  |  |  |  |  |  |  |
| Other | 7 | Were there any other important flaws in the design or statistical methods of the study? |  |  |  | VG | VG | **VG** | VG |  |  |  |  |  |  |  |  |  |  |  |  |  |  |  |  |  |
| CONSENSUS | | |  |  |  |  |  | **VG** |  |  |  |  |  |  |  |  |  |  |  |  |  |  |  |  |  |  |
| Box 10. Responsiveness | | | | | | | | | | | | | | | | | | | | | | | | | | |
| 10a. Criterion approach (i.e. comparison to a gold standard) | | | | | | | | | | | | | | | | | | | | | | | | | | |
| Statistical methods | 1 | For continuous scores: Were correlations between change scores, or the area under the Receiver Operator Curve (ROC) curve calculated? |  |  |  |  |  |  |  |  |  |  |  |  |  |  |  |  |  |  |  |  |  |  |  |  |
|  | 2 | For dichotomous scales: Were sensitivity and specificity (changed versus not changed) determined? |  |  |  |  |  |  |  |  |  |  |  |  |  |  |  |  |  |  |  |  |  |  |  |  |
| Other | 3 | Were there any other important flaws in the design or statistical methods of the study? |  |  |  |  |  |  |  |  |  |  |  |  |  |  |  |  |  |  |  |  |  |  |  |  |
| CONSENSUS | | |  |  |  |  |  |  |  |  |  |  |  |  |  |  |  |  |  |  |  |  |  |  |  |  |
| 10b. Construct approach (i.e. hypotheses testing; comparison with other outcome measurement instruments) | | | | | | | | | | | | | | | | | | | | | | | | | | |
| Design requirements | 4 | Is it clear what the comparator instrument(s) measure(s)? |  |  |  |  |  |  |  |  |  |  |  |  |  |  |  |  |  |  |  |  |  |  |  |  |
|  | 5 | Were the measurement properties of the comparator instrument(s) sufficient? |  |  |  |  |  |  |  |  |  |  |  |  |  |  |  |  |  |  |  |  |  |  |  |  |
| Statistical methods | 6 | Was the statistical method appropriate for the hypotheses to be tested? |  |  |  |  |  |  |  |  |  |  |  |  |  |  |  |  |  |  |  |  |  |  |  |  |
| Other | 7 | Were there any other important flaws in the design or statistical methods of the study? |  |  |  |  |  |  |  |  |  |  |  |  |  |  |  |  |  |  |  |  |  |  |  |  |
| CONSENSUS | | |  |  |  |  |  |  |  |  |  |  |  |  |  |  |  |  |  |  |  |  |  |  |  |  |
| 10c. Construct approach (i.e. hypotheses testing: comparison between subgroups) | | | | | | | | | | | | | | | | | | | | | | | | | | |
| Design requirements | 8 | Was an A description provided of important characteristics of the subgroups? |  |  |  |  |  |  |  |  |  |  |  |  |  |  |  |  |  |  |  |  |  |  |  |  |
| Statistical methods | 9 | Was the statistical method appropriate for the hypotheses to be tested? |  |  |  |  |  |  |  |  |  |  |  |  |  |  |  |  |  |  |  |  |  |  |  |  |
| Other | 10 | Were there any other important flaws in the design or statistical methods of the study? |  |  |  |  |  |  |  |  |  |  |  |  |  |  |  |  |  |  |  |  |  |  |  |  |
| CONSENSUS | | |  |  |  |  |  |  |  |  |  |  |  |  |  |  |  |  |  |  |  |  |  |  |  |  |
| 10d. Construct approach (i.e. hypotheses testing: before and after intervention) | | | | | | | | | | | | | | | | | | | | | | | | | | |
| Design requirements | 11 | Was an A description provided of the intervention given? |  |  |  |  |  |  |  |  |  |  |  |  |  |  |  |  |  |  |  |  |  |  |  |  |
| Statistical methods | 12 | Was the statistical method appropriate for the hypotheses to be tested? |  |  |  |  |  |  |  |  |  |  |  |  |  |  |  |  |  |  |  |  |  |  |  |  |
| Other | 13 | Were there any other important flaws in the design or statistical methods of the study? |  |  |  |  |  |  |  |  |  |  |  |  |  |  |  |  |  |  |  |  |  |  |  |  |
| CONSENSUS | | |  |  |  |  |  |  |  |  |  |  |  |  |  |  |  |  |  |  |  |  |  |  |  |  |
| % | | | **100.00%** | | | **57.69%** | | | **76.19%** | | | **91.67%** | | | **91.30%** | | | **93.33%** | | | **85.00%** | | | **57.89%** | | |

A: adequate; C: consensus; D: doubtful; I: inadequate; N/A: not applicable; R1: Reviewer 1; R2: Reviewer 2; SV: structural validity; U: unidimensionality; VG: very good; Y: yes

| MUDI | | | | | | | | | | | |
| --- | --- | --- | --- | --- | --- | --- | --- | --- | --- | --- | --- |
| COSMIN Risk of Bias Checklist | | | Robinson & Shea, 2002 | | | Robinson et al., 2007 | | | Lim et al., 2009 | | |
|  | Item | Requirements | R1 | R2 | **C** | R1 | R2 | **C** | R1 | R2 | **C** |
| Box 1. PROM Development | | | | | | | | | | | |
| 1a. PROM design | | | | | | | | | | | |
|  | 1 | Is a clear description provided of the construct to be measured? | VG | VG | **VG** |  |  |  |  |  |  |
|  | 2 | Is the origin of the construct clear: was a theory, conceptual framework or disease model used or clear rationale provided to define the construct to be measured? | VG | VG | **VG** |  |  |  |  |  |  |
|  | 3 | Is a clear description provided of the target population for which the PROM was developed? | VG | VG | **VG** |  |  |  |  |  |  |
|  | 4 | Is a clear description provided of the context of use | VG | VG | **VG** |  |  |  |  |  |  |
|  | 5 | Was the PROM development study performed in a sample representing the target population for which the PROM was developed? | VG | VG | **VG** |  |  |  |  |  |  |
|  | 6 | Was an appropriate qualitative data collection method used to identify relevant items for a new PROM? | VG | D | **VG** |  |  |  |  |  |  |
|  | 7 | Were skilled group moderators/interviewers used? | D | D | **D** |  |  |  |  |  |  |
|  | 8 | Were the group meetings or interviews based on an appropriate topic or interview guide? | A | A | **A** |  |  |  |  |  |  |
|  | 9 | Were the group meetings or interviews recorded and transcribed verbatim? | D | D | **A** |  |  |  |  |  |  |
|  | 10 | Was an appropriate approach used to analyse the data? | VG | A | **VG** |  |  |  |  |  |  |
|  | 11 | Was at least part of the data coded independently? | D | D | **D** |  |  |  |  |  |  |
|  | 12 | Was data collection continued until saturation was reached? | A | D | **A** |  |  |  |  |  |  |
|  | 13 | For quantitative studies (surveys): was the sample size appropriate? |  | I | **D** |  |  |  |  |  |  |
| 1b. Cognitive interview study or pilot test | | | | | | | | | | | |
|  | 14 | Was a cognitive interview study or other pilot test conducted? | VG |  | **VG** |  |  |  |  |  |  |
| General design requirements | 15 | Was the cognitive interview study or other pilot test performed in a sample representing the target population? | VG |  | **VG** |  |  |  |  |  |  |
| Comprehensibility | 16 | Were patients asked about the comprehensibility of the PROM? |  |  |  |  |  |  |  |  |  |
|  | 17 | Were all items tested in their final form? |  |  |  |  |  |  |  |  |  |
|  | 18 | Was an appropriate qualitative method used to assess the comprehensibility of the PROM instructions, items, response options, and recall period? |  |  |  |  |  |  |  |  |  |
|  | 19 | Was each item tested in an appropriate number of patients? |  |  |  |  |  |  |  |  |  |
|  |  | For qualitative studies |  |  |  |  |  |  |  |  |  |
|  |  | For quantitative (survey) studies |  |  |  |  |  |  |  |  |  |
|  | 20 | Were skilled interviewers used? |  |  |  |  |  |  |  |  |  |
|  | 21 | Were the interviews based on an appropriate interview guide? |  |  |  |  |  |  |  |  |  |
|  | 22 | Were the interviews recorded and transcribed verbatim? |  |  |  |  |  |  |  |  |  |
|  | 23 | Was an appropriate approach used to analyse the data? |  |  |  |  |  |  |  |  |  |
|  | 24 | Were at least two researchers involved in the analysis? |  |  |  |  |  |  |  |  |  |
|  | 25 | Were problems regarding the comprehensibility of the PROM instructions, items, response options, and recall period appropriately addressed by adapting the PROM? |  |  |  |  |  |  |  |  |  |
| Comprehensiveness | 26 | Were patients asked about the comprehensiveness of the PROM? |  |  | **VG** |  |  |  |  |  |  |
|  | 27 | Was the final set of items tested? |  |  | **VG** |  |  |  |  |  |  |
|  | 28 | Was an appropriate method used for assessing the comprehensiveness of the PROM? |  |  | **A** |  |  |  |  |  |  |
|  | 29 | Was each item tested in an appropriate number of patients? |  |  |  |  |  |  |  |  |  |
|  |  | For qualitative studies |  |  | **A** |  |  |  |  |  |  |
|  |  | For quantitative (survey) studies |  |  |  |  |  |  |  |  |  |
|  | 30 | Were skilled interviewers used? |  |  | **D** |  |  |  |  |  |  |
|  | 31 | Were the interviews based on an appropriate interview guide? |  |  | **D** |  |  |  |  |  |  |
|  | 32 | Were the interviews recorded and transcribed verbatim? |  |  | **D** |  |  |  |  |  |  |
|  | 33 | Was an appropriate approach used to analyse the data? |  |  | **VG** |  |  |  |  |  |  |
|  | 34 | Were at least two researchers involved in the analysis? |  |  | **D** |  |  |  |  |  |  |
|  | 35 | Were problems regarding the comprehensiveness of the PROM appropriately addressed by adapting the PROM? |  |  | **VG** |  |  |  |  |  |  |
| CONSENSUS | | |  |  | **D** |  |  |  |  |  |  |
| Box 2. Content validity | | | | | | | | | | | |
| 2a. Asking patients about relevance | | | | | | | | | | | |
| Design requirements | 1 | Was an appropriate method used to ask patients whether each item is relevant for their experience with the condition? | VG |  |  |  |  |  |  |  |  |
|  | 2 | Was each item tested in an appropriate number of patients? |  |  |  |  |  |  |  |  |  |
|  |  | For qualitative studies | VG |  |  |  |  |  |  |  |  |
|  |  | For quantitative (survey) studies |  |  |  |  |  |  |  |  |  |
|  | 3 | Were skilled group moderators/interviewers used? | D |  |  |  |  |  |  |  |  |
|  | 4 | Were the group meetings or interviews based on an appropriate topic or interview guide? | D |  |  |  |  |  |  |  |  |
|  | 5 | Were the group meetings or interviews recorded and transcribed verbatim? | D |  |  |  |  |  |  |  |  |
| Analyses | 6 | Was an appropriate approach used to analyse the data? | VG |  |  |  |  |  |  |  |  |
|  | 7 | Were at least two researchers involved in the analysis? | D |  |  |  |  |  |  |  |  |
| 2b. Asking patients about comprehensiveness | | | | | | | | | | | |
| Design requirements | 8 | Was an appropriate method used for assessing the comprehensiveness of the PROM? | VG |  |  |  |  |  |  |  |  |
|  | 9 | Was each item tested in an appropriate number of patients? |  |  |  |  |  |  |  |  |  |
|  |  | For qualitative studies | VG |  |  |  |  |  |  |  |  |
|  |  | For quantitative (survey) studies |  |  |  |  |  |  |  |  |  |
|  | 10 | Were skilled group moderators/interviewers used? | VG |  |  |  |  |  |  |  |  |
|  | 11 | Were the group meetings or interviews based on an appropriate topic or interview guide? | D |  |  |  |  |  |  |  |  |
|  | 12 | Were the group meetings or interviews recorded and transcribed verbatim? | D |  |  |  |  |  |  |  |  |
| Analyses | 13 | Was an appropriate approach used to analyse the data? | VG |  |  |  |  |  |  |  |  |
|  | 14 | Were at least two researchers involved in the analysis? | D |  |  |  |  |  |  |  |  |
| 2c. Asking patients about comprehensibility | | | | | | | | | | | |
| Design requirements | 15 | Was an appropriate qualitative method used for assessing the comprehensibility of the PROM instructions, items, response options, and recall period? |  |  |  |  |  |  |  |  |  |
|  | 16 | Was each item tested in an appropriate number of patients? |  |  |  |  |  |  |  |  |  |
|  |  | For qualitative studies |  |  |  |  |  |  |  |  |  |
|  |  | For quantitative (survey) studies |  |  |  |  |  |  |  |  |  |
|  | 17 | Were skilled group moderators/interviewers used? |  |  |  |  |  |  |  |  |  |
|  | 18 | Were the group meetings or interviews based on an appropriate topic or interview guide? |  |  |  |  |  |  |  |  |  |
|  | 19 | Were the group meetings or interviews recorded and transcribed verbatim? |  |  |  |  |  |  |  |  |  |
| Analyses | 20 | Was an appropriate approach used to analyse the data? |  |  |  |  |  |  |  |  |  |
|  | 21 | Were at least two researchers involved in the analysis? |  |  |  |  |  |  |  |  |  |
| 2d. Asking professionals about relevance | | | | | | | | | | | |
| Design requirements | 22 | Was an appropriate method used to ask professionals whether each item is relevant for the construct of interest? | VG | A | **A** |  |  |  |  |  |  |
|  | 23 | Were professionals from all relevant disciplines included? | VG | VG | **VG** |  |  |  |  |  |  |
|  | 24 | Was each item tested in an appropriate number of professionals? |  |  |  |  |  |  |  |  |  |
|  |  | For qualitative studies | VG | VG | **VG** |  |  |  |  |  |  |
|  |  | For quantitative (survey) studies |  | D |  |  |  |  |  |  |  |
| Analyses | 25 | Was an appropriate approach used to analyse the data? | VG | VG | **VG** |  |  |  |  |  |  |
|  | 26 | Were at least two researchers involved in the analysis? | D | D | **D** |  |  |  |  |  |  |
|  | | | | | | | | | | | |
| Design requirements | 27 | Was an appropriate method used for assessing the comprehensiveness of the PROM? | VG |  |  |  |  |  |  |  |  |
|  | 28 | Were professionals from all relevant disciplines included? | VG |  |  |  |  |  |  |  |  |
|  | 29 | Was each item tested in an appropriate number of professionals? |  |  |  |  |  |  |  |  |  |
|  |  | For qualitative studies | VG |  |  |  |  |  |  |  |  |
|  |  | For quantitative (survey) studies |  |  |  |  |  |  |  |  |  |
| Analyses | 30 | Was an appropriate approach used to analyse the data? | A |  |  |  |  |  |  |  |  |
|  | 31 | Were at least two researchers involved in the analysis? | D |  |  |  |  |  |  |  |  |
| CONSENSUS |  |  |  |  | **D** |  |  |  |  |  |  |
| Box 3. Structural validity | | | | | | | | | | | |
|  |  | Does the scale consist of effect indicators, i.e. is it based on a reflective model? |  | Y | **Y** |  |  |  |  | Y | **Y** |
|  |  | Does the study concern unidimensionality or structural validity? |  | SV | **SV** |  |  |  | SV | SV | **SV** |
| Statistical methods | 1 | For CTT: Was exploratory or confirmatory factor analysis performed? |  | A | **A** |  |  |  | VG | VG | **A** |
|  | 2 | For IRT/Rasch: does the chosen model fit to the research question? |  |  |  |  |  |  |  |  |  |
|  | 3 | Was the sample size included in the analysis A? |  |  |  |  |  |  |  |  |  |
|  |  | For FA |  | A | **A** |  |  |  | VG | VG | **VG** |
|  |  | For Rasch/1PL models |  |  |  |  |  |  |  |  |  |
|  |  | For 2PL parametric IRT models OR Mokken scale analysis |  |  |  |  |  |  |  |  |  |
| Other | 4 | Were there any other important flaws in the design or statistical methods of the study? |  | VG | **VG** |  |  |  |  | VG | **VG** |
| CONSENSUS | | |  |  | **A** |  |  |  |  |  | **A** |
| Box 4. Internal consistency | | | | | | | | | | | |
|  |  | Does the scale consist of effect indicators, i.e. is it based on a reflective model? |  | Y | **Y** | Y | Y | **Y** |  | Y | Y |
| Design requirements | 1 | Was an internal consistency statistic calculated for each unidimensional scale or subscale separately? | I | I | **I** | I | I | **I** | D | I | I |
| Statistical methods | 2 | For continuous scores: Was Cronbach’s alpha or omega calculated? | VG | VG | **VG** | VG | VG | **VG** | VG | VG | VG |
|  | 3 | For dichotomous scores: Was Cronbach’s alpha or KR-20 calculated? |  |  |  |  |  |  |  |  |  |
|  | 4 | For IRT-based scores: Was standard error of the theta (SE (θ)) or reliability coefficient of estimated latent trait value (index of (subject or item) separation) calculated? |  |  |  |  |  |  |  |  |  |
| Other | 5 | Were there any other important flaws in the design or statistical methods of the study? |  | VG | **VG** |  | VG | **VG** |  | VG | VG |
| CONSENSUS | | |  |  | **I** |  |  | **I** |  |  | I |
| Box 5. Cross-cultural validity/Measurement invariance | | | | | | | | | | | |
| Design requirements | 1 | Were the samples similar for relevant characteristics except for the group variable? |  |  |  |  |  |  | VG |  |  |
| Statistical methods | 2 | Was an appropriate approach used to analyse the data? |  |  |  |  |  |  | D |  |  |
|  | 3 | Was the sample size included in the analysis A? |  |  |  |  |  |  |  |  |  |
|  |  | For Regression analyses or IRT/Rasch based analyses |  |  |  |  |  |  | D |  |  |
|  |  | For MGCFA* |  |  |  |  |  |  | A |  |  |
| Other | 4 | Were there any other important flaws in the design or statistical methods of the study? |  |  |  |  |  |  |  |  |  |
| CONSENSUS | | |  |  |  |  |  |  |  |  |  |
| Box 6. Reliability | | | | | | | | | | | |
| Design requirements | 1 | Were patients stable in the interim period on the construct to be measured? |  |  |  |  |  |  |  |  |  |
|  | 2 | Was the time interval appropriate? |  |  |  |  |  |  |  |  |  |
|  | 3 | Were the test conditions similar for the measurements? e.g. type of administration, environment, instructions |  |  |  |  |  |  |  |  |  |
| Statistical methods | 4 | For continuous scores: Was an intraclass correlation coefficient (ICC) calculated? |  |  |  |  |  |  |  |  |  |
|  | 5 | For dichotomous/nominal/ordinal scores: Was kappa calculated? |  |  |  |  |  |  |  |  |  |
|  | 6 | For ordinal scores: Was a weighted kappa calculated? |  |  |  |  |  |  |  |  |  |
|  | 7 | For ordinal scores: Was the weighting scheme described? e.g. linear, quadratic |  |  |  |  |  |  |  |  |  |
| Other | 8 | Were there any other important flaws in the design or statistical methods of the study? |  |  |  |  |  |  |  |  |  |
| CONSENSUS | | |  |  |  |  |  |  |  |  |  |
| Box 7. Measurement error | | | | | | | | | | | |
| Design requirements | 1 | Were patients stable in the interim period on the construct to be measured? |  |  |  |  |  |  |  |  |  |
|  | 2 | Was the time interval appropriate? |  |  |  |  |  |  |  |  |  |
|  | 3 | Were the test conditions similar for the measurements? e.g. type of administration, environment, instructions |  |  |  |  |  |  |  |  |  |
| Statistical methods | 4 | For continuous scores: Was the Standard Error of Measurement (SEM), Smallest Detectable Change (SDC) or Limits of Agreement (LoA) calculated? |  |  |  |  |  |  |  |  |  |
|  | 5 | For dichotomous/nominal/ordinal scores: Was the percentage (positive and negative) agreement calculated? |  |  |  |  |  |  |  |  |  |
| Other | 6 | Were there any other important flaws in the design or statistical methods of the study? |  |  |  |  |  |  |  |  |  |
| CONSENSUS | | |  |  |  |  |  |  |  |  |  |
| Box 8. Criterion validity | | | | | | | | | | | |
| Statistical methods | 1 | For continuous scores: Were correlations, or the area under the receiver operating curve calculated? |  |  |  |  |  |  |  |  |  |
|  | 2 | For dichotomous scores: Were sensitivity and specificity determined? |  |  |  |  |  |  |  |  |  |
| Other | 3 | Were there any other important flaws in the design or statistical methods of the study? |  |  |  |  |  |  |  |  |  |
| CONSENSUS | | |  |  |  |  |  |  |  |  |  |
| Box 9. Hypotheses testing for construct validity | | | | | | | | | | | |
| 9a. Comparison with other outcome measurement instruments (convergent validity) | | | | | | | | | | | |
| Design requirements | 1 | Is it clear what the comparator instrument(s) measure(s)? | VG | VG | **VG** | VG | VG | **VG** | VG | VG | **VG** |
|  | 2 | Were the measurement properties of the comparator instrument(s) sufficient? | VG | D | **A** | I | D | **A** | VG | D | **A** |
| Statistical methods | 3 | Was the statistical method appropriate for the hypotheses to be tested? | VG | A | **A** | VG | A | **A** | VG | D | **D** |
| Other | 4 | Were there any other important flaws in the design or statistical methods of the study? | VG | VG | **D** |  | VG | **D** |  | VG | **D** |
| CONSENSUS | | |  |  | **D** |  |  | **D** |  |  | **D** |
| 9b. Comparison between subgroups (discriminative or known-groups validity) | | | | | | | | | | | |
| Design requirements | 5 | Was an A description provided of important characteristics of the subgroups? |  |  |  |  |  |  |  | A |  |
| Statistical methods | 6 | Was the statistical method appropriate for the hypotheses to be tested? |  |  |  |  |  |  |  | D |  |
| Other | 7 | Were there any other important flaws in the design or statistical methods of the study? |  |  |  |  |  |  |  | I |  |
| CONSENSUS | | |  |  |  |  |  |  |  |  |  |
| Box 10. Responsiveness | | | | | | | | | | | |
| 10a. Criterion approach (i.e. comparison to a gold standard) | | | | | | | | | | | |
| Statistical methods | 1 | For continuous scores: Were correlations between change scores, or the area under the Receiver Operator Curve (ROC) curve calculated? |  |  |  |  |  |  |  |  |  |
|  | 2 | For dichotomous scales: Were sensitivity and specificity (changed versus not changed) determined? |  |  |  |  |  |  |  |  |  |
| Other | 3 | Were there any other important flaws in the design or statistical methods of the study? |  |  |  |  |  |  |  |  |  |
| CONSENSUS | | |  |  |  |  |  |  |  |  |  |
| 10b. Construct approach (i.e. hypotheses testing; comparison with other outcome measurement instruments) | | | | | | | | | | | |
| Design requirements | 4 | Is it clear what the comparator instrument(s) measure(s)? | VG |  |  | VG |  |  | VG |  |  |
|  | 5 | Were the measurement properties of the comparator instrument(s) sufficient? | VG |  |  | VG |  |  | VG |  |  |
| Statistical methods | 6 | Was the statistical method appropriate for the hypotheses to be tested? | A |  |  | D |  |  | VG |  |  |
| Other | 7 | Were there any other important flaws in the design or statistical methods of the study? | VG |  |  | D |  |  |  |  |  |
| CONSENSUS | | |  |  |  |  |  |  |  |  |  |
| 10c. Construct approach (i.e. hypotheses testing: comparison between subgroups) | | | | | | | | | | | |
| Design requirements | 8 | Was an A description provided of important characteristics of the subgroups? |  |  |  |  |  |  |  |  |  |
| Statistical methods | 9 | Was the statistical method appropriate for the hypotheses to be tested? |  |  |  |  |  |  |  |  |  |
| Other | 10 | Were there any other important flaws in the design or statistical methods of the study? |  |  |  |  |  |  |  |  |  |
| CONSENSUS | | |  |  |  |  |  |  |  |  |  |
| 10d. Construct approach (i.e. hypotheses testing: before and after intervention) | | | | | | | | | | | |
| Design requirements | 11 | Was an A description provided of the intervention given? |  |  |  |  | D | **D** |  |  |  |
| Statistical methods | 12 | Was the statistical method appropriate for the hypotheses to be tested? |  |  |  |  | VG | **VG** |  |  |  |
| Other | 13 | Were there any other important flaws in the design or statistical methods of the study? |  |  |  |  | VG | **VG** |  |  |  |
| CONSENSUS | | |  |  |  |  |  | **D** |  |  |  |
| % | | | **26.32%** | | | **26.67%** | | | **17.39%** | | |

A: adequate; C: consensus; D: doubtful; I: inadequate; R1: Reviewer 1; R2: Reviewer 2; SV: structural validity; VG: very good; Y: yes

| MYMOP1 | | | | | | | | | | | | | | | | | |
| --- | --- | --- | --- | --- | --- | --- | --- | --- | --- | --- | --- | --- | --- | --- | --- | --- | --- |
| COSMIN Risk of Bias Checklist | | | Paterson, 1996 | | | Paterson & Britten, 2000 | | | Paterson et al., 2000 | | | Chung et al., 2010 | | | Murphy et al., 2018 | | |
|  | Item | Requirements | R1 | R2 | **C** | R1 | R2 | **C** | R1 | R2 | **C** | R1 | R2 | **C** | R1 | R2 | **C** |
| Box 1. PROM Development | | | | | | | | | | | | | | | | | |
| 1a. PROM design | | | | | | | | | | | | | | | | | |
|  | 1 | Is a clear description provided of the construct to be measured? |  | I |  |  |  |  |  |  |  |  |  |  |  |  |  |
|  | 2 | Is the origin of the construct clear: was a theory, conceptual framework or disease model used or clear rationale provided to define the construct to be measured? |  | D |  |  |  |  |  |  |  |  |  |  |  |  |  |
|  | 3 | Is a clear description provided of the target population for which the PROM was developed? |  | I |  |  |  |  |  |  |  |  |  |  |  |  |  |
|  | 4 | Is a clear description provided of the context of use |  | D |  |  |  |  |  |  |  |  |  |  |  |  |  |
|  | 5 | Was the PROM development study performed in a sample representing the target population for which the PROM was developed? |  | VG |  |  |  |  |  |  |  |  |  |  |  |  |  |
|  | 6 | Was an appropriate qualitative data collection method used to identify relevant items for a new PROM? |  | D |  |  |  |  |  |  |  |  |  |  |  |  |  |
|  | 7 | Were skilled group moderators/interviewers used? |  | D |  |  |  |  |  |  |  |  |  |  |  |  |  |
|  | 8 | Were the group meetings or interviews based on an appropriate topic or interview guide? |  |  |  |  |  |  |  |  |  |  |  |  |  |  |  |
|  | 9 | Were the group meetings or interviews recorded and transcribed verbatim? |  |  |  |  |  |  |  |  |  |  |  |  |  |  |  |
|  | 10 | Was an appropriate approach used to analyse the data? |  |  |  |  |  |  |  |  |  |  |  |  |  |  |  |
|  | 11 | Was at least part of the data coded independently? |  |  |  |  |  |  |  |  |  |  |  |  |  |  |  |
|  | 12 | Was data collection continued until saturation was reached? |  |  |  |  |  |  |  |  |  |  |  |  |  |  |  |
|  | 13 | For quantitative studies (surveys): was the sample size appropriate? |  |  |  |  |  |  |  |  |  |  |  |  |  |  |  |
| 1b. Cognitive interview study or pilot test | | | | | | | | | | | | | | | | | |
|  | 14 | Was a cognitive interview study or other pilot test conducted? |  | I |  |  |  |  |  |  |  |  |  |  |  |  |  |
| General design requirements | 15 | Was the cognitive interview study or other pilot test performed in a sample representing the target population? |  |  |  |  |  |  |  |  |  |  |  |  |  |  |  |
| Comprehensibility | 16 | Were patients asked about the comprehensibility of the PROM? |  |  |  |  |  |  |  |  |  |  |  |  |  |  |  |
|  | 17 | Were all items tested in their fIl form? |  |  |  |  |  |  |  |  |  |  |  |  |  |  |  |
|  | 18 | Was an appropriate qualitative method used to assess the comprehensibility of the PROM instructions, items, response options, and recall period? |  |  |  |  |  |  |  |  |  |  |  |  |  |  |  |
|  | 19 | Was each item tested in an appropriate number of patients? |  |  |  |  |  |  |  |  |  |  |  |  |  |  |  |
|  |  | For qualitative studies |  |  |  |  |  |  |  |  |  |  |  |  |  |  |  |
|  |  | For quantitative (survey) studies |  |  |  |  |  |  |  |  |  |  |  |  |  |  |  |
|  | 20 | Were skilled interviewers used? |  |  |  |  |  |  |  |  |  |  |  |  |  |  |  |
|  | 21 | Were the interviews based on an appropriate interview guide? |  |  |  |  |  |  |  |  |  |  |  |  |  |  |  |
|  | 22 | Were the interviews recorded and transcribed verbatim? |  |  |  |  |  |  |  |  |  |  |  |  |  |  |  |
|  | 23 | Was an appropriate approach used to analyse the data? |  |  |  |  |  |  |  |  |  |  |  |  |  |  |  |
|  | 24 | Were at least two researchers involved in the analysis? |  |  |  |  |  |  |  |  |  |  |  |  |  |  |  |
|  | 25 | Were problems regarding the comprehensibility of the PROM instructions, items, response options, and recall period appropriately addressed by adapting the PROM? |  |  |  |  |  |  |  |  |  |  |  |  |  |  |  |
| Comprehensiveness | 26 | Were patients asked about the comprehensiveness of the PROM? |  |  |  |  |  |  |  |  |  |  |  |  |  |  |  |
|  | 27 | Was the final set of items tested? |  |  |  |  |  |  |  |  |  |  |  |  |  |  |  |
|  | 28 | Was an appropriate method used for assessing the comprehensiveness of the PROM? |  |  |  |  |  |  |  |  |  |  |  |  |  |  |  |
|  | 29 | Was each item tested in an appropriate number of patients? |  |  |  |  |  |  |  |  |  |  |  |  |  |  |  |
|  |  | For qualitative studies |  |  |  |  |  |  |  |  |  |  |  |  |  |  |  |
|  |  | For quantitative (survey) studies |  |  |  |  |  |  |  |  |  |  |  |  |  |  |  |
|  | 30 | Were skilled interviewers used? |  |  |  |  |  |  |  |  |  |  |  |  |  |  |  |
|  | 31 | Were the interviews based on an appropriate interview guide? |  |  |  |  |  |  |  |  |  |  |  |  |  |  |  |
|  | 32 | Were the interviews recorded and transcribed verbatim? |  |  |  |  |  |  |  |  |  |  |  |  |  |  |  |
|  | 33 | Was an appropriate approach used to analyse the data? |  |  |  |  |  |  |  |  |  |  |  |  |  |  |  |
|  | 34 | Were at least two researchers involved in the analysis? |  |  |  |  |  |  |  |  |  |  |  |  |  |  |  |
|  | 35 | Were problems regarding the comprehensiveness of the PROM appropriately addressed by adapting the PROM? |  |  |  |  |  |  |  |  |  |  |  |  |  |  |  |
| CONSENSUS | | |  |  |  |  |  |  |  |  |  |  |  |  |  |  |  |
| Box 2. Content validity | | | | | | | | | | | | | | | | | |
| 2a. Asking patients about relevance | | | | | | | | | | | | | | | | | |
| Design requirements | 1 | Was an appropriate method used to ask patients whether each item is relevant for their experience with the condition? |  | I |  | VG | A | **A** |  |  |  |  |  |  | D | D | **D** |
|  | 2 | Was each item tested in an appropriate number of patients? |  |  |  |  |  |  |  |  |  |  |  |  |  |  |  |
|  |  | For qualitative studies |  |  |  | VG | VG | **VG** |  |  |  |  |  |  | A | VG | **VG** |
|  |  | For quantitative (survey) studies |  |  |  |  |  |  |  |  |  |  |  |  |  |  |  |
|  | 3 | Were skilled group moderators/interviewers used? |  |  |  | VG | D | **A** |  |  |  |  |  |  | D | VG | **A** |
|  | 4 | Were the group meetings or interviews based on an appropriate topic or interview guide? |  |  |  | A | A | **A** |  |  |  |  |  |  | VG | VG | **VG** |
|  | 5 | Were the group meetings or interviews recorded and transcribed verbatim? |  |  |  | VG | VG | **VG** |  |  |  |  |  |  | VG | VG | **VG** |
| Analyses | 6 | Was an appropriate approach used to analyse the data? |  |  |  | VG | VG | **VG** |  |  |  |  |  |  | VG | VG | **VG** |
|  | 7 | Were at least two researchers involved in the analysis? |  |  |  | A | D | **A** |  |  |  |  |  |  | VG | VG | **VG** |
| 2b. Asking patients about comprehensiveness | | | | | | | | | | | | | | | | | |
| Design requirements | 8 | Was an appropriate method used for assessing the comprehensiveness of the PROM? |  | I |  | VG | A | **A** |  |  |  |  |  |  | VG |  |  |
|  | 9 | Was each item tested in an appropriate number of patients? |  |  |  |  |  |  |  |  |  |  |  |  |  |  |  |
|  |  | For qualitative studies |  |  |  | VG | VG | **VG** |  |  |  |  |  |  | VG |  |  |
|  |  | For quantitative (survey) studies |  |  |  |  |  |  |  |  |  |  |  |  |  |  |  |
|  | 10 | Were skilled group moderators/interviewers used? |  |  |  | VG | D | **A** |  |  |  |  |  |  | D |  |  |
|  | 11 | Were the group meetings or interviews based on an appropriate topic or interview guide? |  |  |  | VG | A | **A** |  |  |  |  |  |  | VG |  |  |
|  | 12 | Were the group meetings or interviews recorded and transcribed verbatim? |  |  |  | VG | VG | **VG** |  |  |  |  |  |  | VG |  |  |
| Analyses | 13 | Was an appropriate approach used to analyse the data? |  |  |  | VG | VG | **VG** |  |  |  |  |  |  | VG |  |  |
|  | 14 | Were at least two researchers involved in the analysis? |  |  |  | A | D | **A** |  |  |  |  |  |  | D |  |  |
| 2c. Asking patients about comprehensibility | | | | | | | | | | | | | | | | | |
| Design requirements | 15 | Was an appropriate qualitative method used for assessing the comprehensibility of the PROM instructions, items, response options, and recall period? |  | I |  | VG | D | **A** |  |  |  | VG | VG | VG | A | VG | **VG** |
|  | 16 | Was each item tested in an appropriate number of patients? |  |  |  |  |  |  |  |  |  |  |  |  |  |  |  |
|  |  | For qualitative studies |  |  |  | VG | VG | **VG** |  |  |  | VG | VG | VG | VG | VG | **VG** |
|  |  | For quantitative (survey) studies |  |  |  |  |  |  |  |  |  |  |  |  |  |  |  |
|  | 17 | Were skilled group moderators/interviewers used? |  |  |  | VG | D | **A** |  |  |  | D | D | D | D | VG | **A** |
|  | 18 | Were the group meetings or interviews based on an appropriate topic or interview guide? |  |  |  | VG | A | **A** |  |  |  | D | D | D | VG | VG | **VG** |
|  | 19 | Were the group meetings or interviews recorded and transcribed verbatim? |  |  |  | VG | VG | **VG** |  |  |  | D | D | D | VG | VG | **VG** |
| Analyses | 20 | Was an appropriate approach used to analyse the data? |  |  |  | VG | VG | **VG** |  |  |  | D | D | D | VG | VG | **VG** |
|  | 21 | Were at least two researchers involved in the analysis? |  |  |  | A | D | **A** |  |  |  | D | A | A | D | VG | **VG** |
| 2d. Asking professionals about relevance | | | | | | | | | | | | | | | | | |
| Design requirements | 22 | Was an appropriate method used to ask professionals whether each item is relevant for the construct of interest? |  | D |  |  | I |  |  |  |  |  |  |  | I |  |  |
|  | 23 | Were professionals from all relevant disciplines included? |  |  |  |  |  |  |  |  |  |  |  |  |  |  |  |
|  | 24 | Was each item tested in an appropriate number of professionals? |  |  |  |  |  |  |  |  |  |  |  |  |  |  |  |
|  |  | For qualitative studies |  |  |  |  |  |  |  |  |  |  |  |  |  |  |  |
|  |  | For quantitative (survey) studies |  |  |  |  |  |  |  |  |  |  |  |  |  |  |  |
| Analyses | 25 | Was an appropriate approach used to analyse the data? |  |  |  |  |  |  |  |  |  |  |  |  |  |  |  |
|  | 26 | Were at least two researchers involved in the analysis? |  |  |  |  |  |  |  |  |  |  |  |  |  |  |  |
| 2e. Asking professionals about comprehensiveness | | | | | | | | | | | | | | | | | |
| Design requirements | 27 | Was an appropriate method used for assessing the comprehensiveness of the PROM? |  | D |  |  | I |  |  |  |  |  |  |  | I |  |  |
|  | 28 | Were professionals from all relevant disciplines included? |  |  |  |  |  |  |  |  |  |  |  |  |  |  |  |
|  | 29 | Was each item tested in an appropriate number of professionals? |  |  |  |  |  |  |  |  |  |  |  |  |  |  |  |
|  |  | For qualitative studies |  |  |  |  |  |  |  |  |  |  |  |  |  |  |  |
|  |  | For quantitative (survey) studies |  |  |  |  |  |  |  |  |  |  |  |  |  |  |  |
| Analyses | 30 | Was an appropriate approach used to analyse the data? |  |  |  |  |  |  |  |  |  |  |  |  |  |  |  |
|  | 31 | Were at least two researchers involved in the analysis? |  |  |  |  |  |  |  |  |  |  |  |  |  |  |  |
| CONSENSUS | | |  |  |  |  |  | **A** |  |  |  |  |  | **D** |  |  | **D** |
| Box 3. Structural validity | | | | | | | | | | | | | | | | | |
|  |  | Does the scale consist of effect indicators, i.e. is it based on a reflective model? |  |  |  |  |  |  |  |  |  |  |  |  |  |  |  |
|  |  | Does the study concern unidimensionality or structural validity? |  |  |  |  |  |  |  |  |  |  |  |  |  |  |  |
| Statistical methods | 1 | For CTT: Was exploratory or confirmatory factor analysis performed? |  |  |  |  |  |  |  |  |  |  |  |  |  |  |  |
|  | 2 | For IRT/Rasch: does the chosen model fit to the research question? |  |  |  |  |  |  |  |  |  |  |  |  |  |  |  |
|  | 3 | Was the sample size included in the analysis A? |  |  |  |  |  |  |  |  |  |  |  |  |  |  |  |
|  |  | For FA |  |  |  |  |  |  |  |  |  |  |  |  |  |  |  |
|  |  | For Rasch/1PL models |  |  |  |  |  |  |  |  |  |  |  |  |  |  |  |
|  |  | For 2PL parametric IRT models OR Mokken scale analysis |  |  |  |  |  |  |  |  |  |  |  |  |  |  |  |
| Other | 4 | Were there any other important flaws in the design or statistical methods of the study? |  |  |  |  |  |  |  |  |  |  |  |  |  |  |  |
| CONSENSUS | | |  |  |  |  |  |  |  |  |  |  |  |  |  |  |  |
| Box 4. Internal consistency | | | | | | | | | | | | | | | | | |
|  |  | Does the scale consist of effect indicators, i.e. is it based on a reflective model? |  |  |  |  |  |  |  |  |  |  |  |  |  |  |  |
| Design requirements | 1 | Was an internal consistency statistic calculated for each unidimensional scale or subscale separately? |  |  |  |  |  |  |  |  |  |  |  |  |  |  |  |
| Statistical methods | 2 | For continuous scores: Was Cronbach’s alpha or omega calculated? |  |  |  |  |  |  |  |  |  |  |  |  |  |  |  |
|  | 3 | For dichotomous scores: Was Cronbach’s alpha or KR-20 calculated? |  |  |  |  |  |  |  |  |  |  |  |  |  |  |  |
|  | 4 | For IRT-based scores: Was standard error of the theta (SE (θ)) or reliability coefficient of estimated latent trait value (index of (subject or item) separation) calculated? |  |  |  |  |  |  |  |  |  |  |  |  |  |  |  |
| Other | 5 | Were there any other important flaws in the design or statistical methods of the study? |  |  |  |  |  |  |  |  |  |  |  |  |  |  |  |
| CONSENSUS | | |  |  |  |  |  |  |  |  |  |  |  |  |  |  |  |
| Box 5. Cross-cultural validity/Measurement invariance | | | | | | | | | | | | | | | | | |
| Design requirements | 1 | Were the samples similar for relevant characteristics except for the group variable? |  |  |  |  |  |  |  |  |  |  |  |  |  |  |  |
| Statistical methods | 2 | Was an appropriate approach used to analyse the data? |  |  |  |  |  |  |  |  |  |  |  |  |  |  |  |
|  | 3 | Was the sample size included in the analysis A? |  |  |  |  |  |  |  |  |  |  |  |  |  |  |  |
|  |  | For Regression analyses or IRT/Rasch based analyses |  |  |  |  |  |  |  |  |  |  |  |  |  |  |  |
|  |  | For MGCFA* |  |  |  |  |  |  |  |  |  |  |  |  |  |  |  |
| Other | 4 | Were there any other important flaws in the design or statistical methods of the study? |  |  |  |  |  |  |  |  |  |  |  |  |  |  |  |
| CONSENSUS | | |  |  |  |  |  |  |  |  |  |  |  |  |  |  |  |
| Box 6. Reliability | | | | | | | | | | | | | | | | | |
| Design requirements | 1 | Were patients stable in the interim period on the construct to be measured? |  |  |  |  |  |  |  |  |  |  |  |  |  |  |  |
|  | 2 | Was the time interval appropriate? |  |  |  |  |  |  |  |  |  |  |  |  |  |  |  |
|  | 3 | Were the test conditions similar for the measurements? e.g. type of administration, environment, instructions |  |  |  |  |  |  |  |  |  |  |  |  |  |  |  |
| Statistical methods | 4 | For continuous scores: Was an intraclass correlation coefficient (ICC) calculated? |  |  |  |  |  |  |  |  |  |  |  |  |  |  |  |
|  | 5 | For dichotomous/nominal/ordinal scores: Was kappa calculated? |  |  |  |  |  |  |  |  |  |  |  |  |  |  |  |
|  | 6 | For ordinal scores: Was a weighted kappa calculated? |  |  |  |  |  |  |  |  |  |  |  |  |  |  |  |
|  | 7 | For ordinal scores: Was the weighting scheme described? e.g. linear, quadratic |  |  |  |  |  |  |  |  |  |  |  |  |  |  |  |
| Other | 8 | Were there any other important flaws in the design or statistical methods of the study? |  |  |  |  |  |  |  |  |  |  |  |  |  |  |  |
| CONSENSUS | | |  |  |  |  |  |  |  |  |  |  |  |  |  |  |  |
| Box 7. Measurement error | | | | | | | | | | | | | | | | | |
| Design requirements | 1 | Were patients stable in the interim period on the construct to be measured? |  |  |  |  |  |  |  |  |  |  |  |  |  |  |  |
|  | 2 | Was the time interval appropriate? |  |  |  |  |  |  |  |  |  |  |  |  |  |  |  |
|  | 3 | Were the test conditions similar for the measurements? e.g. type of administration, environment, instructions |  |  |  |  |  |  |  |  |  |  |  |  |  |  |  |
| Statistical methods | 4 | For continuous scores: Was the Standard Error of Measurement (SEM), Smallest Detectable Change (SDC) or Limits of Agreement (LoA) calculated? |  |  |  |  |  |  |  |  |  |  |  |  |  |  |  |
|  | 5 | For dichotomous/nominal/ordinal scores: Was the percentage (positive and negative) agreement calculated? |  |  |  |  |  |  |  |  |  |  |  |  |  |  |  |
| Other | 6 | Were there any other important flaws in the design or statistical methods of the study? |  |  |  |  |  |  |  |  |  |  |  |  |  |  |  |
| CONSENSUS | | |  |  |  |  |  |  |  |  |  |  |  |  |  |  |  |
| Box 8. Criterion validity | | | | | | | | | | | | | | | | | |
| Statistical methods | 1 | For continuous scores: Were correlations, or the area under the receiver operating curve calculated? |  |  |  |  |  |  |  |  |  | VG |  |  |  |  |  |
|  | 2 | For dichotomous scores: Were sensitivity and specificity determined? |  |  |  |  |  |  |  |  |  |  |  |  |  |  |  |
| Other | 3 | Were there any other important flaws in the design or statistical methods of the study? |  |  |  |  |  |  |  |  |  |  |  |  |  |  |  |
| CONSENSUS | | |  |  |  |  |  |  |  |  |  |  |  |  |  |  |  |
| Box 9. Hypotheses testing for construct validity | | | | | | | | | | | | | | | | | |
| 9a. Comparison with other outcome measurement instruments (convergent validity) | | | | | | | | | | | | | | | | | |
| Design requirements | 1 | Is it clear what the comparator instrument(s) measure(s)? | VG | VG | **VG** |  |  |  |  |  |  | VG | VG | **VG** |  |  |  |
|  | 2 | Were the measurement properties of the comparator instrument(s) sufficient? | VG | VG | **VG** |  |  |  |  |  |  | VG | VG | **VG** |  |  |  |
| Statistical methods | 3 | Was the statistical method appropriate for the hypotheses to be tested? | VG |  | **VG** |  |  |  |  |  |  | VG | VG | **VG** |  |  |  |
| Other | 4 | Were there any other important flaws in the design or statistical methods of the study? | VG | VG | **VG** |  |  |  |  |  |  | VG | VG | **VG** |  |  |  |
| CONSENSUS | | |  |  | **VG** |  |  |  |  |  |  |  |  | **VG** |  |  |  |
| 9b. Comparison between subgroups (discriminative or known-groups validity) | | | | | | | | | | | | | | | | | |
| Design requirements | 5 | Was an A description provided of important characteristics of the subgroups? |  |  |  |  |  |  | VG |  |  |  |  |  |  |  |  |
| Statistical methods | 6 | Was the statistical method appropriate for the hypotheses to be tested? |  |  |  |  |  |  | VG |  |  |  |  |  |  |  |  |
| Other | 7 | Were there any other important flaws in the design or statistical methods of the study? |  |  |  |  |  |  |  |  |  |  |  |  |  |  |  |
| CONSENSUS | | |  |  |  |  |  |  |  |  |  |  |  |  |  |  |  |
| Box 10. Responsiveness | | | | | | | | | | | | | | | | | |
| 10a. Criterion approach (i.e. comparison to a gold standard) | | | | | | | | | | | | | | | | | |
| Statistical methods | 1 | For continuous scores: Were correlations between change scores, or the area under the Receiver Operator Curve (ROC) curve calculated? |  | VG |  |  |  |  |  |  |  |  |  |  |  |  |  |
|  | 2 | For dichotomous scales: Were sensitivity and specificity (changed versus not changed) determined? |  |  |  |  |  |  |  |  |  |  |  |  |  |  |  |
| Other | 3 | Were there any other important flaws in the design or statistical methods of the study? |  | VG |  |  |  |  |  |  |  |  |  |  |  |  |  |
| CONSENSUS | | |  |  |  |  |  |  |  |  |  |  |  |  |  |  |  |
| 10b. Construct approach (i.e. hypotheses testing; comparison with other outcome measurement instruments) | | | | | | | | | | | | | | | | | |
| Design requirements | 4 | Is it clear what the comparator instrument(s) measure(s)? | VG |  | **VG** |  |  |  | VG | VG | **VG** | VG | VG | **VG** |  |  |  |
|  | 5 | Were the measurement properties of the comparator instrument(s) sufficient? | VG |  | **VG** |  |  |  | A | VG | **A** | VG | A | **VG** |  |  |  |
| Statistical methods | 6 | Was the statistical method appropriate for the hypotheses to be tested? | D |  | **D** |  |  |  | D | VG | **D** | VG | A | **VG** |  |  |  |
| Other | 7 | Were there any other important flaws in the design or statistical methods of the study? | VG |  | **VG** |  |  |  | VG |  | **VG** | VG | VG | **VG** |  |  |  |
| CONSENSUS | | |  |  | **D** |  |  |  |  |  | **D** |  |  | **VG** |  |  |  |
| 10c. Construct approach (i.e. hypotheses testing: comparison between subgroups) | | | | | | | | | | | | | | | | | |
| Design requirements | 8 | Was an A description provided of important characteristics of the subgroups? | A |  | **A** |  |  |  | A | VG | **A** |  |  |  |  |  |  |
| Statistical methods | 9 | Was the statistical method appropriate for the hypotheses to be tested? | VG |  | **VG** |  |  |  | A | VG | **A** |  |  |  |  |  |  |
| Other | 10 | Were there any other important flaws in the design or statistical methods of the study? | VG |  | **VG** |  |  |  | VG |  | **VG** |  |  |  |  |  |  |
| CONSENSUS | | |  |  | **A** |  |  |  |  |  | **A** |  |  |  |  |  |  |
| 10d. Construct approach (i.e. hypotheses testing: before and after intervention) | | | | | | | | | | | | | | | | | |
| Design requirements | 11 | Was an A description provided of the intervention given? | D |  | **D** |  |  |  | D |  | **D** |  |  |  |  |  |  |
| Statistical methods | 12 | Was the statistical method appropriate for the hypotheses to be tested? | VG |  | **VG** |  |  |  | A |  | **A** |  |  |  |  |  |  |
| Other | 13 | Were there any other important flaws in the design or statistical methods of the study? | VG |  | **VG** |  |  |  | VG |  | **VG** |  |  |  |  |  |  |
| CONSENSUS | | |  |  | **D** |  |  |  |  |  | **D** |  |  |  |  |  |  |
| % | | | **10.34%** | | | **43.48%** | | | **8.33%** | | | **75.00%** | | | **39.13%** | | |

A: adequate; C: consensus; D: doubtful; I: inadequate; N/A: not applicable; R1: Reviewer 1; R2: Reviewer 2; SV: structural validity

| MYMOP 2 | | | | | | | | | | | |
| --- | --- | --- | --- | --- | --- | --- | --- | --- | --- | --- | --- |
| COSMIN Risk of Bias Checklist | | | Peterson, 2004 | | | Herman et al., 2014 | | | Ersoy et al., 2013 | | |
|  | Item | Requirements | R1 | R2 | **C** | R1 | R2 | **C** | R1 | R2 | **C** |
| Box 1. PROM Development | | | | | | | | | | | |
| 1a. PROM design | | | | | | | | | | | |
|  | 1 | Is a clear description provided of the construct to be measured? |  |  |  |  |  |  |  |  |  |
|  | 2 | Is the origin of the construct clear: was a theory, conceptual framework or disease model used or clear rationale provided to define the construct to be measured? |  |  |  |  |  |  |  |  |  |
|  | 3 | Is a clear description provided of the target population for which the PROM was developed? |  |  |  |  |  |  |  |  |  |
|  | 4 | Is a clear description provided of the context of use |  |  |  |  |  |  |  |  |  |
|  | 5 | Was the PROM development study performed in a sample representing the target population for which the PROM was developed? |  |  |  |  |  |  |  |  |  |
|  | 6 | Was an appropriate qualitative data collection method used to identify relevant items for a new PROM? |  |  |  |  |  |  |  |  |  |
|  | 7 | Were skilled group moderators/interviewers used? |  |  |  |  |  |  |  |  |  |
|  | 8 | Were the group meetings or interviews based on an appropriate topic or interview guide? |  |  |  |  |  |  |  |  |  |
|  | 9 | Were the group meetings or interviews recorded and transcribed verbatim? |  |  |  |  |  |  |  |  |  |
|  | 10 | Was an appropriate approach used to analyse the data? |  |  |  |  |  |  |  |  |  |
|  | 11 | Was at least part of the data coded independently? |  |  |  |  |  |  |  |  |  |
|  | 12 | Was data collection continued until saturation was reached? |  |  |  |  |  |  |  |  |  |
|  | 13 | For quantitative studies (surveys): was the sample size appropriate? |  |  |  |  |  |  |  |  |  |
| 1b. Cognitive interview study or pilot test | | | | | | | | | | | |
|  | 14 | Was a cognitive interview study or other pilot test conducted? |  |  |  |  |  |  |  |  |  |
| General design requirements | 15 | Was the cognitive interview study or other pilot test performed in a sample representing the target population? |  |  |  |  |  |  |  |  |  |
| Comprehensibility | 16 | Were patients asked about the comprehensibility of the PROM? |  |  |  |  |  |  |  |  |  |
|  | 17 | Were all items tested in their final form? |  |  |  |  |  |  |  |  |  |
|  | 18 | Was an appropriate qualitative method used to assess the comprehensibility of the PROM instructions, items, response options, and recall period? |  |  |  |  |  |  |  |  |  |
|  | 19 | Was each item tested in an appropriate number of patients? |  |  |  |  |  |  |  |  |  |
|  |  | For qualitative studies |  |  |  |  |  |  |  |  |  |
|  |  | For quantitative (survey) studies |  |  |  |  |  |  |  |  |  |
|  | 20 | Were skilled interviewers used? |  |  |  |  |  |  |  |  |  |
|  | 21 | Were the interviews based on an appropriate interview guide? |  |  |  |  |  |  |  |  |  |
|  | 22 | Were the interviews recorded and transcribed verbatim? |  |  |  |  |  |  |  |  |  |
|  | 23 | Was an appropriate approach used to analyse the data? |  |  |  |  |  |  |  |  |  |
|  | 24 | Were at least two researchers involved in the analysis? |  |  |  |  |  |  |  |  |  |
|  | 25 | Were problems regarding the comprehensibility of the PROM instructions, items, response options, and recall period appropriately addressed by adapting the PROM? |  |  |  |  |  |  |  |  |  |
| Comprehensiveness | 26 | Were patients asked about the comprehensiveness of the PROM? |  |  |  |  |  |  |  |  |  |
|  | 27 | Was the final set of items tested? |  |  |  |  |  |  |  |  |  |
|  | 28 | Was an appropriate method used for assessing the comprehensiveness of the PROM? |  |  |  |  |  |  |  |  |  |
|  | 29 | Was each item tested in an appropriate number of patients? |  |  |  |  |  |  |  |  |  |
|  |  | For qualitative studies |  |  |  |  |  |  |  |  |  |
|  |  | For quantitative (survey) studies |  |  |  |  |  |  |  |  |  |
|  | 30 | Were skilled interviewers used? |  |  |  |  |  |  |  |  |  |
|  | 31 | Were the interviews based on an appropriate interview guide? |  |  |  |  |  |  |  |  |  |
|  | 32 | Were the interviews recorded and transcribed verbatim? |  |  |  |  |  |  |  |  |  |
|  | 33 | Was an appropriate approach used to analyse the data? |  |  |  |  |  |  |  |  |  |
|  | 34 | Were at least two researchers involved in the analysis? |  |  |  |  |  |  |  |  |  |
|  | 35 | Were problems regarding the comprehensiveness of the PROM appropriately addressed by adapting the PROM? |  |  |  |  |  |  |  |  |  |
| CONSENSUS | | |  |  |  |  |  |  |  |  |  |
| Box 2. Content validity | | | | | | | | | | | |
| 2a. Asking patients about relevance | | | | | | | | | | | |
| Design requirements | 1 | Was an appropriate method used to ask patients whether each item is relevant for their experience with the condition? | VG | VG | **VG** |  |  |  |  |  |  |
|  | 2 | Was each item tested in an appropriate number of patients? |  |  |  |  |  |  |  |  |  |
|  |  | For qualitative studies | VG | VG | **VG** |  |  |  |  |  |  |
|  |  | For quantitative (survey) studies |  |  |  |  |  |  |  |  |  |
|  | 3 | Were skilled group moderators/interviewers used? | VG | VG | **VG** |  |  |  |  |  |  |
|  | 4 | Were the group meetings or interviews based on an appropriate topic or interview guide? | A | D | **A** |  |  |  |  |  |  |
|  | 5 | Were the group meetings or interviews recorded and transcribed verbatim? | VG | D | **A** |  |  |  |  |  |  |
| Analyses | 6 | Was an appropriate approach used to analyse the data? | VG | VG | **VG** |  |  |  |  |  |  |
|  | 7 | Were at least two researchers involved in the analysis? | VG | VG | **VG** |  |  |  |  |  |  |
| 2b. Asking patients about comprehensiveness | | | | | | | | | | | |
| Design requirements | 8 | Was an appropriate method used for assessing the comprehensiveness of the PROM? | VG |  |  |  |  |  |  |  |  |
|  | 9 | Was each item tested in an appropriate number of patients? |  |  |  |  |  |  |  |  |  |
|  |  | For qualitative studies | VG |  |  |  |  |  |  |  |  |
|  |  | For quantitative (survey) studies |  |  |  |  |  |  |  |  |  |
|  | 10 | Were skilled group moderators/interviewers used? | VG |  |  |  |  |  |  |  |  |
|  | 11 | Were the group meetings or interviews based on an appropriate topic or interview guide? | A |  |  |  |  |  |  |  |  |
|  | 12 | Were the group meetings or interviews recorded and transcribed verbatim? | VG |  |  |  |  |  |  |  |  |
| Analyses | 13 | Was an appropriate approach used to analyse the data? | VG |  |  |  |  |  |  |  |  |
|  | 14 | Were at least two researchers involved in the analysis? | VG |  |  |  |  |  |  |  |  |
| 2c. Asking patients about comprehensibility | | | | | | | | | | | |
| Design requirements | 15 | Was an appropriate qualitative method used for assessing the comprehensibility of the PROM instructions, items, response options, and recall period? | VG | VG | **VG** |  |  |  |  |  |  |
|  | 16 | Was each item tested in an appropriate number of patients? |  |  |  |  |  |  |  |  |  |
|  |  | For qualitative studies | VG | VG | **VG** |  |  |  |  |  |  |
|  |  | For quantitative (survey) studies |  |  |  |  |  |  |  |  |  |
|  | 17 | Were skilled group moderators/interviewers used? | VG | VG | **VG** |  |  |  |  |  |  |
|  | 18 | Were the group meetings or interviews based on an appropriate topic or interview guide? | A | D | **A** |  |  |  |  |  |  |
|  | 19 | Were the group meetings or interviews recorded and transcribed verbatim? | VG | D | **A** |  |  |  |  |  |  |
| Analyses | 20 | Was an appropriate approach used to analyse the data? | VG | VG | **VG** |  |  |  |  |  |  |
|  | 21 | Were at least two researchers involved in the analysis? | VG | VG | **VG** |  |  |  |  |  |  |
| 2d. Asking professionals about relevance | | | | | | | | | | | |
| Design requirements | 22 | Was an appropriate method used to ask professionals whether each item is relevant for the construct of interest? |  |  |  |  |  |  |  |  |  |
|  | 23 | Were professionals from all relevant disciplines included? |  |  |  |  |  |  |  |  |  |
|  | 24 | Was each item tested in an appropriate number of professionals? |  |  |  |  |  |  |  |  |  |
|  |  | For qualitative studies |  |  |  |  |  |  |  |  |  |
|  |  | For quantitative (survey) studies |  |  |  |  |  |  |  |  |  |
| Analyses | 25 | Was an appropriate approach used to analyse the data? |  |  |  |  |  |  |  |  |  |
|  | 26 | Were at least two researchers involved in the analysis? |  |  |  |  |  |  |  |  |  |
|  | | | | | | | | | | | |
| Design requirements | 27 | Was an appropriate method used for assessing the comprehensiveness of the PROM? |  |  |  |  |  |  |  |  |  |
|  | 28 | Were professionals from all relevant disciplines included? |  |  |  |  |  |  |  |  |  |
|  | 29 | Was each item tested in an appropriate number of professionals? |  |  |  |  |  |  |  |  |  |
|  |  | For qualitative studies |  |  |  |  |  |  |  |  |  |
|  |  | For quantitative (survey) studies |  |  |  |  |  |  |  |  |  |
| Analyses | 30 | Was an appropriate approach used to analyse the data? |  |  |  |  |  |  |  |  |  |
|  | 31 | Were at least two researchers involved in the analysis? |  |  |  |  |  |  |  |  |  |
| CONSENSUS |  |  |  |  | **A** |  |  |  |  |  |  |
| Box 3. Structural validity | | | | | | | | | | | |
|  |  | Does the scale consist of effect indicators, i.e. is it based on a reflective model? |  |  |  |  |  |  |  |  |  |
|  |  | Does the study concern unidimensionality or structural validity? |  |  |  |  |  |  |  |  |  |
| Statistical methods | 1 | For CTT: Was exploratory or confirmatory factor analysis performed? |  |  |  |  |  |  |  |  |  |
|  | 2 | For IRT/Rasch: does the chosen model fit to the research question? |  |  |  |  |  |  |  |  |  |
|  | 3 | Was the sample size included in the analysis A? |  |  |  |  |  |  |  |  |  |
|  |  | For FA |  |  |  |  |  |  |  |  |  |
|  |  | For Rasch/1PL models |  |  |  |  |  |  |  |  |  |
|  |  | For 2PL parametric IRT models OR Mokken scale analysis |  |  |  |  |  |  |  |  |  |
| Other | 4 | Were there any other important flaws in the design or statistical methods of the study? |  |  |  |  |  |  |  |  |  |
| CONSENSUS | | |  |  |  |  |  |  |  |  |  |
| Box 4. Internal consistency | | | | | | | | | | | |
|  |  | Does the scale consist of effect indicators, i.e. is it based on a reflective model? |  |  |  |  |  |  |  |  |  |
| Design requirements | 1 | Was an internal consistency statistic calculated for each unidimensional scale or subscale separately? |  |  |  |  |  |  |  |  |  |
| Statistical methods | 2 | For continuous scores: Was Cronbach’s alpha or omega calculated? |  |  |  |  |  |  |  |  |  |
|  | 3 | For dichotomous scores: Was Cronbach’s alpha or KR-20 calculated? |  |  |  |  |  |  |  |  |  |
|  | 4 | For IRT-based scores: Was standard error of the theta (SE (θ)) or reliability coefficient of estimated latent trait value (index of (subject or item) separation) calculated? |  |  |  |  |  |  |  |  |  |
| Other | 5 | Were there any other important flaws in the design or statistical methods of the study? |  |  |  |  |  |  |  |  |  |
| CONSENSUS | | |  |  |  |  |  |  |  |  |  |
| Box 5. Cross-cultural validity/Measurement invariance | | | | | | | | | | | |
| Design requirements | 1 | Were the samples similar for relevant characteristics except for the group variable? |  |  |  |  |  |  |  |  |  |
| Statistical methods | 2 | Was an appropriate approach used to analyse the data? |  |  |  |  |  |  |  |  |  |
|  | 3 | Was the sample size included in the analysis A? |  |  |  |  |  |  |  |  |  |
|  |  | For Regression analyses or IRT/Rasch based analyses |  |  |  |  |  |  |  |  |  |
|  |  | For MGCFA* |  |  |  |  |  |  |  |  |  |
| Other | 4 | Were there any other important flaws in the design or statistical methods of the study? |  |  |  |  |  |  |  |  |  |
| CONSENSUS | | |  |  |  |  |  |  |  |  |  |
| Box 6. Reliability | | | | | | | | | | | |
| Design requirements | 1 | Were patients stable in the interim period on the construct to be measured? |  |  |  |  |  |  |  |  |  |
|  | 2 | Was the time interval appropriate? |  |  |  |  |  |  |  |  |  |
|  | 3 | Were the test conditions similar for the measurements? e.g. type of administration, environment, instructions |  |  |  |  |  |  |  |  |  |
| Statistical methods | 4 | For continuous scores: Was an intraclass correlation coefficient (ICC) calculated? |  |  |  |  |  |  |  |  |  |
|  | 5 | For dichotomous/nominal/ordinal scores: Was kappa calculated? |  |  |  |  |  |  |  |  |  |
|  | 6 | For ordinal scores: Was a weighted kappa calculated? |  |  |  |  |  |  |  |  |  |
|  | 7 | For ordinal scores: Was the weighting scheme described? e.g. linear, quadratic |  |  |  |  |  |  |  |  |  |
| Other | 8 | Were there any other important flaws in the design or statistical methods of the study? |  |  |  |  |  |  |  |  |  |
| CONSENSUS | | |  |  |  |  |  |  |  |  |  |
| Box 7. Measurement error | | | | | | | | | | | |
| Design requirements | 1 | Were patients stable in the interim period on the construct to be measured? |  |  |  |  |  |  |  |  |  |
|  | 2 | Was the time interval appropriate? |  |  |  |  |  |  |  |  |  |
|  | 3 | Were the test conditions similar for the measurements? e.g. type of administration, environment, instructions |  |  |  |  |  |  |  |  |  |
| Statistical methods | 4 | For continuous scores: Was the Standard Error of Measurement (SEM), Smallest Detectable Change (SDC) or Limits of Agreement (LoA) calculated? |  |  |  |  |  |  |  |  |  |
|  | 5 | For dichotomous/nominal/ordinal scores: Was the percentage (positive and negative) agreement calculated? |  |  |  |  |  |  |  |  |  |
| Other | 6 | Were there any other important flaws in the design or statistical methods of the study? |  |  |  |  |  |  |  |  |  |
| CONSENSUS | | |  |  |  |  |  |  |  |  |  |
| Box 8. Criterion validity | | | | | | | | | | | |
| Statistical methods | 1 | For continuous scores: Were correlations, or the area under the receiver operating curve calculated? |  |  |  |  |  |  |  |  |  |
|  | 2 | For dichotomous scores: Were sensitivity and specificity determined? |  |  |  |  |  |  |  |  |  |
| Other | 3 | Were there any other important flaws in the design or statistical methods of the study? |  |  |  |  |  |  |  |  |  |
| CONSENSUS | | |  |  |  |  |  |  |  |  |  |
| Box 9. Hypotheses testing for construct validity | | | | | | | | | | | |
| 9a. Comparison with other outcome measurement instruments (convergent validity) | | | | | | | | | | | |
| Design requirements | 1 | Is it clear what the comparator instrument(s) measure(s)? |  |  |  | VG | VG | **VG** | VG | VG | **VG** |
|  | 2 | Were the measurement properties of the comparator instrument(s) sufficient? |  |  |  | VG | VG | **VG** | A | VG | **A** |
| Statistical methods | 3 | Was the statistical method appropriate for the hypotheses to be tested? |  |  |  |  | VG | **VG** | A | VG | **VG** |
| Other | 4 | Were there any other important flaws in the design or statistical methods of the study? |  |  |  | VG | VG | **VG** | VG | VG | **VG** |
| CONSENSUS | | |  |  |  |  |  | **VG** |  |  | **A** |
| 9b. Comparison between subgroups (discriminative or known-groups validity) | | | | | | | | | | | |
| Design requirements | 5 | Was an A description provided of important characteristics of the subgroups? |  |  |  |  |  |  |  |  |  |
| Statistical methods | 6 | Was the statistical method appropriate for the hypotheses to be tested? |  |  |  |  |  |  |  |  |  |
| Other | 7 | Were there any other important flaws in the design or statistical methods of the study? |  |  |  |  |  |  |  |  |  |
| CONSENSUS | | |  |  |  |  |  |  |  |  |  |
| Box 10. Responsiveness | | | | | | | | | | | |
| 10a. Criterion approach (i.e. comparison to a gold standard) | | | | | | | | | | | |
| Statistical methods | 1 | For continuous scores: Were correlations between change scores, or the area under the Receiver Operator Curve (ROC) curve calculated? |  |  |  |  |  |  |  |  |  |
|  | 2 | For dichotomous scales: Were sensitivity and specificity (changed versus not changed) determined? |  |  |  |  |  |  |  |  |  |
| Other | 3 | Were there any other important flaws in the design or statistical methods of the study? |  |  |  |  |  |  |  |  |  |
| CONSENSUS | | |  |  |  |  |  |  |  |  |  |
| 10b. Construct approach (i.e. hypotheses testing; comparison with other outcome measurement instruments) | | | | | | | | | | | |
| Design requirements | 4 | Is it clear what the comparator instrument(s) measure(s)? |  |  |  |  |  |  |  | VG | VG |
|  | 5 | Were the measurement properties of the comparator instrument(s) sufficient? |  |  |  |  |  |  |  | VG | VG |
| Statistical methods | 6 | Was the statistical method appropriate for the hypotheses to be tested? |  |  |  |  |  |  |  | D | D |
| Other | 7 | Were there any other important flaws in the design or statistical methods of the study? |  |  |  |  |  |  |  | VG | VG |
| CONSENSUS | | |  |  |  |  |  |  |  |  | D |
| 10c. Construct approach (i.e. hypotheses testing: comparison between subgroups) | | | | | | | | | | | |
| Design requirements | 8 | Was an A description provided of important characteristics of the subgroups? |  |  |  |  | A | **A** |  |  |  |
| Statistical methods | 9 | Was the statistical method appropriate for the hypotheses to be tested? |  |  |  |  | VG | **VG** |  |  |  |
| Other | 10 | Were there any other important flaws in the design or statistical methods of the study? |  |  |  |  | VG | **VG** |  |  |  |
| CONSENSUS | | |  |  |  |  |  | **A** |  |  |  |
| 10d. Construct approach (i.e. hypotheses testing: before and after intervention) | | | | | | | | | | | |
| Design requirements | 11 | Was an A description provided of the intervention given? |  |  |  |  |  |  |  |  |  |
| Statistical methods | 12 | Was the statistical method appropriate for the hypotheses to be tested? |  |  |  |  |  |  |  |  |  |
| Other | 13 | Were there any other important flaws in the design or statistical methods of the study? |  |  |  |  |  |  |  |  |  |
| CONSENSUS | | |  |  |  |  |  |  |  |  |  |
| % | | | **47.62%** | | | **42.86%** | | | **25.00%** | | |

A: adequate; C: consensus; D: doubtful; R1: Reviewer 1; R2: Reviewer 2; VG: very good

| NMF2S | | | | | |
| --- | --- | --- | --- | --- | --- |
| COSMIN Risk of Bias Checklist | | | Faggianelli et al., 2024 | | |
|  | Item | Requirements | R1 | R2 | **C** |
| Box 3. Structural validity | | | | | |
|  |  | Does the scale consist of effect indicators, i.e. is it based on a reflective model? | Y | Y | **Y** |
|  |  | Does the study concern unidimensionality or structural validity? | SV | SV | **SV** |
| Statistical methods | 1 | For CTT: Was exploratory or confirmatory factor analysis performed? | VG | VG | **VG** |
|  | 2 | For IRT/Rasch: does the chosen model fit to the research question? | N/A | N/A | **N/A** |
|  | 3 | Was the sample size included in the analysis A? |  |  |  |
|  |  | For FA | VG | VG | **VG** |
|  |  | For Rasch/1PL models |  |  |  |
|  |  | For 2PL parametric IRT models OR Mokken scale analysis |  |  |  |
| Other | 4 | Were there any other important flaws in the design or statistical methods of the study? | VG | VG | **VG** |
| CONSENSUS | | |  |  | **VG** |
| 10d. Construct approach (i.e. hypotheses testing: before and after intervention) | | | | | |
| Design requirements | 11 | Was an A description provided of the intervention given? | VG | VG | **VG** |
| Statistical methods | 12 | Was the statistical method appropriate for the hypotheses to be tested? | VG | VG | **VG** |
| Other | 13 | Were there any other important flaws in the design or statistical methods of the study? | VG | VG | **VG** |
| CONSENSUS | | |  |  | **VG** |
| % | | | 100% | | |

A: adequate; C: consensus; N/A: not applicable; R1: Reviewer 1; R2: Reviewer 2; SV: structural validity; VG: very good

| POSQ | | | | | |
| --- | --- | --- | --- | --- | --- |
| COSMIN Risk of Bias Checklist | | | Matza et al., 2005 | | |
|  | Item | Requirements | R1 | R2 | **C** |
| Box 1. PROM Development | | | | | |
| 1a. PROM design | | | | | |
|  | 1 | Is a clear description provided of the construct to be measured? |  |  |  |
|  | 2 | Is the origin of the construct clear: was a theory, conceptual framework or disease model used or clear rationale provided to define the construct to be measured? |  |  |  |
|  | 3 | Is a clear description provided of the target population for which the PROM was developed? |  |  |  |
|  | 4 | Is a clear description provided of the context of use |  |  |  |
|  | 5 | Was the PROM development study performed in a sample representing the target population for which the PROM was developed? |  |  |  |
|  | 6 | Was an appropriate qualitative data collection method used to identify relevant items for a new PROM? |  |  |  |
|  | 7 | Were skilled group moderators/interviewers used? |  |  |  |
|  | 8 | Were the group meetings or interviews based on an appropriate topic or interview guide? |  |  |  |
|  | 9 | Were the group meetings or interviews recorded and transcribed verbatim? |  |  |  |
|  | 10 | Was an appropriate approach used to analyse the data? |  |  |  |
|  | 11 | Was at least part of the data coded independently? |  |  |  |
|  | 12 | Was data collection continued until saturation was reached? |  |  |  |
|  | 13 | For quantitative studies (surveys): was the sample size appropriate? |  |  |  |
| 1b. Cognitive interview study or pilot test | | | | | |
|  | 14 | Was a cognitive interview study or other pilot test conducted? |  |  |  |
| General design requirements | 15 | Was the cognitive interview study or other pilot test performed in a sample representing the target population? |  |  |  |
| Comprehensibility | 16 | Were patients asked about the comprehensibility of the PROM? |  |  |  |
|  | 17 | Were all items tested in their final form? |  |  |  |
|  | 18 | Was an appropriate qualitative method used to assess the comprehensibility of the PROM instructions, items, response options, and recall period? |  |  |  |
|  | 19 | Was each item tested in an appropriate number of patients? |  |  |  |
|  |  | For qualitative studies |  |  |  |
|  |  | For quantitative (survey) studies |  |  |  |
|  | 20 | Were skilled interviewers used? |  |  |  |
|  | 21 | Were the interviews based on an appropriate interview guide? |  |  |  |
|  | 22 | Were the interviews recorded and transcribed verbatim? |  |  |  |
|  | 23 | Was an appropriate approach used to analyse the data? |  |  |  |
|  | 24 | Were at least two researchers involved in the analysis? |  |  |  |
|  | 25 | Were problems regarding the comprehensibility of the PROM instructions, items, response options, and recall period appropriately addressed by adapting the PROM? |  |  |  |
| Comprehensiveness | 26 | Were patients asked about the comprehensiveness of the PROM? |  |  |  |
|  | 27 | Was the final set of items tested? |  |  |  |
|  | 28 | Was an appropriate method used for assessing the comprehensiveness of the PROM? |  |  |  |
|  | 29 | Was each item tested in an appropriate number of patients? |  |  |  |
|  |  | For qualitative studies |  |  |  |
|  |  | For quantitative (survey) studies |  |  |  |
|  | 30 | Were skilled interviewers used? |  |  |  |
|  | 31 | Were the interviews based on an appropriate interview guide? |  |  |  |
|  | 32 | Were the interviews recorded and transcribed verbatim? |  |  |  |
|  | 33 | Was an appropriate approach used to analyse the data? |  |  |  |
|  | 34 | Were at least two researchers involved in the analysis? |  |  |  |
|  | 35 | Were problems regarding the comprehensiveness of the PROM appropriately addressed by adapting the PROM? |  |  |  |
| CONSENSUS | | |  |  |  |
| Box 2. Content validity | | | | | |
| 2a. Asking patients about relevance | | | | | |
| Design requirements | 1 | Was an appropriate method used to ask patients whether each item is relevant for their experience with the condition? |  |  |  |
|  | 2 | Was each item tested in an appropriate number of patients? |  |  |  |
|  |  | For qualitative studies |  |  |  |
|  |  | For quantitative (survey) studies |  |  |  |
|  | 3 | Were skilled group moderators/interviewers used? |  |  |  |
|  | 4 | Were the group meetings or interviews based on an appropriate topic or interview guide? |  |  |  |
|  | 5 | Were the group meetings or interviews recorded and transcribed verbatim? |  |  |  |
| Analyses | 6 | Was an appropriate approach used to analyse the data? |  |  |  |
|  | 7 | Were at least two researchers involved in the analysis? |  |  |  |
| 2b. Asking patients about comprehensiveness | | | | | |
| Design requirements | 8 | Was an appropriate method used for assessing the comprehensiveness of the PROM? |  |  |  |
|  | 9 | Was each item tested in an appropriate number of patients? |  |  |  |
|  |  | For qualitative studies |  |  |  |
|  |  | For quantitative (survey) studies |  |  |  |
|  | 10 | Were skilled group moderators/interviewers used? |  |  |  |
|  | 11 | Were the group meetings or interviews based on an appropriate topic or interview guide? |  |  |  |
|  | 12 | Were the group meetings or interviews recorded and transcribed verbatim? |  |  |  |
| Analyses | 13 | Was an appropriate approach used to analyse the data? |  |  |  |
|  | 14 | Were at least two researchers involved in the analysis? |  |  |  |
| 2c. Asking patients about comprehensibility | | | | | |
| Design requirements | 15 | Was an appropriate qualitative method used for assessing the comprehensibility of the PROM instructions, items, response options, and recall period? |  |  |  |
|  | 16 | Was each item tested in an appropriate number of patients? |  |  |  |
|  |  | For qualitative studies |  |  |  |
|  |  | For quantitative (survey) studies |  |  |  |
|  | 17 | Were skilled group moderators/interviewers used? |  |  |  |
|  | 18 | Were the group meetings or interviews based on an appropriate topic or interview guide? |  |  |  |
|  | 19 | Were the group meetings or interviews recorded and transcribed verbatim? |  |  |  |
| Analyses | 20 | Was an appropriate approach used to analyse the data? |  |  |  |
|  | 21 | Were at least two researchers involved in the analysis? |  |  |  |
| 2d. Asking professionals about relevance | | | | | |
| Design requirements | 22 | Was an appropriate method used to ask professionals whether each item is relevant for the construct of interest? |  |  |  |
|  | 23 | Were professionals from all relevant disciplines included? |  |  |  |
|  | 24 | Was each item tested in an appropriate number of professionals? |  |  |  |
|  |  | For qualitative studies |  |  |  |
|  |  | For quantitative (survey) studies |  |  |  |
| Analyses | 25 | Was an appropriate approach used to analyse the data? |  |  |  |
|  | 26 | Were at least two researchers involved in the analysis? |  |  |  |
| 2e. Asking professionals about comprehensiveness | | | | | |
| Design requirements | 27 | Was an appropriate method used for assessing the comprehensiveness of the PROM? |  |  |  |
|  | 28 | Were professionals from all relevant disciplines included? |  |  |  |
|  | 29 | Was each item tested in an appropriate number of professionals? |  |  |  |
|  |  | For qualitative studies |  |  |  |
|  |  | For quantitative (survey) studies |  |  |  |
| Analyses | 30 | Was an appropriate approach used to analyse the data? |  |  |  |
|  | 31 | Were at least two researchers involved in the analysis? |  |  |  |
| CONSENSUS | | |  |  |  |
| Box 3. Structural validity | | | | | |
|  |  | Does the scale consist of effect indicators, i.e. is it based on a reflective model? |  |  |  |
|  |  | Does the study concern unidimensionality or structural validity? |  |  |  |
| Statistical methods | 1 | For CTT: Was exploratory or confirmatory factor analysis performed? |  |  |  |
|  | 2 | For IRT/Rasch: does the chosen model fit to the research question? |  |  |  |
|  | 3 | Was the sample size included in the analysis A? |  |  |  |
|  |  | For FA |  |  |  |
|  |  | For Rasch/1PL models |  |  |  |
|  |  | For 2PL parametric IRT models OR Mokken scale analysis |  |  |  |
| Other | 4 | Were there any other important flaws in the design or statistical methods of the study? |  |  |  |
| CONSENSUS | | |  |  |  |
| Box 4. Internal consistency | | | | | |
|  |  | Does the scale consist of effect indicators, i.e. is it based on a reflective model? | Y |  |  |
| Design requirements | 1 | Was an internal consistency statistic calculated for each unidimensional scale or subscale separately? | VG |  |  |
| Statistical methods | 2 | For continuous scores: Was Cronbach’s alpha or omega calculated? | VG |  |  |
|  | 3 | For dichotomous scores: Was Cronbach’s alpha or KR-20 calculated? |  |  |  |
|  | 4 | For IRT-based scores: Was standard error of the theta (SE (θ)) or reliability coefficient of estimated latent trait value (index of (subject or item) separation) calculated? |  |  |  |
| Other | 5 | Were there any other important flaws in the design or statistical methods of the study? | VG |  |  |
| CONSENSUS | | |  |  |  |
| Box 5. Cross-cultural validity/Measurement invariance | | | | | |
| Design requirements | 1 | Were the samples similar for relevant characteristics except for the group variable? |  |  |  |
| Statistical methods | 2 | Was an appropriate approach used to analyse the data? |  |  |  |
|  | 3 | Was the sample size included in the analysis A? |  |  |  |
|  |  | For Regression analyses or IRT/Rasch based analyses |  |  |  |
|  |  | For MGCFA* |  |  |  |
| Other | 4 | Were there any other important flaws in the design or statistical methods of the study? |  |  |  |
| CONSENSUS | | |  |  |  |
| Box 6. Reliability | | | | | |
| Design requirements | 1 | Were patients stable in the interim period on the construct to be measured? | VG | A | **VG** |
|  | 2 | Was the time interval appropriate? | VG | VG | **VG** |
|  | 3 | Were the test conditions similar for the measurements? e.g. type of administration, environment, instructions | A | A | **A** |
| Statistical methods | 4 | For continuous scores: Was an intraclass correlation coefficient (ICC) calculated? | VG | A | **A** |
|  | 5 | For dichotomous/nominal/ordinal scores: Was kappa calculated? |  |  |  |
|  | 6 | For ordinal scores: Was a weighted kappa calculated? |  |  |  |
|  | 7 | For ordinal scores: Was the weighting scheme described? e.g. linear, quadratic |  |  |  |
| Other | 8 | Were there any other important flaws in the design or statistical methods of the study? | VG | VG | **VG** |
| CONSENSUS | | |  |  | **A** |
| Box 7. Measurement error | | | | | |
| Design requirements | 1 | Were patients stable in the interim period on the construct to be measured? |  |  |  |
|  | 2 | Was the time interval appropriate? |  |  |  |
|  | 3 | Were the test conditions similar for the measurements? e.g. type of administration, environment, instructions |  |  |  |
| Statistical methods | 4 | For continuous scores: Was the Standard Error of Measurement (SEM), Smallest Detectable Change (SDC) or Limits of Agreement (LoA) calculated? |  |  |  |
|  | 5 | For dichotomous/nominal/ordinal scores: Was the percentage (positive and negative) agreement calculated? |  |  |  |
| Other | 6 | Were there any other important flaws in the design or statistical methods of the study? |  |  |  |
| CONSENSUS | | |  |  |  |
| Box 8. Criterion validity | | | | | |
| Statistical methods | 1 | For continuous scores: Were correlations, or the area under the receiver operating curve calculated? |  |  |  |
|  | 2 | For dichotomous scores: Were sensitivity and specificity determined? |  |  |  |
| Other | 3 | Were there any other important flaws in the design or statistical methods of the study? |  |  |  |
| CONSENSUS | | |  |  |  |
| Box 9. Hypotheses testing for construct validity | | | | | |
| 9a. Comparison with other outcome measurement instruments (convergent validity) | | | | | |
| Design requirements | 1 | Is it clear what the comparator instrument(s) measure(s)? |  |  |  |
|  | 2 | Were the measurement properties of the comparator instrument(s) sufficient? |  |  |  |
| Statistical methods | 3 | Was the statistical method appropriate for the hypotheses to be tested? |  |  |  |
| Other | 4 | Were there any other important flaws in the design or statistical methods of the study? |  |  |  |
| CONSENSUS | | |  |  |  |
| 9b. Comparison between subgroups (discriminative or known-groups validity) | | | | | |
| Design requirements | 5 | Was an A description provided of important characteristics of the subgroups? |  |  |  |
| Statistical methods | 6 | Was the statistical method appropriate for the hypotheses to be tested? |  |  |  |
| Other | 7 | Were there any other important flaws in the design or statistical methods of the study? |  |  |  |
| CONSENSUS | | |  |  |  |
| Box 10. Responsiveness | | | | | |
| 10a. Criterion approach (i.e. comparison to a gold standard) | | | | | |
| Statistical methods | 1 | For continuous scores: Were correlations between change scores, or the area under the Receiver Operator Curve (ROC) curve calculated? |  |  |  |
|  | 2 | For dichotomous scales: Were sensitivity and specificity (changed versus not changed) determined? |  |  |  |
| Other | 3 | Were there any other important flaws in the design or statistical methods of the study? |  |  |  |
| CONSENSUS | | |  |  |  |
| 10b. Construct approach (i.e. hypotheses testing; comparison with other outcome measurement instruments) | | | | | |
| Design requirements | 4 | Is it clear what the comparator instrument(s) measure(s)? |  |  |  |
|  | 5 | Were the measurement properties of the comparator instrument(s) sufficient? |  |  |  |
| Statistical methods | 6 | Was the statistical method appropriate for the hypotheses to be tested? |  |  |  |
| Other | 7 | Were there any other important flaws in the design or statistical methods of the study? |  |  |  |
| CONSENSUS | | |  |  |  |
| 10c. Construct approach (i.e. hypotheses testing: comparison between subgroups) | | | | | |
| Design requirements | 8 | Was an A description provided of important characteristics of the subgroups? |  |  |  |
| Statistical methods | 9 | Was the statistical method appropriate for the hypotheses to be tested? |  |  |  |
| Other | 10 | Were there any other important flaws in the design or statistical methods of the study? |  |  |  |
| CONSENSUS | | |  |  |  |
| 10d. Construct approach (i.e. hypotheses testing: before and after intervention) | | | | | |
| Design requirements | 11 | Was an A description provided of the intervention given? |  |  |  |
| Statistical methods | 12 | Was the statistical method appropriate for the hypotheses to be tested? |  |  |  |
| Other | 13 | Were there any other important flaws in the design or statistical methods of the study? |  |  |  |
| CONSENSUS | | |  |  |  |
| % | | | 33.33% | | |

A: adequate; C: consensus; R1: Reviewer 1; R2: Reviewer 2; VG: very good; Y: yes

| PSYCHLOPS | | | | | | | | | | | | | | | | | | | | | | | | | | | | | | | | |
| --- | --- | --- | --- | --- | --- | --- | --- | --- | --- | --- | --- | --- | --- | --- | --- | --- | --- | --- | --- | --- | --- | --- | --- | --- | --- | --- | --- | --- | --- | --- | --- | --- |
| COSMIN Risk of Bias Checklist | | | Ashworth et al., 2004 | | | Ashworth, Robinson, Godfrey, Shepherd, Evans, Seed, Parmentier & Tylee, 2005 | | | Ashworth, Robinson, Godfrey, Parmentier, Shepherd, Christey, Wright & Matthews, 2005 | | | Ashworth et al., 2009 | | | Evans et al., 2010 | | | Czachowski et al., 2011 | | | Héðinsson et al., 2013 | | | Alves et al., 2020 | | | Sales et al., 2021 | | | Austin et al., 2021 | | |
|  | Item | Requirement | R1 | R2 | **C** | R1 | R2 | **C** | R1 | R2 | **C** | R1 | R2 | **C** | R1 | R2 | **C** | R1 | R2 | **C** | R1 | R2 | **C** | R1 | R2 | **C** | R1 | R2 | **C** | R1 | R2 | **C** |
| Box 1. PROM Development | | | | | | | | | | | | | | | | | | | | | | | | | | | | | | | | |
| 1a. PROM design | | | | | | | | | | | | | | | | | | | | | | | | | | | | | | | | |
|  | 1 | Is a clear description provided of the construct to be measured? | VG | VG | **VG** |  |  |  |  |  |  |  |  |  |  |  |  |  |  |  |  |  |  |  |  |  |  |  |  |  |  |  |
|  | 2 | Is the origin of the construct clear: was a theory, conceptual framework or disease model used or clear rationale provided to define the construct to be measured? | VG | VG | **VG** |  |  |  |  |  |  |  |  |  |  |  |  |  |  |  |  |  |  |  |  |  |  |  |  |  |  |  |
|  | 3 | Is a clear description provided of the target population for which the PROM was developed? | VG | VG | **VG** |  |  |  |  |  |  |  |  |  |  |  |  |  |  |  |  |  |  |  |  |  |  |  |  |  |  |  |
|  | 4 | Is a clear description provided of the context of use | VG | VG | **VG** |  |  |  |  |  |  |  |  |  |  |  |  |  |  |  |  |  |  |  |  |  |  |  |  |  |  |  |
|  | 5 | Was the PROM development study performed in a sample representing the target population for which the PROM was developed? | VG | A | **VG** |  |  |  |  |  |  |  |  |  |  |  |  |  |  |  |  |  |  |  |  |  |  |  |  |  |  |  |
|  | 6 | Was an appropriate qualitative data collection method used to identify relevant items for a new PROM? | D | VG | **D** |  |  |  |  |  |  |  |  |  |  |  |  |  |  |  |  |  |  |  |  |  |  |  |  |  |  |  |
|  | 7 | Were skilled group moderators/interviewers used? | N/A | N/A | **N/A** |  |  |  |  |  |  |  |  |  |  |  |  |  |  |  |  |  |  |  |  |  |  |  |  |  |  |  |
|  | 8 | Were the group meetings or interviews based on an appropriate topic or interview guide? | N/A | N/A | **N/A** |  |  |  |  |  |  |  |  |  |  |  |  |  |  |  |  |  |  |  |  |  |  |  |  |  |  |  |
|  | 9 | Were the group meetings or interviews recorded and transcribed verbatim? | N/A | N/A | **N/A** |  |  |  |  |  |  |  |  |  |  |  |  |  |  |  |  |  |  |  |  |  |  |  |  |  |  |  |
|  | 10 | Was an appropriate approach used to analyse the data? | D | D | **D** |  |  |  |  |  |  |  |  |  |  |  |  |  |  |  |  |  |  |  |  |  |  |  |  |  |  |  |
|  | 11 | Was at least part of the data coded independently? | N/A | N/A | **N/A** |  |  |  |  |  |  |  |  |  |  |  |  |  |  |  |  |  |  |  |  |  |  |  |  |  |  |  |
|  | 12 | Was data collection continued until saturation was reached? | D | N/A | **N/A** |  |  |  |  |  |  |  |  |  |  |  |  |  |  |  |  |  |  |  |  |  |  |  |  |  |  |  |
|  | 13 | For quantitative studies (surveys): was the sample size appropriate? | D | N/A | **N/A** |  |  |  |  |  |  |  |  |  |  |  |  |  |  |  |  |  |  |  |  |  |  |  |  |  |  |  |
| 1b. Cognitive interview study or pilot test | | | | | | | | | | | | | | | | | | | | | | | | | | | | | | | | |
|  | 14 | Was a cognitive interview study or other pilot test conducted? | I | **VG** | **VG** |  |  |  |  |  |  |  |  |  |  |  |  |  |  |  |  |  |  |  |  |  |  |  |  |  |  |  |
| General design requirements | 15 | Was the cognitive interview study or other pilot test performed in a sample representing the target population? |  | **VG** | **VG** |  |  |  |  |  |  |  |  |  |  |  |  |  |  |  |  |  |  |  |  |  |  |  |  |  |  |  |
| Comprehensibility | 16 | Were patients asked about the comprehensibility of the PROM? |  | **I** | **I** |  |  |  |  |  |  |  |  |  |  |  |  |  |  |  |  |  |  |  |  |  |  |  |  |  |  |  |
|  | 17 | Were all items tested in their final form? |  |  |  |  |  |  |  |  |  |  |  |  |  |  |  |  |  |  |  |  |  |  |  |  |  |  |  |  |  |  |
|  | 18 | Was an appropriate qualitative method used to assess the comprehensibility of the PROM instructions, items, response options, and recall period? |  |  |  |  |  |  |  |  |  |  |  |  |  |  |  |  |  |  |  |  |  |  |  |  |  |  |  |  |  |  |
|  | 19 | Was each item tested in an appropriate number of patients? |  |  |  |  |  |  |  |  |  |  |  |  |  |  |  |  |  |  |  |  |  |  |  |  |  |  |  |  |  |  |
|  |  | For qualitative studies |  |  |  |  |  |  |  |  |  |  |  |  |  |  |  |  |  |  |  |  |  |  |  |  |  |  |  |  |  |  |
|  |  | For quantitative (survey) studies |  |  |  |  |  |  |  |  |  |  |  |  |  |  |  |  |  |  |  |  |  |  |  |  |  |  |  |  |  |  |
|  | 20 | Were skilled interviewers used? |  |  |  |  |  |  |  |  |  |  |  |  |  |  |  |  |  |  |  |  |  |  |  |  |  |  |  |  |  |  |
|  | 21 | Were the interviews based on an appropriate interview guide? |  |  |  |  |  |  |  |  |  |  |  |  |  |  |  |  |  |  |  |  |  |  |  |  |  |  |  |  |  |  |
|  | 22 | Were the interviews recorded and transcribed verbatim? |  |  |  |  |  |  |  |  |  |  |  |  |  |  |  |  |  |  |  |  |  |  |  |  |  |  |  |  |  |  |
|  | 23 | Was an appropriate approach used to analyse the data? |  |  |  |  |  |  |  |  |  |  |  |  |  |  |  |  |  |  |  |  |  |  |  |  |  |  |  |  |  |  |
|  | 24 | Were at least two researchers involved in the analysis? |  |  |  |  |  |  |  |  |  |  |  |  |  |  |  |  |  |  |  |  |  |  |  |  |  |  |  |  |  |  |
|  | 25 | Were problems regarding the comprehensibility of the PROM instructions, items, response options, and recall period appropriately addressed by adapting the PROM? |  |  |  |  |  |  |  |  |  |  |  |  |  |  |  |  |  |  |  |  |  |  |  |  |  |  |  |  |  |  |
| Comprehensiveness | 26 | Were patients asked about the comprehensiveness of the PROM? |  | **D** | **D** |  |  |  |  |  |  |  |  |  |  |  |  |  |  |  |  |  |  |  |  |  |  |  |  |  |  |  |
|  | 27 | Was the final set of items tested? |  |  |  |  |  |  |  |  |  |  |  |  |  |  |  |  |  |  |  |  |  |  |  |  |  |  |  |  |  |  |
|  | 28 | Was an appropriate method used for assessing the comprehensiveness of the PROM? |  |  |  |  |  |  |  |  |  |  |  |  |  |  |  |  |  |  |  |  |  |  |  |  |  |  |  |  |  |  |
|  | 29 | Was each item tested in an appropriate number of patients? |  |  |  |  |  |  |  |  |  |  |  |  |  |  |  |  |  |  |  |  |  |  |  |  |  |  |  |  |  |  |
|  |  | For qualitative studies |  |  |  |  |  |  |  |  |  |  |  |  |  |  |  |  |  |  |  |  |  |  |  |  |  |  |  |  |  |  |
|  |  | For quantitative (survey) studies |  |  |  |  |  |  |  |  |  |  |  |  |  |  |  |  |  |  |  |  |  |  |  |  |  |  |  |  |  |  |
|  | 30 | Were skilled interviewers used? |  |  |  |  |  |  |  |  |  |  |  |  |  |  |  |  |  |  |  |  |  |  |  |  |  |  |  |  |  |  |
|  | 31 | Were the interviews based on an appropriate interview guide? |  |  |  |  |  |  |  |  |  |  |  |  |  |  |  |  |  |  |  |  |  |  |  |  |  |  |  |  |  |  |
|  | 32 | Were the interviews recorded and transcribed verbatim? |  |  |  |  |  |  |  |  |  |  |  |  |  |  |  |  |  |  |  |  |  |  |  |  |  |  |  |  |  |  |
|  | 33 | Was an appropriate approach used to analyse the data? |  |  |  |  |  |  |  |  |  |  |  |  |  |  |  |  |  |  |  |  |  |  |  |  |  |  |  |  |  |  |
|  | 34 | Were at least two researchers involved in the analysis? |  |  |  |  |  |  |  |  |  |  |  |  |  |  |  |  |  |  |  |  |  |  |  |  |  |  |  |  |  |  |
|  | 35 | Were problems regarding the comprehensiveness of the PROM appropriately addressed by adapting the PROM? |  |  |  |  |  |  |  |  |  |  |  |  |  |  |  |  |  |  |  |  |  |  |  |  |  |  |  |  |  |  |
| CONSENSUS |  |  |  |  | **I** |  |  |  |  |  |  |  |  |  |  |  |  |  |  |  |  |  |  |  |  |  |  |  |  |  |  |  |
| Box 2. Content validity | | | | | | | | | | | | | | | | | | | | | | | | | | | | | | | | |
| 2a. Asking patients about relevance | | | | | | | | | | | | | | | | | | | | | | | | | | | | | | | | |
| Design requirements | 1 | Was an appropriate method used to ask patients whether each item is relevant for their experience with the condition? |  |  |  |  |  |  |  |  |  |  |  |  |  |  |  |  |  |  |  |  |  |  |  |  |  |  |  |  |  |  |
|  | 2 | Was each item tested in an appropriate number of patients? |  |  |  |  |  |  |  |  |  |  |  |  |  |  |  |  |  |  |  |  |  |  |  |  |  |  |  |  |  |  |
|  |  | For qualitative studies |  |  |  |  |  |  |  |  |  |  |  |  |  |  |  |  |  |  |  |  |  |  |  |  |  |  |  |  |  |  |
|  |  | For quantitative (survey) studies |  |  |  |  |  |  |  |  |  |  |  |  |  |  |  |  |  |  |  |  |  |  |  |  |  |  |  |  |  |  |
|  | 3 | Were skilled group moderators/interviewers used? |  |  |  |  |  |  |  |  |  |  |  |  |  |  |  |  |  |  |  |  |  |  |  |  |  |  |  |  |  |  |
|  | 4 | Were the group meetings or interviews based on an appropriate topic or interview guide? |  |  |  |  |  |  |  |  |  |  |  |  |  |  |  |  |  |  |  |  |  |  |  |  |  |  |  |  |  |  |
|  | 5 | Were the group meetings or interviews recorded and transcribed verbatim? |  |  |  |  |  |  |  |  |  |  |  |  |  |  |  |  |  |  |  |  |  |  |  |  |  |  |  |  |  |  |
| Analyses | 6 | Was an appropriate approach used to analyse the data? |  |  |  |  |  |  |  |  |  |  |  |  |  |  |  |  |  |  |  |  |  |  |  |  |  |  |  |  |  |  |
|  | 7 | Were at least two researchers involved in the analysis? |  |  |  |  |  |  |  |  |  |  |  |  |  |  |  |  |  |  |  |  |  |  |  |  |  |  |  |  |  |  |
| 2b. Asking patients about comprehensiveness | | | | | | | | | | | | | | | | | | | | | | | | | | | | | | | | |
| Design requirements | 8 | Was an appropriate method used for assessing the comprehensiveness of the PROM? |  |  |  |  |  |  |  |  |  |  |  |  |  |  |  |  |  |  |  |  |  |  |  |  |  |  |  |  |  |  |
|  | 9 | Was each item tested in an appropriate number of patients? |  |  |  |  |  |  |  |  |  |  |  |  |  |  |  |  |  |  |  |  |  |  |  |  |  |  |  |  |  |  |
|  |  | For qualitative studies |  |  |  |  |  |  |  |  |  |  |  |  |  |  |  |  |  |  |  |  |  |  |  |  |  |  |  |  |  |  |
|  |  | For quantitative (survey) studies |  |  |  |  |  |  |  |  |  |  |  |  |  |  |  |  |  |  |  |  |  |  |  |  |  |  |  |  |  |  |
|  | 10 | Were skilled group moderators/interviewers used? |  |  |  |  |  |  |  |  |  |  |  |  |  |  |  |  |  |  |  |  |  |  |  |  |  |  |  |  |  |  |
|  | 11 | Were the group meetings or interviews based on an appropriate topic or interview guide? |  |  |  |  |  |  |  |  |  |  |  |  |  |  |  |  |  |  |  |  |  |  |  |  |  |  |  |  |  |  |
|  | 12 | Were the group meetings or interviews recorded and transcribed verbatim? |  |  |  |  |  |  |  |  |  |  |  |  |  |  |  |  |  |  |  |  |  |  |  |  |  |  |  |  |  |  |
| Analyses | 13 | Was an appropriate approach used to analyse the data? |  |  |  |  |  |  |  |  |  |  |  |  |  |  |  |  |  |  |  |  |  |  |  |  |  |  |  |  |  |  |
|  | 14 | Were at least two researchers involved in the analysis? |  |  |  |  |  |  |  |  |  |  |  |  |  |  |  |  |  |  |  |  |  |  |  |  |  |  |  |  |  |  |
| 2c. Asking patients about comprehensibility | | | | | | | | | | | | | | | | | | | | | | | | | | | | | | | | |
| Design requirements | 15 | Was an appropriate qualitative method used for assessing the comprehensibility of the PROM instructions, items, response options, and recall period? |  |  |  |  |  |  |  |  |  |  |  |  |  |  |  |  |  |  |  |  |  |  |  |  |  |  |  |  |  |  |
|  | 16 | Was each item tested in an appropriate number of patients? |  |  |  |  |  |  |  |  |  |  |  |  |  |  |  |  |  |  |  |  |  |  |  |  |  |  |  |  |  |  |
|  |  | For qualitative studies |  |  |  |  |  |  |  |  |  |  |  |  |  |  |  |  |  |  |  |  |  |  |  |  |  |  |  |  |  |  |
|  |  | For quantitative (survey) studies |  |  |  |  |  |  |  |  |  |  |  |  |  |  |  |  |  |  |  |  |  |  |  |  |  |  |  |  |  |  |
|  | 17 | Were skilled group moderators/interviewers used? |  |  |  |  |  |  |  |  |  |  |  |  |  |  |  |  |  |  |  |  |  |  |  |  |  |  |  |  |  |  |
|  | 18 | Were the group meetings or interviews based on an appropriate topic or interview guide? |  |  |  |  |  |  |  |  |  |  |  |  |  |  |  |  |  |  |  |  |  |  |  |  |  |  |  |  |  |  |
|  | 19 | Were the group meetings or interviews recorded and transcribed verbatim? |  |  |  |  |  |  |  |  |  |  |  |  |  |  |  |  |  |  |  |  |  |  |  |  |  |  |  |  |  |  |
| Analyses | 20 | Was an appropriate approach used to analyse the data? |  |  |  |  |  |  |  |  |  |  |  |  |  |  |  |  |  |  |  |  |  |  |  |  |  |  |  |  |  |  |
|  | 21 | Were at least two researchers involved in the analysis? |  |  |  |  |  |  |  |  |  |  |  |  |  |  |  |  |  |  |  |  |  |  |  |  |  |  |  |  |  |  |
| 2d. Asking professionals about relevance | | | | | | | | | | | | | | | | | | | | | | | | | | | | | | | | |
| Design requirements | 22 | Was an appropriate method used to ask professionals whether each item is relevant for the construct of interest? |  |  |  |  |  |  | A | VG | **VG** |  |  |  |  |  |  |  |  |  |  |  |  |  |  |  |  |  |  |  |  |  |
|  | 23 | Were professionals from all relevant disciplines included? |  |  |  |  |  |  | A | D | **D** |  |  |  |  |  |  |  |  |  |  |  |  |  |  |  |  |  |  |  |  |  |
|  | 24 | Was each item tested in an appropriate number of professionals? |  |  |  |  |  |  |  |  |  |  |  |  |  |  |  |  |  |  |  |  |  |  |  |  |  |  |  |  |  |  |
|  |  | For qualitative studies |  |  |  |  |  |  |  | A | **A** |  |  |  |  |  |  |  |  |  |  |  |  |  |  |  |  |  |  |  |  |  |
|  |  | For quantitative (survey) studies |  |  |  |  |  |  | D |  |  |  |  |  |  |  |  |  |  |  |  |  |  |  |  |  |  |  |  |  |  |  |
| Analyses | 25 | Was an appropriate approach used to analyse the data? |  |  |  |  |  |  | A | VG | **VG** |  |  |  |  |  |  |  |  |  |  |  |  |  |  |  |  |  |  |  |  |  |
|  | 26 | Were at least two researchers involved in the analysis? |  |  |  |  |  |  | A | D | **D** |  |  |  |  |  |  |  |  |  |  |  |  |  |  |  |  |  |  |  |  |  |
| 2e. Asking professionals about comprehensiveness | | | | | | | | | | | | | | | | | | | | | | | | | | | | | | | | |
| Design requirements | 27 | Was an appropriate method used for assessing the comprehensiveness of the PROM? |  |  |  |  |  |  |  | VG |  |  |  |  |  |  |  |  |  |  |  |  |  |  |  |  |  |  |  |  |  |  |
|  | 28 | Were professionals from all relevant disciplines included? |  |  |  |  |  |  |  | D |  |  |  |  |  |  |  |  |  |  |  |  |  |  |  |  |  |  |  |  |  |  |
|  | 29 | Was each item tested in an appropriate number of professionals? |  |  |  |  |  |  |  |  |  |  |  |  |  |  |  |  |  |  |  |  |  |  |  |  |  |  |  |  |  |  |
|  |  | For qualitative studies |  |  |  |  |  |  |  | A |  |  |  |  |  |  |  |  |  |  |  |  |  |  |  |  |  |  |  |  |  |  |
|  |  | For quantitative (survey) studies |  |  |  |  |  |  |  |  |  |  |  |  |  |  |  |  |  |  |  |  |  |  |  |  |  |  |  |  |  |  |
| Analyses | 30 | Was an appropriate approach used to analyse the data? |  |  |  |  |  |  |  | VG |  |  |  |  |  |  |  |  |  |  |  |  |  |  |  |  |  |  |  |  |  |  |
|  | 31 | Were at least two researchers involved in the analysis? |  |  |  |  |  |  |  | D |  |  |  |  |  |  |  |  |  |  |  |  |  |  |  |  |  |  |  |  |  |  |
| CONSENSUSUS | | |  |  |  |  |  |  |  |  | **D** |  |  |  |  |  |  |  |  |  |  |  |  |  |  |  |  |  |  |  |  |  |
| Box 3. Structural validity | | | | | | | | | | | | | | | | | | | | | | | | | | | | | | | | |
|  |  | Does the scale consist of effect indicators, i.e. is it based on a reflective model? |  |  |  |  |  |  |  |  |  |  |  |  |  |  |  |  |  |  |  |  |  |  |  |  | Y | Y | **Y** |  |  |  |
|  |  | Does the study concern unidimensionality or structural validity? |  |  |  |  |  |  |  |  |  |  |  |  |  |  |  |  |  |  |  |  |  |  |  |  | U | U | **U** |  |  |  |
| Statistical methods | 1 | For CTT: Was exploratory or confirmatory factor analysis performed? |  |  |  |  |  |  |  |  |  |  |  |  |  |  |  |  |  |  |  |  |  |  |  |  | A | VG | **VG** |  |  |  |
|  | 2 | For IRT/Rasch: does the chosen model fit to the research question? |  |  |  |  |  |  |  |  |  |  |  |  |  |  |  |  |  |  |  |  |  |  |  |  | N/A |  | **N/A** |  |  |  |
|  | 3 | Was the sample size included in the analysis A? |  |  |  |  |  |  |  |  |  |  |  |  |  |  |  |  |  |  |  |  |  |  |  |  |  |  |  |  |  |  |
|  |  | For FA |  |  |  |  |  |  |  |  |  |  |  |  |  |  |  |  |  |  |  |  |  |  |  |  | VG | VG | **VG** |  |  |  |
|  |  | For Rasch/1PL models |  |  |  |  |  |  |  |  |  |  |  |  |  |  |  |  |  |  |  |  |  |  |  |  |  |  |  |  |  |  |
|  |  | For 2PL parametric IRT models OR Mokken scale analysis |  |  |  |  |  |  |  |  |  |  |  |  |  |  |  |  |  |  |  |  |  |  |  |  |  |  |  |  |  |  |
| Other | 4 | Were there any other important flaws in the design or statistical methods of the study? |  |  |  |  |  |  |  |  |  |  |  |  |  |  |  |  |  |  |  |  |  |  |  |  | VG | VG | **VG** |  |  |  |
| CONSENSUS |  |  |  |  |  |  |  |  |  |  |  |  |  |  |  |  |  |  |  |  |  |  |  |  |  |  |  |  | **VG** |  |  |  |
| Box 4. Internal consistency | | | | | | | | | | | | | | | | | | | | | | | | | | | | | | | | |
|  |  | Does the scale consist of effect indicators, i.e. is it based on a reflective model? |  |  |  | Y | Y | **Y** |  |  |  | Y | Y | **Y** | Y | Y | **Y** | Y | Y | **Y** | Y | Y | **Y** | Y | Y | **Y** | Y | Y | **Y** | Y | Y | **Y** |
| Design requirements | 1 | Was an internal consistency statistic calculated for each unidimensional scale or subscale separately? |  |  |  | VG | VG | **VG** |  |  |  | VG | VG | **VG** | VG | VG | **VG** | D | VG | **VG** | VG | VG | **VG** | VG | VG | **VG** | VG | VG | **VG** | VG | VG | **VG** |
| Statistical methods | 2 | For continuous scores: Was Cronbach’s alpha or omega calculated? |  |  |  | VG | VG | **VG** |  |  |  | VG | VG | **VG** | VG | VG | **VG** | VG | VG | **VG** | VG | VG | **VG** | VG | VG | **VG** | VG | VG | **VG** | VG | VG | **VG** |
|  | 3 | For dichotomous scores: Was Cronbach’s alpha or KR-20 calculated? |  |  |  | N/A |  | **N/A** |  |  |  | N/A |  | **N/A** |  |  | **N/A** |  |  | **N/A** | N/A |  | **N/A** | N/A |  | **N/A** | N/A |  | **N/A** | N/A |  | **N/A** |
|  | 4 | For IRT-based scores: Was standard error of the theta (SE (θ)) or reliability coefficient of estimated latent trait value (index of (subject or item) separation) calculated? |  |  |  | N/A |  | **N/A** |  |  |  | N/A |  | **N/A** |  |  | **N/A** |  |  | **N/A** | N/A |  | **N/A** | N/A |  | **N/A** | N/A |  | **VG** | N/A |  | **N/A** |
| Other | 5 | Were there any other important flaws in the design or statistical methods of the study? |  |  |  | VG | VG | **VG** |  |  |  | VG | VG | **VG** |  | VG | **VG** | VG | VG | **VG** | I | VG | **VG** | VG | VG | **VG** | VG | VG | **VG** | VG | VG | **VG** |
| CONSENSUS | | |  |  |  |  |  | **VG** |  |  |  |  |  | **VG** |  |  | **VG** |  |  | **VG** |  |  | **VG** |  |  | **VG** |  |  | **VG** |  |  | **VG** |
| Box 5. Cross-cultural validity/Measurement invariance | | | | | | | | | | | | | | | | | | | | | | | | | | | | | | | |  |
| Design requirements | 1 | Were the samples similar for relevant characteristics except for the group variable? |  |  |  |  |  |  |  |  |  |  |  |  |  |  |  |  |  |  |  |  |  |  |  |  |  |  |  |  |  |  |
| Statistical methods | 2 | Was an appropriate approach used to analyse the data? |  |  |  |  |  |  |  |  |  |  |  |  |  |  |  |  |  |  |  |  |  |  |  |  |  |  |  |  |  |  |
|  | 3 | Was the sample size included in the analysis A? |  |  |  |  |  |  |  |  |  |  |  |  |  |  |  |  |  |  |  |  |  |  |  |  |  |  |  |  |  |  |
|  |  | For Regression analyses or IRT/Rasch based analyses |  |  |  |  |  |  |  |  |  |  |  |  |  |  |  |  |  |  |  |  |  |  |  |  |  |  |  |  |  |  |
|  |  | For MGCFA* |  |  |  |  |  |  |  |  |  |  |  |  |  |  |  |  |  |  |  |  |  |  |  |  |  |  |  |  |  |  |
| Other | 4 | Were there any other important flaws in the design or statistical methods of the study? |  |  |  |  |  |  |  |  |  |  |  |  |  |  |  |  |  |  |  |  |  |  |  |  |  |  |  |  |  |  |
| CONSENSUS |  |  |  |  |  |  |  |  |  |  |  |  |  |  |  |  |  |  |  |  |  |  |  |  |  |  |  |  |  |  |  |  |
| Box 6. Reliability |  |  |  |  |  |  |  |  |  |  |  |  |  |  |  |  |  |  |  |  |  |  |  |  |  |  |  |  |  |  |  |  |
| Design requirements | 1 | Were patients stable in the interim period on the construct to be measured? |  |  |  |  |  |  |  |  |  |  |  |  | A | A | **A** |  |  |  |  |  |  |  |  |  |  |  |  |  |  |  |
|  | 2 | Was the time interval appropriate? |  |  |  |  |  |  |  |  |  |  |  |  | VG | VG | **VG** |  |  |  |  |  |  |  |  |  |  |  |  |  |  |  |
|  | 3 | Were the test conditions similar for the measurements? e.g. type of administration, environment, instructions |  |  |  |  |  |  |  |  |  |  |  |  | A | A | **A** |  |  |  |  |  |  |  |  |  |  |  |  |  |  |  |
| Statistical methods | 4 | For continuous scores: Was an intraclass correlation coefficient (ICC) calculated? |  |  |  |  |  |  |  |  |  |  |  |  | VG | A | **A** |  |  |  |  |  |  |  |  |  |  |  |  |  |  |  |
|  | 5 | For dichotomous/nominal/ordinal scores: Was kappa calculated? |  |  |  |  |  |  |  |  |  |  |  |  | N/A |  | **N/A** |  |  |  |  |  |  |  |  |  |  |  |  |  |  |  |
|  | 6 | For ordinal scores: Was a weighted kappa calculated? |  |  |  |  |  |  |  |  |  |  |  |  | N/A |  | **N/A** |  |  |  |  |  |  |  |  |  |  |  |  |  |  |  |
|  | 7 | For ordinal scores: Was the weighting scheme described? e.g. linear, quadratic |  |  |  |  |  |  |  |  |  |  |  |  | N/A |  | **N/A** |  |  |  |  |  |  |  |  |  |  |  |  |  |  |  |
| Other | 8 | Were there any other important flaws in the design or statistical methods of the study? |  |  |  |  |  |  |  |  |  |  |  |  | VG | VG | **VG** |  |  |  |  |  |  |  |  |  |  |  |  |  |  |  |
| CONSENSUS |  |  |  |  |  |  |  |  |  |  |  |  |  |  |  |  | **A** |  |  |  |  |  |  |  |  |  |  |  |  |  |  |  |
| Box 7. Measurement error | | | | | | | | | | | | | | | | | | | | | | | | | | | | | | | | |
| Design requirements | 1 | Were patients stable in the interim period on the construct to be measured? |  |  |  |  |  |  |  |  |  |  |  |  |  |  |  |  |  |  |  |  |  |  |  |  |  |  |  |  |  |  |
|  | 2 | Was the time interval appropriate? |  |  |  |  |  |  |  |  |  |  |  |  |  |  |  |  |  |  |  |  |  |  |  |  |  |  |  |  |  |  |
|  | 3 | Were the test conditions similar for the measurements? e.g. type of administration, environment, instructions |  |  |  |  |  |  |  |  |  |  |  |  |  |  |  |  |  |  |  |  |  |  |  |  |  |  |  |  |  |  |
| Statistical methods | 4 | For continuous scores: Was the Standard Error of Measurement (SEM), Smallest Detectable Change (SDC) or Limits of Agreement (LoA) calculated? |  |  |  |  |  |  |  |  |  |  |  |  |  |  |  |  |  |  |  |  |  |  |  |  |  |  |  |  |  |  |
|  | 5 | For dichotomous/nominal/ordinal scores: Was the percentage (positive and negative) agreement calculated? |  |  |  |  |  |  |  |  |  |  |  |  |  |  |  |  |  |  |  |  |  |  |  |  |  |  |  |  |  |  |
| Other | 6 | Were there any other important flaws in the design or statistical methods of the study? |  |  |  |  |  |  |  |  |  |  |  |  |  |  |  |  |  |  |  |  |  |  |  |  |  |  |  |  |  |  |
| CONSENSUS |  |  |  |  |  |  |  |  |  |  |  |  |  |  |  |  |  |  |  |  |  |  |  |  |  |  |  |  |  |  |  |  |
| Box 8. Criterion validity | | | | | | | | | | | | | | | | | | | | | | | | | | | | | | | | |
| Statistical methods | 1 | For continuous scores: Were correlations, or the area under the receiver operating curve calculated? |  |  |  |  |  |  |  |  |  |  |  |  |  |  |  |  |  |  |  |  |  |  |  |  |  |  |  |  |  |  |
|  | 2 | For dichotomous scores: Were sensitivity and specificity determined? |  |  |  |  |  |  |  |  |  |  |  |  |  |  |  |  |  |  |  |  |  |  |  |  |  |  |  |  |  |  |
| Other | 3 | Were there any other important flaws in the design or statistical methods of the study? |  |  |  |  |  |  |  |  |  |  |  |  |  |  |  |  |  |  |  |  |  |  |  |  |  |  |  |  |  |  |
| CONSENS |  |  |  |  |  |  |  |  |  |  |  |  |  |  |  |  |  |  |  |  |  |  |  |  |  |  |  |  |  |  |  |  |
| Box 9. Hypotheses testing for construct validity | | | | | | | | | | | | | | | | | | | | | | | | | | | | | | | | |
| 9a. Comparison with other outcome measurement instruments (convergent validity) | | | | | | | | | | | | | | | | | | | | | | | | | | | | | | | | |
| Design requirements | 1 | Is it clear what the comparator instrument(s) measure(s)? |  |  |  | VG |  | **VG** |  |  |  | VG |  | **VG** |  |  |  | VG | VG | **VG** | VG | VG | **VG** | VG | VG | **VG** | VG | VG | **VG** | VG |  |  |
|  | 2 | Were the measurement properties of the comparator instrument(s) sufficient? |  |  |  | VG |  | **VG** |  |  |  | VG |  | **VG** |  |  |  | I | I | **I** | VG | VG | **VG** | VG | VG | **VG** | VG | VG | **VG** | VG |  |  |
| Statistical methods | 3 | Was the statistical method appropriate for the hypotheses to be tested? |  |  |  | VG |  | **VG** |  |  |  | VG |  | **VG** |  |  |  | VG | A | **VG** | VG | VG | **VG** | VG | A | **A** | VG | A | **A** | VG |  |  |
| Other | 4 | Were there any other important flaws in the design or statistical methods of the study? |  |  |  | VG |  | **VG** |  |  |  | VG |  | **VG** |  |  |  | VG | VG | **VG** | VG | VG | **VG** | VG | VG | **VG** | VG | VG | **VG** | VG |  |  |
| CONSENSUS |  |  |  |  |  |  |  | **VG** |  |  |  |  |  | **VG** |  |  |  |  |  | **I** |  |  | **VG** |  |  | **A** |  |  | **A** |  |  |  |
| 9b. Comparison between subgroups (discriminative or known-groups validity) | | | | | | | | | | | | | | | | | | | | | | | | | | | | | | | | |
| Design requirements | 5 | Was an A description provided of important characteristics of the subgroups? |  |  |  |  |  |  |  |  |  |  |  |  |  |  |  |  |  |  |  |  |  | VG |  |  |  |  |  |  |  |  |
| Statistical methods | 6 | Was the statistical method appropriate for the hypotheses to be tested? |  |  |  |  |  |  |  |  |  |  |  |  |  |  |  |  |  |  |  |  |  | VG |  |  |  |  |  |  |  |  |
| Other | 7 | Were there any other important flaws in the design or statistical methods of the study? |  |  |  |  |  |  |  |  |  |  |  |  |  |  |  |  |  |  |  |  |  | VG |  |  |  |  |  |  |  |  |
| CONSENSUS | | |  |  |  |  |  |  |  |  |  |  |  |  |  |  |  |  |  |  |  |  |  |  |  |  |  |  |  |  |  |  |
| Box 10. Responsiveness | | | | | | | | | | | | | | | | | | | | | | | | | | | | | | | | |
| 10a. Criterion approach (i.e. comparison to a gold standard) | | | | | | | | | | | | | | | | | | | | | | | | | | | | | | | | |
| Statistical methods | 1 | For continuous scores: Were correlations between change scores, or the area under the Receiver Operator Curve (ROC) curve calculated? |  |  |  |  |  |  |  |  |  |  |  |  |  |  |  |  |  |  |  |  |  |  |  |  |  |  |  |  |  |  |
|  | 2 | For dichotomous scales: Were sensitivity and specificity (changed versus not changed) determined? |  |  |  |  |  |  |  |  |  |  |  |  |  |  |  |  |  |  |  |  |  |  |  |  |  |  |  |  |  |  |
| Other | 3 | Were there any other important flaws in the design or statistical methods of the study? |  |  |  |  |  |  |  |  |  |  |  |  |  |  |  |  |  |  |  |  |  |  |  |  |  |  |  |  |  |  |
| CONSENSUS | | |  |  |  |  |  |  |  |  |  |  |  |  |  |  |  |  |  |  |  |  |  |  |  |  |  |  |  |  |  |  |
| 10b. Construct approach (i.e. hypotheses testing; comparison with other outcome measurement instruments) | | | | | | | | | | | | | | | | | | | | | | | | | | | | | | | | |
| Design requirements | 4 | Is it clear what the comparator instrument(s) measure(s)? |  |  |  | VG | VG | **VG** |  |  |  | VG | VG | **VG** |  |  |  |  |  |  | VG | VG | **VG** |  |  |  | VG |  | **VG** | VG | VG | **VG** |
|  | 5 | Were the measurement properties of the comparator instrument(s) sufficient? |  |  |  | VG | VG | **VG** |  |  |  | VG | VG | **VG** |  |  |  |  |  |  | VG | VG | **VG** |  |  |  | VG |  | **VG** | VG | A | **A** |
| Statistical methods | 6 | Was the statistical method appropriate for the hypotheses to be tested? |  |  |  | VG | VG | **VG** |  |  |  | VG | A | **VG** |  |  |  |  |  |  | VG | VG | **VG** |  |  |  | VG |  | **A** | VG | A | **A** |
| Other | 7 | Were there any other important flaws in the design or statistical methods of the study? |  |  |  | VG |  | **VG** |  |  |  | VG | VG | **VG** |  |  |  |  |  |  | VG | VG | **VG** |  |  |  | D |  | **VG** | VG | VG | **VG** |
| CONSENSUS | | |  |  |  |  |  | **VG** |  |  |  |  |  | **VG** |  |  |  |  |  |  |  |  | **VG** |  |  |  |  |  | **A** |  |  | **A** |
| 10c. Construct approach (i.e. hypotheses testing: comparison between subgroups) | | | | | | | | | | | | | | | | | | | | | | | | | | | | | | | | |
| Design requirements | 8 | Was an A description provided of important characteristics of the subgroups? |  |  |  | VG |  |  |  |  |  |  |  |  |  |  |  | VG |  |  | A |  |  |  |  |  |  |  |  |  |  |  |
| Statistical methods | 9 | Was the statistical method appropriate for the hypotheses to be tested? |  |  |  | A |  |  |  |  |  |  |  |  |  |  |  | VG |  |  | VG |  |  |  |  |  |  |  |  |  |  |  |
| Other | 10 | Were there any other important flaws in the design or statistical methods of the study? |  |  |  | VG |  |  |  |  |  |  |  |  |  |  |  | VG |  |  | VG |  |  |  |  |  |  |  |  |  |  |  |
| CONSENSUS | | |  |  |  |  |  |  |  |  |  |  |  |  |  |  |  |  |  |  |  |  |  |  |  |  |  |  |  |  |  |  |
| 10d. Construct approach (i.e. hypotheses testing: before and after intervention) |  |  |  |  |  |  |  |  |  |  |  |  |  |  |  |  |  |  |  |  |  |  |  |  |  |  |  |  |  |  |  |  |
| Design requirements | 11 | Was an A description provided of the intervention given? |  |  |  |  |  | **VG** |  |  |  |  | VG | **VG** |  |  |  |  | VG | **VG** |  |  |  |  |  |  | VG | D | **D** |  | D | **VG** |
| Statistical methods | 12 | Was the statistical method appropriate for the hypotheses to be tested? |  |  |  |  |  | **VG** |  |  |  |  | VG | **VG** |  |  |  |  | VG | **VG** |  |  |  |  |  |  | VG | VG | **VG** |  | A | **A** |
| Other | 13 | Were there any other important flaws in the design or statistical methods of the study? |  |  |  |  |  | **VG** |  |  |  |  | VG | **VG** |  |  |  |  | VG | **VG** |  |  |  |  |  |  | VG | VG | **VG** |  | VG | **VG** |
| CONSENSUS | | |  |  |  |  |  | **VG** |  |  |  |  |  | **VG** |  |  |  |  |  | **VG** |  |  |  |  |  |  |  |  | **D** |  |  | **A** |
| % | | | **52.94%** | | | **41.18%** | | | **0.00%** | | | **41.18%** | | | **58.33%** | | | **42.86%** | | | **64.71%** | | | **53.85%** | | | **56.52%** | | | **35.29%** | | |

A: adequate; C: consensus; D: doubtful; I: inadequate; N/A: not applicable; R1: Reviewer 1; R2: Reviewer 2; U: unidimensionality; VG: very good; Y: yes

| Questionnaire for female lower urinary tract symptoms and their impairment of quality of life | | | | | |
| --- | --- | --- | --- | --- | --- |
| COSMIN Risk of Bias Checklist | | | Bernstein et al., 1996 | | |
|  | Item | Requirements | R1 | R2 | **C** |
| Box 1. PROM Development | | | | | |
| 1a. PROM design | | | | | |
|  | 1 | Is a clear description provided of the construct to be measured? | VG | VG | **VG** |
|  | 2 | Is the origin of the construct clear: was a theory, conceptual framework or disease model used or clear rationale provided to define the construct to be measured? | VG | VG | **VG** |
|  | 3 | Is a clear description provided of the target population for which the PROM was developed? | VG | VG | **VG** |
|  | 4 | Is a clear description provided of the context of use | VG | VG | **VG** |
|  | 5 | Was the PROM development study performed in a sample representing the target population for which the PROM was developed? | I | I | **I** |
|  | 6 | Was an appropriate qualitative data collection method used to identify relevant items for a new PROM? |  |  |  |
|  | 7 | Were skilled group moderators/interviewers used? |  |  |  |
|  | 8 | Were the group meetings or interviews based on an appropriate topic or interview guide? |  |  |  |
|  | 9 | Were the group meetings or interviews recorded and transcribed verbatim? |  |  |  |
|  | 10 | Was an appropriate approach used to analyse the data? |  |  |  |
|  | 11 | Was at least part of the data coded independently? |  |  |  |
|  | 12 | Was data collection continued until saturation was reached? |  |  |  |
|  | 13 | For quantitative studies (surveys): was the sample size appropriate? | N/A |  | **N/A** |
| 1b. Cognitive interview study or pilot test | | | | | |
|  | 14 | Was a cognitive interview study or other pilot test conducted? | I | I | **I** |
| General design requirements | 15 | Was the cognitive interview study or other pilot test performed in a sample representing the target population? |  |  |  |
| Comprehensibility | 16 | Were patients asked about the comprehensibility of the PROM? |  |  |  |
|  | 17 | Were all items tested in their final form? |  |  |  |
|  | 18 | Was an appropriate qualitative method used to assess the comprehensibility of the PROM instructions, items, response options, and recall period? |  |  |  |
|  | 19 | Was each item tested in an appropriate number of patients? |  |  |  |
|  |  | For qualitative studies |  |  |  |
|  |  | For quantitative (survey) studies |  |  |  |
|  | 20 | Were skilled interviewers used? |  |  |  |
|  | 21 | Were the interviews based on an appropriate interview guide? |  |  |  |
|  | 22 | Were the interviews recorded and transcribed verbatim? |  |  |  |
|  | 23 | Was an appropriate approach used to analyse the data? |  |  |  |
|  | 24 | Were at least two researchers involved in the analysis? |  |  |  |
|  | 25 | Were problems regarding the comprehensibility of the PROM instructions, items, response options, and recall period appropriately addressed by adapting the PROM? |  |  |  |
| Comprehensiveness | 26 | Were patients asked about the comprehensiveness of the PROM? |  |  |  |
|  | 27 | Was the final set of items tested? |  |  |  |
|  | 28 | Was an appropriate method used for assessing the comprehensiveness of the PROM? |  |  |  |
|  | 29 | Was each item tested in an appropriate number of patients? |  |  |  |
|  |  | For qualitative studies |  |  |  |
|  |  | For quantitative (survey) studies |  |  |  |
|  | 30 | Were skilled interviewers used? |  |  |  |
|  | 31 | Were the interviews based on an appropriate interview guide? |  |  |  |
|  | 32 | Were the interviews recorded and transcribed verbatim? |  |  |  |
|  | 33 | Was an appropriate approach used to analyse the data? |  |  |  |
|  | 34 | Were at least two researchers involved in the analysis? |  |  |  |
|  | 35 | Were problems regarding the comprehensiveness of the PROM appropriately addressed by adapting the PROM? |  |  |  |
| CONSENSUS | | |  |  | **I** |
| Box 2. Content validity | | | | | |
| 2a. Asking patients about relevance | | | | | |
| Design requirements | 1 | Was an appropriate method used to ask patients whether each item is relevant for their experience with the condition? |  |  |  |
|  | 2 | Was each item tested in an appropriate number of patients? |  |  |  |
|  |  | For qualitative studies |  |  |  |
|  |  | For quantitative (survey) studies |  |  |  |
|  | 3 | Were skilled group moderators/interviewers used? |  |  |  |
|  | 4 | Were the group meetings or interviews based on an appropriate topic or interview guide? |  |  |  |
|  | 5 | Were the group meetings or interviews recorded and transcribed verbatim? |  |  |  |
| Analyses | 6 | Was an appropriate approach used to analyse the data? |  |  |  |
|  | 7 | Were at least two researchers involved in the analysis? |  |  |  |
| 2b. Asking patients about comprehensiveness | | | | | |
| Design requirements | 8 | Was an appropriate method used for assessing the comprehensiveness of the PROM? |  |  |  |
|  | 9 | Was each item tested in an appropriate number of patients? |  |  |  |
|  |  | For qualitative studies |  |  |  |
|  |  | For quantitative (survey) studies |  |  |  |
|  | 10 | Were skilled group moderators/interviewers used? |  |  |  |
|  | 11 | Were the group meetings or interviews based on an appropriate topic or interview guide? |  |  |  |
|  | 12 | Were the group meetings or interviews recorded and transcribed verbatim? |  |  |  |
| Analyses | 13 | Was an appropriate approach used to analyse the data? |  |  |  |
|  | 14 | Were at least two researchers involved in the analysis? |  |  |  |
| 2c. Asking patients about comprehensibility | | | | | |
| Design requirements | 15 | Was an appropriate qualitative method used for assessing the comprehensibility of the PROM instructions, items, response options, and recall period? |  |  |  |
|  | 16 | Was each item tested in an appropriate number of patients? |  |  |  |
|  |  | For qualitative studies |  |  |  |
|  |  | For quantitative (survey) studies |  |  |  |
|  | 17 | Were skilled group moderators/interviewers used? |  |  |  |
|  | 18 | Were the group meetings or interviews based on an appropriate topic or interview guide? |  |  |  |
|  | 19 | Were the group meetings or interviews recorded and transcribed verbatim? |  |  |  |
| Analyses | 20 | Was an appropriate approach used to analyse the data? |  |  |  |
|  | 21 | Were at least two researchers involved in the analysis? |  |  |  |
| 2d. Asking professionals about relevance | | | | | |
| Design requirements | 22 | Was an appropriate method used to ask professionals whether each item is relevant for the construct of interest? |  |  |  |
|  | 23 | Were professionals from all relevant disciplines included? |  |  |  |
|  | 24 | Was each item tested in an appropriate number of professionals? |  |  |  |
|  |  | For qualitative studies |  |  |  |
|  |  | For quantitative (survey) studies |  |  |  |
| Analyses | 25 | Was an appropriate approach used to analyse the data? |  |  |  |
|  | 26 | Were at least two researchers involved in the analysis? |  |  |  |
| 2e. Asking professionals about comprehensiveness | | | | | |
| Design requirements | 27 | Was an appropriate method used for assessing the comprehensiveness of the PROM? |  |  |  |
|  | 28 | Were professionals from all relevant disciplines included? |  |  |  |
|  | 29 | Was each item tested in an appropriate number of professionals? |  |  |  |
|  |  | For qualitative studies |  |  |  |
|  |  | For quantitative (survey) studies |  |  |  |
| Analyses | 30 | Was an appropriate approach used to analyse the data? |  |  |  |
|  | 31 | Were at least two researchers involved in the analysis? |  |  |  |
| CONSENSUS | | |  |  |  |
| Box 3. Structural validity | | | | | |
|  |  | Does the scale consist of effect indicators, i.e. is it based on a reflective model? |  |  |  |
|  |  | Does the study concern unidimensionality or structural validity? |  |  |  |
| Statistical methods | 1 | For CTT: Was exploratory or confirmatory factor analysis performed? |  |  |  |
|  | 2 | For IRT/Rasch: does the chosen model fit to the research question? |  |  |  |
|  | 3 | Was the sample size included in the analysis A? |  |  |  |
|  |  | For FA |  |  |  |
|  |  | For Rasch/1PL models |  |  |  |
|  |  | For 2PL parametric IRT models OR Mokken scale analysis |  |  |  |
| Other | 4 | Were there any other important flaws in the design or statistical methods of the study? |  |  |  |
| CONSENSUS | | |  |  |  |
| Box 4. Internal consistency | | | | | |
|  |  | Does the scale consist of effect indicators, i.e. is it based on a reflective model? |  |  |  |
| Design requirements | 1 | Was an internal consistency statistic calculated for each unidimensional scale or subscale separately? |  |  |  |
| Statistical methods | 2 | For continuous scores: Was Cronbach’s alpha or omega calculated? |  |  |  |
|  | 3 | For dichotomous scores: Was Cronbach’s alpha or KR-20 calculated? |  |  |  |
|  | 4 | For IRT-based scores: Was standard error of the theta (SE (θ)) or reliability coefficient of estimated latent trait value (index of (subject or item) separation) calculated? |  |  |  |
| Other | 5 | Were there any other important flaws in the design or statistical methods of the study? |  |  |  |
| CONSENSUS | | |  |  |  |
| Box 5. Cross-cultural validity/Measurement invariance | | | | | |
| Design requirements | 1 | Were the samples similar for relevant characteristics except for the group variable? |  |  |  |
| Statistical methods | 2 | Was an appropriate approach used to analyse the data? |  |  |  |
|  | 3 | Was the sample size included in the analysis A? |  |  |  |
|  |  | For Regression analyses or IRT/Rasch based analyses |  |  |  |
|  |  | For MGCFA* |  |  |  |
| Other | 4 | Were there any other important flaws in the design or statistical methods of the study? |  |  |  |
| CONSENS | | |  |  |  |
| Box 6. Reliability | | | | | |
| Design requirements | 1 | Were patients stable in the interim period on the construct to be measured? | A | VG | **A** |
|  | 2 | Was the time interval appropriate? | VG | VG | **VG** |
|  | 3 | Were the test conditions similar for the measurements? e.g. type of administration, environment, instructions | A | A | **A** |
| Statistical methods | 4 | For continuous scores: Was an intraclass correlation coefficient (ICC) calculated? | I | D | **IA** |
|  | 5 | For dichotomous/nominal/ordinal scores: Was kappa calculated? | N/A |  | **N/A** |
|  | 6 | For ordinal scores: Was a weighted kappa calculated? | N/A |  | **N/A** |
|  | 7 | For ordinal scores: Was the weighting scheme described? e.g. linear, quadratic | N/A |  | **N/A** |
| Other | 8 | Were there any other important flaws in the design or statistical methods of the study? | VG | VG | **VG** |
| CONSENSUS | | |  |  | **A** |
| Box 7. Measurement error | | | | | |
| Design requirements | 1 | Were patients stable in the interim period on the construct to be measured? |  |  |  |
|  | 2 | Was the time interval appropriate? |  |  |  |
|  | 3 | Were the test conditions similar for the measurements? e.g. type of administration, environment, instructions |  |  |  |
| Statistical methods | 4 | For continuous scores: Was the Standard Error of Measurement (SEM), Smallest Detectable Change (SDC) or Limits of Agreement (LoA) calculated? |  |  |  |
|  | 5 | For dichotomous/nominal/ordinal scores: Was the percentage (positive and negative) agreement calculated? |  |  |  |
| Other | 6 | Were there any other important flaws in the design or statistical methods of the study? |  |  |  |
| CONSENSUS | | |  |  |  |
| Box 8. Criterion validity | | | | | |
| Statistical methods | 1 | For continuous scores: Were correlations, or the area under the receiver operating curve calculated? |  |  |  |
|  | 2 | For dichotomous scores: Were sensitivity and specificity determined? |  |  |  |
| Other | 3 | Were there any other important flaws in the design or statistical methods of the study? |  |  |  |
| CONSENSUS | | |  |  |  |
| Box 9. Hypotheses testing for construct validity | | | | | |
| 9a. Comparison with other outcome measurement instruments (convergent validity) |  |  |  |  |  |
| Design requirements | 1 | Is it clear what the comparator instrument(s) measure(s)? |  |  |  |
|  | 2 | Were the measurement properties of the comparator instrument(s) sufficient? |  |  |  |
| Statistical methods | 3 | Was the statistical method appropriate for the hypotheses to be tested? |  |  |  |
| Other | 4 | Were there any other important flaws in the design or statistical methods of the study? |  |  |  |
| CONSENSUS | | |  |  |  |
| 9b. Comparison between subgroups (discriminative or known-groups validity) | | | | | |
| Design requirements | 5 | Was an A description provided of important characteristics of the subgroups? |  |  |  |
| Statistical methods | 6 | Was the statistical method appropriate for the hypotheses to be tested? |  |  |  |
| Other | 7 | Were there any other important flaws in the design or statistical methods of the study? |  |  |  |
| CONSENSUS | | |  |  |  |
| Box 10. Responsiveness | | | | | |
| 10a. Criterion approach (i.e. comparison to a gold standard) | | | | | |
| Statistical methods | 1 | For continuous scores: Were correlations between change scores, or the area under the Receiver Operator Curve (ROC) curve calculated? |  |  |  |
|  | 2 | For dichotomous scales: Were sensitivity and specificity (changed versus not changed) determined? |  |  |  |
| Other | 3 | Were there any other important flaws in the design or statistical methods of the study? |  |  |  |
| CONSENSUS | | |  |  |  |
| 10b. Construct approach (i.e. hypotheses testing; comparison with other outcome measurement instruments) | | | | | |
| Design requirements | 4 | Is it clear what the comparator instrument(s) measure(s)? |  |  |  |
|  | 5 | Were the measurement properties of the comparator instrument(s) sufficient? |  |  |  |
| Statistical methods | 6 | Was the statistical method appropriate for the hypotheses to be tested? |  |  |  |
| Other | 7 | Were there any other important flaws in the design or statistical methods of the study? |  |  |  |
| CONSENSUS | | |  |  |  |
| 10c. Construct approach (i.e. hypotheses testing: comparison between subgroups) | | | | | |
| Design requirements | 8 | Was an A description provided of important characteristics of the subgroups? |  |  |  |
| Statistical methods | 9 | Was the statistical method appropriate for the hypotheses to be tested? |  |  |  |
| Other | 10 | Were there any other important flaws in the design or statistical methods of the study? |  |  |  |
| CONSENSUS | | |  |  |  |
| 10d. Construct approach (i.e. hypotheses testing: before and after intervention) | | | | | |
| Design requirements | 11 | Was an A description provided of the intervention given? |  |  |  |
| Statistical methods | 12 | Was the statistical method appropriate for the hypotheses to be tested? |  |  |  |
| Other | 13 | Were there any other important flaws in the design or statistical methods of the study? |  |  |  |
| CONSENSUS | | |  |  |  |
| % | | | **60.00%** | | |

A: adequate; C: consensus; D: doubtful; I: inadequate; N/A: not applicable; R1: Reviewer 1; R2: Reviewer 2; VG: very good

| QUAL-E | | | | | | | | | | | |
| --- | --- | --- | --- | --- | --- | --- | --- | --- | --- | --- | --- |
| COSMIN Risk of Bias Checklist | | | Steinhauser et al., 2002 | | | Steinhauser et al., 2004 | | | Wilkinson et al., 2014 | | |
|  | Item | Requirements | R1 | R2 | **C** | R1 | R2 | **C** | R1 | R2 | **C** |
| Box 1. PROM Development | | | | | | | | | | | |
| 1a. PROM design | | | | | | | | | | | |
|  | 1 | Is a clear description provided of the construct to be measured? | VG | VG | **VG** |  |  |  |  |  |  |
|  | 2 | Is the origin of the construct clear: was a theory, conceptual framework or disease model used or clear rationale provided to define the construct to be measured? | D | VG | **VG** |  |  |  |  |  |  |
|  | 3 | Is a clear description provided of the target population for which the PROM was developed? | VG | VG | **VG** |  |  |  |  |  |  |
|  | 4 | Is a clear description provided of the context of use | VG | VG | **VG** |  |  |  |  |  |  |
|  | 5 | Was the PROM development study performed in a sample representing the target population for which the PROM was developed? | VG | VG | **VG** |  |  |  |  |  |  |
|  | 6 | Was an appropriate qualitative data collection method used to identify relevant items for a new PROM? | VG | VG | **VG** |  |  |  |  |  |  |
|  | 7 | Were skilled group moderators/interviewers used? | D | D | **D** |  |  |  |  |  |  |
|  | 8 | Were the group meetings or interviews based on an appropriate topic or interview guide? | VG | D | **VG** |  |  |  |  |  |  |
|  | 9 | Were the group meetings or interviews recorded and transcribed verbatim? | VG | D | **VG** |  |  |  |  |  |  |
|  | 10 | Was an appropriate approach used to analyse the data? | VG | D | **VG** |  |  |  |  |  |  |
|  | 11 | Was at least part of the data coded independently? | A | D | **A** |  |  |  |  |  |  |
|  | 12 | Was data collection continued until saturation was reached? | A | D | **A** |  |  |  |  |  |  |
|  | 13 | For quantitative studies (surveys): was the sample size appropriate? | VG | N/A | **VG** |  |  |  |  |  |  |
| 1b. Cognitive interview study or pilot test | | | | | | | | | | | |
|  | 14 | Was a cognitive interview study or other pilot test conducted? | VG | VG | **VG** |  |  |  |  |  |  |
| General design requirements | 15 | Was the cognitive interview study or other pilot test performed in a sample representing the target population? | VG | VG | **VG** |  |  |  |  |  |  |
| Comprehensibility | 16 | Were patients asked about the comprehensibility of the PROM? | D | VG | **VG** |  |  |  |  |  |  |
|  | 17 | Were all items tested in their final form? |  | VG | **VG** |  |  |  |  |  |  |
|  | 18 | Was an appropriate qualitative method used to assess the comprehensibility of the PROM instructions, items, response options, and recall period? |  | VG | **VG** |  |  |  |  |  |  |
|  | 19 | Was each item tested in an appropriate number of patients? |  |  |  |  |  |  |  |  |  |
|  |  | For qualitative studies |  | VG | **VG** |  |  |  |  |  |  |
|  |  | For quantitative (survey) studies |  |  |  |  |  |  |  |  |  |
|  | 20 | Were skilled interviewers used? |  | D | **VG** |  |  |  |  |  |  |
|  | 21 | Were the interviews based on an appropriate interview guide? |  | D | **VG** |  |  |  |  |  |  |
|  | 22 | Were the interviews recorded and transcribed verbatim? |  | D | **VG** |  |  |  |  |  |  |
|  | 23 | Was an appropriate approach used to analyse the data? |  | D | **VG** |  |  |  |  |  |  |
|  | 24 | Were at least two researchers involved in the analysis? |  | D | **VG** |  |  |  |  |  |  |
|  | 25 | Were problems regarding the comprehensibility of the PROM instructions, items, response options, and recall period appropriately addressed by adapting the PROM? |  | VG | **VG** |  |  |  |  |  |  |
| Comprehensiveness | 26 | Were patients asked about the comprehensiveness of the PROM? | VG | D | **D** |  |  |  |  |  |  |
|  | 27 | Was the final set of items tested? | A |  |  |  |  |  |  |  |  |
|  | 28 | Was an appropriate method used for assessing the comprehensiveness of the PROM? | A |  |  |  |  |  |  |  |  |
|  | 29 | Was each item tested in an appropriate number of patients? |  |  |  |  |  |  |  |  |  |
|  |  | For qualitative studies | VG |  |  |  |  |  |  |  |  |
|  |  | For quantitative (survey) studies |  |  |  |  |  |  |  |  |  |
|  | 30 | Were skilled interviewers used? | D |  |  |  |  |  |  |  |  |
|  | 31 | Were the interviews based on an appropriate interview guide? | D |  |  |  |  |  |  |  |  |
|  | 32 | Were the interviews recorded and transcribed verbatim? | D |  |  |  |  |  |  |  |  |
|  | 33 | Was an appropriate approach used to analyse the data? | A |  |  |  |  |  |  |  |  |
|  | 34 | Were at least two researchers involved in the analysis? | D |  |  |  |  |  |  |  |  |
|  | 35 | Were problems regarding the comprehensiveness of the PROM appropriately addressed by adapting the PROM? | A |  |  |  |  |  |  |  |  |
| CONSENSUS | | |  |  | **D** |  |  |  |  |  |  |
| Box 2. Content validity | | | | | | | | | | | |
| 2a. Asking patients about relevance | | | | | | | | | | | |
| Design requirements | 1 | Was an appropriate method used to ask patients whether each item is relevant for their experience with the condition? | I |  |  |  |  |  | VG |  |  |
|  | 2 | Was each item tested in an appropriate number of patients? |  |  |  |  |  |  |  |  |  |
|  |  | For qualitative studies | D |  |  |  |  |  | VG |  |  |
|  |  | For quantitative (survey) studies |  |  |  |  |  |  |  |  |  |
|  | 3 | Were skilled group moderators/interviewers used? | D |  |  |  |  |  | VG |  |  |
|  | 4 | Were the group meetings or interviews based on an appropriate topic or interview guide? | D |  |  |  |  |  | A |  |  |
|  | 5 | Were the group meetings or interviews recorded and transcribed verbatim? | I |  |  |  |  |  | VG |  |  |
| Analyses | 6 | Was an appropriate approach used to analyse the data? | D |  |  |  |  |  | VG |  |  |
|  | 7 | Were at least two researchers involved in the analysis? | D |  |  |  |  |  | VG |  |  |
| 2b. Asking patients about comprehensiveness | | | | | | | | | | | |
| Design requirements | 8 | Was an appropriate method used for assessing the comprehensiveness of the PROM? | I |  |  |  |  |  |  | VG |  |
|  | 9 | Was each item tested in an appropriate number of patients? |  |  |  |  |  |  |  |  |  |
|  |  | For qualitative studies | D |  |  |  |  |  |  | VG |  |
|  |  | For quantitative (survey) studies |  |  |  |  |  |  |  |  |  |
|  | 10 | Were skilled group moderators/interviewers used? | D |  |  |  |  |  |  | VG |  |
|  | 11 | Were the group meetings or interviews based on an appropriate topic or interview guide? | D |  |  |  |  |  |  | VG |  |
|  | 12 | Were the group meetings or interviews recorded and transcribed verbatim? | I |  |  |  |  |  |  | VG |  |
| Analyses | 13 | Was an appropriate approach used to analyse the data? | D |  |  |  |  |  |  | VG |  |
|  | 14 | Were at least two researchers involved in the analysis? | D |  |  |  |  |  |  | VG |  |
| 2c. Asking patients about comprehensibility | | | | | | | | | | | |
| Design requirements | 15 | Was an appropriate qualitative method used for assessing the comprehensibility of the PROM instructions, items, response options, and recall period? | I |  |  |  |  |  | VG |  | **VG** |
|  | 16 | Was each item tested in an appropriate number of patients? |  |  |  |  |  |  |  |  |  |
|  |  | For qualitative studies | D |  |  |  |  |  | VG |  | **VG** |
|  |  | For quantitative (survey) studies |  |  |  |  |  |  |  |  |  |
|  | 17 | Were skilled group moderators/interviewers used? | D |  |  |  |  |  | VG |  | **VG** |
|  | 18 | Were the group meetings or interviews based on an appropriate topic or interview guide? | D |  |  |  |  |  | A |  | **A** |
|  | 19 | Were the group meetings or interviews recorded and transcribed verbatim? | I |  |  |  |  |  | VG |  | **VG** |
| Analyses | 20 | Was an appropriate approach used to analyse the data? | I |  |  |  |  |  | VG |  | **VG** |
|  | 21 | Were at least two researchers involved in the analysis? | D |  |  |  |  |  | VG |  | **VG** |
| 2d. Asking professionals about relevance | | | | | | | | | | | |
| Design requirements | 22 | Was an appropriate method used to ask professionals whether each item is relevant for the construct of interest? | I |  |  |  |  |  | VG |  |  |
|  | 23 | Were professionals from all relevant disciplines included? | D |  |  |  |  |  | A |  |  |
|  | 24 | Was each item tested in an appropriate number of professionals? |  |  |  |  |  |  |  |  |  |
|  |  | For qualitative studies | D |  |  |  |  |  | D |  |  |
|  |  | For quantitative (survey) studies |  |  |  |  |  |  |  |  |  |
| Analyses | 25 | Was an appropriate approach used to analyse the data? | I |  |  |  |  |  | VG |  |  |
|  | 26 | Were at least two researchers involved in the analysis? | D |  |  |  |  |  | VG |  |  |
| 2e. Asking professionals about comprehensiveness | | | | | | | | | | | |
| Design requirements | 27 | Was an appropriate method used for assessing the comprehensiveness of the PROM? | I |  |  |  |  |  |  |  |  |
|  | 28 | Were professionals from all relevant disciplines included? | D |  |  |  |  |  |  |  |  |
|  | 29 | Was each item tested in an appropriate number of professionals? |  |  |  |  |  |  |  |  |  |
|  |  | For qualitative studies | D |  |  |  |  |  |  |  |  |
|  |  | For quantitative (survey) studies |  |  |  |  |  |  |  |  |  |
| Analyses | 30 | Was an appropriate approach used to analyse the data? | I |  |  |  |  |  |  |  |  |
|  | 31 | Were at least two researchers involved in the analysis? | D |  |  |  |  |  |  |  |  |
| CONSENSUS |  |  |  |  |  |  |  |  |  |  | **A** |
| Box 3. Structural validity | | | | | | | | | | | |
|  |  | Does the scale consist of effect indicators, i.e. is it based on a reflective model? | Y | Y | **Y** | Y | Y | **Y** |  |  |  |
|  |  | Does the study concern unidimensionality or structural validity? | SV | SV | **SV** | SV | SV | **SV** |  |  |  |
| Statistical methods | 1 | For CTT: Was exploratory or confirmatory factor analysis performed? | A | A | **A** | VG | VG | **VG** |  |  |  |
|  | 2 | For IRT/Rasch: does the chosen model fit to the research question? | N/A | N/A | **N/A** | N/A | N/A | **N/A** |  |  |  |
|  | 3 | Was the sample size included in the analysis A? |  |  |  |  |  |  |  |  |  |
|  |  | For FA | VG | I | **VG** | VG | VG | **VG** |  |  |  |
|  |  | For Rasch/1PL models |  |  |  |  |  |  |  |  |  |
|  |  | For 2PL parametric IRT models OR Mokken scale analysis |  |  |  |  |  |  |  |  |  |
| Other | 4 | Were there any other important flaws in the design or statistical methods of the study? | VG | VG | **VG** |  | VG |  |  |  |  |
| CONSENSUS | | |  |  | **A** |  |  | **VG** |  |  |  |
| Box 4. Internal consistency | | | | | | | | | | | |
|  |  | Does the scale consist of effect indicators, i.e. is it based on a reflective model? | Y |  | **Y** |  | Y | **Y** |  |  |  |
| Design requirements | 1 | Was an internal consistency statistic calculated for each unidimensional scale or subscale separately? | VG | VG | **VG** | VG | VG | **VG** |  |  |  |
| Statistical methods | 2 | For continuous scores: Was Cronbach’s alpha or omega calculated? | VG | VG | **VG** | VG | VG | **VG** |  |  |  |
|  | 3 | For dichotomous scores: Was Cronbach’s alpha or KR-20 calculated? | N/A | N/A | **N/A** |  | N/A | **N/A** |  |  |  |
|  | 4 | For IRT-based scores: Was standard error of the theta (SE (θ)) or reliability coefficient of estimated latent trait value (index of (subject or item) separation) calculated? | N/A | N/A | **N/A** |  | N/A | **N/A** |  |  |  |
| Other | 5 | Were there any other important flaws in the design or statistical methods of the study? | VG | VG | **VG** |  | VG | **N/A** |  |  |  |
| CONSENSUS | | |  |  | **VG** |  |  | **VG** |  |  |  |
| Box 5. Cross-cultural validity/Measurement invariance | | | | | | | | | | | |
| Design requirements | 1 | Were the samples similar for relevant characteristics except for the group variable? |  |  |  |  |  |  |  |  |  |
| Statistical methods | 2 | Was an appropriate approach used to analyse the data? |  |  |  |  |  |  |  |  |  |
|  | 3 | Was the sample size included in the analysis A? |  |  |  |  |  |  |  |  |  |
|  |  | For Regression analyses or IRT/Rasch based analyses |  |  |  |  |  |  |  |  |  |
|  |  | For MGCFA* |  |  |  |  |  |  |  |  |  |
| Other | 4 | Were there any other important flaws in the design or statistical methods of the study? |  |  |  |  |  |  |  |  |  |
| CONSENSUS | | |  |  |  |  |  |  |  |  |  |
| Box 6. Reliability | | | | | | | | | | | |
| Design requirements | 1 | Were patients stable in the interim period on the construct to be measured? |  |  |  | D | D | **D** |  |  |  |
|  | 2 | Was the time interval appropriate? |  |  |  | VG | D | **VG** |  |  |  |
|  | 3 | Were the test conditions similar for the measurements? e.g. type of administration, environment, instructions |  |  |  | A | D | **D** |  |  |  |
| Statistical methods | 4 | For continuous scores: Was an intraclass correlation coefficient (ICC) calculated? |  |  |  | A | A | **A** |  |  |  |
|  | 5 | For dichotomous/nominal/ordinal scores: Was kappa calculated? |  |  |  |  | N/A | **N/A** |  |  |  |
|  | 6 | For ordinal scores: Was a weighted kappa calculated? |  |  |  |  | N/A | **N/A** |  |  |  |
|  | 7 | For ordinal scores: Was the weighting scheme described? e.g. linear, quadratic |  |  |  |  | N/A | **N/A** |  |  |  |
| Other | 8 | Were there any other important flaws in the design or statistical methods of the study? |  |  |  |  | VG | **VG** |  |  |  |
| CONSENSUS | | |  |  |  |  |  | **D** |  |  |  |
| Box 7. Measurement error | | | | | | | | | | | |
| Design requirements | 1 | Were patients stable in the interim period on the construct to be measured? |  |  |  | D |  |  |  |  |  |
|  | 2 | Was the time interval appropriate? |  |  |  |  |  |  |  |  |  |
|  | 3 | Were the test conditions similar for the measurements? e.g. type of administration, environment, instructions |  |  |  |  |  |  |  |  |  |
| Statistical methods | 4 | For continuous scores: Was the Standard Error of Measurement (SEM), Smallest Detectable Change (SDC) or Limits of Agreement (LoA) calculated? |  |  |  |  |  |  |  |  |  |
|  | 5 | For dichotomous/nominal/ordinal scores: Was the percentage (positive and negative) agreement calculated? |  |  |  |  |  |  |  |  |  |
| Other | 6 | Were there any other important flaws in the design or statistical methods of the study? |  |  |  |  |  |  |  |  |  |
| CONSENSUS | | |  |  |  |  |  |  |  |  |  |
| Box 8. Criterion validity | | | | | | | | | | | |
| Statistical methods | 1 | For continuous scores: Were correlations, or the area under the receiver operating curve calculated? |  |  |  |  |  |  |  |  |  |
|  | 2 | For dichotomous scores: Were sensitivity and specificity determined? |  |  |  |  |  |  |  |  |  |
| Other | 3 | Were there any other important flaws in the design or statistical methods of the study? |  |  |  |  |  |  |  |  |  |
| CONSENSUS | | |  |  |  |  |  |  |  |  |  |
| Box 9. Hypotheses testing for construct validity | | | | | | | | | | | |
| 9a. Comparison with other outcome measurement instruments (convergent validity) | | | | | | | | | | | |
| Design requirements | 1 | Is it clear what the comparator instrument(s) measure(s)? |  |  |  | VG | VG | **VG** |  |  |  |
|  | 2 | Were the measurement properties of the comparator instrument(s) sufficient? |  |  |  |  | D | **D** |  |  |  |
| Statistical methods | 3 | Was the statistical method appropriate for the hypotheses to be tested? |  |  |  |  | A | **A** |  |  |  |
| Other | 4 | Were there any other important flaws in the design or statistical methods of the study? |  |  |  |  | VG | **VG** |  |  |  |
| CONSENSUS | | |  |  |  |  |  | **D** |  |  |  |
| 9b. Comparison between subgroups (discriminative or known-groups validity) | | | | | | | | | | | |
| Design requirements | 5 | Was an A description provided of important characteristics of the subgroups? |  |  |  |  |  |  |  |  |  |
| Statistical methods | 6 | Was the statistical method appropriate for the hypotheses to be tested? |  |  |  |  |  |  |  |  |  |
| Other | 7 | Were there any other important flaws in the design or statistical methods of the study? |  |  |  |  |  |  |  |  |  |
| CONSENSUS | | |  |  |  |  |  |  |  |  |  |
| Box 10. Responsiveness | | | | | | | | | | | |
| 10a. Criterion approach (i.e. comparison to a gold standard) | | | | | | | | | | | |
| Statistical methods | 1 | For continuous scores: Were correlations between change scores, or the area under the Receiver Operator Curve (ROC) curve calculated? |  |  |  |  |  |  |  |  |  |
|  | 2 | For dichotomous scales: Were sensitivity and specificity (changed versus not changed) determined? |  |  |  |  |  |  |  |  |  |
| Other | 3 | Were there any other important flaws in the design or statistical methods of the study? |  |  |  |  |  |  |  |  |  |
| CONSENSUS | | |  |  |  |  |  |  |  |  |  |
| 10b. Construct approach (i.e. hypotheses testing; comparison with other outcome measurement instruments) | | | | | | | | | | | |
| Design requirements | 4 | Is it clear what the comparator instrument(s) measure(s)? |  |  |  |  |  |  |  |  |  |
|  | 5 | Were the measurement properties of the comparator instrument(s) sufficient? |  |  |  |  |  |  |  |  |  |
| Statistical methods | 6 | Was the statistical method appropriate for the hypotheses to be tested? |  |  |  |  |  |  |  |  |  |
| Other | 7 | Were there any other important flaws in the design or statistical methods of the study? |  |  |  |  |  |  |  |  |  |
| CONSENSUS | | |  |  |  |  |  |  |  |  |  |
| 10c. Construct approach (i.e. hypotheses testing: comparison between subgroups) | | | | | | | | | | | |
| Design requirements | 8 | Was an A description provided of important characteristics of the subgroups? |  |  |  |  |  |  |  |  |  |
| Statistical methods | 9 | Was the statistical method appropriate for the hypotheses to be tested? |  |  |  |  |  |  |  |  |  |
| Other | 10 | Were there any other important flaws in the design or statistical methods of the study? |  |  |  |  |  |  |  |  |  |
| CONSENSUS | | |  |  |  |  |  |  |  |  |  |
| 10d. Construct approach (i.e. hypotheses testing: before and after intervention) | | | | | | | | | | | |
| Design requirements | 11 | Was an A description provided of the intervention given? |  |  |  |  |  |  |  |  |  |
| Statistical methods | 12 | Was the statistical method appropriate for the hypotheses to be tested? |  |  |  |  |  |  |  |  |  |
| Other | 13 | Were there any other important flaws in the design or statistical methods of the study? |  |  |  |  |  |  |  |  |  |
| CONSENSUS | | |  |  |  |  |  |  |  |  |  |
| % | | | **23.08%** | | | **40.00%** | | | **0.00%** | | |

A: adequate; C: consensus; D: doubtful; I: inadequate; N/A: not applicable; R1: Reviewer 1; R2: Reviewer 2; SV: structural validity; VG: very good; Y: yes

| QUAL-EC | | | | | |
| --- | --- | --- | --- | --- | --- |
| COSMIN Risk of Bias Checklist | | | Lo et al., 2011 | | |
|  | Item | Requirements | R1 | R2 | **C** |
| Box 1. PROM Development | | | | | |
| 1a. PROM design | | | | | |
|  | 1 | Is a clear description provided of the construct to be measured? | VG | VG | **VG** |
|  | 2 | Is the origin of the construct clear: was a theory, conceptual framework or disease model used or clear rationale provided to define the construct to be measured? | D | VG | **VG** |
|  | 3 | Is a clear description provided of the target population for which the PROM was developed? | VG | VG | **VG** |
|  | 4 | Is a clear description provided of the context of use | VG | VG | **VG** |
|  | 5 | Was the PROM development study performed in a sample representing the target population for which the PROM was developed? | VG | VG | **VG** |
|  | 6 | Was an appropriate qualitative data collection method used to identify relevant items for a new PROM? | VG | VG | **VG** |
|  | 7 | Were skilled group moderators/interviewers used? | D | D | **D** |
|  | 8 | Were the group meetings or interviews based on an appropriate topic or interview guide? | VG | D | **VG** |
|  | 9 | Were the group meetings or interviews recorded and transcribed verbatim? | VG | D | **VG** |
|  | 10 | Was an appropriate approach used to analyse the data? | VG | D | **VG** |
|  | 11 | Was at least part of the data coded independently? | A | D | **A** |
|  | 12 | Was data collection continued until saturation was reached? | A | D | **A** |
|  | 13 | For quantitative studies (surveys): was the sample size appropriate? | VG | N/A | **VG** |
| 1b. Cognitive interview study or pilot test | | | | | |
|  | 14 | Was a cognitive interview study or other pilot test conducted? | VG | VG | **VG** |
| General design requirements | 15 | Was the cognitive interview study or other pilot test performed in a sample representing the target population? | VG | VG | **VG** |
| Comprehensibility | 16 | Were patients asked about the comprehensibility of the PROM? | D | VG | **VG** |
|  | 17 | Were all items tested in their final form? |  | VG | **VG** |
|  | 18 | Was an appropriate qualitative method used to assess the comprehensibility of the PROM instructions, items, response options, and recall period? |  | VG | **VG** |
|  | 19 | Was each item tested in an appropriate number of patients? |  |  |  |
|  |  | For qualitative studies |  | VG | **VG** |
|  |  | For quantitative (survey) studies |  |  |  |
|  | 20 | Were skilled interviewers used? | VG | D | **VG** |
|  | 21 | Were the interviews based on an appropriate interview guide? |  | D | **VG** |
|  | 22 | Were the interviews recorded and transcribed verbatim? |  | D | **VG** |
|  | 23 | Was an appropriate approach used to analyse the data? |  | D | **VG** |
|  | 24 | Were at least two researchers involved in the analysis? |  | D | **VG** |
|  | 25 | Were problems regarding the comprehensibility of the PROM instructions, items, response options, and recall period appropriately addressed by adapting the PROM? |  | VG | **VG** |
| Comprehensiveness | 26 | Were patients asked about the comprehensiveness of the PROM? | VG | D | **D** |
|  | 27 | Was the final set of items tested? | A |  |  |
|  | 28 | Was an appropriate method used for assessing the comprehensiveness of the PROM? | A |  |  |
|  | 29 | Was each item tested in an appropriate number of patients? |  |  |  |
|  |  | For qualitative studies | VG |  |  |
|  |  | For quantitative (survey) studies |  |  |  |
|  | 30 | Were skilled interviewers used? | D |  |  |
|  | 31 | Were the interviews based on an appropriate interview guide? | D |  |  |
|  | 32 | Were the interviews recorded and transcribed verbatim? | D |  |  |
|  | 33 | Was an appropriate approach used to analyse the data? | A |  |  |
|  | 34 | Were at least two researchers involved in the analysis? | D |  |  |
|  | 35 | Were problems regarding the comprehensiveness of the PROM appropriately addressed by adapting the PROM? | A |  |  |
| CONSENSUS | | |  |  | **D** |
| Box 2. Content validity | | | | | |
| 2a. Asking patients about relevance | | | | | |
| Design requirements | 1 | Was an appropriate method used to ask patients whether each item is relevant for their experience with the condition? |  |  |  |
|  | 2 | Was each item tested in an appropriate number of patients? |  |  |  |
|  |  | For qualitative studies |  |  |  |
|  |  | For quantitative (survey) studies |  |  |  |
|  | 3 | Were skilled group moderators/interviewers used? |  |  |  |
|  | 4 | Were the group meetings or interviews based on an appropriate topic or interview guide? |  |  |  |
|  | 5 | Were the group meetings or interviews recorded and transcribed verbatim? |  |  |  |
| Analyses | 6 | Was an appropriate approach used to analyse the data? |  |  |  |
|  | 7 | Were at least two researchers involved in the analysis? |  |  |  |
| 2b. Asking patients about comprehensiveness | | | | | |
| Design requirements | 8 | Was an appropriate method used for assessing the comprehensiveness of the PROM? |  |  |  |
|  | 9 | Was each item tested in an appropriate number of patients? |  |  |  |
|  |  | For qualitative studies |  |  |  |
|  |  | For quantitative (survey) studies |  |  |  |
|  | 10 | Were skilled group moderators/interviewers used? |  |  |  |
|  | 11 | Were the group meetings or interviews based on an appropriate topic or interview guide? |  |  |  |
|  | 12 | Were the group meetings or interviews recorded and transcribed verbatim? |  |  |  |
| Analyses | 13 | Was an appropriate approach used to analyse the data? |  |  |  |
|  | 14 | Were at least two researchers involved in the analysis? |  |  |  |
| 2c. Asking patients about comprehensibility | | | | | |
| Design requirements | 15 | Was an appropriate qualitative method used for assessing the comprehensibility of the PROM instructions, items, response options, and recall period? |  |  |  |
|  | 16 | Was each item tested in an appropriate number of patients? |  |  |  |
|  |  | For qualitative studies |  |  |  |
|  |  | For quantitative (survey) studies |  |  |  |
|  | 17 | Were skilled group moderators/interviewers used? |  |  |  |
|  | 18 | Were the group meetings or interviews based on an appropriate topic or interview guide? |  |  |  |
|  | 19 | Were the group meetings or interviews recorded and transcribed verbatim? |  |  |  |
| Analyses | 20 | Was an appropriate approach used to analyse the data? |  |  |  |
|  | 21 | Were at least two researchers involved in the analysis? |  |  |  |
| 2d. Asking professionals about relevance | | | | | |
| Design requirements | 22 | Was an appropriate method used to ask professionals whether each item is relevant for the construct of interest? |  |  |  |
|  | 23 | Were professionals from all relevant disciplines included? |  |  |  |
|  | 24 | Was each item tested in an appropriate number of professionals? |  |  |  |
|  |  | For qualitative studies |  |  |  |
|  |  | For quantitative (survey) studies |  |  |  |
| Analyses | 25 | Was an appropriate approach used to analyse the data? |  |  |  |
|  | 26 | Were at least two researchers involved in the analysis? |  |  |  |
| 2e. Asking professionals about comprehensiveness | | | | | |
| Design requirements | 27 | Was an appropriate method used for assessing the comprehensiveness of the PROM? |  |  |  |
|  | 28 | Were professionals from all relevant disciplines included? |  |  |  |
|  | 29 | Was each item tested in an appropriate number of professionals? |  |  |  |
|  |  | For qualitative studies |  |  |  |
|  |  | For quantitative (survey) studies |  |  |  |
| Analyses | 30 | Was an appropriate approach used to analyse the data? |  |  |  |
|  | 31 | Were at least two researchers involved in the analysis? |  |  |  |
| CONSENSUS | | |  |  |  |
| Box 3. Structural validity | | | | | |
|  |  | Does the scale consist of effect indicators, i.e. is it based on a reflective model? | Y | Y | **Y** |
|  |  | Does the study concern unidimensionality or structural validity? | SV | SV | **SV** |
| Statistical methods | 1 | For CTT: Was exploratory or confirmatory factor analysis performed? | VG | VG | **VG** |
|  | 2 | For IRT/Rasch: does the chosen model fit to the research question? |  | N/A | **N/A** |
|  | 3 | Was the sample size included in the analysis A? |  |  |  |
|  |  | For FA | VG | VG | **VG** |
|  |  | For Rasch/1PL models |  |  |  |
|  |  | For 2PL parametric IRT models OR Mokken scale analysis |  |  |  |
| Other | 4 | Were there any other important flaws in the design or statistical methods of the study? | VG | VG | **VG** |
| CONSENSUS | | |  |  | **VG** |
| Box 4. Internal consistency | | | | | |
|  |  | Does the scale consist of effect indicators, i.e. is it based on a reflective model? |  | Y | **Y** |
| Design requirements | 1 | Was an internal consistency statistic calculated for each unidimensional scale or subscale separately? | VG | VG | **VG** |
| Statistical methods | 2 | For continuous scores: Was Cronbach’s alpha or omega calculated? | VG | VG | **VG** |
|  | 3 | For dichotomous scores: Was Cronbach’s alpha or KR-20 calculated? | N/A | N/A | **N/A** |
|  | 4 | For IRT-based scores: Was standard error of the theta (SE (θ)) or reliability coefficient of estimated latent trait value (index of (subject or item) separation) calculated? | N/A | N/A | **N/A** |
| Other | 5 | Were there any other important flaws in the design or statistical methods of the study? | VG | VG | **VG** |
| CONSENSUS | | |  |  | **VG** |
| Box 5. Cross-cultural validity/Measurement invariance | | | | | |
| Design requirements | 1 | Were the samples similar for relevant characteristics except for the group variable? |  |  |  |
| Statistical methods | 2 | Was an appropriate approach used to analyse the data? |  |  |  |
|  | 3 | Was the sample size included in the analysis A? |  |  |  |
|  |  | For Regression analyses or IRT/Rasch based analyses |  |  |  |
|  |  | For MGCFA* |  |  |  |
| Other | 4 | Were there any other important flaws in the design or statistical methods of the study? |  |  |  |
| CONSENSUS | | |  |  |  |
| Box 6. Reliability | | | | | |
| Design requirements | 1 | Were patients stable in the interim period on the construct to be measured? |  |  |  |
|  | 2 | Was the time interval appropriate? |  |  |  |
|  | 3 | Were the test conditions similar for the measurements? e.g. type of administration, environment, instructions |  |  |  |
| Statistical methods | 4 | For continuous scores: Was an intraclass correlation coefficient (ICC) calculated? |  |  |  |
|  | 5 | For dichotomous/nominal/ordinal scores: Was kappa calculated? |  |  |  |
|  | 6 | For ordinal scores: Was a weighted kappa calculated? |  |  |  |
|  | 7 | For ordinal scores: Was the weighting scheme described? e.g. linear, quadratic |  |  |  |
| Other | 8 | Were there any other important flaws in the design or statistical methods of the study? |  |  |  |
| CONSENSUS | | |  |  |  |
| Box 7. Measurement error | | | | | |
| Design requirements | 1 | Were patients stable in the interim period on the construct to be measured? |  |  |  |
|  | 2 | Was the time interval appropriate? |  |  |  |
|  | 3 | Were the test conditions similar for the measurements? e.g. type of administration, environment, instructions |  |  |  |
| Statistical methods | 4 | For continuous scores: Was the Standard Error of Measurement (SEM), Smallest Detectable Change (SDC) or Limits of Agreement (LoA) calculated? |  |  |  |
|  | 5 | For dichotomous/nominal/ordinal scores: Was the percentage (positive and negative) agreement calculated? |  |  |  |
| Other | 6 | Were there any other important flaws in the design or statistical methods of the study? |  |  |  |
| CONSENSUS | | |  |  |  |
| Box 8. Criterion validity | | | | | |
| Statistical methods | 1 | For continuous scores: Were correlations, or the area under the receiver operating curve calculated? |  |  |  |
|  | 2 | For dichotomous scores: Were sensitivity and specificity determined? |  |  |  |
| Other | 3 | Were there any other important flaws in the design or statistical methods of the study? |  |  |  |
| CONSENSUS | | |  |  |  |
| Box 9. Hypotheses testing for construct validity | | | | | |
| 9a. Comparison with other outcome measurement instruments (convergent validity) | | | | | |
| Design requirements | 1 | Is it clear what the comparator instrument(s) measure(s)? | VG | VG | **VG** |
|  | 2 | Were the measurement properties of the comparator instrument(s) sufficient? | I | A | **A** |
| Statistical methods | 3 | Was the statistical method appropriate for the hypotheses to be tested? |  | A | **A** |
| Other | 4 | Were there any other important flaws in the design or statistical methods of the study? |  | VG | **VG** |
| CONSENSUS | | |  |  | **A** |
| 9b. Comparison between subgroups (discriminative or known-groups validity) | | | | | |
| Design requirements | 5 | Was an A description provided of important characteristics of the subgroups? |  |  |  |
| Statistical methods | 6 | Was the statistical method appropriate for the hypotheses to be tested? |  |  |  |
| Other | 7 | Were there any other important flaws in the design or statistical methods of the study? |  |  |  |
| CONSENSUS | | |  |  |  |
| Box 10. Responsiveness | | | | | |
| 10a. Criterion approach (i.e. comparison to a gold standard) | | | | | |
| Statistical methods | 1 | For continuous scores: Were correlations between change scores, or the area under the Receiver Operator Curve (ROC) curve calculated? |  |  |  |
|  | 2 | For dichotomous scales: Were sensitivity and specificity (changed versus not changed) determined? |  |  |  |
| Other | 3 | Were there any other important flaws in the design or statistical methods of the study? |  |  |  |
| CONSENSUS | | |  |  |  |
| 10b. Construct approach (i.e. hypotheses testing; comparison with other outcome measurement instruments) | | | | | |
| Design requirements | 4 | Is it clear what the comparator instrument(s) measure(s)? |  |  |  |
|  | 5 | Were the measurement properties of the comparator instrument(s) sufficient? |  |  |  |
| Statistical methods | 6 | Was the statistical method appropriate for the hypotheses to be tested? |  |  |  |
| Other | 7 | Were there any other important flaws in the design or statistical methods of the study? |  |  |  |
| CONSENSUS | | |  |  |  |
| 10c. Construct approach (i.e. hypotheses testing: comparison between subgroups) | | | | | |
| Design requirements | 8 | Was an A description provided of important characteristics of the subgroups? |  |  |  |
| Statistical methods | 9 | Was the statistical method appropriate for the hypotheses to be tested? |  |  |  |
| Other | 10 | Were there any other important flaws in the design or statistical methods of the study? |  |  |  |
| CONSENSUS | | |  |  |  |
| 10d. Construct approach (i.e. hypotheses testing: before and after intervention) | | | | | |
| Design requirements | 11 | Was an A description provided of the intervention given? |  |  |  |
| Statistical methods | 12 | Was the statistical method appropriate for the hypotheses to be tested? |  |  |  |
| Other | 13 | Were there any other important flaws in the design or statistical methods of the study? |  |  |  |
| CONSENSUS | | |  |  |  |
| % | | | 38.00% | | |

A: adequate; C: consensus; D: doubtful; I: inadequate; N/A: not applicable; R1: Reviewer 1; R2: Reviewer 2; SV: structural validity; VG: very good; Y: yes

| SF-LDQ | | | | | | | | | | | | | | |
| --- | --- | --- | --- | --- | --- | --- | --- | --- | --- | --- | --- | --- | --- | --- |
| COSMIN Risk of Bias Checklist | | | Fraser et al., 2007 | | | Gatta et al., 2010 | | | Nkurunziza et al., 2016 | | | Alabdulkareem et al., 2021 | | |
|  | Item | Requirements | R1 | R2 | **C** | R1 | R2 | **C** | R1 | R2 | **C** | R1 | R2 | **C** |
| Box 1. PROM Development | | | | | | | | | | | | | | |
| 1a. PROM design | | | | | | | | | | | | | | |
|  | 1 | Is a clear description provided of the construct to be measured? | VG | VG | **VG** |  |  |  |  |  |  |  |  |  |
|  | 2 | Is the origin of the construct clear: was a theory, conceptual framework or disease model used or clear rationale provided to define the construct to be measured? | VG | VG | **VG** | VG |  |  | VG |  |  | VG |  |  |
|  | 3 | Is a clear description provided of the target population for which the PROM was developed? | VG | VG | **VG** | VG |  |  | VG |  |  | VG |  |  |
|  | 4 | Is a clear description provided of the context of use | VG | VG | **VG** | VG |  |  | VG |  |  | VG |  |  |
|  | 5 | Was the PROM development study performed in a sample representing the target population for which the PROM was developed? | I | I | **I** | I |  |  | I |  |  | I |  |  |
|  | 6 | Was an appropriate qualitative data collection method used to identify relevant items for a new PROM? |  |  |  |  |  |  |  |  |  |  |  |  |
|  | 7 | Were skilled group moderators/interviewers used? |  |  |  |  |  |  |  |  |  |  |  |  |
|  | 8 | Were the group meetings or interviews based on an appropriate topic or interview guide? |  |  |  |  |  |  |  |  |  |  |  |  |
|  | 9 | Were the group meetings or interviews recorded and transcribed verbatim? |  |  |  |  |  |  |  |  |  |  |  |  |
|  | 10 | Was an appropriate approach used to analyse the data? |  |  |  |  |  |  |  |  |  |  |  |  |
|  | 11 | Was at least part of the data coded independently? |  |  |  |  |  |  |  |  |  |  |  |  |
|  | 12 | Was data collection continued until saturation was reached? |  |  |  |  |  |  |  |  |  |  |  |  |
|  | 13 | For quantitative studies (surveys): was the sample size appropriate? | N/A |  |  | N/A |  |  | N/A |  |  | N/A |  |  |
| 1b. Cognitive interview study or pilot test | | | | | | | | | | | | | | |
|  | 14 | Was a cognitive interview study or other pilot test conducted? | VG | VG | **VG** | I |  |  | VG |  |  | VG |  |  |
| General design requirements | 15 | Was the cognitive interview study or other pilot test performed in a sample representing the target population? | VG | VG | **VG** | D |  |  | I |  |  | I |  |  |
| Comprehensibility | 16 | Were patients asked about the comprehensibility of the PROM? | I | I | **I** | I |  |  | I |  |  | D |  |  |
|  | 17 | Were all items tested in their final form? |  |  |  |  |  |  |  |  |  |  |  |  |
|  | 18 | Was an appropriate qualitative method used to assess the comprehensibility of the PROM instructions, items, response options, and recall period? |  |  |  |  |  |  |  |  |  |  |  |  |
|  | 19 | Was each item tested in an appropriate number of patients? |  |  |  |  |  |  |  |  |  |  |  |  |
|  |  | For qualitative studies |  |  |  |  |  |  |  |  |  |  |  |  |
|  |  | For quantitative (survey) studies |  |  |  |  |  |  |  |  |  |  |  |  |
|  | 20 | Were skilled interviewers used? |  |  |  |  |  |  |  |  |  |  |  |  |
|  | 21 | Were the interviews based on an appropriate interview guide? |  |  |  |  |  |  |  |  |  |  |  |  |
|  | 22 | Were the interviews recorded and transcribed verbatim? |  |  |  |  |  |  |  |  |  |  |  |  |
|  | 23 | Was an appropriate approach used to analyse the data? |  |  |  |  |  |  |  |  |  |  |  |  |
|  | 24 | Were at least two researchers involved in the analysis? |  |  |  |  |  |  |  |  |  |  |  |  |
|  | 25 | Were problems regarding the comprehensibility of the PROM instructions, items, response options, and recall period appropriately addressed by adapting the PROM? |  |  |  |  |  |  |  |  |  |  |  |  |
| Comprehensiveness | 26 | Were patients asked about the comprehensiveness of the PROM? | D | D | **D** | D |  |  | D |  |  | D |  |  |
|  | 27 | Was the final set of items tested? |  |  |  |  |  |  |  |  |  |  |  |  |
|  | 28 | Was an appropriate method used for assessing the comprehensiveness of the PROM? |  |  |  |  |  |  |  |  |  |  |  |  |
|  | 29 | Was each item tested in an appropriate number of patients? |  |  |  |  |  |  |  |  |  |  |  |  |
|  |  | For qualitative studies |  |  |  |  |  |  |  |  |  |  |  |  |
|  |  | For quantitative (survey) studies |  |  |  |  |  |  |  |  |  |  |  |  |
|  | 30 | Were skilled interviewers used? |  |  |  |  |  |  |  |  |  |  |  |  |
|  | 31 | Were the interviews based on an appropriate interview guide? |  |  |  |  |  |  |  |  |  |  |  |  |
|  | 32 | Were the interviews recorded and transcribed verbatim? |  |  |  |  |  |  |  |  |  |  |  |  |
|  | 33 | Was an appropriate approach used to analyse the data? |  |  |  |  |  |  |  |  |  |  |  |  |
|  | 34 | Were at least two researchers involved in the analysis? |  |  |  |  |  |  |  |  |  |  |  |  |
|  | 35 | Were problems regarding the comprehensiveness of the PROM appropriately addressed by adapting the PROM? |  |  |  |  |  |  |  |  |  |  |  |  |
| CONSENSUS | | |  |  | **I** |  |  |  |  |  |  |  |  |  |
| Box 2. Content validity | | | | | | | | | | | | | | |
| 2a. Asking patients about relevance | | | | | | | | | | | | | | |
| Design requirements | 1 | Was an appropriate method used to ask patients whether each item is relevant for their experience with the condition? |  |  |  |  |  |  |  |  |  |  |  |  |
|  | 2 | Was each item tested in an appropriate number of patients? |  |  |  |  |  |  |  |  |  |  |  |  |
|  |  | For qualitative studies |  |  |  |  |  |  |  |  |  |  |  |  |
|  |  | For quantitative (survey) studies |  |  |  |  |  |  |  |  |  |  |  |  |
|  | 3 | Were skilled group moderators/interviewers used? |  |  |  |  |  |  |  |  |  |  |  |  |
|  | 4 | Were the group meetings or interviews based on an appropriate topic or interview guide? |  |  |  |  |  |  |  |  |  |  |  |  |
|  | 5 | Were the group meetings or interviews recorded and transcribed verbatim? |  |  |  |  |  |  |  |  |  |  |  |  |
| Analyses | 6 | Was an appropriate approach used to analyse the data? |  |  |  |  |  |  |  |  |  |  |  |  |
|  | 7 | Were at least two researchers involved in the analysis? |  |  |  |  |  |  |  |  |  |  |  |  |
| 2b. Asking patients about comprehensiveness | | | | | | | | | | | | | | |
| Design requirements | 8 | Was an appropriate method used for assessing the comprehensiveness of the PROM? |  |  |  |  |  |  |  |  |  |  |  |  |
|  | 9 | Was each item tested in an appropriate number of patients? |  |  |  |  |  |  |  |  |  |  |  |  |
|  |  | For qualitative studies |  |  |  |  |  |  |  |  |  |  |  |  |
|  |  | For quantitative (survey) studies |  |  |  |  |  |  |  |  |  |  |  |  |
|  | 10 | Were skilled group moderators/interviewers used? |  |  |  |  |  |  |  |  |  |  |  |  |
|  | 11 | Were the group meetings or interviews based on an appropriate topic or interview guide? |  |  |  |  |  |  |  |  |  |  |  |  |
|  | 12 | Were the group meetings or interviews recorded and transcribed verbatim? |  |  |  |  |  |  |  |  |  |  |  |  |
| Analyses | 13 | Was an appropriate approach used to analyse the data? |  |  |  |  |  |  |  |  |  |  |  |  |
|  | 14 | Were at least two researchers involved in the analysis? |  |  |  |  |  |  |  |  |  |  |  |  |
| 2c. Asking patients about comprehensibility | | | | | | | | | | | | | | |
| Design requirements | 15 | Was an appropriate qualitative method used for assessing the comprehensibility of the PROM instructions, items, response options, and recall period? |  | D | **D** |  |  |  |  |  |  |  |  |  |
|  | 16 | Was each item tested in an appropriate number of patients? |  |  |  |  |  |  |  |  |  |  |  |  |
|  |  | For qualitative studies |  |  |  |  |  |  |  |  |  |  |  |  |
|  |  | For quantitative (survey) studies |  | VG | **VG** |  |  |  |  |  |  |  |  |  |
|  | 17 | Were skilled group moderators/interviewers used? |  |  | **D** |  |  |  |  |  |  |  |  |  |
|  | 18 | Were the group meetings or interviews based on an appropriate topic or interview guide? |  |  | **D** |  |  |  |  |  |  |  |  |  |
|  | 19 | Were the group meetings or interviews recorded and transcribed verbatim? |  |  | **D** |  |  |  |  |  |  |  |  |  |
| Analyses | 20 | Was an appropriate approach used to analyse the data? |  | D | **D** |  |  |  |  |  |  |  |  |  |
|  | 21 | Were at least two researchers involved in the analysis? |  | D | **D** |  |  |  |  |  |  |  |  |  |
| 2d. Asking professionals about relevance | | | | | | | | | | | | | | |
| Design requirements | 22 | Was an appropriate method used to ask professionals whether each item is relevant for the construct of interest? |  |  |  |  |  |  |  |  |  |  |  |  |
|  | 23 | Were professionals from all relevant disciplines included? |  |  |  |  |  |  |  |  |  |  |  |  |
|  | 24 | Was each item tested in an appropriate number of professionals? |  |  |  |  |  |  |  |  |  |  |  |  |
|  |  | For qualitative studies |  |  |  |  |  |  |  |  |  |  |  |  |
|  |  | For quantitative (survey) studies |  |  |  |  |  |  |  |  |  |  |  |  |
| Analyses | 25 | Was an appropriate approach used to analyse the data? |  |  |  |  |  |  |  |  |  |  |  |  |
|  | 26 | Were at least two researchers involved in the analysis? |  |  |  |  |  |  |  |  |  |  |  |  |
| 2e. Asking professionals about comprehensiveness | | | | | | | | | | | | | | |
| Design requirements | 27 | Was an appropriate method used for assessing the comprehensiveness of the PROM? |  |  |  |  |  |  |  |  |  |  |  |  |
|  | 28 | Were professionals from all relevant disciplines included? |  |  |  |  |  |  |  |  |  |  |  |  |
|  | 29 | Was each item tested in an appropriate number of professionals? |  |  |  |  |  |  |  |  |  |  |  |  |
|  |  | For qualitative studies |  |  |  |  |  |  |  |  |  |  |  |  |
|  |  | For quantitative (survey) studies |  |  |  |  |  |  |  |  |  |  |  |  |
| Analyses | 30 | Was an appropriate approach used to analyse the data? |  |  |  |  |  |  |  |  |  |  |  |  |
|  | 31 | Were at least two researchers involved in the analysis? |  |  |  |  |  |  |  |  |  |  |  |  |
| CONSENSUS | | |  |  | **D** |  |  |  |  |  |  |  |  |  |
| Box 3. Structural validity | | | | | | | | | | | | | | |
|  |  | Does the scale consist of effect indicators, i.e. is it based on a reflective model? |  |  |  |  |  |  |  |  |  |  |  |  |
|  |  | Does the study concern unidimensionality or structural validity? |  |  |  |  |  |  |  |  |  |  |  |  |
| Statistical methods | 1 | For CTT: Was exploratory or confirmatory factor analysis performed? |  |  |  |  |  |  |  |  |  |  |  |  |
|  | 2 | For IRT/Rasch: does the chosen model fit to the research question? |  |  |  |  |  |  |  |  |  |  |  |  |
|  | 3 | Was the sample size included in the analysis A? |  |  |  |  |  |  |  |  |  |  |  |  |
|  |  | For FA |  |  |  |  |  |  |  |  |  |  |  |  |
|  |  | For Rasch/1PL models |  |  |  |  |  |  |  |  |  |  |  |  |
|  |  | For 2PL parametric IRT models OR Mokken scale analysis |  |  |  |  |  |  |  |  |  |  |  |  |
| Other | 4 | Were there any other important flaws in the design or statistical methods of the study? |  |  |  |  |  |  |  |  |  |  |  |  |
| CONSENSUS | | |  |  |  |  |  |  |  |  |  |  |  |  |
| Box 4. Internal consistency | | | | | | | | | | | | | | |
|  |  | Does the scale consist of effect indicators, i.e. is it based on a reflective model? | Y | Y | **Y** | Y | Y | **Y** | Y | Y | **Y** |  |  |  |
| Design requirements | 1 | Was an internal consistency statistic calculated for each unidimensional scale or subscale separately? | VG | VG | **VG** | VG | VG | **VG** | VG | VG | **VG** |  |  |  |
| Statistical methods | 2 | For continuous scores: Was Cronbach’s alpha or omega calculated? | VG | VG | **VG** | VG | VG | **VG** | VG | VG | **VG** |  |  |  |
|  | 3 | For dichotomous scores: Was Cronbach’s alpha or KR-20 calculated? | N/A |  | **N/A** | N/A |  | **N/A** | N/A |  | **N/A** |  |  |  |
|  | 4 | For IRT-based scores: Was standard error of the theta (SE (θ)) or reliability coefficient of estimated latent trait value (index of (subject or item) separation) calculated? | N/A |  | **N/A** | N/A |  | **N/A** | N/A |  | **N/A** |  |  |  |
| Other | 5 | Were there any other important flaws in the design or statistical methods of the study? | VG | VG | **VG** | VG | VG | **VG** | VG | VG | **VG** |  |  |  |
| CONSENSUS | | |  |  | **VG** |  |  | **VG** |  |  | **VG** |  |  |  |
| Box 5. Cross-cultural validity/Measurement invariance | | | | | | | | | | | | | | |
| Design requirements | 1 | Were the samples similar for relevant characteristics except for the group variable? |  |  |  |  |  |  |  |  |  |  |  |  |
| Statistical methods | 2 | Was an appropriate approach used to analyse the data? |  |  |  |  |  |  |  |  |  |  |  |  |
|  | 3 | Was the sample size included in the analysis A? |  |  |  |  |  |  |  |  |  |  |  |  |
|  |  | For Regression analyses or IRT/Rasch based analyses |  |  |  |  |  |  |  |  |  |  |  |  |
|  |  | For MGCFA* |  |  |  |  |  |  |  |  |  |  |  |  |
| Other | 4 | Were there any other important flaws in the design or statistical methods of the study? |  |  |  |  |  |  |  |  |  |  |  |  |
| CONSENSUS | | |  |  |  |  |  |  |  |  |  |  |  |  |
| Box 6. Reliability | | | | | | | | | | | | | | |
| Design requirements | 1 | Were patients stable in the interim period on the construct to be measured? | A | A | **A** | A | A | **A** | A | A | **A** |  |  |  |
|  | 2 | Was the time interval appropriate? | VG | D | **D** | VG | VG | **VG** | VG | VG | **VG** |  |  |  |
|  | 3 | Were the test conditions similar for the measurements? e.g. type of administration, environment, instructions | A | A | **A** | A | A | **A** | I | I | **I** |  |  |  |
| Statistical methods | 4 | For continuous scores: Was an intraclass correlation coefficient (ICC) calculated? | D | D | **D** | D | D | **D** | D | D | **D** |  |  |  |
|  | 5 | For dichotomous/nominal/ordinal scores: Was kappa calculated? | N/A |  | **N/A** | N/A |  | **N/A** | N/A |  | **N/A** |  |  |  |
|  | 6 | For ordinal scores: Was a weighted kappa calculated? | N/A |  | **N/A** | N/A |  | **N/A** | N/A |  | **N/A** |  |  |  |
|  | 7 | For ordinal scores: Was the weighting scheme described? e.g. linear, quadratic | N/A |  | **N/A** | N/A |  | **N/A** | N/A |  | **N/A** |  |  |  |
| Other | 8 | Were there any other important flaws in the design or statistical methods of the study? | VG | VG | **VG** | VG | VG | **VG** | VG | VG | **VG** |  |  |  |
| CONSENSUS | | |  |  | **D** |  |  | **D** |  |  | **I** |  |  |  |
| Box 7. Measurement error | | | | | | | | | | | | | | |
| Design requirements | 1 | Were patients stable in the interim period on the construct to be measured? |  |  |  |  |  |  |  |  |  |  |  |  |
|  | 2 | Was the time interval appropriate? |  |  |  |  |  |  |  |  |  |  |  |  |
|  | 3 | Were the test conditions similar for the measurements? e.g. type of administration, environment, instructions |  |  |  |  |  |  |  |  |  |  |  |  |
| Statistical methods | 4 | For continuous scores: Was the Standard Error of Measurement (SEM), Smallest Detectable Change (SDC) or Limits of Agreement (LoA) calculated? |  |  |  |  |  |  |  |  |  |  |  |  |
|  | 5 | For dichotomous/nominal/ordinal scores: Was the percentage (positive and negative) agreement calculated? |  |  |  |  |  |  |  |  |  |  |  |  |
| Other | 6 | Were there any other important flaws in the design or statistical methods of the study? |  |  |  |  |  |  |  |  |  |  |  |  |
| CONSENSUS | | |  |  |  |  |  |  |  |  |  |  |  |  |
| Box 8. Criterion validity | | | | | | | | | | | | | | |
| Statistical methods | 1 | For continuous scores: Were correlations, or the area under the receiver operating curve calculated? | VG | VG | **VG** | VG | VG | **VG** | N/A | VG | **VG** | VG | VG | **VG** |
|  | 2 | For dichotomous scores: Were sensitivity and specificity determined? | VG | VG | **VG** | VG | VG | **VG** | VG | VG | **VG** | VG | VG | **VG** |
| Other | 3 | Were there any other important flaws in the design or statistical methods of the study? | VG | VG | **VG** | VG | VG | **VG** | VG | VG | **VG** | VG | VG | **VG** |
| CONSENSUS | | |  |  | **VG** |  |  | **VG** |  |  | **VG** |  |  | **VG** |
| Box 9. Hypotheses testing for construct validity | | | | | | | | | | | | | | |
| 9a. Comparison with other outcome measurement instruments (convergent validity) | | | | | | | | | | | | | | |
| Design requirements | 1 | Is it clear what the comparator instrument(s) measure(s)? |  |  |  |  |  |  |  |  |  | VG | VG | **VG** |
|  | 2 | Were the measurement properties of the comparator instrument(s) sufficient? |  |  |  |  |  |  |  |  |  | VG | VG | **VG** |
| Statistical methods | 3 | Was the statistical method appropriate for the hypotheses to be tested? |  |  |  |  |  |  |  |  |  | VG | A | **VG** |
| Other | 4 | Were there any other important flaws in the design or statistical methods of the study? |  |  |  |  |  |  |  |  |  | VG | VG | **VG** |
| CONSENSUS | | |  |  |  |  |  |  |  |  |  |  |  | **VG** |
| 9b. Comparison between subgroups (discriminative or known-groups validity) | | | | | | | | | | | | | | |
| Design requirements | 5 | Was an A description provided of important characteristics of the subgroups? | VG | VG | **VG** | VG | VG | **VG** | D |  |  |  |  |  |
| Statistical methods | 6 | Was the statistical method appropriate for the hypotheses to be tested? | VG | VG | **VG** | VG | D | **VG** | VG |  |  |  |  |  |
| Other | 7 | Were there any other important flaws in the design or statistical methods of the study? | VG | VG | **VG** | VG | VG | **VG** | VG |  |  |  |  |  |
| CONSENSUS | | |  |  | **VG** |  |  | **VG** |  |  |  |  |  |  |
| Box 10. Responsiveness | | | | | | | | | | | | | | |
| 10a. Criterion approach (i.e. comparison to a gold standard) | | | | | | | | | | | | | | |
| Statistical methods | 1 | For continuous scores: Were correlations between change scores, or the area under the Receiver Operator Curve (ROC) curve calculated? |  |  |  |  |  |  |  |  |  |  |  |  |
|  | 2 | For dichotomous scales: Were sensitivity and specificity (changed versus not changed) determined? |  |  |  |  |  |  |  |  |  |  |  |  |
| Other | 3 | Were there any other important flaws in the design or statistical methods of the study? |  |  |  |  |  |  |  |  |  |  |  |  |
| CONSENSUS | | |  |  |  |  |  |  |  |  |  |  |  |  |
| 10b. Construct approach (i.e. hypotheses testing; comparison with other outcome measurement instruments) | | | | | | | | | | | | | | |
| Design requirements | 4 | Is it clear what the comparator instrument(s) measure(s)? |  |  |  |  |  |  |  |  |  |  |  |  |
|  | 5 | Were the measurement properties of the comparator instrument(s) sufficient? |  |  |  |  |  |  |  |  |  |  |  |  |
| Statistical methods | 6 | Was the statistical method appropriate for the hypotheses to be tested? |  |  |  |  |  |  |  |  |  |  |  |  |
| Other | 7 | Were there any other important flaws in the design or statistical methods of the study? |  |  |  |  |  |  |  |  |  |  |  |  |
| CONSENSUS | | |  |  |  |  |  |  |  |  |  |  |  |  |
| 10c. Construct approach (i.e. hypotheses testing: comparison between subgroups) | | | | | | | | | | | | | | |
| Design requirements | 8 | Was an A description provided of important characteristics of the subgroups? |  |  |  |  |  |  |  |  |  |  |  |  |
| Statistical methods | 9 | Was the statistical method appropriate for the hypotheses to be tested? |  |  |  |  |  |  |  |  |  |  |  |  |
| Other | 10 | Were there any other important flaws in the design or statistical methods of the study? |  |  |  |  |  |  |  |  |  |  |  |  |
| CONSENSUS | | |  |  |  |  |  |  |  |  |  |  |  |  |
| 10d. Construct approach (i.e. hypotheses testing: before and after intervention) | | | | | | | | | | | | | | |
| Design requirements | 11 | Was an A description provided of the intervention given? | D | VG | **D** | VG | VG | **VG** | D | VG | **VG** |  |  |  |
| Statistical methods | 12 | Was the statistical method appropriate for the hypotheses to be tested? | VG | VG | **VG** | VG |  | **VG** | VG | VG | **VG** |  |  |  |
| Other | 13 | Were there any other important flaws in the design or statistical methods of the study? | VG | VG | **VG** | VG |  | **VG** | VG | VG | **VG** |  |  |  |
| CONSENSUS | | |  |  | **D** |  |  | **VG** |  |  | **VG** |  |  |  |
| % | | | **65.79%** | | | **46.88%** | | | **40.63%** | | | **37.50%** | | |

A: adequate; C: consensus; D: doubtful; I: inadequate; N/A: not applicable; R1: Reviewer 1; R2: Reviewer 2; VG: very good; Y: yes

| UDI | | | | | | | | | | | | | | | | | |
| --- | --- | --- | --- | --- | --- | --- | --- | --- | --- | --- | --- | --- | --- | --- | --- | --- | --- |
| COSMIN Risk of Bias Checklist | | | Shumaker et al., 1994 | | | Harvey et al., 2001 | | | Hagen et al., 2002 | | | van der Vaart et al., 2003 | | | Artibani et al., 2006 | | |
|  | Item | Requirements | R1 | R2 | **C** | R1 | R2 | **C** | R1 | R2 | **C** | R1 | R2 | **C** | R1 | R2 | **C** |
| Box 1. PROM Development | | | | | | | | | | | | | | | | | |
| 1a. PROM design | | | | | | | | | | | | | | | | | |
|  | 1 | Is a clear description provided of the construct to be measured? | VG | VG | **VG** |  |  |  |  |  |  |  |  |  |  |  |  |
|  | 2 | Is the origin of the construct clear: was a theory, conceptual framework or disease model used or clear rationale provided to define the construct to be measured? | VG | VG | **VG** |  |  |  |  |  |  |  |  |  |  |  |  |
|  | 3 | Is a clear description provided of the target population for which the PROM was developed? | VG | VG | **VG** |  |  |  |  |  |  |  |  |  |  |  |  |
|  | 4 | Is a clear description provided of the context of use | VG | VG | **VG** |  |  |  |  |  |  |  |  |  |  |  |  |
|  | 5 | Was the PROM development study performed in a sample representing the target population for which the PROM was developed? | VG | VG | **VG** |  |  |  |  |  |  |  |  |  |  |  |  |
|  | 6 | Was an appropriate qualitative data collection method used to identify relevant items for a new PROM? | VG | VG | **VG** |  |  |  |  |  |  |  |  |  |  |  |  |
|  | 7 | Were skilled group moderators/interviewers used? | D | D | **D** |  |  |  |  |  |  |  |  |  |  |  |  |
|  | 8 | Were the group meetings or interviews based on an appropriate topic or interview guide? | A | A | **A** |  |  |  |  |  |  |  |  |  |  |  |  |
|  | 9 | Were the group meetings or interviews recorded and transcribed verbatim? | D | D | **D** |  |  |  |  |  |  |  |  |  |  |  |  |
|  | 10 | Was an appropriate approach used to analyse the data? | D | D | **D** |  |  |  |  |  |  |  |  |  |  |  |  |
|  | 11 | Was at least part of the data coded independently? | D | D | **D** |  |  |  |  |  |  |  |  |  |  |  |  |
|  | 12 | Was data collection continued until saturation was reached? | D | D | **D** |  |  |  |  |  |  |  |  |  |  |  |  |
|  | 13 | For quantitative studies (surveys): was the sample size appropriate? | N/A |  | **N/A** |  |  |  |  |  |  |  |  |  |  |  |  |
| 1b. Cognitive interview study or pilot test | | | | | | | | | | | | | | | | | |
|  | 14 | Was a cognitive interview study or other pilot test conducted? | I | I | **I** |  |  |  |  |  |  |  |  |  |  | VG |  |
| General design requirements | 15 | Was the cognitive interview study or other pilot test performed in a sample representing the target population? |  |  |  |  |  |  |  |  |  |  |  |  |  | VG |  |
| Comprehensibility | 16 | Were patients asked about the comprehensibility of the PROM? |  |  |  |  |  |  |  |  |  |  |  |  |  | VG |  |
|  | 17 | Were all items tested in their fIl form? |  |  |  |  |  |  |  |  |  |  |  |  |  | VG |  |
|  | 18 | Was an appropriate qualitative method used to assess the comprehensibility of the PROM instructions, items, response options, and recall period? |  |  |  |  |  |  |  |  |  |  |  |  |  | A |  |
|  | 19 | Was each item tested in an appropriate number of patients? |  |  |  |  |  |  |  |  |  |  |  |  |  |  |  |
|  |  | For qualitative studies |  |  |  |  |  |  |  |  |  |  |  |  |  | VG |  |
|  |  | For quantitative (survey) studies |  |  |  |  |  |  |  |  |  |  |  |  |  |  |  |
|  | 20 | Were skilled interviewers used? |  |  |  |  |  |  |  |  |  |  |  |  |  | D |  |
|  | 21 | Were the interviews based on an appropriate interview guide? |  |  |  |  |  |  |  |  |  |  |  |  |  | D |  |
|  | 22 | Were the interviews recorded and transcribed verbatim? |  |  |  |  |  |  |  |  |  |  |  |  |  | D |  |
|  | 23 | Was an appropriate approach used to analyse the data? |  |  |  |  |  |  |  |  |  |  |  |  |  | D |  |
|  | 24 | Were at least two researchers involved in the analysis? |  |  |  |  |  |  |  |  |  |  |  |  |  | D |  |
|  | 25 | Were problems regarding the comprehensibility of the PROM instructions, items, response options, and recall period appropriately addressed by adapting the PROM? |  |  |  |  |  |  |  |  |  |  |  |  |  | I |  |
| Comprehensiveness | 26 | Were patients asked about the comprehensiveness of the PROM? |  |  |  |  |  |  |  |  |  |  |  |  |  | D |  |
|  | 27 | Was the final set of items tested? |  |  |  |  |  |  |  |  |  |  |  |  |  |  |  |
|  | 28 | Was an appropriate method used for assessing the comprehensiveness of the PROM? |  |  |  |  |  |  |  |  |  |  |  |  |  |  |  |
|  | 29 | Was each item tested in an appropriate number of patients? |  |  |  |  |  |  |  |  |  |  |  |  |  |  |  |
|  |  | For qualitative studies |  |  |  |  |  |  |  |  |  |  |  |  |  |  |  |
|  |  | For quantitative (survey) studies |  |  |  |  |  |  |  |  |  |  |  |  |  |  |  |
|  | 30 | Were skilled interviewers used? |  |  |  |  |  |  |  |  |  |  |  |  |  |  |  |
|  | 31 | Were the interviews based on an appropriate interview guide? |  |  |  |  |  |  |  |  |  |  |  |  |  |  |  |
|  | 32 | Were the interviews recorded and transcribed verbatim? |  |  |  |  |  |  |  |  |  |  |  |  |  |  |  |
|  | 33 | Was an appropriate approach used to analyse the data? |  |  |  |  |  |  |  |  |  |  |  |  |  |  |  |
|  | 34 | Were at least two researchers involved in the analysis? |  |  |  |  |  |  |  |  |  |  |  |  |  |  |  |
|  | 35 | Were problems regarding the comprehensiveness of the PROM appropriately addressed by adapting the PROM? |  |  |  |  |  |  |  |  |  |  |  |  |  |  |  |
| CONSENSUS | | |  |  | **I** |  |  |  |  |  |  |  |  |  |  |  |  |
| Box 2. Content validity | | | | | | | | | | | | | | | | | |
| 2a. Asking patients about relevance | | | | | | | | | | | | | | | | | |
| Design requirements | 1 | Was an appropriate method used to ask patients whether each item is relevant for their experience with the condition? |  |  |  |  |  |  |  |  |  |  |  |  |  |  |  |
|  | 2 | Was each item tested in an appropriate number of patients? |  |  |  |  |  |  |  |  |  |  |  |  |  |  |  |
|  |  | For qualitative studies |  |  |  |  |  |  |  |  |  |  |  |  |  |  |  |
|  |  | For quantitative (survey) studies |  |  |  |  |  |  |  |  |  |  |  |  |  |  |  |
|  | 3 | Were skilled group moderators/interviewers used? |  |  |  |  |  |  |  |  |  |  |  |  |  |  |  |
|  | 4 | Were the group meetings or interviews based on an appropriate topic or interview guide? |  |  |  |  |  |  |  |  |  |  |  |  |  |  |  |
|  | 5 | Were the group meetings or interviews recorded and transcribed verbatim? |  |  |  |  |  |  |  |  |  |  |  |  |  |  |  |
| Analyses | 6 | Was an appropriate approach used to analyse the data? |  |  |  |  |  |  |  |  |  |  |  |  |  |  |  |
|  | 7 | Were at least two researchers involved in the analysis? |  |  |  |  |  |  |  |  |  |  |  |  |  |  |  |
| 2b. Asking patients about comprehensiveness | | | | | | | | | | | | | | | | | |
| Design requirements | 8 | Was an appropriate method used for assessing the comprehensiveness of the PROM? |  |  |  |  |  |  |  |  |  |  |  |  |  |  |  |
|  | 9 | Was each item tested in an appropriate number of patients? |  |  |  |  |  |  |  |  |  |  |  |  |  |  |  |
|  |  | For qualitative studies |  |  |  |  |  |  |  |  |  |  |  |  |  |  |  |
|  |  | For quantitative (survey) studies |  |  |  |  |  |  |  |  |  |  |  |  |  |  |  |
|  | 10 | Were skilled group moderators/interviewers used? |  |  |  |  |  |  |  |  |  |  |  |  |  |  |  |
|  | 11 | Were the group meetings or interviews based on an appropriate topic or interview guide? |  |  |  |  |  |  |  |  |  |  |  |  |  |  |  |
|  | 12 | Were the group meetings or interviews recorded and transcribed verbatim? |  |  |  |  |  |  |  |  |  |  |  |  |  |  |  |
| Analyses | 13 | Was an appropriate approach used to analyse the data? |  |  |  |  |  |  |  |  |  |  |  |  |  |  |  |
|  | 14 | Were at least two researchers involved in the analysis? |  |  |  |  |  |  |  |  |  |  |  |  |  |  |  |
| 2c. Asking patients about comprehensibility | | | | | | | | | | | | | | | | | |
| Design requirements | 15 | Was an appropriate qualitative method used for assessing the comprehensibility of the PROM instructions, items, response options, and recall period? |  |  |  |  |  |  |  |  |  |  |  |  | A |  | **A** |
|  | 16 | Was each item tested in an appropriate number of patients? |  |  |  |  |  |  |  |  |  |  |  |  |  |  |  |
|  |  | For qualitative studies |  |  |  |  |  |  |  |  |  |  |  |  | VG |  | **VG** |
|  |  | For quantitative (survey) studies |  |  |  |  |  |  |  |  |  |  |  |  |  |  |  |
|  | 17 | Were skilled group moderators/interviewers used? |  |  |  |  |  |  |  |  |  |  |  |  | D |  | **D** |
|  | 18 | Were the group meetings or interviews based on an appropriate topic or interview guide? |  |  |  |  |  |  |  |  |  |  |  |  | D |  | **D** |
|  | 19 | Were the group meetings or interviews recorded and transcribed verbatim? |  |  |  |  |  |  |  |  |  |  |  |  | D |  | **D** |
| Analyses | 20 | Was an appropriate approach used to analyse the data? |  |  |  |  |  |  |  |  |  |  |  |  | D |  | **D** |
|  | 21 | Were at least two researchers involved in the analysis? |  |  |  |  |  |  |  |  |  |  |  |  | D |  | **D** |
| 2d. Asking professionals about relevance | | | | | | | | | | | | | | | | | |
| Design requirements | 22 | Was an appropriate method used to ask professionals whether each item is relevant for the construct of interest? |  |  |  |  |  |  |  |  |  |  |  |  |  |  |  |
|  | 23 | Were professionals from all relevant disciplines included? |  |  |  |  |  |  |  |  |  |  |  |  |  |  |  |
|  | 24 | Was each item tested in an appropriate number of professionals? |  |  |  |  |  |  |  |  |  |  |  |  |  |  |  |
|  |  | For qualitative studies |  |  |  |  |  |  |  |  |  |  |  |  |  |  |  |
|  |  | For quantitative (survey) studies |  |  |  |  |  |  |  |  |  |  |  |  |  |  |  |
| Analyses | 25 | Was an appropriate approach used to analyse the data? |  |  |  |  |  |  |  |  |  |  |  |  |  |  |  |
|  | 26 | Were at least two researchers involved in the analysis? |  |  |  |  |  |  |  |  |  |  |  |  |  |  |  |
| 2e. Asking professionals about comprehensiveness | | | | | | | | | | | | | | | | | |
| Design requirements | 27 | Was an appropriate method used for assessing the comprehensiveness of the PROM? |  |  |  |  |  |  |  |  |  |  |  |  |  |  |  |
|  | 28 | Were professionals from all relevant disciplines included? |  |  |  |  |  |  |  |  |  |  |  |  |  |  |  |
|  | 29 | Was each item tested in an appropriate number of professionals? |  |  |  |  |  |  |  |  |  |  |  |  |  |  |  |
|  |  | For qualitative studies |  |  |  |  |  |  |  |  |  |  |  |  |  |  |  |
|  |  | For quantitative (survey) studies |  |  |  |  |  |  |  |  |  |  |  |  |  |  |  |
| Analyses | 30 | Was an appropriate approach used to analyse the data? |  |  |  |  |  |  |  |  |  |  |  |  |  |  |  |
|  | 31 | Were at least two researchers involved in the analysis? |  |  |  |  |  |  |  |  |  |  |  |  |  |  |  |
| CONSENSUS | | |  |  |  |  |  |  |  |  |  |  |  |  |  |  | **D** |
| Box 3. Structural validity | | | | | | | | | | | | | | | | | |
|  |  | Does the scale consist of effect indicators, i.e. is it based on a reflective model? | Y | Y | **Y** |  |  |  |  |  |  |  | Y | **Y** | Y | Y | **Y** |
|  |  | Does the study concern unidimensionality or structural validity? | SV | SV | **SV** |  |  |  |  |  |  |  | SV | **SV** | SV | SV | **SV** |
| Statistical methods | 1 | For CTT: Was exploratory or confirmatory factor analysis performed? | A | A | **A** |  |  |  |  |  |  |  | A | **A** | VG | A | **VG** |
|  | 2 | For IRT/Rasch: does the chosen model fit to the research question? | N/A | N/A | **N/A** |  |  |  |  |  |  |  | N/A | **N/A** |  | N/A | **N/A** |
|  | 3 | Was the sample size included in the analysis A? |  |  |  |  |  |  |  |  |  |  |  |  |  |  |  |
|  |  | For FA | VG | VG | **VG** |  |  |  |  |  |  |  | VG | **VG** | A | I | **A** |
|  |  | For Rasch/1PL models |  |  |  |  |  |  |  |  |  |  |  |  |  |  |  |
|  |  | For 2PL parametric IRT models OR Mokken scale analysis |  |  |  |  |  |  |  |  |  |  |  |  |  |  |  |
| Other | 4 | Were there any other important flaws in the design or statistical methods of the study? | VG | VG | **VG** |  |  |  |  |  |  |  | VG | **VG** | VG | VG | **VG** |
| CONSENSUS | | |  |  | **A** |  |  |  |  |  |  |  |  | **A** |  |  | **A** |
| Box 4. Internal consistency | | | | | | | | | | | | | | | | | |
|  |  | Does the scale consist of effect indicators, i.e. is it based on a reflective model? | Y | Y | **Y** | Y | Y | **Y** |  |  |  | Y | Y |  | Y | Y |  |
| Design requirements | 1 | Was an internal consistency statistic calculated for each unidimensional scale or subscale separately? | VG | VG | **VG** | D | I | **I** |  |  |  | VG | VG | **VG** | VG | VG | **VG** |
| Statistical methods | 2 | For continuous scores: Was Cronbach’s alpha or omega calculated? | VG | VG | **VG** | VG | VG | **VG** |  |  |  | VG | VG | **VG** | VG | VG | **VG** |
|  | 3 | For dichotomous scores: Was Cronbach’s alpha or KR-20 calculated? | N/A | N/A | **N/A** |  | N/A | **N/A** |  |  |  |  | N/A | **N/A** |  | N/A | **N/A** |
|  | 4 | For IRT-based scores: Was standard error of the theta (SE (θ)) or reliability coefficient of estimated latent trait value (index of (subject or item) separation) calculated? | N/A | N/A | **N/A** |  | N/A | **N/A** |  |  |  |  | N/A | **N/A** |  | N/A | **N/A** |
| Other | 5 | Were there any other important flaws in the design or statistical methods of the study? | VG | VG | **VG** | VG | VG | **VG** |  |  |  | VG | VG | **VG** | VG | VG | **VG** |
| CONSENSUS | | |  |  | **VG** |  |  | **I** |  |  |  |  |  | **VG** |  |  | **VG** |
| Box 5. Cross-cultural validity/Measurement invariance | | | | | | | | | | | | | | | | | |
| Design requirements | 1 | Were the samples similar for relevant characteristics except for the group variable? |  |  |  |  |  |  |  |  |  |  |  |  |  |  |  |
| Statistical methods | 2 | Was an appropriate approach used to analyse the data? |  |  |  |  |  |  |  |  |  |  |  |  |  |  |  |
|  | 3 | Was the sample size included in the analysis A? |  |  |  |  |  |  |  |  |  |  |  |  |  |  |  |
|  |  | For Regression analyses or IRT/Rasch based analyses |  |  |  |  |  |  |  |  |  |  |  |  |  |  |  |
|  |  | For MGCFA* |  |  |  |  |  |  |  |  |  |  |  |  |  |  |  |
| Other | 4 | Were there any other important flaws in the design or statistical methods of the study? |  |  |  |  |  |  |  |  |  |  |  |  |  |  |  |
| CONSENSUS | | |  |  |  |  |  |  |  |  |  |  |  |  |  |  |  |
| Box 6. Reliability | | | | | | | | | | | | | | | | | |
| Design requirements | 1 | Were patients stable in the interim period on the construct to be measured? |  |  |  |  |  |  | A | A | **A** |  |  |  |  | A | **A** |
|  | 2 | Was the time interval appropriate? |  |  |  |  |  |  | VG | D | **VG** |  |  |  | VG | VG | **VG** |
|  | 3 | Were the test conditions similar for the measurements? e.g. type of administration, environment, instructions |  |  |  |  |  |  | D | A | **A** |  |  |  | A | A | **A** |
| Statistical methods | 4 | For continuous scores: Was an intraclass correlation coefficient (ICC) calculated? |  |  |  |  |  |  |  | I | **N/A** |  |  |  |  | D | **D** |
|  | 5 | For dichotomous/nominal/ordinal scores: Was kappa calculated? |  |  |  |  |  |  |  | N/A | **N/A** |  |  |  |  | N/A | **N/A** |
|  | 6 | For ordinal scores: Was a weighted kappa calculated? |  |  |  |  |  |  | VG | N/A | **VG** |  |  |  |  | N/A | **N/A** |
|  | 7 | For ordinal scores: Was the weighting scheme described? e.g. linear, quadratic |  |  |  |  |  |  |  | N/A | **A** |  |  |  |  | N/A | **N/A** |
| Other | 8 | Were there any other important flaws in the design or statistical methods of the study? |  |  |  |  |  |  | VG | VG | **VG** |  |  |  |  | VG | **VG** |
| CONSENSUS | | |  |  |  |  |  |  |  |  | **A** |  |  |  |  |  | **D** |
| Box 7. Measurement error | | | | | | | | | | | | | | | | | |
| Design requirements | 1 | Were patients stable in the interim period on the construct to be measured? |  |  |  |  |  |  |  |  |  |  |  |  |  |  |  |
|  | 2 | Was the time interval appropriate? |  |  |  |  |  |  |  |  |  |  |  |  |  |  |  |
|  | 3 | Were the test conditions similar for the measurements? e.g. type of administration, environment, instructions |  |  |  |  |  |  |  |  |  |  |  |  |  |  |  |
| Statistical methods | 4 | For continuous scores: Was the Standard Error of Measurement (SEM), Smallest Detectable Change (SDC) or Limits of Agreement (LoA) calculated? |  |  |  |  |  |  |  |  |  |  |  |  |  |  |  |
|  | 5 | For dichotomous/nominal/ordinal scores: Was the percentage (positive and negative) agreement calculated? |  |  |  |  |  |  |  |  |  |  |  |  |  |  |  |
| Other | 6 | Were there any other important flaws in the design or statistical methods of the study? |  |  |  |  |  |  |  |  |  |  |  |  |  |  |  |
| CONSENSUS | | |  |  |  |  |  |  |  |  |  |  |  |  |  |  |  |
| Box 8. Criterion validity | | | | | | | | | | | | | | | | | |
| Statistical methods | 1 | For continuous scores: Were correlations, or the area under the receiver operating curve calculated? | VG | VG |  | VG | VG | **VG** |  | VG |  |  | VG |  |  | I |  |
|  | 2 | For dichotomous scores: Were sensitivity and specificity determined? | N/A | N/A |  |  | N/A | **N/A** |  | N/A |  |  | N/A |  |  | N/A |  |
| Other | 3 | Were there any other important flaws in the design or statistical methods of the study? | VG | VG |  | VG | VG | **VG** |  | VG |  |  | VG |  |  | VG |  |
| CONSENSUS | | |  |  |  |  |  | **VG** |  |  |  |  |  |  |  |  |  |
| Box 9. Hypotheses testing for construct validity | | | | | | | | | | | | | | | | | |
| 9a. Comparison with other outcome measurement instruments (convergent validity) | | | | | | | | | | | | | | | | | |
| Design requirements | 1 | Is it clear what the comparator instrument(s) measure(s)? |  | VG | **VG** |  |  |  |  |  |  | VG | VG | **VG** |  |  | **VG** |
|  | 2 | Were the measurement properties of the comparator instrument(s) sufficient? |  | A | **A** |  |  |  |  |  |  | VG | VG | **VG** | I |  | **I** |
| Statistical methods | 3 | Was the statistical method appropriate for the hypotheses to be tested? |  | D | **D** |  |  |  |  |  |  | VG | A | **A** | A |  | **D** |
| Other | 4 | Were there any other important flaws in the design or statistical methods of the study? |  | VG | **VG** |  |  |  |  |  |  |  | VG | **VG** |  |  | **VG** |
| CONSENSUS | | |  |  | **D** |  |  |  |  |  |  |  |  | **A** |  |  | **I** |
| 9b. Comparison between subgroups (discriminative or known-groups validity) | | | | | | | | | | | | | | | | | |
| Design requirements | 5 | Was an A description provided of important characteristics of the subgroups? |  | VG |  | VG |  |  |  |  |  | VG |  | **A** | VG | VG | **VG** |
| Statistical methods | 6 | Was the statistical method appropriate for the hypotheses to be tested? |  | D |  | A |  |  |  |  |  | A |  | **A** | VG | VG | **VG** |
| Other | 7 | Were there any other important flaws in the design or statistical methods of the study? |  | VG |  | VG |  |  |  |  |  | VG |  | **VG** | VG | VG | **VG** |
| CONSENSUS | | |  |  |  |  |  |  |  |  |  |  |  | **A** |  |  | **VG** |
| Box 10. Responsiveness | | | | | | | | | | | | | | | | | |
| 10a. Criterion approach (i.e. comparison to a gold standard) | | | | | | | | | | | | | | | | | |
| Statistical methods | 1 | For continuous scores: Were correlations between change scores, or the area under the Receiver Operator Curve (ROC) curve calculated? | VG | VG | **VG** |  |  |  |  |  |  |  | VG |  |  |  |  |
|  | 2 | For dichotomous scales: Were sensitivity and specificity (changed versus not changed) determined? | N/A | N/A | **N/A** |  |  |  |  |  |  |  | N/A |  |  |  |  |
| Other | 3 | Were there any other important flaws in the design or statistical methods of the study? | VG | VG | **VG** |  |  |  |  |  |  |  | VG |  |  |  |  |
| CONSENSUS | | |  |  | **VG** |  |  |  |  |  |  |  |  |  |  |  |  |
| 10b. Construct approach (i.e. hypotheses testing; comparison with other outcome measurement instruments) | | | | | | | | | | | | | | | | | |
| Design requirements | 4 | Is it clear what the comparator instrument(s) measure(s)? |  |  |  | VG | VG | **VG** |  |  |  |  |  |  |  |  |  |
|  | 5 | Were the measurement properties of the comparator instrument(s) sufficient? |  |  |  | I | I | **I** |  |  |  |  |  |  |  |  |  |
| Statistical methods | 6 | Was the statistical method appropriate for the hypotheses to be tested? |  |  |  | VG | A | **A** |  |  |  |  |  |  |  |  |  |
| Other | 7 | Were there any other important flaws in the design or statistical methods of the study? |  |  |  | VG | VG | **VG** |  |  |  |  |  |  |  |  |  |
| CONSENSUS | | |  |  |  |  |  | **I** |  |  |  |  |  |  |  |  |  |
| 10c. Construct approach (i.e. hypotheses testing: comparison between subgroups) | | | | | | | | | | | | | | | | | |
| Design requirements | 8 | Was an A description provided of important characteristics of the subgroups? |  |  |  |  |  |  | VG |  | **VG** |  |  |  |  |  |  |
| Statistical methods | 9 | Was the statistical method appropriate for the hypotheses to be tested? |  |  |  |  |  |  | A |  | **A** |  |  |  | VG |  |  |
| Other | 10 | Were there any other important flaws in the design or statistical methods of the study? |  |  |  |  |  |  | VG |  | **VG** |  |  |  |  |  |  |
| CONSENSUS | | |  |  |  |  |  |  |  |  | **A** |  |  |  |  |  |  |
| 10d. Construct approach (i.e. hypotheses testing: before and after intervention) | | | | | | | | | | | | | | | | | |
| Design requirements | 11 | Was an A description provided of the intervention given? |  |  |  |  | VG | **VG** |  | VG | **VG** |  |  |  |  | D | **D** |
| Statistical methods | 12 | Was the statistical method appropriate for the hypotheses to be tested? |  |  |  |  | VG | **VG** |  | D | **A** |  |  |  |  | VG | **VG** |
| Other | 13 | Were there any other important flaws in the design or statistical methods of the study? |  |  |  |  | VG | **VG** |  | VG | **VG** |  |  |  |  | I | **D** |
| CONSENSUS | | |  |  |  |  |  | **VG** |  |  | **A** |  |  |  |  |  | **D** |
| % | | | **79.49%** | | | **42.11%** | | | **11.76%** | | | **24.00%** | | | **23.08%** | | |

A: adequate; C: consensus; D: doubtful; I: inadequate; N/A: not applicable; R1: Reviewer 1; R2: Reviewer 2; SV: structural validity; VG: very good; Y: yes

| U-UDI | | | | | | | | | | | |
| --- | --- | --- | --- | --- | --- | --- | --- | --- | --- | --- | --- |
| COSMIN Risk of Bias Checklist | | | Brown et al., 1999 | | | Lubek et al., 1999 | | | Borello France et al., 2008 | | |
|  | Item | Requirements | R1 | R2 | **C** | R1 | R2 | **C** | R1 | R2 | **C** |
| Box 1. PROM Development | | | | | | | | | | | |
| 1a. PROM design | | | | | | | | | | | |
|  | 1 | Is a clear description provided of the construct to be measured? | VG | VG | **VG** |  |  |  |  |  |  |
|  | 2 | Is the origin of the construct clear: was a theory, conceptual framework or disease model used or clear rationale provided to define the construct to be measured? | VG | VG | **VG** |  |  |  |  |  |  |
|  | 3 | Is a clear description provided of the target population for which the PROM was developed? | VG | VG | **VG** |  |  |  |  |  |  |
|  | 4 | Is a clear description provided of the context of use | VG | VG | **VG** |  |  |  |  |  |  |
|  | 5 | Was the PROM development study performed in a sample representing the target population for which the PROM was developed? | VG | VG | **VG** |  |  |  |  |  |  |
|  | 6 | Was an appropriate qualitative data collection method used to identify relevant items for a new PROM? | VG | VG | **VG** |  |  |  |  |  |  |
|  | 7 | Were skilled group moderators/interviewers used? | D | D | **D** |  |  |  |  |  |  |
|  | 8 | Were the group meetings or interviews based on an appropriate topic or interview guide? | A | D | **D** |  |  |  |  |  |  |
|  | 9 | Were the group meetings or interviews recorded and transcribed verbatim? | D | D | **D** |  |  |  |  |  |  |
|  | 10 | Was an appropriate approach used to analyse the data? | D | D | **D** |  |  |  |  |  |  |
|  | 11 | Was at least part of the data coded independently? | D | D | **D** |  |  |  |  |  |  |
|  | 12 | Was data collection continued until saturation was reached? | D | A | **D** |  |  |  |  |  |  |
|  | 13 | For quantitative studies (surveys): was the sample size appropriate? | N/A | N/A | **N/A** |  |  |  |  |  |  |
| 1b. Cognitive interview study or pilot test | | | | | | | | | | | |
|  | 14 | Was a cognitive interview study or other pilot test conducted? |  | VG | **V** |  |  |  |  |  |  |
| General design requirements | 15 | Was the cognitive interview study or other pilot test performed in a sample representing the target population? |  | VG | **VG** |  |  |  |  |  |  |
| Comprehensibility | 16 | Were patients asked about the comprehensibility of the PROM? |  | I | **I** |  |  |  |  |  |  |
|  | 17 | Were all items tested in their final form? |  |  |  |  |  |  |  |  |  |
|  | 18 | Was an appropriate qualitative method used to assess the comprehensibility of the PROM instructions, items, response options, and recall period? |  |  |  |  |  |  |  |  |  |
|  | 19 | Was each item tested in an appropriate number of patients? |  |  |  |  |  |  |  |  |  |
|  |  | For qualitative studies |  |  |  |  |  |  |  |  |  |
|  |  | For quantitative (survey) studies |  |  |  |  |  |  |  |  |  |
|  | 20 | Were skilled interviewers used? |  |  |  |  |  |  |  |  |  |
|  | 21 | Were the interviews based on an appropriate interview guide? |  |  |  |  |  |  |  |  |  |
|  | 22 | Were the interviews recorded and transcribed verbatim? |  |  |  |  |  |  |  |  |  |
|  | 23 | Was an appropriate approach used to analyse the data? |  |  |  |  |  |  |  |  |  |
|  | 24 | Were at least two researchers involved in the analysis? |  |  |  |  |  |  |  |  |  |
|  | 25 | Were problems regarding the comprehensibility of the PROM instructions, items, response options, and recall period appropriately addressed by adapting the PROM? |  |  |  |  |  |  |  |  |  |
| Comprehensiveness | 26 | Were patients asked about the comprehensiveness of the PROM? |  | D | **D** |  |  |  |  |  |  |
|  | 27 | Was the final set of items tested? |  |  |  |  |  |  |  |  |  |
|  | 28 | Was an appropriate method used for assessing the comprehensiveness of the PROM? |  |  |  |  |  |  |  |  |  |
|  | 29 | Was each item tested in an appropriate number of patients? |  |  |  |  |  |  |  |  |  |
|  |  | For qualitative studies |  |  |  |  |  |  |  |  |  |
|  |  | For quantitative (survey) studies |  |  |  |  |  |  |  |  |  |
|  | 30 | Were skilled interviewers used? |  |  |  |  |  |  |  |  |  |
|  | 31 | Were the interviews based on an appropriate interview guide? |  |  |  |  |  |  |  |  |  |
|  | 32 | Were the interviews recorded and transcribed verbatim? |  |  |  |  |  |  |  |  |  |
|  | 33 | Was an appropriate approach used to analyse the data? |  |  |  |  |  |  |  |  |  |
|  | 34 | Were at least two researchers involved in the analysis? |  |  |  |  |  |  |  |  |  |
|  | 35 | Were problems regarding the comprehensiveness of the PROM appropriately addressed by adapting the PROM? |  |  |  |  |  |  |  |  |  |
| CONSENSUS | | |  |  | **I** |  |  |  |  |  |  |
| Box 2. Content validity | | | | | | | | | | | |
| 2a. Asking patients about relevance | | | | | | | | | | | |
| Design requirements | 1 | Was an appropriate method used to ask patients whether each item is relevant for their experience with the condition? |  |  |  |  |  |  |  |  |  |
|  | 2 | Was each item tested in an appropriate number of patients? |  |  |  |  |  |  |  |  |  |
|  |  | For qualitative studies |  |  |  |  |  |  |  |  |  |
|  |  | For quantitative (survey) studies |  |  |  |  |  |  |  |  |  |
|  | 3 | Were skilled group moderators/interviewers used? |  |  |  |  |  |  |  |  |  |
|  | 4 | Were the group meetings or interviews based on an appropriate topic or interview guide? |  |  |  |  |  |  |  |  |  |
|  | 5 | Were the group meetings or interviews recorded and transcribed verbatim? |  |  |  |  |  |  |  |  |  |
| Analyses | 6 | Was an appropriate approach used to analyse the data? |  |  |  |  |  |  |  |  |  |
|  | 7 | Were at least two researchers involved in the analysis? |  |  |  |  |  |  |  |  |  |
| 2b. Asking patients about comprehensiveness | | | | | | | | | | | |
| Design requirements | 8 | Was an appropriate method used for assessing the comprehensiveness of the PROM? |  |  |  |  |  |  |  |  |  |
|  | 9 | Was each item tested in an appropriate number of patients? |  |  |  |  |  |  |  |  |  |
|  |  | For qualitative studies |  |  |  |  |  |  |  |  |  |
|  |  | For quantitative (survey) studies |  |  |  |  |  |  |  |  |  |
|  | 10 | Were skilled group moderators/interviewers used? |  |  |  |  |  |  |  |  |  |
|  | 11 | Were the group meetings or interviews based on an appropriate topic or interview guide? |  |  |  |  |  |  |  |  |  |
|  | 12 | Were the group meetings or interviews recorded and transcribed verbatim? |  |  |  |  |  |  |  |  |  |
| Analyses | 13 | Was an appropriate approach used to analyse the data? |  |  |  |  |  |  |  |  |  |
|  | 14 | Were at least two researchers involved in the analysis? |  |  |  |  |  |  |  |  |  |
| 2c. Asking patients about comprehensibility | | | | | | | | | | | |
| Design requirements | 15 | Was an appropriate qualitative method used for assessing the comprehensibility of the PROM instructions, items, response options, and recall period? |  |  |  |  |  |  |  |  |  |
|  | 16 | Was each item tested in an appropriate number of patients? |  |  |  |  |  |  |  |  |  |
|  |  | For qualitative studies |  |  |  |  |  |  |  |  |  |
|  |  | For quantitative (survey) studies |  |  |  |  |  |  |  |  |  |
|  | 17 | Were skilled group moderators/interviewers used? |  |  |  |  |  |  |  |  |  |
|  | 18 | Were the group meetings or interviews based on an appropriate topic or interview guide? |  |  |  |  |  |  |  |  |  |
|  | 19 | Were the group meetings or interviews recorded and transcribed verbatim? |  |  |  |  |  |  |  |  |  |
| Analyses | 20 | Was an appropriate approach used to analyse the data? |  |  |  |  |  |  |  |  |  |
|  | 21 | Were at least two researchers involved in the analysis? |  |  |  |  |  |  |  |  |  |
| 2d. Asking professionals about relevance | | | | | | | | | | | |
| Design requirements | 22 | Was an appropriate method used to ask professionals whether each item is relevant for the construct of interest? |  |  |  |  |  |  |  |  |  |
|  | 23 | Were professionals from all relevant disciplines included? |  |  |  |  |  |  |  |  |  |
|  | 24 | Was each item tested in an appropriate number of professionals? |  |  |  |  |  |  |  |  |  |
|  |  | For qualitative studies |  |  |  |  |  |  |  |  |  |
|  |  | For quantitative (survey) studies |  |  |  |  |  |  |  |  |  |
| Analyses | 25 | Was an appropriate approach used to analyse the data? |  |  |  |  |  |  |  |  |  |
|  | 26 | Were at least two researchers involved in the analysis? |  |  |  |  |  |  |  |  |  |
|  | | | | | | | | | | | |
| Design requirements | 27 | Was an appropriate method used for assessing the comprehensiveness of the PROM? |  |  |  |  |  |  |  |  |  |
|  | 28 | Were professionals from all relevant disciplines included? |  |  |  |  |  |  |  |  |  |
|  | 29 | Was each item tested in an appropriate number of professionals? |  |  |  |  |  |  |  |  |  |
|  |  | For qualitative studies |  |  |  |  |  |  |  |  |  |
|  |  | For quantitative (survey) studies |  |  |  |  |  |  |  |  |  |
| Analyses | 30 | Was an appropriate approach used to analyse the data? |  |  |  |  |  |  |  |  |  |
|  | 31 | Were at least two researchers involved in the analysis? |  |  |  |  |  |  |  |  |  |
| CONSENSUS |  |  |  |  |  |  |  |  |  |  |  |
| Box 3. Structural validity | | | | | | | | | | | |
|  |  | Does the scale consist of effect indicators, i.e. is it based on a reflective model? | Y | Y | **Y** |  |  |  |  |  |  |
|  |  | Does the study concern unidimensionality or structural validity? | U | SV | **SV** |  |  |  |  |  |  |
| Statistical methods | 1 | For CTT: Was exploratory or confirmatory factor analysis performed? | A | A | **A** |  |  |  |  |  |  |
|  | 2 | For IRT/Rasch: does the chosen model fit to the research question? | N/A |  | **N/A** |  |  |  |  |  |  |
|  | 3 | Was the sample size included in the analysis A? |  |  |  |  |  |  |  |  |  |
|  |  | For FA | A | D | **A** |  |  |  |  |  |  |
|  |  | For Rasch/1PL models |  |  |  |  |  |  |  |  |  |
|  |  | For 2PL parametric IRT models OR Mokken scale analysis |  |  |  |  |  |  |  |  |  |
| Other | 4 | Were there any other important flaws in the design or statistical methods of the study? | VG | VG | **VG** |  |  |  |  |  |  |
| CONSENSUS | | |  |  | **A** |  |  |  |  |  |  |
| Box 4. Internal consistency | | | | | | | | | | | |
|  |  | Does the scale consist of effect indicators, i.e. is it based on a reflective model? | Y | Y | **Y** | Y | Y | **Y** |  |  |  |
| Design requirements | 1 | Was an internal consistency statistic calculated for each unidimensional scale or subscale separately? | VG | D | **VG** | VG | VG | **VG** |  |  |  |
| Statistical methods | 2 | For continuous scores: Was Cronbach’s alpha or omega calculated? | VG | VG | **VG** | VG | VG | **VG** |  |  |  |
|  | 3 | For dichotomous scores: Was Cronbach’s alpha or KR-20 calculated? | N/A |  | **N/A** | N/A |  | **N/A** |  |  |  |
|  | 4 | For IRT-based scores: Was standard error of the theta (SE (θ)) or reliability coefficient of estimated latent trait value (index of (subject or item) separation) calculated? | N/A |  | **N/A** | N/A |  | **N/A** |  |  |  |
| Other | 5 | Were there any other important flaws in the design or statistical methods of the study? | VG | VG | **VG** | VG | VG | **VG** |  |  |  |
| CONSENSUS | | |  |  | **VG** |  |  | **VG** |  |  |  |
| Box 5. Cross-cultural validity/Measurement invariance | | | | | | | | | | | |
| Design requirements | 1 | Were the samples similar for relevant characteristics except for the group variable? |  |  |  |  |  |  |  |  |  |
| Statistical methods | 2 | Was an appropriate approach used to analyse the data? |  |  |  |  |  |  |  |  |  |
|  | 3 | Was the sample size included in the analysis A? |  |  |  |  |  |  |  |  |  |
|  |  | For Regression analyses or IRT/Rasch based analyses |  |  |  |  |  |  |  |  |  |
|  |  | For MGCFA* |  |  |  |  |  |  |  |  |  |
| Other | 4 | Were there any other important flaws in the design or statistical methods of the study? |  |  |  |  |  |  |  |  |  |
| CONSENSUS | | |  |  |  |  |  |  |  |  |  |
| Box 6. Reliability | | | | | | | | | | | |
| Design requirements | 1 | Were patients stable in the interim period on the construct to be measured? | A | A | **A** | VG | A | **A** | VG | VG | **VG** |
|  | 2 | Was the time interval appropriate? | VG | VG | **VG** | D | VG | **A** | VG | D | **VG** |
|  | 3 | Were the test conditions similar for the measurements? e.g. type of administration, environment, instructions | A | A | **A** | A | A | **A** | A | A | **A** |
| Statistical methods | 4 | For continuous scores: Was an intraclass correlation coefficient (ICC) calculated? | VG | A | **A** | VG | A | **A** | VG | VG | **VG** |
|  | 5 | For dichotomous/nominal/ordinal scores: Was kappa calculated? | N/A |  | **N/A** | N/A |  |  | N/A |  | **N/A** |
|  | 6 | For ordinal scores: Was a weighted kappa calculated? | N/A |  | **N/A** | N/A |  |  | N/A |  | **N/A** |
|  | 7 | For ordinal scores: Was the weighting scheme described? e.g. linear, quadratic | N/A |  | **N/A** | N/A |  |  | N/A |  | **N/A** |
| Other | 8 | Were there any other important flaws in the design or statistical methods of the study? | VG | VG | **VG** | VG | VG | **VG** | VG | VG | **VG** |
| CONSENSUS | | |  |  | **A** |  |  | **A** |  |  | **A** |
| Box 7. Measurement error | | | | | | | | | | | |
| Design requirements | 1 | Were patients stable in the interim period on the construct to be measured? |  |  |  |  |  |  |  |  |  |
|  | 2 | Was the time interval appropriate? |  |  |  |  |  |  |  |  |  |
|  | 3 | Were the test conditions similar for the measurements? e.g. type of administration, environment, instructions |  |  |  |  |  |  |  |  |  |
| Statistical methods | 4 | For continuous scores: Was the Standard Error of Measurement (SEM), Smallest Detectable Change (SDC) or Limits of Agreement (LoA) calculated? |  |  |  |  |  |  |  |  |  |
|  | 5 | For dichotomous/nominal/ordinal scores: Was the percentage (positive and negative) agreement calculated? |  |  |  |  |  |  |  |  |  |
| Other | 6 | Were there any other important flaws in the design or statistical methods of the study? |  |  |  |  |  |  |  |  |  |
| CONSENSUS | | |  |  |  |  |  |  |  |  |  |
| Box 8. Criterion validity | | | | | | | | | | | |
| Statistical methods | 1 | For continuous scores: Were correlations, or the area under the receiver operating curve calculated? |  |  |  |  |  |  |  |  |  |
|  | 2 | For dichotomous scores: Were sensitivity and specificity determined? |  |  |  |  |  |  |  |  |  |
| Other | 3 | Were there any other important flaws in the design or statistical methods of the study? |  |  |  |  |  |  |  |  |  |
| CONSENSUS | | |  |  |  |  |  |  |  |  |  |
| Box 9. Hypotheses testing for construct validity | | | | | | | | | | | |
| 9a. Comparison with other outcome measurement instruments (convergent validity) | | | | | | | | | | | |
| Design requirements | 1 | Is it clear what the comparator instrument(s) measure(s)? | VG | VG | **VG** | VG |  |  |  |  |  |
|  | 2 | Were the measurement properties of the comparator instrument(s) sufficient? | VG | VG | **VG** | VG |  |  |  |  |  |
| Statistical methods | 3 | Was the statistical method appropriate for the hypotheses to be tested? | I | A | **A** | VG |  |  |  |  |  |
| Other | 4 | Were there any other important flaws in the design or statistical methods of the study? | VG | VG | **VG** | VG |  |  |  |  |  |
| CONSENSUS | | |  |  | **A** |  |  |  |  |  |  |
| 9b. Comparison between subgroups (discriminative or known-groups validity) | | | | | | | | | | | |
| Design requirements | 5 | Was an A description provided of important characteristics of the subgroups? |  |  |  | VG | A | **A** |  |  |  |
| Statistical methods | 6 | Was the statistical method appropriate for the hypotheses to be tested? |  |  |  | VG | VG | **VG** |  |  |  |
| Other | 7 | Were there any other important flaws in the design or statistical methods of the study? |  |  |  | VG | VG | **VG** |  |  |  |
| CONSENSUS | | |  |  |  |  |  | **A** |  |  |  |
| Box 10. Responsiveness | | | | | | | | | | | |
| 10a. Criterion approach (i.e. comparison to a gold standard) | | | | | | | | | | | |
| Statistical methods | 1 | For continuous scores: Were correlations between change scores, or the area under the Receiver Operator Curve (ROC) curve calculated? |  |  |  |  |  |  |  |  |  |
|  | 2 | For dichotomous scales: Were sensitivity and specificity (changed versus not changed) determined? |  |  |  |  |  |  |  |  |  |
| Other | 3 | Were there any other important flaws in the design or statistical methods of the study? |  |  |  |  |  |  |  |  |  |
| CONSENSUS | | |  |  |  |  |  |  |  |  |  |
| 10b. Construct approach (i.e. hypotheses testing; comparison with other outcome measurement instruments) | | | | | | | | | | | |
| Design requirements | 4 | Is it clear what the comparator instrument(s) measure(s)? |  |  |  |  |  |  |  |  |  |
|  | 5 | Were the measurement properties of the comparator instrument(s) sufficient? |  |  |  |  |  |  |  |  |  |
| Statistical methods | 6 | Was the statistical method appropriate for the hypotheses to be tested? |  |  |  |  |  |  |  |  |  |
| Other | 7 | Were there any other important flaws in the design or statistical methods of the study? |  |  |  |  |  |  |  |  |  |
| CONSENSUS | | |  |  |  |  |  |  |  |  |  |
| 10c. Construct approach (i.e. hypotheses testing: comparison between subgroups) | | | | | | | | | | | |
| Design requirements | 8 | Was an A description provided of important characteristics of the subgroups? |  |  |  | VG |  |  |  |  |  |
| Statistical methods | 9 | Was the statistical method appropriate for the hypotheses to be tested? |  |  |  | VG |  |  |  |  |  |
| Other | 10 | Were there any other important flaws in the design or statistical methods of the study? |  |  |  | VG |  |  |  |  |  |
| CONSENSUS | | |  |  |  |  |  |  |  |  |  |
| 10d. Construct approach (i.e. hypotheses testing: before and after intervention) | | | | | | | | | | | |
| Design requirements | 11 | Was an A description provided of the intervention given? |  |  |  |  | VG | **VG** |  |  |  |
| Statistical methods | 12 | Was the statistical method appropriate for the hypotheses to be tested? |  |  |  |  | VG | **VG** |  |  |  |
| Other | 13 | Were there any other important flaws in the design or statistical methods of the study? |  |  |  |  | VG | **VG** |  |  |  |
| CONSENSUS | | |  |  |  |  |  | **VG** |  |  |  |
| % | | | **38.71%** | | | **29.63%** | | | **50.00%** | | |

A: adequate; C: consensus; D: doubtful; I: inadequate; N/A: not applicable; R1: Reviewer 1; R2: Reviewer 2; SV: structural validity; U: unidimensionality; VG: very good; Y: yes
