# Supplementary material for: How is the “most bothersome symptom” construct measured across health conditions? A systematic review of patient-reported outcome measures
Source: Patient Relat Outcome Meas. 2026 Sep 20;17(1):107–22. doi: 10.1080/1179271X.2026.2732841 (PMC13596887; doi:10.1080/1179271X.2026.2732841)
Supplement: Supplementary file 5 [file DPRO_A_2732841_SM9474.docx]

**Appendix 1: PRISMA-COSMIN checklist**

| **Title** | |
| --- | --- |
| Title  Item #1: Identify the report as a systematic review and include as applicable the following (in any order): outcome domain of interest, population of interest, name/type of OMIs of interest, and measurement properties of interest. | How is the ‘most bothersome symptom’ construct measured by PROMs across health conditions? A systematic review |
| **Abstract – Open Science** | |
| Item #2.1 | Same as item #1 for full checklist |
| Funding  Item #2.2: Specify the primary source of funding for the review. | All funding sources have been identified in the Declarations section. |
| Registration  Item #2.3: Provide the register name and registration number. | PROSPERO (CRD42022210384) |
| **Abstract – Background** | |
| Objectives  Item #2.4: Provide an explicit statement of the main objective(s) or question(s) the review addresses. | The aims of the review are presented in the Abstract’s Introduction. The four key elements are explicitly described: 1) most bothersome symptom, 2) any health condition, 3) PROMs, 4) all available measurement properties, |
| **Abstract – Methods** | |
| Eligibility criteria  Item #2.5: Specify the inclusion and exclusion criteria for the review. | Inclusion of PROMs is included in the Abstract’s Methods. |
| Information sources  Item #2.6: Specify the information sources (e.g., databases, registers) used to identify studies and the date when each was last searched. | The databases searched are clearly written in the Abstract’s Methods. |
| Risk of bias  Item #2.7: Specify the methods used to assess risk of bias in the included studies. | Risk of bias of included studies was evaluated using the COSMIN ROB checklist. |
| Measurement properties  Item #2.8: Specify the methods used to rate the results of a measurement property. | Measurement properties were evaluated by the COSMIN criteria for good measurement properties |
| Synthesis methods  Item #2.9: Specify the methods used to present and synthesize results. | COSMIN criteria and GRADE criteria were used. |
| **Abstract – Results** | |
| Included studies  Item #2.10: Give the total number of included OMIs and study reports. | 155 PROMs were included relating to 255 articles. |
| Synthesis of results  Item #2.11: Present the syntheses of results of OMIs, indicating the certainty of the evidence. | Overall most PROMs had poor psychometric properties, namely content validity. |
| **Abstract – Discussion** | |
| Limitations of evidence  Item #2.12: Provide a brief summary of the limitations of the evidence included in the review (e.g., study risk of bias, inconsistency, and imprecision). | Poor psychometric properties are common amongst PROMs measuring the ‘most bothersome symptom’. |
| Interpretation  Item #2.13: Provide a general interpretation of the results and important implications. | Implications for further establishment of psychometric properties, especially content validity have been presented. |
| **Plain Language Summary** | |
| Plain language summary  Item #3: If allowed by the journal, provide a plain language summary with background information and key findings. | A plain language has been provided as per the journal’s author guidelines. |
| **Open Science** | |
| Registration and protocol  Item #4a: Provide registration information for the review, including register name and registration number, or state that the review was not registered. | OSF and Prospero registration are reported in the Methods, paragraph 1. |
| Item #4b: Indicate where the review protocol can be accessed, or state that a protocol was not prepared. | OSF and Prospero registration are reported in the Methods, paragraph 1. |
| Item #4c: Describe and explain any amendments to information provided at registration or in the protocol. | Amendments are reported in Appendix 3. |
| Support  Item #5: Describe sources of financial or non-financial support for the review, and the role of the funders in the review. | All funding sources have been identified in the Declarations section. |
| Competing interests  Item #6: Declare any competing interests of review authors. | All competing interests have been identified in the Declarations section. |
| Availability of data, code, and other materials  Item #7: Report which of the following are publicly available and where they can be found: template data collection forms; data extracted from included studies; data used for all analyses; analytic code; any other materials used in the review. | All relevant data are presented within the paper, appendices, or supporting files. |
| **Introduction** | |
| Rationale  Item #8: Describe the rationale for the review in the context of existing knowledge. | Rationale provided in that the ‘most bothersome symptom’ outcome is emerging in core outcome sets and is recommended by the FDA for drug trials for menopause and migraine, yet little is known about PROMs measuring it. |
| Objectives  Item #9: Provide an explicit statement of the objective(s) or question(s) the review addresses and include as applicable the following (in any order): outcome domain of interest, population of interest, name/type of OMIs of interest, and measurement properties of interest. | The review question directly addresses the four key components and is outlined in the aims written in the final paragraph. |
| **Methods** | |
| Followed guidelines  Item #10: Specify, with references, the methodology and/or guidelines used to conduct the systematic review. | COSMIN guidelines were followed to conduct this review. |
| Eligibility criteria  Item #11: Specify the inclusion and exclusion criteria for the review. | Eligibility criteria are outlined in section 2.1 |
| Information sources  Item #12: Specify all databases, registers, preprint servers, websites, organizations, reference lists and other sources searched or consulted to identify studies. Specify the date when each source was last searched or consulted. | Databases searched are outlined in section 2.2 |
| Search strategy  Item #13: Present the full search strategies for all databases, registers, and websites, including any filters and limits used. | Search strategy presented in Appendix 2. |
| Selection process  Item #14: Specify the methods used to decide whether a study met the inclusion criteria of the review, e.g., including how many reviewers screened each record and each report retrieved, whether they worked independently, and if applicable, details of automation tools/AI used in the process. | Described in section 2.3 |
| Data collection process  Item #15: Specify the methods used to collect data from reports, e.g., including how many reviewers collected data from each report, whether they worked independently, any processes for obtaining or confirming data from study investigators, and if applicable, details of automation tools/AI used in the process. | Described in section 2.4 |
| Data items  Item #16: List and define which data were extracted (e.g., characteristics of study populations and OMIs, measurement properties’ results, and aspects of feasibility and interpretability). Describe methods used to deal with any missing or unclear information. | Described in section 2.4 |
| Study risk of bias assessment  Item #17: Specify the methods used to assess risk of bias in the included studies, e.g., including details of the tool(s) used, how many reviewers assessed each study and whether they worked independently, and if applicable, details of automation tools/AI used in the process. | Described in section 2.5.1 |
| Measurement properties  Item #18: Specify the methods used to rate the results of a measurement property for each individual study and for the summarized or pooled results, e.g., including how many reviewers rated each study and whether they worked independently. | Described in section 2.5.2 |
| Synthesis methods  Item #19a: Describe the processes used to decide which studies were eligible for each synthesis. | Described in section 2.5.2 |
| Item #19b: Describe any methods used to synthesize results. | Described in section 2.5.2 |
| Item #19c: If applicable, describe any methods used to explore possible causes of inconsistency among study results (e.g., subgroup analysis). | Not applicable to this review. |
| Item #19d: If applicable, describe any sensitivity analyses conducted to assess robustness of the synthesized results. | Not applicable to this review. |
| Certainty assessment  Item #20: Describe any methods used to assess certainty (or confidence) in the body of evidence. | Described in 2.5.3 |
| Formulating recommendations  Item #21: If appropriate, describe any methods used to formulate recommendations regarding the suitability of OMIs for a particular use. | Not applicable to this review. |
| **Results** | |
| Study selection  Item #22a: Describe the results of the search and selection process, from the number of records identified in the search to the number of study reports included in the review, ideally using a flow diagram. If applicable, also report the final number of OMIs included and the number of study reports relevant to each OMI. | Presented in section 3.1 and Figure 1. |
| Item #22b: Cite study reports that might appear to meet the inclusion criteria, but which were excluded, and explain why they were excluded. | A list of excluded PROMs and the reasons for exclusion are available on request. |
| OMI characteristics  Item #23a: Present characteristics of each included OMI, with appropriate references. | Characteristics of the included PROMs are presented in Table 2, and Supplementary Tables S2, S4 – S13. |
| Item #23b: If applicable, present interpretability aspects for each included OMI. | Not applicable to this review. |
| Item #23c: If applicable, present feasibility aspects for each included OMI. | Average time for completion can be found in Supplementary Tables S2, S4-S13. |
| Study characteristics  Item #24: Cite each included study report evaluating one or more measurement properties and present its characteristics. | Information is presented in Supplementary Table S17. |
| Risk of bias in studies  Item #25: Present assessments of risk of bias for each included study. | Presented in Table 3. |
| Results of individual studies  Item #26: For all measurement properties, present for each study: (a) the reported result and (b) the rating against quality criteria, ideally using structured tables or plots. | Described in Appendix 6. |
| Results of syntheses  Item #27a: Present results of all syntheses conducted. For each measurement property of an OMI, present: (a) the summarized or pooled result and (b) the overall rating against quality criteria. | Described in Appendix 6. |
| Item #27b: If applicable, present results of all investigations of possible causes of inconsistency among study results. | Not applicable to this review. |
| Item #27c: If applicable, present results of all sensitivity analyses conducted to assess the robustness of the synthesized results. | Not applicable to this review. |
| Certainty of evidence  Item #28: Present assessments of certainty (or confidence) in the body of evidence for each measurement property of an OMI assessed. | Presented in Table 3. |
| Recommendations  Item #29: If appropriate, make recommendations for suitable OMIs for a particular use. | Recommendations for MQOL, QUAL-EC, and PSYCHLOPS have been discussed. |
| **DISCUSSION** | |
| Discussion  Item #30a: Provide a general interpretation of the results in the context of other evidence. | Described in paragraphs 2, 3, 6, 7, 9. |
| Item #30b: Discuss any limitations of the evidence included in the review. | Described in paragraphs 2, 4, 5, 6, 8. |
| Item #30c: Discuss any limitations of the review processes used. | Described in paragraph 9. |
| Item #30d: Discuss implications of the results for practice, policy, and future research. | Described in paragraphs 2, 3, 4, 5, 6, 8, 9. |

**Appendix 2: Search strategies**

Database: Ovid MEDLINE(R) Epub Ahead of Print, In Process & Other Non-Indexed Citations, Ovid MEDLINE (R) Daily, and Ovid MEDLINE (R) 1946-10Jan2022

Search Strategy:

--------------------------------------------------------------------------------

1	exp "Surveys and Questionnaires"/ (1143571)

2	exp "reproducibility of results"/ (437206)

3	(questionnaire* adj3 (health or bother* or symptom* or measure* or validat* or evaluat* or assess* or reliability or reproducibility)).tw. (118362)

4	(questionnaire* adj3 Quality of life).tw. (16499)

5	(instrument* adj3 (health or bother* or symptom* or measure* or validity or validation or assess* or reliability or reproducibility)).tw. (43277)

6	(brief form adj3 (questionnaire* or survey* or assess* or validation or validity or health or measure* or evaluat*)).tw. (57)

7	(short form adj3 (questionnaire* or survey* or assess* or validation or validity or health or measure* or evaluat*)).tw. (20933)

8	(Scale* adj3 (measure* or assessment or development or analog* or evaluat*)).tw. (123157)

9	or/1-8 (1693461)

10	((evaluat* or measur*) adj2 severity).tw. (13592)

11	symptom*.tw. (1272923)

12	10 and 11 (3790)

13	9 or 12 (1695135)

14	((bothersome or bother or bothering or troublesome or troubling or worrisome or worrying or annoy* or upset* or burden*) adj4 (symptom or symptoms or problem*)).tw. (12529)

15	13 and 14 (4886)

16	exp animals/ not humans.sh. (4932094)

17	15 not 16 (4884)

Database: Embase <1980 to 2022 Week 2>

Search Strategy:

--------------------------------------------------------------------------------

1	questionnaire/ (780063)

2	exp reproducibility/ (235995)

3	(questionnaire* adj3 (health or bother* or symptom* or measure* or validat* or evaluat* or assess* or reliability or reproducibility)).tw. (175216)

4	(questionnaire* adj3 Quality of life).tw. (26448)

5	(instrument* adj3 (health or bother* or symptom* or measure* or validity or validation or assess* or reliability or reproducibility)).tw. (53975)

6	(brief form adj3 (questionnaire* or survey* or assess* or validation or validity or health or measure* or evaluat*)).tw. (68)

7	(short form adj3 (questionnaire* or survey* or assess* or validation or validity or health or measure* or evaluat*)).tw. (28301)

8	(Scale* adj3 (measure* or assessment or development or analog* or evaluat*)).tw. (172758)

9	or/1-8 (1248698)

10	((evaluat* or measur*) adj2 severity).tw. (21468)

11	symptom*.tw. (1833866)

12	10 and 11 (6492)

13	9 or 12 (1252792)

14	((bothersome or bother or bothering or troublesome or troubling or worrisome or worrying or annoy* or upset* or burden*) adj4 (symptom or symptoms or problem*)).tw. (21946)

15	13 and 14 (6643)

Database: APA PsycInfo <1806 to November Week 2 2022>

Search Strategy:

--------------------------------------------------------------------------------

1	((bothersome or bother or bothering or troublesome or troubling or worrisome or worrying or annoy* or upset* or burden*) adj4 (symptom or symptoms or problem*)).tw. (3744)

Database: PROQOLID <10 January 2022>

Search Strategy:

--------------------------------------------------------------------------------

1	bother* or trouble* or worry or annoy* or upset* or burden*

**Ovid MEDLINE(R) ALL, May 18^th^ 2026**

1	exp "Surveys and Questionnaires"/	1382255

2	exp "reproducibility of results"/	513259

3	(questionnaire* adj3 (health or bother* or symptom* or measure* or validat* or evaluat* or assess* or reliability or reproducibility)).tw.	173477

4	(questionnaire* adj3 Quality of life).tw.	23237

5	(instrument* adj3 (health or bother* or symptom* or measure* or validity or validation or assess* or reliability or reproducibility)).tw.	58008

6	(brief form adj3 (questionnaire* or survey* or assess* or validation or validity or health or measure* or evaluat*)).tw.	90

7	(short form adj3 (questionnaire* or survey* or assess* or validation or validity or health or measure* or evaluat*)).tw.	30504

8	(Scale* adj3 (measure* or assessment or development or analog* or evaluat*)).tw.	179824

9	or/1-8	2084889

10	((evaluat* or measur*) adj2 severity).tw.	19172

11	symptom*.tw.	1703487

12	10 and 11	5552

13	9 or 12	2087579

14	((bothersome or bother or bothering or troublesome or troubling or worrisome or worrying or annoy* or upset* or burden*) adj4 (symptom or symptoms or problem*)).tw.	21259

15	13 and 14	7813

16	exp animals/ not humans.sh.	5453972

17	15 not 16	7810

18	17	7810

19	limit 17 to yr="2022 -Current"	2944

20	reliability.mp.	279815

21	validity.mp.	259087

22	"measurement propert*".mp.	5053

23	"psychometric propert*".mp. [mp=title, book title, abstract, original title, name of substance word, subject heading word, floating sub-heading word, keyword heading word, organism supplementary concept word, protocol supplementary concept word, rare disease supplementary concept word, unique identifier, synonyms, population supplementary concept word, anatomy supplementary concept word]	41112

24	20 or 21 or 22 or 23	462302

25	19 and 24	230

**Embase <1974 to 2026 May 18>, 18^th^ May 2026**

1	questionnaire/	1178449

2	exp reproducibility/	304379

3	(questionnaire* adj3 (health or bother* or symptom* or measure* or validat* or evaluat* or assess* or reliability or reproducibility)).tw.	270399

4	(questionnaire* adj3 Quality of life).tw.	41987

5	(instrument* adj3 (health or bother* or symptom* or measure* or validity or validation or assess* or reliability or reproducibility)).tw.	74279

6	(brief form adj3 (questionnaire* or survey* or assess* or validation or validity or health or measure* or evaluat*)).tw.	113

7	(short form adj3 (questionnaire* or survey* or assess* or validation or validity or health or measure* or evaluat*)).tw.	42345

8	(Scale* adj3 (measure* or assessment or development or analog* or evaluat*)).tw.	264409

9	or/1-8	1828745

10	((evaluat* or measur*) adj2 severity).tw.	31758

11	symptom*.tw.	2641735

12	10 and 11	10119

13	9 or 12	1835007

14	((bothersome or bother or bothering or troublesome or troubling or worrisome or worrying or annoy* or upset* or burden*) adj4 (symptom or symptoms or problem*)).tw.	39233

15	13 and 14	11849

16	limit 15 to yr="2022 -Current"	4642

17	reliability.mp.	458768

18	validity.mp.	358095

19	"measurement propert*".mp.	6283

20	"psychometric propert*".mp. [mp=title, abstract, heading word, drug trade name, original title, device manufacturer, drug manufacturer, device trade name, keyword heading word, floating subheading word, candidate term word]	46522

21	17 or 18 or 19 or 20	709557

22	16 and 21	341

**APA PsycInfo <1806 to May 2026 Week 2>, May 18^th^ 2026**

1	((bothersome or bother or bothering or troublesome or troubling or worrisome or worrying or annoy* or upset* or burden*) adj4 (symptom or symptoms or problem*)).tw.	5481

2	limit 1 to yr="2022 -Current"	1567

3	reliability.mp.	140623

4	validity.mp.	219576

5	"measurement propert*".mp.	2047

6	"psychometric propert*".mp. [mp=title, abstract, heading word, table of contents, key concepts, original title, tests & measures, mesh word]	39777

7	3 or 4 or 5 or 6	274398

8	2 and 7	85

**Appendix 3: Changes to study protocol**

During the screening process, the research team discussed the review inclusion/exclusion criteria and progress which resulted in several clarifications and modifications to the review protocol. The following PROMs became ineligible: those which solely measured side effects or adverse events of treatments, and those which used the term ‘problem’ (as a potential synonym for ‘bother’). Originally, we intended to include all studies developing, validating, or using eligible PROMs. However, due to the sheer number of identified studies and the lack of useful information in studies using PROMs, we elected to restrict inclusion to development/validation studies where applicable, with the inclusion of a single representative study using the PROM only when no development/validation study existed.

During screening of titles and abstracts, inclusion criteria were amended to only include studies describing development and/or validation if they were found, or only one representative study which used a PROM as part of its methodology if there was an absence of a development or psychometric paper. For PROMs where only a representative study was found during screening, an external search was conducted for the original development and/or validation paper.
